# The miR-29 transcriptome in endocrine-sensitive and resistant breast cancer cells

**DOI:** 10.1038/s41598-017-05727-w

**Published:** 2017-07-12

**Authors:** Penn Muluhngwi, Negin Alizadeh-Rad, Stephany L. Vittitow, Ted S. Kalbfleisch, Carolyn M. Klinge

**Affiliations:** Department of Biochemistry & Molecular Genetics, Center for Genetics and Molecular Medicine University of Louisville, Louisville, KY 40292 USA

## Abstract

Aberrant microRNA expression contributes to breast cancer progression and endocrine resistance. We reported that although tamoxifen stimulated miR-29b-1/a transcription in tamoxifen (TAM)-resistant breast cancer cells, ectopic expression of miR-29b-1/a did not drive TAM-resistance in MCF-7 breast cancer cells. However, miR-29b-1/a overexpression significantly repressed TAM-resistant LCC9 cell proliferation, suggesting that miR-29b-1/a is not mediating TAM resistance but acts as a tumor suppressor in TAM-resistant cells. The target genes mediating this tumor suppressor activity were unknown. Here, we identify miR-29b-1 and miR-29a target transcripts in both MCF-7 and LCC9 cells. We find that miR-29b-1 and miR-29a regulate common and unique transcripts in each cell line. The cell-specific and common downregulated genes were characterized using the MetaCore Gene Ontology (GO) enrichment analysis algorithm. LCC9-sepecific miR-29b-1/a-regulated GO processes include oxidative phosphorylation, ATP metabolism, and apoptosis. Extracellular flux analysis of cells transfected with anti- or pre- miR-29a confirmed that miR-29a inhibits mitochondrial bioenergetics in LCC9 cells. qPCR,luciferase reporter assays, and western blot also verified the ATP synthase subunit genes *ATP5G1* and *ATPIF1* as *bone fide* miR29b-1/a targets. Our results suggest that miR-29 repression of TAM-resistant breast cancer cell proliferation is mediated in part through repression of genes important in mitochondrial bioenergetics.

## Introduction

microRNAs (miRNA, miRs) are 22 nt non-coding RNAs that recognize and bind complementary seed sequences in the 3′-UTR region of a target messenger RNA (mRNA)^1, 2^. This results in translational repression and/or transcriptional degradation of the target gene. By targeting several mRNAs, miRNAs regulate several cellular and biological processes including cell cycle, cell proliferation and cell differentiation, apoptosis, cellular respiration and glycolysis (reviewed in refs 3–6). Aberrant miRNA expression mediates disease initiation and progression in breast and other cancers^7^.

Seventy percent of breast tumors express estrogen receptor alpha (ERα) implying eligibility for endocrine therapies including selective estrogen receptor modulators (SERMs), *e*.*g*., Tamoxifen (TAM), and aromatase inhibitors (AI), *e*.*g*., letrozole. The efficacy of endocrine therapy is limited by relapse in ~40% of patients^8, 9^. Endocrine resistance is mediated by multiple mechanisms depending on the type of therapy used (reviewed in refs 10–14). Alterations in miRNA expression are also implicated in endocrine-resistance (reviewed in refs 15 and 16).

Previously, we reported miR-29b-1 and miR-29a were downregulated by TAM in TAM- sensitive (TAM-S) MCF-7 BC cells and upregulated in TAM-resistant (TAM-R) LCC2, LCC9, and LY2 BC cells^17^. There are four miR-29 family members in the human genome: miR-29b-2 and miR-29c (chromosome 1q32.2) and miR-29b-1 and miR-29a (chromosome 7q32.3). miR-29b-1 and miR-29a are separated by ~652 bp on the same pri-miRNA transcript^18–21^. Upon processing, mature miR-29b-1 preferentially localizes to the nucleus, while mature miR-29a localizes to the cytoplasm^22^. miR-29 family members have been reported to exhibit both tumor suppressive and oncogenic roles in breast cancer^17, 23–25^.

We recently reported that ERα mediates 4-hydroxyTAM (4-OHT) repression of miR-29b-1/a in MCF-7 and upregulation in TAM-R LCC9 and LY2 BC cells. However, inhibition of miR-29b-1 and miR-29a did not sensitize LCC9 or LY2 cells to TAM^17^. Ectopic expression of miR-29b-1/a did not drive TAM resistance in MCF-7 cells, but it did significantly repress proliferation of TAM-R LCC9 and LY2 cells as compared to TAM-S MCF-7 cells. This suggests that miR-29b-1/a may regulate different transcripts, and thus pathways, in TAM-R cells and TAM-S cells. To identify and quantitate mRNA targets regulated by miR-29, we performed RNA-sequencing (RNA-seq) in MCF-7 and LCC9 BC cells transiently transfected with anti-miR-29a, pre-miR-29b-1 or pre-miR-29a. MetaCore^TM^ functional analysis of the RNA-seq data identified several metabolic processes including oxidative phosphorylation (OXPHOS) uniquely regulated by both miR-29b-1 and miR-29a in LCC9 cells. We observed that miR-29a overexpression inhibited mitochondrial bioenergetic function in LCC9 cells, thus correlating function with identified Gene Ontology (GO) processes. We used qPCR, a luciferase reporter assay, and western blot to validate that two subunits of ATP synthase (Complex V in the oxidative phosphorylation respiratory chain), *ATP5G1* and *ATPIF1*, are *bona fide* miR-29b-1/a targets.

## Results

### Identification and characterization of the miR-29b-1 and miR-29a transcriptome in MCF-7 and LCC9 cells

To identify potential miR-29b-1 and miR-29a targets in breast cancer cells and their possible role in tumor suppression, TAM-S MCF-7 and TAM-R LCC9 cells were grown in hormonally depleted medium and transfected with anti-miR-29a (that inhibits both miR-29b-1 and miR-29a expression) and pre-miR-29b-1 or pre-miR-29a (Supplementary Fig. 1). The strategy of comparing the transcriptome of cells transfected with anti-miR-29a *versus* pre-miR-29b-1 and anti-miR-29a *versus* pre-miR-29a was selected to enrich for direct miR-29b-1 and miR-29a targets. As miRNAs historically repress target transcript translation and/or mRNA expression, we focused on genes downregulated with pre-miRNA treatment and upregulated with anti-miR treatment for transcriptome analysis.

**Figure 1:**
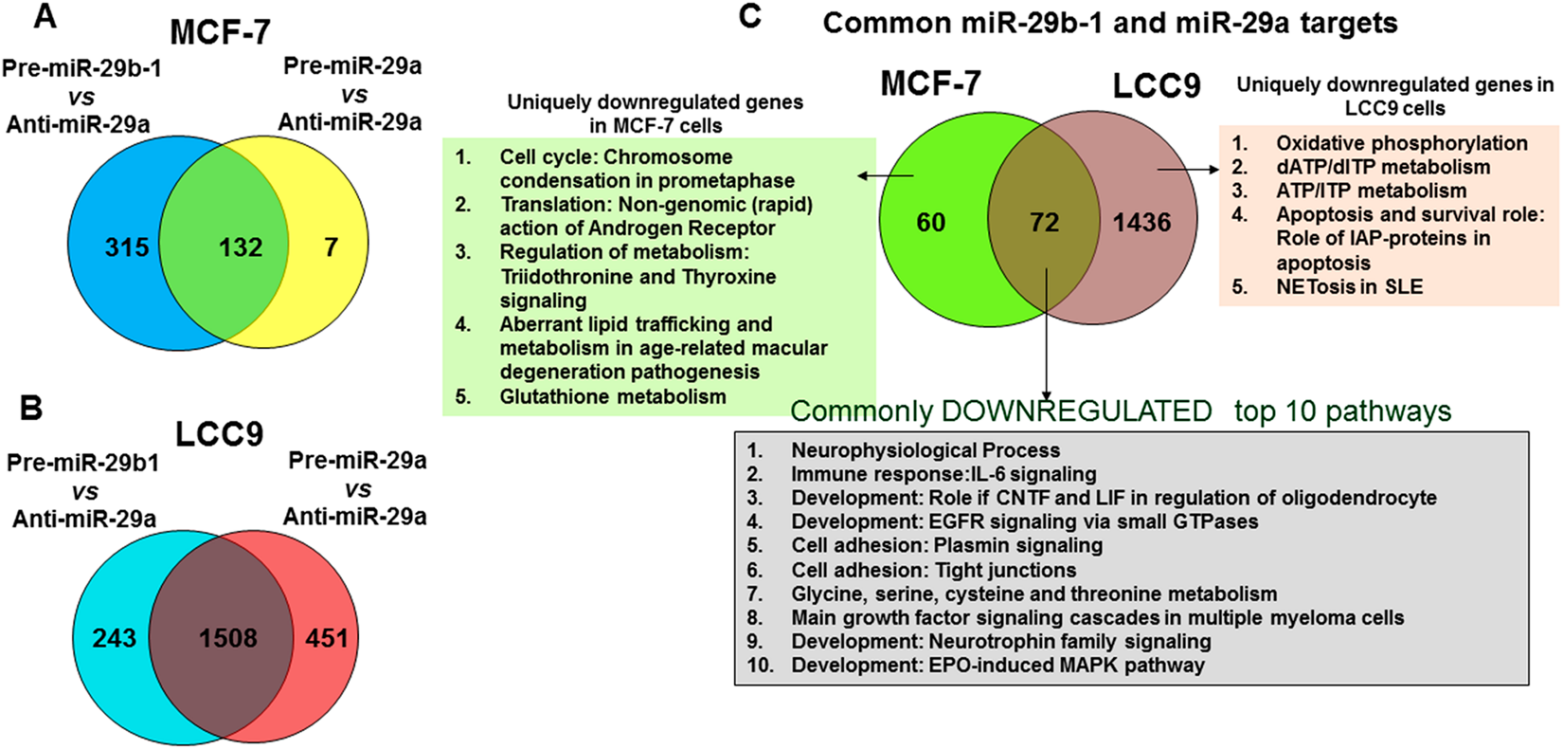
Enrichment analysis of RNA-seq data. Differentially expressed genes were identified in pairwise comparisons: Pre-miR-29b-1 *vs*. Anti-miR-29a and Anti-miR-29a vs. Pre-miR-29a using tophat and cufflink-cuff diff2. The Venn diagrams show the number of differentially and commonly regulated genes identified. GeneGo Pathways Software (MetaCore^TM^) was then used to identify genes significantly downregulated by miR-29b-1 and miR-29a in (**A**) MCF-7 and (**B**) LCC9 cells. (**C**) Pathway analysis of the downregulated genes was performed using MetaCore^TM^. The order of the pathways identified for each comparison are listed in the order provided by MetaCore^TM^ analysis.

miR-29b-1 and miR-29a downregulated 447 and 139 genes in MCF-7 cells respectively (Fig. 1A) and 1,751 and 1,959 genes in LCC9 cells (Fig. 1B; p < 0.05), respectively. The identity of these genes with MetaCore pathway analysis is provided in Supplementary Tables 1 and 2. These data suggest that miR-29b-1 and miR-29a regulate more transcripts in TAM-R LCC9 cells compared to TAM-S MCF-7 cells.

In MCF-7 cells, 132 genes were identified as common targets of both miR-29b-1 and miR-29a (Fig. 1A). In LCC9 cells, 1,508 genes were common targets to both miR-29b-1 and miR-29a (Fig. 1B). MetaCore^TM^ pathway analysis of the common and uniquely regulated genes is provided in Supplementary Figs 2 and 3. Of the common targets, 60 were unique to MCF-7 and 1,436 were unique to LCC9 (Fig. 1A and Supplementary Tables 3). 72 targets were common to both MCF-7 and LCC9 cells (Fig. 1C). Together, these data confirm that miR-29b-1 and miR-29a regulate common and unique targets in MCF-7 and LCC9 cells. MetaCore^TM^ pathway enrichment analysis was performed for each dataset and the top pathways identified are listed in Fig. 1C.

Gene ontology (GO) analysis of the uniquely downregulated transcripts by both miR-29b-1 and miR-29a in LCC9 cells (Fig. 1C) identified organonitrogen compound metabolic and biosynthetic processes and cotranslational protein targeting to the membrane as the top three regulated processes (Supplementary Fig. 4A). The top five regulated process networks included translation initiation, elongation, termination, and cell cycle S phase (Supplementary Fig. 4B).

Pathway enrichment analysis of the uniquely downregulated miR-29b-1/a target genes in LCC9 cells identified pathways including oxidative phosphorylation, ATP metabolism, apoptosis, and cell survival. MetaCore^TM^ network analysis on mitochondrial respiratory complex genes downregulated by miR-29b-1/a is shown in Supplementary Fig. 4C. Several genes including components of respiratory Complex II and III, Cytochrome *c* oxidase, and ATP synthase (Complex IV) were uniquely downregulated by miR-29b-1/a in LCC9 cells (Supplementary Fig. 4C). We previously reported that six genes encoding subunits of ATP synthase that are not regulated by miR-29 were more highly expressed in LCC9 than MCF-7 cells^26^. The role of these genes in endocrine resistance will require further evaluation.

### miR-29a regulates mitochondrial bioenergetics in tamoxifen-resistant LCC9 cells

Because OXPHOS was identified as the top enrichment pathway modulated by miR-29b-1/a in LCC9 cells, and not in MCF-7 cells (Fig. 1C), we postulated that miR-29a regulates mitochondrial bioenergetics activity in LCC9 cells. We focused on miR-29a because its basal expression is higher in LCC9 and other breast cancer cells compared to miR-29b-1^17^. To examine the impact of miR-29a on mitochondrial respiration, MCF-7 and LCC9 cells were transfected with either anti-miR-29a or pre-miR-29a, *versus* control, and grown in hormonally depleted phenol red-free medium for 48 h prior to determining oxygen consumption rate (OCR) and extracellular acidification rate (ECAR; reflects glycolytic rate)^27^ using the Seahorse extracellular flux assay^26, 27^ (Supplementary Fig. 5).

**Figure 2:**
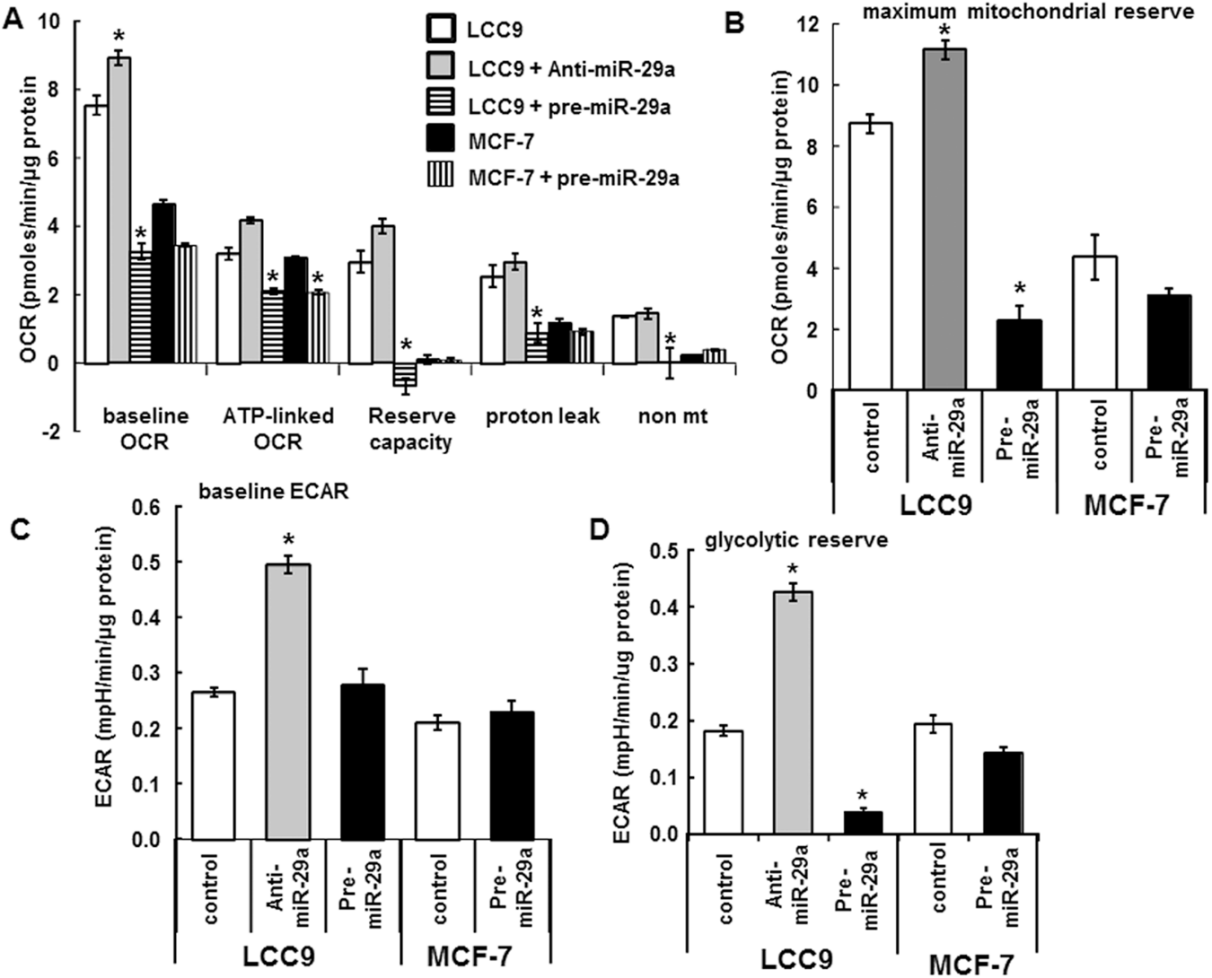
Upregulation of miR-29a represses mitochondrial function in MCF-7 and LCC9 cells. MCF7 and LCC9 cells were plated in XF-24 plates in hormonally depleted medium and transfected with control, anti-miR-29a, or pre-miR-29a for 28 h prior to running the Seahorse extracellular flux assay to determine mitochondrial activity. (**A** and **B**) OCR parameters; (**C** and **D**) ECAR parameters. Each bar is an average of 4 separate wells ± SEM in one experiment. *p < 0.05 *versus* LCC9 or MCF-7 control for the indicated parameter. For LCC9, data were analyzed by one-way ANOVA followed by Tukey’s test. For MCF-7, two-tailed Student’s t-test was performed (NS).

In agreement with our previous work^26^, TAM-R LCC9 cells grown in hormonally depleted medium have increased basal OCR *versus* MCF-7 cells (Fig. 2A). Transfection of LCC9 cells with anti-miR-29b-1 increased while pre-miR-29a repressed basal OCR, ATP-linked OCR (which measures the rate of mitochondrial ATP synthesis) and mitochondrial reserve (also known as reserve respiratory capacity)^28^ (Fig. 2A). Similar results were seen in pre-miR-29a-transfected MCF-7 cells except that growth in hormonally depleted medium ablates reserve capacity, and thus, no effect of miR-29a on reserve capacity was detected (Fig. 2A). The changes in basal OCR suggest that miR-29a modulates metabolic rate in part through electron flow. The regulation of reserve capacity, which is a measurement of the ability to respond to increased energy demand after injection of the uncoupler FCCP, suggests that miR-29-regulated genes adversely impact LCC9’s mitochondrial ability to mitigate cellular stress. Inverse changes, albeit of lesser magnitude, were seen in LCC9 cells transfected with anti-miR-29a.

Compared to MCF-7 cells, LCC9 cells have higher basal proton leak. Proton leak is required for protecting against cellular oxidative damage through lowering ROS produced by the electron transport chain (ETC)^29^. Pre-miR-29a transfection repressed proton leak in LCC9 cells (Fig. 2A). Non-mitochondrial activity, which is attributed to cytoplasmic oxidases, was lower in LCC9 cells transfected with pre-miR-29a (Fig. 2A).

Transfection of LCC9 cells with anti-miR-29a increased while pre-miR-29a transfection decreased maximal mitochondrial (mt) capacity (Fig. 2B). The maximal mitochondrial capacity is the OCR measured after injection of the uncoupler FCCP and is indicative of the mt ability to take up substrates^30^.

We previously reported that ECAR measurements agree with conversion of radio-labelled glucose to H_2_O in MCF-7 and T47D breast cancer cells, demonstrating that ECAR is a reliable measure of glycolysis^27^. LCC9 cells alone or upon transfection with anti-miR-29a had increased basal ECAR than MCF-7 cells (Fig. 2C). The glycolytic response to oligomycin, considered as the glycolytic reserve^31^, was increased by miR-29a repression and reduced by miR-29a overexpression in LCC9 cells (Fig. 2D). These data suggest that miR-29a regulates genes with roles in glycolysis in LCC9 cells. Examination of the RNA-seq data identified *TPI1*, *PGAM1*, *ENO1*, and *LDHA* as possible targets of miR-29b-1/a (Supplementary Fig. 6A). qPCR analysis confirmed that overexpression of miR-29b-1/a inhibited *ENO1* and *LDHA* transcript expression in MCF-7 and LCC9 cells (Supplementary Fig. 6B). Future studies will further evaluate these observations.

### Validation of ATP5G1 and ATPIF1 as bona fide targets of miR-29b-1 and miR-29a

**Figure 3:**
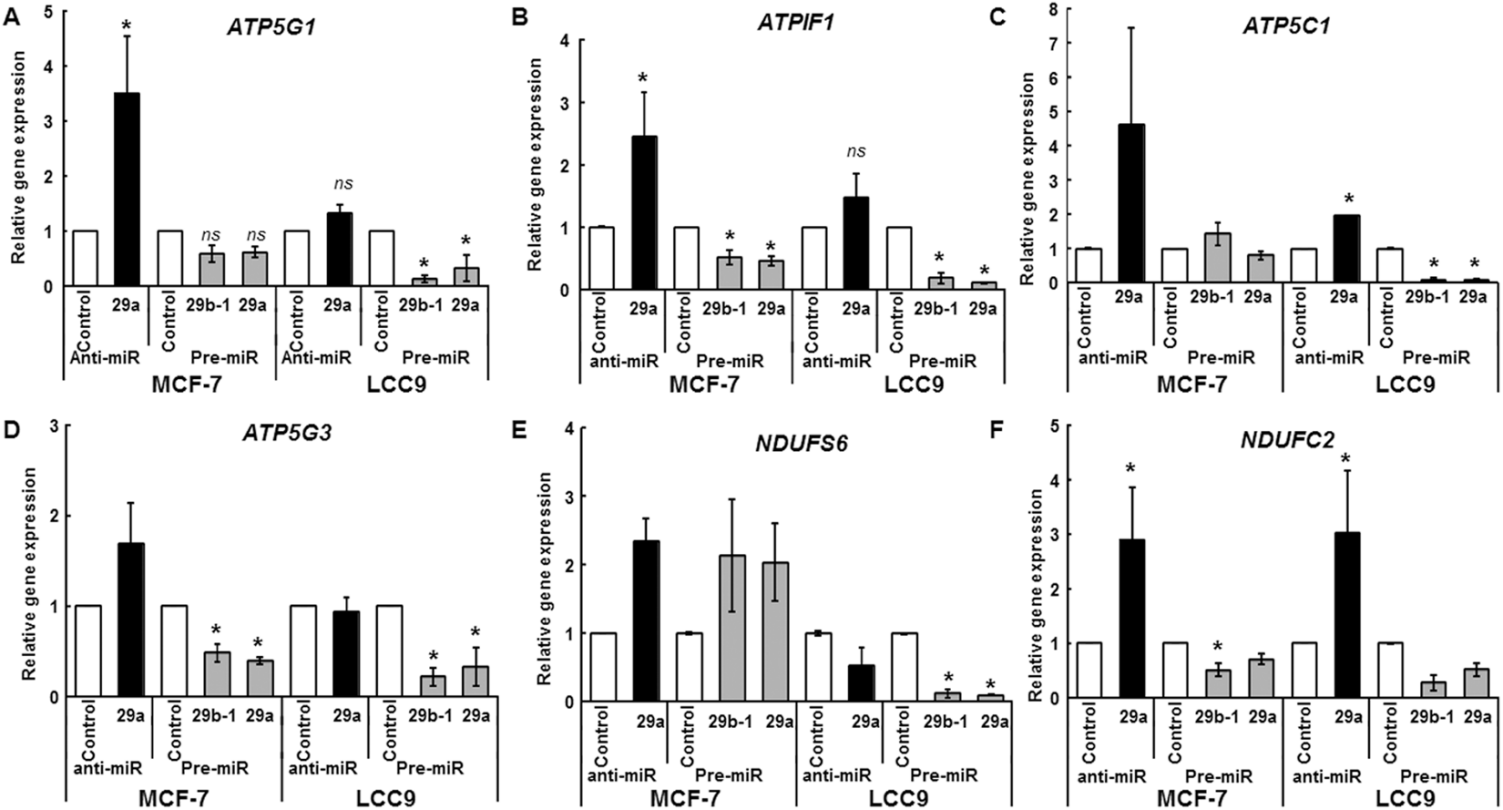
miR-29b-1/a regulates mRNA expression of putative targets in MCF-7 and LCC9 cells. MCF-7 and LCC9 cells were grown in ‘hormone-depleted’ media and transfected with anti-miR-control, anti-miR-29a (3p), pre-miR control or pre-miR-29b-1/a (3p), as indicated. Twenty-four h post transfection, RNA was isolated and qPCR performed. Values were normalized to *18S*. Values are the mean ± SEM of 3 independent experiments. Within each experiment, each sample was run in triplicates. Statistical evaluation was performed using one-way ANOVA followed by Tukey’s test. *p < 0.05 *versus* control transfected cells.

miR-29a regulation of mitochondrial activity is suggestive of direct regulation of mitochondrial complex genes. Analysis of our RNA-seq data revealed miR-29 regulation of mitochondrial genes including *ATP5G1*, *ATP5C1*, *ATPIF1*, *ATP5G3*, *NDUFS6*, and *NDUFC2* (Supplementary Fig. 7). To confirm miR-29 regulation of these putative targets, qPCR was performed in MCF-7 and LCC9 cells transfected with either anti-miR-29a, pre-miR-29b-1, pre-miR-29a or their negative controls (Fig. 3). Anti-miR-29a transfection increased *ATP5G1* and *ATPIF1* mRNA expression in MCF-7 and LCC9 cells (Fig. 3A and B). Conversely, pre-miR-29b-1 and pre-miR-29a transfection repressed *ATP5G1* in LCC9 cells alone and *ATPIF1* mRNA expression in both MCF-7 and LCC9 cells (Fig. 3A and B). These data suggest regulation of *ATP5G1* and *ATPIF1* by miR-29b-1/a.

In LCC9 cells, anti-miR-29a transfection increased A*TP5C1* and *NDUFC2* while pre-miR-29b-1 and pre-miR-29a transfection repressed *ATP5C1*, *ATP5G3*, *NDUFS6* and *NDUFC2* transcript expression (Fig. 3C–F). In MCF-7 cells anti-miR-29a transfection increased *NDUFC2* expression while pre-miR-29b-1 and pre-miR-29a transfection repressed expression of *ATP5G3* and *NDUFC2* transcripts (Fig. 3D and F). Taken together, these data suggest cell line specific differential regulation of miR-29 targets between MCF-7 and LCC9 cells. Notably, *ATP5G1* and *ATP1F1* were downregulated by miR-29b-1/a in both LCC9 and MCF-7 cells (Fig. 3A and B). Further, basal mRNA expression of *ATP5G1*, *ATP5IF1* and *ATP5G3* were higher (lower CT) relative to *ATP5IC1*, *NDUFS6*, and *NDUFC2* in both MCF-7 and LCC9 cells (Supplementary Fig. 8). Hence, we selected *ATP5G1* and *ATPIF1* for further study.

**Figure 4:**
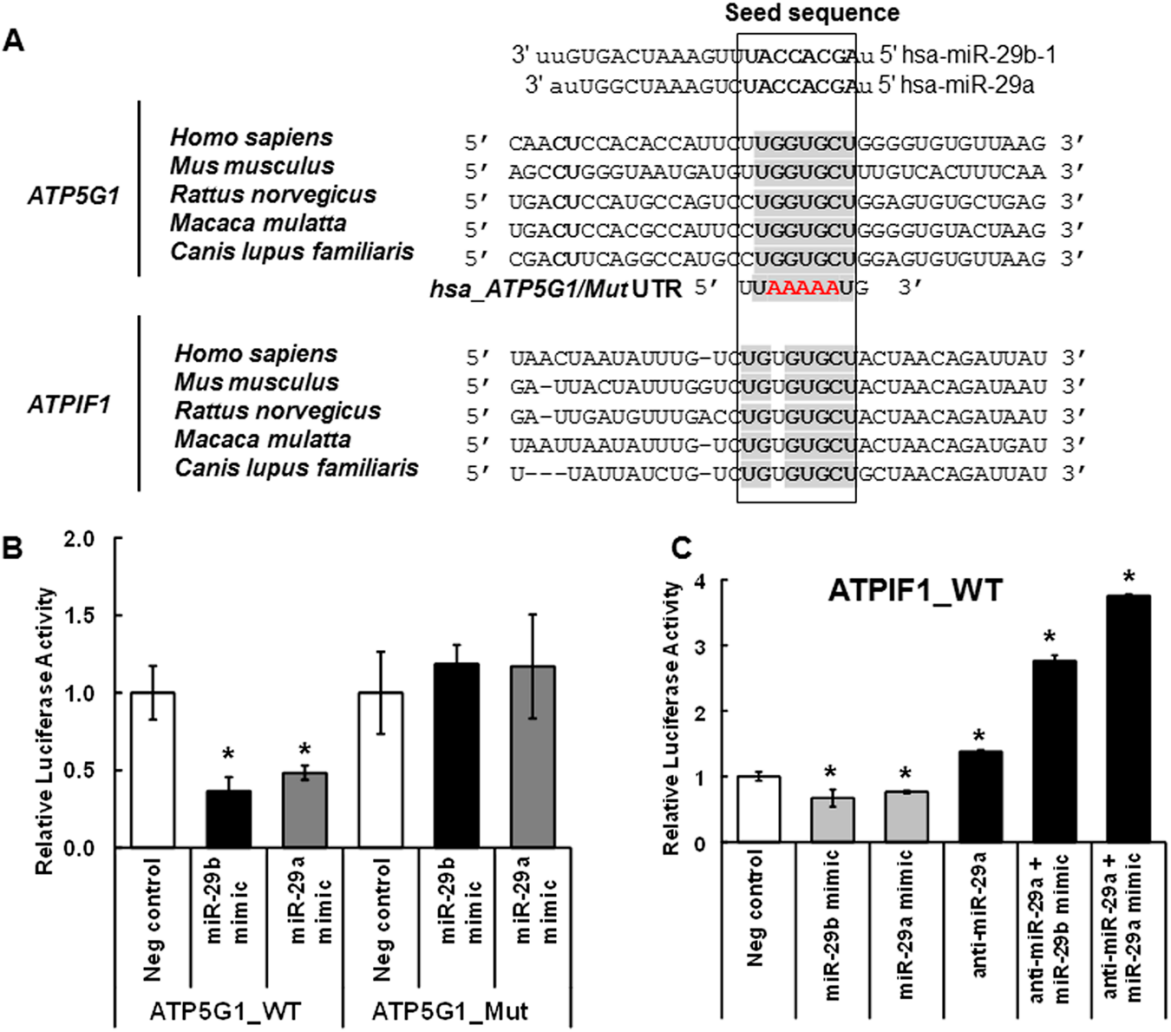
miR-29b-1/a regulate *ATP5G1* and *ATPIF1* 3′-UTRs luciferase reporter activity. (**A**) Alignment of miR-29b-1 and miR-29a, their seed elements (in bold) and the 3′-UTRs of *ATP5G1* and *ATPIF1*. The mutated seed element in *ATP5G1* used in luciferase assay is indicated in red. miR-29 family members have the same seed sequence and their binding sites in the 3′ UTRs of *ATP5G1* and *ATPIF1* are conserved across several species. (**B** and **C**) HEK-293 cells grown in hormonally depleted medium were transiently transfected with pEZ-MT10 dual luciferase reporter containing the 3′ UTR of ATP5G1 wildtype (WT), ATPIF1 WT, miR-29 seed element mutated (Mut) constructs and an RNA mimic negative control or miR-29b or miR-29a mimics +/− Anti-miR-29a for 24 h prior to dual luciferase assay. Data are the avg. of three replicate wells ± SEM. *p < 0.05 *versus* negative control. Statistical analysis used one-way ANOVA followed by Newman-Keuls Multiple Comparison Test.

To determine whether *ATP5G1* and *ATPIF1* are *bona fide* miR-29 targets, we tested the ability of miR-29 to repress the luciferase activity from reporter vectors containing wild type (WT) or mutant (mut) 3′ UTRs of these genes. The miR-29 binding region at the 3′ UTR region of *ATP5G1* and *ATPIF1* are conserved across species (Fig. 4A). We created mutations in the miR-29 recognition sequence in ATP5G1 (Fig. 4A). Cotransfection of miR-29b or miR-29a mimics with the luciferase reporter containing *ATP5G1* 3′ UTR WT sequence repressed luciferase activity (Fig. 4B). Mutation of the *ATP5G1* 3′ UTR (Mut) abrogated repression by the miR-29b and miR-29a mimics (Fig. 4B). These data demonstrate that *ATP5G1* is a *bona fide* direct target of miR-29b and miR-29a. Additionally, miR-29b and miR-29a mimics repressed luciferase activity from the *ATPIF1* 3′ UTR WT luciferase vector and repression was abrogated when cells were cotransfected with anti-miR-29a (Fig. 4C). These data suggest that *ATPIF1* is also a *bona fide* target of miR-29b and miR-29a.

**Figure 5:**
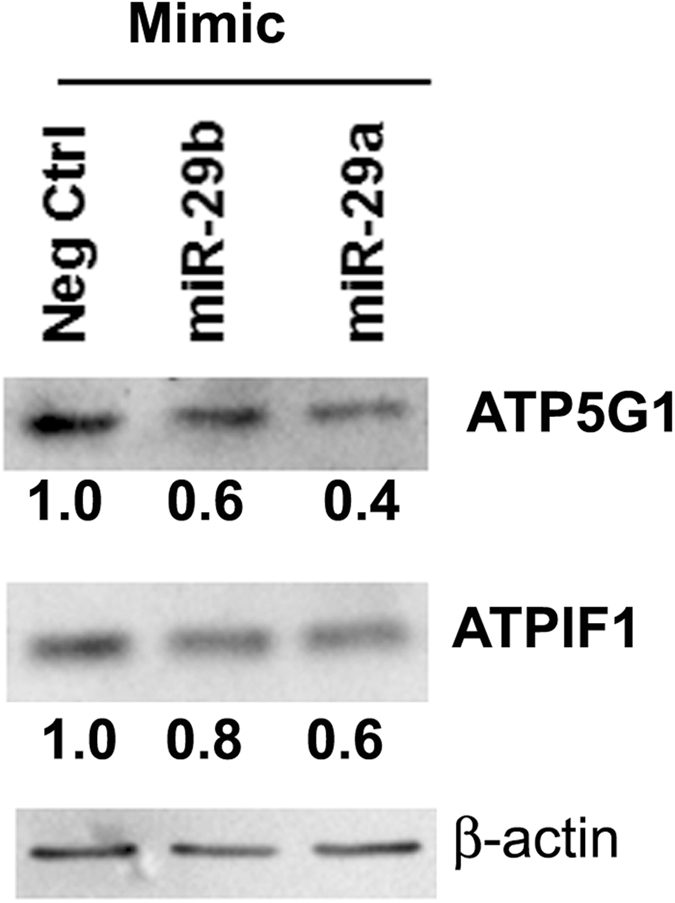
miR-29-b-1/a downregulate ATP5G1 and ATPIF1 protein expression in LCC9 breast cancer cells. LCC9 cells were grown in hormonally depleted medium and transfected with mimic negative control (Neg Ctrl), miR-29b mimic, or miR-29a mimic for 48 h. ATP5G1 and ATPIF1 and β-actin (normalizer) was examined in WCE (10 µg protein/lane). These images were cropped from full-length blots (Supplementary Figs 13–15). The same blot was stripped and reprobed for each protein. Values are ratios of ATP5G1/β-actin or ATPIF1/ β-actin with the Neg Ctrl set to one. This blot if from one experiment.

To further confirm *ATP5G1* and *ATPIF1* as miR-29 *bona fide* targets, we examined whether miR-29b-1 and miR-29a decreased protein expression of these genes. We transfected LCC9 cells with miR-29b and miR-29a mimics and evaluated ATP5G1 and ATPIF1 protein expression in whole cell extracts (Fig. 5). Compared to the negative control transfected cells, mimics of miR-29b-1 and miR-29a decreased protein expression of ATP5G1 and ATPIF1. These data further confirm *ATP5G1* and *ATPIF1* as validated targets of miR-29.

## Discussion

Dysregulation of the miR-29 family members plays a role in breast cancer progression, metastasis, and endocrine resistance^17, 32^. Using miRpower^33^, we observed that lower expression of miR-29b-1 and miR-29a is associated with decreased relapse-free survival (RFS) in overall breast cancer and in patients with ER + primary tumors (Supplementary Fig. 9). However, the precise mechanism(s) and targets of miR-29 in breast cancer are not fully elucidated. Here we identified the miR-29b-1 and miR-29a transcriptomes in MCF-7 TAM-S and LCC9 TAM-R breast cancer cells. Although miR-29b-1 and miR-29a have commonly regulated transcripts, they also have uniquely regulated transcripts. We observed that miR-29 regulates more transcripts in LCC9 than MCF-7 cells. While the biological reason for the higher number of targets of miR-29 identified in LCC9 compared to MCF-7 cells is unknown, we speculate that miR-29 may downregulate a suppressor(s) of transcription in LCC9 cells. One example is *DNMT3B*, a validated target of miR-29b^34^, miR-29a, and miR-29c^35^. DNMT3 family members are gene-specific, *de novo* DNA methyl transferases^36^. We observed that *DNMT3B* is a target of miR-29b-1/a in LCC9, but not MCF-7 cells (Supplementary Fig. 12). The identity of additional suppressors remains to be examined. Alternatively, the expression of components of the complexes involved in miRNA processing and activity, *e*.*g*., DICER1 and RISC complexes, may be higher in LCC9 than MCF-7 cells, cellular components that stabilize miR-29b-1/a may be more abundant in LCC9 cells, or the expression of lncRNAs acting as miR-29 sponges may be higher in MCF-7 cells. Further experiments are needed to address these speculation MetaCore enrichment analysis of unique miR-29 target transcripts in LCC9 cells identified GO pathways involved in oxidative phosphorylation, ATP metabolism, apoptosis and survival. In agreement with this literature-based association, ectopic miR-29a expression resulted in an inhibition of mitochondrial bioenergetics in LCC9 cells. Further, we demonstrate for the first time that *ATP5G1* and *ATPIF1* are validated, *bona fide* miR-29a targets.

*ATP5G1* encodes a subunit (C1) of the inner mitochondrial membrane-bound Fo domain of mitochondrial ATP synthase (complex V) that is involved in H+ transport necessary for the proton-motive force for ATP synthesis^37^. Inhibition of ATP synthase blocks the proliferation of breast cancer cells, but not normal MCF-10A breast epithelial cells^38^. Therapeutic targeting ATP synthase is of interest in blocking proliferation of breast stem-like cancer cells (CSCs) to inhibit disease recurrence and metastasis^39^. *ATPIF1* encodes ATPase Inhibitory Factor I that is in a 1:1 ratio with F1 domain of ATP synthase and functions to limit ATP depletion when the mitochondrial membrane potential falls below a threshold needed for ATP synthesis^40^. *ATPIF1* is required to avoid the consumption of cellular ATP when the ATP synthase enzyme acts in reverse to hydrolyze ATP^41^.

Dysregulation of mitochondrial bioenergetics is common in cancer cells^29^. Thus, identifying impaired mitochondrial pathways is of pathological and therapeutic relevance^42^. Mitochondria dysregulation results from alterations in mitochondrial function and ultrastructure^43^, overproduction of reactive oxygen species (ROS)^44^, and mitochondrial DNA (mtDNA) mutations and deletions^44, 45^. miRNA regulation of mitochondria has previously been reported (reviewed in refs 46 and 47). However, this is the first report that miR-29b-1/a regulate genes in ATP synthase (Complex V).

We examined the expression of *ATP5G1* and *ATPIF1* in relation to overall survival and disease free survival of ERα+ breast cancer patients using KM plotter^48^ (Supplementary Fig. 10). We focused on ERα+ patients because they would be likely to receive TAM and/or AI therapy. We observed that high *ATP1F1* was associated with increased OS in ERα+ breast cancer patients (Supplementary Fig. 10B) while higher *ATP5G1* correlated with reduced RFS in ERα+ breast cancer patients (Supplementary Fig. 10C). Another breast tumor database of gene expression from microarrays also showed that higher *ATP5G1* expression was associated with reduced disease free survival (DFS, which is the same as RFS) in ERα+ breast cancer patients in Ireland (http://glados.ucd.ie/BreastMark/mRNA_analysis.html)^49^. We note that all data in both databases are from primary tumors. Based on our LCC9 and LY2 cell experiments, we would expect that breast cancer patients with endocrine-resistant disease (metastasis) on TAM therapy will have increased miR-29b-1/a which would result in lower *ATP5G1* and *ATPIF1* transcript levels. The lower *ATPIF1* associated with lower OS (Supplementary Fig. 10B) agrees with this speculation; however, the apparent protective effect of lower *ATP5G1* (Supplementary Figs 10C and 11) does not. Because the gene expression data are from primary tumors, the impact of the expression of these genes in metastatic, endocrine-resistant tumors is unknown.

In agreement with the MetaCore pathway enrichment identification of OXPHOS as the top enriched pathway among miR-29b-1/a regulated genes in LCC9 cells, we observed that pre-miR-29a decreased basal OCR, ATP-linked OCR, and mt reserve. Concordantly, anti-miR-29a increased OCR and mt reserve, suggesting that repression of miR-29a may serve as an adaptive response to meet the increased energy demand of cancer cells and promote survival. Basal-like human breast tumors have lower levels of miR-29a, miR-29b, and miR-29c compared with Luminal A and B tumors^50^. Repression of miR-29a increased basal ECAR (glycolysis) in LCC9 cells (Fig. 2C), a finding in agreement with the Warburg effect of increased glycolysis seen in basal-like tumors^51, 52^. Overall changes in OCR and ECAR suggest overexpression of miR-29a has a greater impact on OXPHOS while repression of miR-29a increases both glycolytic activity and mitochondrial activity (OXPHOS) in LCC9 cells. Pre-miR-29a transfection repressed proton leak in LCC9 cells to levels below those seen in MCF-7 cells with hormone deprivation, suggesting miR-29a overexpression depletes mitochondrial OXPHOS, which could lead to activation of apoptosis or other cell death pathways.

LCC9 cells alone or when transfected with anti-miR-29a had a higher maximum mitochondrial capacity than the hormonally depleted MCF-7 cells, suggesting increased bioenergetics reserve. Indeed repression of miR-29a in LCC9 cells increased the glycolytic reserve. This may be attributable in part to the higher non-mitochondrial activity in LCC9 cells. Further metabolomics studies will be required to further assess changes in cellular metabolism with changes in miR-29b/a expression.

We identify several ETC proteins including ATP synthase genes *ATP5G1* and *ATPIF1* as targets of miR-29b-1/a. We experimentally validated that *ATP5G1* and *ATPIF1* are *bona fide* targets of miR-29b and miR-29a. Inhibition of ATP synthase in MCF7, T47D and MDA-MB-231 BC cells induced cell cycle arrest, decreased colony formation, and inhibited cell proliferation^53^. The targeting of ATP synthase complex subunits may account for miR-29’s repression of OCR and of BC cell proliferation^17^. Additional studies are warranted to confirm this suggestion.

## Conclusions

Our RNA-seq transcriptome analysis revealed that miR-29b-1 and miR-29a regulate common and unique gene targets in TAM-S MCF-7 and TAM-R LCC9 BC cells. We focused on common miR-29b-1 and miR-29a targets downregulated uniquely or in common in MCF-7 and LCC9 cells. We report that miR-29b-1 and miR-29a regulate more transcripts in LCC9 than MCF-7 cells. MetaCore pathway enrichment analysis identified OXPHOS as the top GO pathway regulated by miR-29b-1/a target transcripts in LCC9 cells and cell cycle: chromosome condensation in prometaphase as the top GO pathway in MCF-7 cells. Seahorse extracellular flux analyses of LCC9 and MCF-7 cells confirmed regulation of cellular bioenergetics by miR-29a including repression of OXPHOS by ectopic miR-29a expression. We experimentally validated *ATP5G1* and *ATPIF1* as new miR-29 *bona fide* targets suggesting that these targets play a role in mediating miR-29’s anti-proliferative effects in LCC9 and MCF-7 breast cancer cells.

## Materials and Methods

### Cell lines and reagents

The following cells and reagents were used: HEK-293 (ATCC^®^ CRL-1573^™^) and MCF-7 cells were purchased from ATCC. LCC9 cells are ERα+/progesterone receptor (PR) + cells resistant to antiestrogens (tamoxifen and fulvestrant) and were kindly provided by Dr. Robert Clarke, Georgetown University^54^. Transfection reagents included Anti-miR-29a (Anti-miR^TM^s, Ambion), pre-miR-29b-1–3p or pre-miR-29a-3p precursor (Pre-miR^TM^s, Ambion), miR-29a mimic or miR-29b-1 mimic (miR-Vana^TM^, Ambion), Lipofectamine RNAiMAX (Invitrogen), FuGENE® HD (Promega) Pre-miR™ negative control #1 (Ambion), Anti-miR™ negative control #1(Ambion), miR-Vana^TM^ negative control (Ambion), pEZX-MT06_ATP5G1 wild type (WT), pEZX-MT06_ATPIF1 (WT), pEZX-MT06_negative control (miTarget^TM^ miRNA 3′ UTR, GeneCopoeia).

### Transient transfection of miRNAs and anti-miRNAs

Cells were grown as previously described in ref. 17. Briefly, MCF-7 and LCC9 cells were grown in ‘hormone-depleted’ medium: phenol-red free IMEM (Gibco, ThermoFisher) supplemented with 5% dextran-coated charcoal-stripped fetal bovine serum (DCC-FBS, Atlanta Biologicals)^17^ and concomitantly transfected with either anti-miR-29a, pre-miR-29b-1-3p, pre-miR-29a-3p, pre-miR™ negative control #1 or anti-miR™ negative control #1 for a 48 h, as indicated.

### RNA-sequencing

RNA-sequencing was previously described in ref. 26. In brief, MCF-7 and LCC9 breast cancer cells were plated in 100 mm plates, ‘hormone-depleted’ and transfected in triplicate with either pre-miR-29b-1, pre-mir-29a and anti-miR-29a using Lipofectamine (Ambion) and OPTI-MEM® I (Gibco: Life technologies). 24 h post transfection, RNA was isolated using miRCURY RNA isolation kit (Exiqon) according to manufacturer’s instructions and RNA concentration and quality assessed with a NanoDrop spectrophotometer. Using TruSeq Stranded mRNA kit (Illumina, San Diego, CA, USA), mRNA libraries were made using 2 μg of RNA and validated with an Agilent 2100 Bioanalyzer (Santa Clara (CA). After further library quantification with the Illumina Library Quantification Kit, RT-PCR was performed using the ABI Prism qPCR Mix (Kapa Biosystems) on an ABI17900HT real-time PCR instrument. Single read sequencing (75–76 cycles) was then performed using the 500 High-output v2 (75cycle) sequencing kit on an Illumina NextSeq500 instrument. Obtained read sequences were mapped to the human reference genome version GRCh37.1 using the mapping algorithm tophat^55^ version 2.0.2. Using cufflinks version 2.2.1 and annotations found at ENSEMBL, Homo_sapiens GRCh37.73.gtf expression levels at loci were quantified. Raw sequencing data files obtained from our analysis are available at Gene Expression Omnibus (GEO) database: accession number GSE81620. Entrez gene identifiers for significantly expressed genes had a q-value cutoff of 0.05. Samples were then divided into fastq single end sequencing files representing comparisons of pre-miR-29b-1 vs anti-miR-29a and anti-miR-29a vs pre-miR-29a in MCF-7 and LCC9 cells.

### *In silico* network analysis

Data from RNA-seq was analyzed such that transcripts selected had a log2 fold-change greater than 0.34 (or −0.34 for repressed transcripts) and a statistical significant threshold q value less than 0.05. Pathway and network analysis of differentially expressed genes was determined using the web-based software MetaCore^TM^ version 6.27 (GeneGO, Thomson Reuters, New York, N.Y). MetaCore^TM^ is a manually curated data base of over 6 million experimental findings interactions including protein-protein, protein-DNA, protein-RNA, and protein-compounds; metabolic and signaling pathways; and other additional information^56^.

### Metabolic analysis with the Seahorse XF24 extracellular flux analyzer

Metabolic profiles were performed as previously described in refs 26 and 27. Briefly, LCC9 cells were plated at 25,000 cells/well in XF24 plates. Twenty-four h post plating, cells were transfected with either anti-miR-29a or pre-miR-29a for 48 h. One hour prior to running the Seahorse Mito Stress Test assay, the media was replaced with DMEM assay media containing 1 mM sodium pyruvate, 25 mM glucose, and 1.85 g/L NaCl (all from Sigma), 2 mM Glutamax (ThermoFisher), pH 7.4, and maintained at 37 °C in an non-CO_2_ incubator. Sensor cartridges were incubated in XF24 calibrant solution (Agilent Technologies) at least 6 h prior to running assay. Initial measurement of basal ECAR (mpH/min) and OCR (pmol O_2_/min) were taken prior to determining mitochondrial function using sequential injections of oligomycin A (1.5 μM), FCCP (0.5 μM) and combination of antimycin A (10 μM) and rotenone (2 μM)^26^. The protein concentration in each well was determined using the BioRad DC™ Protein Assay (BioRad, Hercules, CA, USA). OCR and ECAR values were normalized to protein concentration/well. Each experimental condition was run in quadruplicate within one experiment and separate experiments were run 3–4 times for statistical evaluation.

### Quantitative real-time PCR (qRT-PCR)

RNA isolation, RT-PCR and qPCR were performed as previously described in ref. 17. TaqMan (Thermo Fisher Scientific) master mix was used for miR-29b-1/a primers (Thermo Fisher Scientific) and SYBR green (QIAGEN) master mix was used for *LDHA* ^57^, *ENO1* ^58^, *CYCS* ^59^, *ATP5G1* ^60^, *ATP5C1* ^61^, *ATPIF1* ^62^, *ATP5G3* ^60^, *NDUFS6* ^63^, *NDUFC2* ^64^. Normalizers included *RNU6B* or *U48* (Thermo Fisher Scientific) for miRNA and *GAPDH* (SYBR green)^65^ and *18S* (TaqMan; Thermo Fisher) for mRNA.

### Site-directed mutagenesis, transient transfection of HEK-293 cells, and Dual luciferase assay

Mutation of the predicted 3′ UTR binding site of miR-29b-1/a on pEZX-MT06 miRNA 3′ UTR target dual luciferase expression vectors was performed using the GeneArt^®^ Site-Directed Mutagenesis PLUS Kit (ThermoFisher Scientific) according to manufacturer’s instructions. Where indicated, HEK-293 cells were plated in 24 well plates and co-transfected with 200 ng of wild type (wt) or mutant (mut) vectors plus a 20 nM final concentration of miR-29b-1/a mimic, anti-miR-29a or negative control mimic using FuGENE HD (Promega) according to manufacturer’s protocol. Twenty-four h post transfection, a dual luciferase assay (Promega) was performed. Luciferase expression was determined relative to negative control mimic and statistical evaluation performed using GraphPad Prism Software (Graph Pad Software, San Diego, CA).

### Western blot

LCC9 cells were transfected with miRNA MIMIC 2.0 (NEG #1, Life Technologies), miR-29b mimic, or miR-29a mimic (Life Technologies) for 48 h. Whole cell extracts (WCE) were prepared RIPA buffer (Sigma) in the presence of complete protease and phosphatase inhibitors. 10 μg of protein, determined by Bio-Rad DC protein quantification assay, were electrophoresed on a 10% SDS-PAGE gel. Following separation, the proteins were transferred to a nitrocellulose membrane and immunoblotted for ATP5G1 (GeneTex, Cat # GTX104671), ATPIF1 (Cell Signaling, Cat #8528) and β-actin (Sigma; loading control). Bands were visualized on a Bio-Rad ChemiDoc™MP imaging system with Image Lab version 5.2.1 software and quantified by UN-SCAN-IT Graph Digitizer version 7.1 software.

### Statistics

Data are represented as mean ± standard error of the mean (SEM) of at least three independent experiments. Statistical analyses were performed using GraphPad Prism 5 (Graph Pad Software, Inc.). One-way analysis of variance (ANOVA) was followed by Tukey’s or Newman-Keuls multiple comparison *post hoc* tests where indicated^17^.

### Data availability statement

Raw sequencing data files obtained from our analysis are available at GEO: accession number GSE81620. All data analysed during this study are included in this published article (and its Supplementary Information files).

## Electronic supplementary material

Supplementary Tables and Figures
