# Supplementary material for: The miR-29 transcriptome in endocrine-sensitive and resistant breast cancer cells
Source: Sci Rep. 2017 Jul 12;7:5205. doi: 10.1038/s41598-017-05727-w (PMC5507892; doi:10.1038/s41598-017-05727-w)

Supplementary Tables (3) and Figures (15)

The miR-29 transcriptome in endocrine-sensitive and resistant breast cancer cells

Penn Muluhngwi, Negin Alizadeh-Rad, Stephany L. Vittitow, Ted S. Kalbfleisch, and Carolyn M. Klinge

List of files:

Supplementary Table 1: miR-29 targets in MCF-7 cells

Supplementary Table 2: miR-29 targets in LCC9 cells

Supplementary Table 3: miR-29 targets common in MCF-7 and LCC9 cells

Supplementary Figure 1: Successful knockdown and upregulation of miR-29b-1 /a in MCF-7 and LCC9 cells.

Supplementary Figure 2: Enrichment analysis of RNA-seq data in MCF-7 and LCC9 cells.

Supplementary Figure 3: Enrichment onthologies of genes regulated by miR-29b-1/a in MCF-7 and LCC9 cells.

Supplementary Figure 4: Enrichment onthologies of genes uniquely downregulated in LCC9 cells.

Supplementary Figure 5: miR-29a regulates mitochondrial function of MCF-7 and LCC9 BC cells.

Supplementary Figure 6: Glycolytic gene expression in MCF-7 and LCC9 BC cells

Supplementary Figure 7: RNA-seq expression profiles of *ATP5G1*, *ATP5C1*, *ATPIF1*, *ATP5G3*, *NDUFS6* and *NDUFC2* in MCF-7 and LCC9 breast cancer cells.

Supplementary Figure 8: Basal expression levels of putative miR-29b-1/a targets in breast cancer cells.

Supplementary Figure 9: Lower expression of miR-29b-1 and miR-29a is statistically associated with decreased relapse-free survival (RFS) in all breast cancer and in patients whose primary tumors are ERα+.

Supplementary Figure 10: Association of *ATP5G1* and *ATPIF1* transcript levels with Overall Survival (OS) and Relapse Free Survival (RFS) from ERα+ breast tumor data

Supplementary Figure 11: Association of *ATP5G1 expression and* Disease Free Survival (DFS) in luminal B breast tumors.

Supplementary Figure 12: *DNMT3B* expression in MCF-7 and LCC9 cells.

Supplementary Figure 13: Uncropped TIFF greyscale image of western blot for ATP5G1, lanes 2-4 are shown in Fig. 5.

Supplementary Figure 14: Uncropped TIFF greyscale image of western blot for ATPIF1, lanes 2-4 are shown in Fig. 5.

Supplementary Figure 15: Uncropped TIFF greyscale image of western blot for beta-actin, lanes 2-4 are shown in Fig. 5. .

Supplementary Table 1: miR-29 targets in MCF-7 cells

Supplementary Table 1A: miR-29a unique targets in MCF-7 cells

					MCF-7_Pre-miR-29b1 vs Anti-miR-29a
	**Gene Symbol**	**Entrez Gene IDs**	**OMIM IDs**	**Gene Name**	**log2(fold_change)**
1.	HIBADH	11112	608475	3-hydroxyisobutyrate dehydrogenase	0.463839
2.	EIF4EBP2	1979	602224	eukaryotic translation initiation factor 4E binding protein 2	0.503226
3.	ABCB7	22	300135;301310	ATP binding cassette subfamily B member 7	0.503745
4.	ADAMTS19	171019	607513	ADAM metallopeptidase with thrombospondin type 1 motif 19	0.895057
5.	ADAM9	8754	602713;612775	ADAM metallopeptidase domain 9	0.538095
6.	ADH5	128	103710	"alcohol dehydrogenase 5 (class III), chi polypeptide"	0.569996
7.	AHNAK	79026	103390	AHNAK nucleoprotein	0.71501
8.	AIF1L	83543	No ID	allograft inflammatory factor 1 like	0.441216
9.	AK4	205	103030	adenylate kinase 4	0.779273
10.	ALDH7A1	501	107323;266100	aldehyde dehydrogenase 7 family member A1	0.609418
11.	FAM117B	150864	No ID	family with sequence similarity 117 member B	0.495994
12.	AMIGO2	347902	615690	adhesion molecule with Ig like domain 2	0.361687
13.	GNA14	57037	No ID	ankyrin repeat and MYND domain containing 2	0.838909
14.	ANO6	196527	262890;608663	anoctamin 6	0.504893
15.	SLC25A5	292	300150	solute carrier family 25 member 5	0.531863
16.	APLP2	334	104776	amyloid beta precursor like protein 2	0.634654
17.	APP	351	104300;104760;605714	amyloid beta precursor protein	0.591877
18.	NCAM2	401	602078;602753	paired like homeobox 2a	0.793964
19.	ARL5B	221079	608909	ADP ribosylation factor like GTPase 5B	0.526453
20.	ARNT	405	126110	aryl hydrocarbon receptor nuclear translocator	0.516884

21.	ACTR2	10097	604221	ARP2 actin related protein 2 homolog	0.388238
22.	ATP11B	23200	605869	ATPase phospholipid transporting 11B (putative)	0.79681
23.	ATP1B1	481	145500;182330	ATPase Na+/K+ transporting subunit beta 1	0.460634
24.	ATP5A1	498	164360;615228;616045	"ATP synthase, H+ transporting, mitochondrial F1 complex, alpha subunit 1, cardiac muscle"	0.4749
25.	ATP5B	506	102910	"ATP synthase, H+ transporting, mitochondrial F1 complex, beta polypeptide"	0.526923
26.	ATP5EP2	432369	No ID	"ATP synthase, H+ transporting, mitochondrial F1 complex, epsilon subunit pseudogene 2"	0.690007
27.	ATP6V1A	523	607027	ATPase H+ transporting V1 subunit A	0.786597
28.	ADK	132	102750;614300	adenosine kinase	0.421368
29.	ADIPOR2	79602	607946	adiponectin receptor 2	0.489597
30.	SGCE	8910	159900;604149	sarcoglycan epsilon	1.66249
31.	PTPLAD1	51495	615940	3-hydroxyacyl-CoA dehydratase 3	0.559162
32.	B3GNT5	84002	615333	"UDP-GlcNAc:betaGal beta-1,3-N-acetylglucosaminyl transferase 5"	0.885252
33.	SMARCC1	6599	601732	"SWI/SNF related, matrix associated, actin dependent regulator of chromatin subfamily c member 1"	0.67286
34.	BCAS1	8537	602968	breast carcinoma amplified sequence 1	0.598178
35.	DBT	1629	248600;248610	dihydrolipoamide branched chain transacylase E2	0.560221
36.	BCKDHB	594	248600;248611	branched chain keto acid dehydrogenase E1 subunit beta	0.648202
37.	GLB1	2720	230500;230600;230650;253010;611458	galactosidase beta 1	0.728066
38.	BRI3BP	140707	615627	BRI3 binding protein	0.55248
39.	BTF3	689	602542	basic transcription factor 3	0.481856
40.	CTNNB1	1499	114500;114550;116806;132600;155255;156240;167000;181030;615075	catenin beta 1	0.506777

41.	APMAP	57136	615884	adipocyte plasma membrane associated protein	0.401089
42.	LINC00205	102723489	No ID	long intergenic non-protein coding RNA 205	0.5533
43.	CADPS2	93664	609978	calcium dependent secretion activator 2	0.738824
44.	CALCOCO1	57658	No ID	calcium binding and coiled-coil domain 1	0.58291
45.	SLC7A2	6542	601872	solute carrier family 7 member 2	0.448744
46.	CBLN4	140689	615029	cerebellin 4 precursor	0.974278
47.	CD302	9936	612246	CD302 molecule	0.766404
48.	CD44	960	107269;172290;609027	CD44 molecule (Indian blood group)	0.780123
49.	CDC42	998	116952;616737	cell division cycle 42	0.484322
50.	CHP1	11261	606988	calcineurin like EF-hand protein 1	0.466563
51.	CHSY1	22856	605282;608183	chondroitin sulfate synthase 1	0.45663
52.	GNS	2799	252940;607664	glucosamine (N-acetyl)-6-sulfatase	0.758816
53.	CLEC3A	10143	613588	C-type lectin domain family 3 member A	1.36846
54.	CMTM4	146223	607887	CKLF like MARVEL transmembrane domain containing 4	0.845446
55.	CMTM6	54918	607889	CKLF like MARVEL transmembrane domain containing 6	0.527145
56.	CREG1	8804	No ID	cellular repressor of E1A stimulated genes 1	0.546034
57.	EMB	7514	602559	exportin 1	0.636447
58.	SCP2	10106	608711	CTD small phosphatase 2	0.553321
59.	SPANXA2-OT1	619455	No ID	SPANXA2 overlapping transcript 1	1.15675
60.	CYP1B1	1545	137750;137760;231300;600975;601771;604229	cytochrome P450 family 1 subfamily B member 1	0.589318
61.	ATP2A2	488	101900;108740;124200	ATPase sarcoplasmic/endoplasmic reticulum Ca2+ transporting 2	0.486506
62.	CLSTN2	64084	611323	calsyntenin 2	0.820318
63.	CETN2	1069	300006	centrin 2	0.545244
64.	MPP7	8208	601245	chromatin assembly factor 1 subunit B	0.669472
65.	CLTC	1213	118955	clathrin heavy chain	0.518539
66.	COL12A1	1303	120320;616470;616471	collagen type XII alpha 1 chain	0.997639

67.	COL14A1	7373	120324	collagen type XIV alpha 1 chain	1.07939
68.	CUL4B	8450	300304;300354	cullin 4B	0.423513
69.	CCNG2	901	603203	cyclin G2	0.608184
70.	CCNI	10983	No ID	cyclin I	0.704477
71.	CD55	1604	125240;613793	CD55 molecule (Cromer blood group)	0.445821
72.	DAZAP2	9802	607431	DAZ associated protein 2	0.471512
73.	OSTC	58505	No ID	oligosaccharyltransferase complex non-catalytic subunit	0.72726
74.	DCLK1	9201	604742	doublecortin like kinase 1	0.709475
75.	PTPRJ	5795	600925	"protein tyrosine phosphatase, receptor type J"	0.58445
76.	GLUD1	2746	138130;606762	glutamate dehydrogenase 1	0.534844
77.	DIO2	1734	601413	"deiodinase, iodothyronine, type II"	1.43126
78.	ENSG00000264364	140735	608942	dynein light chain LC8-type 2	0.449333
79.	DNAJC25	548645	No ID	DnaJ heat shock protein family (Hsp40) member C25	0.668936
80.	METTL9	51108	609388	methyltransferase like 9	0.574265
81.	DSP	1834	125420;125485;125490;125500;605594	dentin sialophosphoprotein	0.456755
82.	DSTN	11034	609114	"destrin, actin depolymerizing factor"	0.521062
83.	EEF1A1P5	158078	No ID	eukaryotic translation elongation factor 1 alpha 1 pseudogene 5	0.643883
84.	EEF1A1P9	441032	No ID	eukaryotic translation elongation factor 1 alpha 1 pseudogene 9	0.712999
85.	EIF3L	51386	No ID	eukaryotic translation initiation factor 3 subunit L	0.67883
86.	ELOVL5	60481	611805;615957	ELOVL fatty acid elongase 5	0.424171
87.	ENPP1	5167	125853;173335;208000;601665;602475;613312;615522	ectonucleotide pyrophosphatase/phosphodiesterase 1	0.6384
88.	ETNK2	55224	609859	ethanolamine kinase 2	0.768113
89.	RUNX1T1	862	133435	RUNX1 translocation partner 1	1.0154
90.	HSP90B1	7184	191175	heat shock protein 90 beta family member 1	0.483563
91.	EPCAM	4072	185535;613217;613244	epithelial cell adhesion molecule	0.437596
92.	EFNB3	1949	602297	ephrin B3	0.479075

93.	EPS15	2060	600051	epidermal growth factor receptor pathway substrate 15	0.470549
94.	ERGIC3	51614	616971	ERGIC and golgi 3	0.570885
95.	EXT2	2132	133701;601224;608210;616682	exostosin glycosyltransferase 2	0.687945
96.	FBXO21	23014	609095	F-box protein 21	0.455003
97.	TMEM150C	441027	No ID	transmembrane protein 150C	0.490383
98.	FRAT2	23401	605006	frequently rearranged in advanced T-cell lymphomas 2	0.467266
99.	FUCA1	2517	230000;612280	"fucosidase, alpha-L- 1, tissue"	0.487582
100.	FUT8	2530	602589	fucosyltransferase 8	0.594184
101.	FZD6	8323	603409;614157	frizzled class receptor 6	0.421184
102.	PLS1	5357	602734	plastin 1	0.599518
103.	FRAS1	80144	219000;607830	Fraser extracellular matrix complex subunit 1	0.69899
104.	FUT9	10690	606865	fucosyltransferase 9	0.59
105.	GNG12	55970	615405	G protein subunit gamma 12	0.612162
106.	GDE1	51573	605943	glycerophosphodiester phosphodiesterase 1	0.537638
107.	GFRA3	2676	605710	GDNF family receptor alpha 3	1.04305
108.	GPD1L	23171	272120;611777;611778	glycerol-3-phosphate dehydrogenase 1-like	0.41666
109.	GPNMB	10457	604368	glycoprotein nmb	0.744162
110.	TMEM181	57583	613209	transmembrane protein 181	0.553905
111.	GSTA4	2941	605450	glutathione S-transferase alpha 4	0.934667
112.	GLO1	2739	138750	glyoxalase I	0.402551
113.	AGR2	10551	606358	"anterior gradient 2, protein disulphide isomerase family member"	0.467375
114.	HINT3	135114	609998	histidine triad nucleotide binding protein 3	0.476442
115.	HIPK1	204851	608003	homeodomain interacting protein kinase 1	0.559793
116.	BRK1	55845	611183	"BRICK1, SCAR/WAVE actin nucleating complex subunit"	0.475471
117.	HEATR5B	54497	No ID	HEAT repeat containing 5B	0.587967
118.	H3F3A	3020	601058;601128	H3 histone family member 3A	0.414925
119.	IGBP1	3476	300139;300472	immunoglobulin (CD79A) binding protein 1	0.415533

120.	IGSF1	3547	300137;300888	immunoglobulin superfamily member 1	0.733403
121.	IL1R1	3554	147810	interleukin 1 receptor type 1	0.78683
122.	IL13RA1	3597	300119	interleukin 13 receptor subunit alpha 1	0.618167
123.	IL1RAPL2	26280	300277	interleukin 1 receptor accessory protein like 2	1.43643
124.	IL23R	149233	605606;607562;612261	interleukin 23 receptor	0.643553
125.	INSIG1	3638	602055	insulin induced gene 1	0.423326
126.	ITGA6	3655	147556;226730	integrin subunit alpha 6	0.884631
127.	ARL6IP5	10550	605709	ADP ribosylation factor like GTPase 6 interacting protein 5	0.590619
128.	PRDX1	56659	607367	potassium two pore domain channel subfamily K member 13	0.489384
129.	KCTD20	222658	615932	potassium channel tetramerization domain containing 20	0.604093
130.	KCTD3	51133	613272	potassium channel tetramerization domain containing 3	0.691036
131.	FAM168A	23201	616316	family with sequence similarity 168 member A	0.502726
132.	KIAA1244	57221	No ID	ARFGEF family member 3	0.440378
133.	KIAA1324	57535	611298	KIAA1324	0.548838
134.	TXNDC16	57544	616179	thioredoxin domain containing 16	0.69446
135.	EPG5	57724	242840;615068	ectopic P-granules autophagy protein 5 homolog	0.619241
136.	KIAA2018	205717	No ID	upstream transcription factor family member 3	0.447222
137.	KIF1B	23095	118210;171300;256700;605995	kinesin family member 1B	0.540446
138.	KIF3B	9371	603754	kinesin family member 3B	0.526819
139.	FERMT2	10979	607746	fermitin family member 2	0.588173
140.	LAMP2	3920	300257;309060	lysosomal associated membrane protein 2	0.463606
141.	RPSA	3921	150370;271400	ribosomal protein SA	0.444295
142.	LANCL1	10314	604155	LanC like 1	0.573726
143.	LAPTM4B	55353	613296	lysosomal protein transmembrane 4 beta	0.683263
144.	ARHGEF12	23365	604763	Rho guanine nucleotide exchange factor 12	0.500601
145.	LDHA	3939	150000;612933	lactate dehydrogenase A	0.484053

146.	LDOC1	23641	300402	leucine zipper down-regulated in cancer 1	0.464045
147.	LRRFIP1	9208	603256	LRR binding FLII interacting protein 1	0.420842
148.	LSM11	134353	No ID	"LSM11, U7 small nuclear RNA associated"	0.491352
149.	LRRC58	116064	No ID	leucine rich repeat containing 58	0.483267
150.	MAN1A1	4121	604344	mannosidase alpha class 1A member 1	0.666365
151.	MAML2	84441	607537	mastermind like transcriptional coactivator 2	0.631339
152.	MAOB	4129	309860	monoamine oxidase B	0.878248
153.	MAP2	4133	157130	microtubule associated protein 2	0.770299
154.	MBOAT2	129642	611949	membrane bound O-acyltransferase domain containing 2	0.764623
155.	MCCC2	64087	210210;609014	methylcrotonoyl-CoA carboxylase 2	0.586974
156.	SLC16A7	9194	603654	solute carrier family 16 member 7	0.753861
157.	ME1	4199	154250	malic enzyme 1	0.482464
158.	MAP3K1	4214	600982;613762	mitogen-activated protein kinase kinase kinase 1	0.465687
159.	MEST	4232	601029	mesoderm specific transcript	0.752552
160.	MGST3	4259	604564	microsomal glutathione S-transferase 3	0.545689
161.	DUSP4	1846	602747	dual specificity phosphatase 4	0.405569
162.	MMP16	4325	602262	matrix metallopeptidase 16	1.14912
163.	MORF4L2	9643	300409	mortality factor 4 like 2	0.413342
164.	MTMR2	8898	601382;603557	myotubularin related protein 2	0.65462
165.	MUCL1	118430	610857	mucin like 1	0.762445
166.	MUT	4594	251000;609058	methylmalonyl-CoA mutase	0.487788
167.	PAM	23077	610392	"MYC binding protein 2, E3 ubiquitin protein ligase"	0.934899
168.	MYO1B	4430	606537	myosin IB	0.632889
169.	MYOF	26509	604603	myoferlin	0.462151
170.	MYO6	4646	600970;606346;607821	myosin VI	0.654896
171.	N4BP2	55728	No ID	NEDD4 binding protein 2	0.685434
172.	NCKAP5	344148	608789	NCK associated protein 5	1.05894
173.	SLC8A1	6546	182305	solute carrier family 8 member A1	0.551218
174.	NDRG3	57446	605273	NDRG family member 3	0.666619

175.	NEBL	10529	605491	nebulette	0.609686
176.	NEK6	10783	604884	NIMA related kinase 6	0.502781
177.	BNIP3L	665	605368	BCL2 interacting protein 3 like	0.588141
178.	NPC2	10577	601015;607625	NPC intracellular cholesterol transporter 2	0.423578
179.	NQO1	1728	125860	NAD(P)H quinone dehydrogenase 1	0.46134
180.	MAGED1	9500	300224	MAGE family member D1	0.787782
181.	NRCAM	4897	601581	neuronal cell adhesion molecule	0.717003
182.	NRK	203447	300791	Nik related kinase	1.47776
183.	REST	5978	600571;616806	RE1 silencing transcription factor	0.481066
184.	IVNS1ABP	10625	609209	influenza virus NS1A binding protein	0.366829
185.	PRDX6	9588	602316	peroxiredoxin 6	0.390866
186.	NRG3	10718	605533	neuregulin 3	1.70994
187.	NBEA	26960	604889	neurobeachin	0.768566
188.	NUS1	116150	610463;617082	NUS1 dehydrodolichyl diphosphate synthase subunit	0.412875
189.	P4HA1	5033	176710	prolyl 4-hydroxylase subunit alpha 1	0.456554
190.	PABPC1	26986	604679	poly(A) binding protein cytoplasmic 1	0.624322
191.	PABPC4L	132430	No ID	poly(A) binding protein cytoplasmic 4 like	0.881233
192.	PAPSS1	9061	603262	3'-phosphoadenosine 5'-phosphosulfate synthase 1	0.637146
193.	PCDHB16;ME1	57717	604967;606345	protocadherin beta 16	0.666527
194.	PCDHB3	56132	604967;606329	protocadherin beta 3	0.596374
195.	PCDHB16;PCDHB8	56128	604967;606334	protocadherin beta 8	0.82234
196.	PCYOX1	51449	610995	prenylcysteine oxidase 1	0.669556
197.	PDE4B	5142	600127	phosphodiesterase 4B	1.3981
198.	PGK1	5230	300653;311800	phosphoglycerate kinase 1	0.542224
199.	PGM1	55276	172000	phosphoglucomutase 2	0.744899
200.	PHLDA1	22822	605335	pleckstrin homology like domain family A member 1	0.438257
201.	PHTF2	57157	616785	putative homeodomain transcription factor 2	0.763051
202.	PIR	11025	604820	leukocyte immunoglobulin like receptor B3	0.681574
203.	PJA2	9867	No ID	praja ring finger ubiquitin ligase 2	0.408532

204.	PRKACA	5566	601639;615830	protein kinase cAMP-activated catalytic subunit alpha	0.544697
205.	PLCXD3	345557	617016	phosphatidylinositol specific phospholipase C X domain containing 3	0.475518
206.	PLOD2	5352	601865;609220	"procollagen-lysine,2-oxoglutarate 5-dioxygenase 2"	0.631555
207.	PLXDC2	84898	606827	plexin domain containing 2	0.481995
208.	PPP2R2B	5521	604325;604326	protein phosphatase 2 regulatory subunit Bbeta	1.6416
209.	PPM1H	57460	616016	"protein phosphatase, Mg2+/Mn2+ dependent 1H"	0.51243
210.	PRICKLE2	166336	607459;608501;613832	prickle planar cell polarity protein 2	0.384805
211.	PRR11	55771	615920	proline rich 11	0.602035
212.	PSD3	23362	614440	pleckstrin and Sec7 domain containing 3	0.758157
213.	PTPLA	9200	255310;610467	3-hydroxyacyl-CoA dehydratase 1	0.703489
214.	PTDSS1	9791	151050;612792	phosphatidylserine synthase 1	0.457234
215.	RABL5	64792	No ID	intraflagellar transport 22	0.534114
216.	GNB2L1	10399	176981	receptor for activated C kinase 1	0.346332
217.	SMS	10743	182290;607642	retinoic acid induced 1	0.406627
218.	REG4	83998	609846	regenerating family member 4	0.92634
219.	RNF130	55819	No ID	ring finger protein 130	0.61701
220.	ROBO1	6091	602430	roundabout guidance receptor 1	1.05768
221.	ROCK1	6093	601702	Rho associated coiled-coil containing protein kinase 1	0.448331
222.	RPL11	6135	604175;612562	ribosomal protein L11	0.456786
223.	RPL17	6139	603661	ribosomal protein L17	0.388701
224.	RPL23A	6147	602326	ribosomal protein L23a	0.372125
225.	RPL24	6152	604180	ribosomal protein L24	0.342158
226.	RPL34	6164	616862	ribosomal protein L34	0.423911
227.	RPL35A	6165	180468;612528	ribosomal protein L35a	0.364678
228.	RPL39	6170	300899	ribosomal protein L39	0.429273
229.	RPL4	6124	180479	ribosomal protein L4	0.428614

230.	RPL41	6171	613315	ribosomal protein L41	0.532606
231.	RPL5	6125	603634;612561	ribosomal protein L5	0.538675
232.	RPL6	6128	603703	ribosomal protein L6	0.626526
233.	RPL7A;RPL7	6129	604166	ribosomal protein L7	0.47839
234.	RPL7A	6130	185640	ribosomal protein L7a	0.453726
235.	RPL9	6133	603686	ribosomal protein L9	0.479611
236.	RPS15A	6210	603674	ribosomal protein S15a	0.516768
237.	RPS17	6218	180472;612527	ribosomal protein S17	0.865613
238.	RPS4X	6191	312760	"ribosomal protein S4, X-linked"	0.495312
239.	RPS6	6194	180460	ribosomal protein S6	0.460106
240.	RPS7	6201	603658;612563	ribosomal protein S7	0.360347
241.	RAB13	5872	602672	"RAB13, member RAS oncogene family"	0.389677
242.	SASH1	23328	607955	SAM and SH3 domain containing 1	0.535221
243.	SETD7	80854	606594	SET domain containing lysine methyltransferase 7	0.39308
244.	SH3BGRL	6451	300190	SH3 domain binding glutamate rich protein like	0.771522
245.	SH3BGRL2	83699	615678	SH3 domain binding glutamate rich protein like 2	0.741634
246.	SIDT2	51092	No ID	SID1 transmembrane family member 2	0.837797
247.	SLC19A2	10560	249270;603941	solute carrier family 19 member 2	0.463341
248.	SLC25A15	10166	238970;603861	solute carrier family 25 member 15	0.477293
249.	SLC25A24	29957	608744	solute carrier family 25 member 24	0.375516
250.	SLC25A3	5250	600370;610773	solute carrier family 25 member 3	0.544249
251.	SLC25A43	203427	300641	solute carrier family 25 member 43	0.609365
252.	SLC38A1	81539	608490	solute carrier family 38 member 1	0.655669
253.	SLC39A6	25800	608731	solute carrier family 39 member 6	0.512706
254.	SLC44A1	23446	606105	solute carrier family 44 member 1	0.57194
255.	SLC4A10	57282	605556	solute carrier family 4 member 10	0.74709

256.	SLC4A7	9497	603353	solute carrier family 4 member 7	0.695701
257.	SLIT2	9353	603746	slit guidance ligand 2	1.11886
258.	SNX4	8723	605931	sorting nexin 4	0.522873
259.	SOX2-OT	347689	616338	SOX2 overlapping transcript	1.37425
260.	SEC11A	23478	No ID	"SEC11 homolog A, signal peptidase complex subunit"	0.460174
261.	SRD5A1	6715	184753	steroid 5 alpha-reductase 1	0.655391
262.	SSR2	6746	600867	signal sequence receptor subunit 2	0.479507
263.	NSD1	64324	117550;130650;606681	nuclear receptor binding SET domain protein 1	0.571588
264.	SUCLG2	8801	603922	succinate-CoA ligase GDP-forming beta subunit	0.520378
265.	SULF1	23213	610012	sulfatase 1	0.824325
266.	SUMF1	285362	272200;607939	sulfatase modifying factor 1	0.552387
267.	SEC24D	9871	607186;616294	"SEC24 homolog D, COPII coat complex component"	0.544885
268.	KIAA1598	57698	611171	shootin 1	0.644697
269.	SPAST	6683	182601;604277	spastin	0.461301
270.	STC1	6781	601185	stanniocalcin 1	0.480749
271.	TANC1	85461	611397	"tetratricopeptide repeat, ankyrin repeat and coiled-coil containing 1"	0.46755
272.	TC2N	123036	No ID	"tandem C2 domains, nuclear"	0.538673
273.	TET2	54790	263300;612839;614286	tet methylcytosine dioxygenase 2	0.526523
274.	TFPI	7035	152310	tissue factor pathway inhibitor	0.696434
275.	TGFB2	7042	190220;614816	transforming growth factor beta 2	0.983306
276.	TIMP2	7077	188825	TIMP metallopeptidase inhibitor 2	0.451907
277.	TLE4	7091	605132	transducin like enhancer of split 4	0.807059
278.	TM9SF2	9375	604678	transmembrane 9 superfamily member 2	0.499806
279.	TMEM2	23670	605835	transmembrane protein 2	1.03637
280.	TMEM59	9528	617084	transmembrane protein 59	0.557126
281.	TMEM65	157378	616609	transmembrane protein 65	0.57703
282.	TMEM66	51669	614768	store-operated calcium entry associated regulatory factor	0.480183
283.	LINC00052	145978	No ID	long intergenic non-protein coding RNA 52	0.38313
284.	TPT1	7178	600763	"tumor protein, translationally-controlled 1"	0.449878

285.	TRIB2	28951	609462	tribbles pseudokinase 2	0.548341
286.	TRPS1	7227	150230;190350;190351;604386	transcriptional repressor GATA binding 1	0.702954
287.	TSPAN6	7105	300191	tetraspanin 6	0.473511
288.	TTC3	7267	602259	tetratricopeptide repeat domain 3	0.809686
289.	TUG1	55000	614971	taurine up-regulated 1 (non-protein coding)	0.603472
290.	TUSC3	7991	601385;611093	tumor suppressor candidate 3	0.545792
291.	TSPAN3	10099	613134	tetraspanin 3	0.410548
292.	TMTC1	83857	615855	transmembrane and tetratricopeptide repeat containing 1	0.673343
293.	MARVELD2	153562	610153;610572	MARVEL domain containing 2	0.511093
294.	TSPAN32	10077	603853	tetraspanin 32	1.96707
295.	TUBA1A	7846	602529;611603	tubulin alpha 1a	0.784308
296.	TUBD1	51174	607344	tubulin delta 1	0.463035
297.	UBL3	5412	604711	ubiquitin like 3	0.437758
298.	UCP2	7351	601693;607447	uncoupling protein 2	0.494843
299.	UGDH	7358	603370	UDP-glucose 6-dehydrogenase	0.5911
300.	UGT1A6	54657	143500;191740;218800;606429;606785	UDP glucuronosyltransferase family 1 member A4	0.684012
301.	CSDE1	7812	191510	cold shock domain containing E1	0.572963
302.	SLC27A6	28965	604196	solute carrier family 27 member 6	0.686412
303.	DCAF12	25853	No ID	DDB1 and CUL4 associated factor 12	0.64399
304.	WSB2	55884	No ID	WD repeat and SOCS box containing 2	0.591227
305.	XYLT1	64131	264800;608124;615777	xylosyltransferase 1	0.664025
306.	YES1	7525	164880	"YES proto-oncogene 1, Src family tyrosine kinase"	0.484034
307.	YPEL2	388403	609723	yippee like 2	0.691608
308.	MLTK	51776	609479;616890	sterile alpha motif and leucine zipper containing kinase AZK	0.481224
309.	ZFP91	80829	No ID	ZFP91 zinc finger protein	0.48339
310.	ZNF652	22834	613907	zinc finger protein 652	0.397467
311.	ITGB1	3688	135630	integrin subunit beta 1	0.816957
312.	EEF1G	1937	130593	eukaryotic translation elongation factor 1 gamma	0.53172
313.	EIF3F	8665	603914	eukaryotic translation initiation factor 3 subunit F	0.563518

314.	EIF3E	3646	602210	eukaryotic translation initiation factor 3 subunit E	0.566074
315.	EIF4B	1975	603928	eukaryotic translation initiation factor 4B	0.595304

Supplementary Table 1B: Common miR-29b-1 and miR-29a targets in MCF-7 cells

					MCF-7_Pre-miR-29b1 vs Anti-miR-29a	MCF-7_Pre-miR-29a vs Anti-miR-29a
	**Gene Symbol**	**Entrez Gene IDs**	**OMIM IDs**	**Gene Name**	**log2(fold_change)**	**log2(fold_change)**
316.	VAT1L	57687	No ID	vesicle amine transport 1 like	1.16268	1.0319
317.	AIM1	202	601797	absent in melanoma 1	0.778095	0.658569
318.	AKR1C3	8644	603966	aldo-keto reductase family 1 member C3	0.885048	0.645128
319.	ANKRD50	57182	No ID	ankyrin repeat domain 50	0.73071	0.554446
320.	ARL4C	10123	604787	ADP ribosylation factor like GTPase 4C	0.554121	0.695993
321.	ARPC5	10092	604227	actin related protein 2/3 complex subunit 5	0.586134	0.607786
322.	ATP1A1	476	182310	ATPase Na+/K+ transporting subunit alpha 1	0.64489	0.431066
323.	ATP5C1	509	108729	"ATP synthase, H+ transporting, mitochondrial F1 complex, gamma polypeptide 1"	0.6197	0.540511
324.	ATP7A	538	300011;300489;304150;309400	ATPase copper transporting alpha	0.798753	0.616152
325.	TMSB4X	7114	300159	"thymosin beta 4, X-linked"	0.559619	0.483911
326.	PGM5	5239	600981	phosphoglucomutase 5	2.04583	1.71094
327.	ANXA1	301	151690	annexin A1	0.86142	0.786981
328.	ANXA5	308	131230;614391	annexin A5	0.553169	0.52333
329.	ATRN	8455	603130	attractin	0.717643	0.492748
330.	BAK1P1	600	No ID	BCL2 antagonist/killer 1 pseudogene 1	0.72886	0.853615
331.	BMP5	653	112265	bone morphogenetic protein 5	0.836287	0.658327
332.	XXYLT1	152002	614552	xyloside xylosyltransferase 1	0.716947	0.799999
333.	CCDC167	154467	No ID	coiled-coil domain containing 167	0.573969	0.620072

334.	GINM1	116254	No ID	glycoprotein integral membrane 1	0.688129	0.599535
335.	CD276	80381	605715	CD276 molecule	0.6514	0.687137
336.	CD36	948	173510;248310;608404;610938;611162	CD36 molecule	0.991681	1.19904
337.	SCUBE2	57758	611747	"signal peptide, CUB domain and EGF like domain containing 2"	0.935904	0.815793
338.	CSGALNACT1	55790	616615	chondroitin sulfate N-acetylgalactosaminyltransferase 1	1.41892	1.27735
339.	CMBL	134147	613379	carboxymethylenebutenolidase homolog	0.765169	0.549379
340.	CRISP3	10321	No ID	cysteine rich secretory protein 3	1.1613	0.739149
341.	TET1	80312	607790	tet methylcytosine dioxygenase 1	1.14532	1.06875
342.	CYP19A1	1588	107910;139300;613546	cytochrome P450 family 19 subfamily A member 1	2.32583	2.35814
343.	RCN2	10231	604876	regulator of calcineurin 2	0.531026	0.452163
344.	CAV1	857	601047;606721;612526;615343	caveolin 1	1.16046	1.01584
345.	PPIC	5480	123842	peptidylprolyl isomerase C	1.21937	1.17803
346.	MIB1	57534	608677;615092	mindbomb E3 ubiquitin protein ligase 1	0.73351	0.521844
347.	DNAJC28	54943	No ID	DnaJ heat shock protein family (Hsp40) member C28	1.06775	0.92687
348.	FILIP1L	11259	612993	filamin A interacting protein 1 like	0.667145	0.583131
349.	DSC2	1824	125645;610476	desmocollin 2	1.29529	1.10679
350.	DYNLT1	6993	601554	dynein light chain Tctex-type 1	0.498623	0.60201
351.	CLEC7A	64581	606264;613108;614079	C-type lectin domain family 7 member A	1.28516	1.75287
352.	DSG2	1829	125671;610193;612877	desmoglein 2	0.743464	0.481257
353.	VOPP1	81552	611915	"vesicular, overexpressed in cancer, prosurvival protein 1"	0.476688	0.451507
354.	EPB41L4B	54566	610340	erythrocyte membrane protein band 4.1 like 4B	0.59199	0.530691

355.	ELF5	2001	605169	E74 like ETS transcription factor 5	1.20403	1.00089
356.	ENO1	2023	172430	enolase 1	0.499112	0.49841
357.	ERMP1	79956	611156	endoplasmic reticulum metallopeptidase 1	0.939574	0.679547
358.	EPHA6	285220	600066	EPH receptor A6	1.03363	0.877418
359.	EPHA7	2045	602190	EPH receptor A7	0.879505	0.701687
360.	FBP1	2203	229700;611570	fructose-bisphosphatase 1	0.45042	0.490642
361.	FAM102B	284611	No ID	family with sequence similarity 102 member B	0.61018	0.463769
362.	FAM127C	441518	No ID	family with sequence similarity 127 member C	0.511481	0.559729
363.	FECH	2235	177000;612386	ferrochelatase	1.03486	0.802485
364.	FKBP1A	2280	186945	FK506 binding protein 1A	0.72236	0.541505
365.	C4orf32	132720	No ID	chromosome 4 open reading frame 32	0.716821	0.62176
366.	EFEMP1	2202	126600;601548	EGF containing fibulin like extracellular matrix protein 1	0.943595	0.981479
367.	FREM2	341640	219000;608945	FRAS1 related extracellular matrix protein 2	1.2842	1.13966
368.	GALNT1	2589	602273	polypeptide N-acetylgalactosaminyltransferase 1	0.95207	0.767107
369.	GCSH	2653	238330;605899	glycine cleavage system protein H	0.812153	0.678064
370.	GSTM3	2946	138380	glutathione S-transferase mu 2	0.603347	0.474469
371.	HEPHL1	341208	No ID	hephaestin like 1	1.38329	1.14249
372.	MAP4K4	9448	604666	mitogen-activated protein kinase kinase kinase kinase 4	0.6531	0.563603
373.	HMGCLL1	54511	No ID	3-hydroxymethyl-3-methylglutaryl-CoA lyase like 1	1.15732	1.03274
374.	S100A16	388697	616293	hornerin	0.669323	0.682906
375.	HOXC13	3229	142976;614931	homeobox C13	0.546096	0.67975

376.	IGFBP5	3488	146734	insulin like growth factor binding protein 5	0.52921	0.390971
377.	IGSF3	3321	149700;603491	immunoglobulin superfamily member 3	0.98228	1.12764
378.	ACO1	48	100880	aconitase 1	0.950629	0.626089
379.	ISOC1	51015	No ID	isochorismatase domain containing 1	0.720805	0.671419
380.	ITGB1	3688	135630	integrin subunit beta 1	0.816957	0.659117
381.	F11R	50848	605721	F11 receptor	0.829893	0.805344
382.	JARID2	3720	601594	jumonji and AT-rich interaction domain containing 2	0.474666	0.444702
383.	KCNJ8	3764	239850;272120;600935	potassium voltage-gated channel subfamily J member 8	0.940332	0.848572
384.	KDELC1	79070	611613	KDEL motif containing 1	1.0655	1.27176
385.	KIAA1161	57462	No ID	KIAA1161	0.708471	0.59354
386.	KIAA1549	57670	613344	KIAA1549	0.770061	0.663556
387.	KLHDC3	116138	611248	kelch domain containing 3	0.612973	0.698181
388.	KLHL4	56062	300348	kelch like family member 4	1.07248	0.81465
389.	KLHL5	51088	608064	kelch like family member 5	0.756199	0.602252
390.	KRT23	25984	606194	keratin 23	0.859914	0.780114
391.	KRT80	144501	611161	keratin 80	0.387774	0.516173
392.	LTBP1	4052	150390	latent transforming growth factor beta binding protein 1	1.32021	0.921458
393.	MALL	7851	602022	"mal, T-cell differentiation protein like"	1.60014	1.58334
394.	MBTD1	54799	No ID	mbt domain containing 1	0.616126	0.501719
395.	SLC16A2	6567	300095;300523	solute carrier family 16 member 2	1.25596	0.854277
396.	MAP2K6	5608	601254	mitogen-activated protein kinase kinase 6	1.35071	1.27931
397.	METTL7A	25840	No ID	methyltransferase like 7A	0.81592	0.96939
398.	MIR17HG	407975	609415;614326	miR-17-92a-1 cluster host gene	2.37174	2.2729
399.	MPZL3	196264	611707	myelin protein zero like 3	0.796882	0.842667
400.	MRFAP1	93621	616905	Morf4 family associated protein 1	0.465383	0.536648
401.	MORF4L1	10933	607303	mortality factor 4 like 1	0.614757	0.563526

402.	NRAS	4893	114500;137550;162900;163200;164790;188470;249400;607785;613224;614470	neuroblastoma RAS viral oncogene homolog	0.552152	0.43652
403.	NAALADL2	254827	608806	N-acetylated alpha-linked acidic dipeptidase like 2	0.69365	0.67568
404.	SLC24A3	57419	609839	solute carrier family 24 member 3	0.589257	0.4987
405.	STK38	11329	606964	serine/threonine kinase 38	0.455075	0.426797
406.	NPNT	255743	610306	nephronectin	0.774074	0.579707
407.	NANOS1	340719	608226;615413	nanos C2HC-type zinc finger 1	0.858277	0.9082
408.	GS1-358P8.4;PDK3	5165	300905;300906	pyruvate dehydrogenase kinase 3	0.771457	0.564068
409.	PCP4	5121	601629	Purkinje cell protein 4	0.864802	0.712848
410.	PERP	64065	609301	"PERP, TP53 apoptosis effector"	0.587256	0.421452
411.	PIK3R3	8503	606076	phosphoinositide-3-kinase regulatory subunit 3	0.801967	0.829185
412.	C7orf73	647087	No ID	chromosome 7 open reading frame 73	0.803039	0.703753
413.	KDM5B	10765	605393	lysine demethylase 5B	0.98413	0.779044
414.	BPGM	669	222800;613896	bisphosphoglycerate mutase	0.744332	0.734537
415.	PPT1	5538	256730;600722	palmitoyl-protein thioesterase 1	0.742549	0.688119
416.	PRMT6	55170	608274	protein arginine methyltransferase 6	0.576114	0.729643
417.	PSG9	5678	176398	pregnancy specific beta-1-glycoprotein 9	0.84128	0.757355
418.	PANX1	24145	608420	pannexin 1	0.666987	0.487432
419.	PKP1	5317	601975;604536	plakophilin 1	1.25349	1.28295
420.	RPS24	6229	602412;610629	ribosomal protein S24	0.555705	0.488549
421.	RPS3A	6189	180478	ribosomal protein S3A	0.687786	0.491714
422.	RTN3	10313	604249	reticulon 3	0.612048	0.503042
423.	S100A10	6281	114085	S100 calcium binding protein A10	0.574568	0.414773
424.	SERINC5	256987	614551	serine incorporator 5	0.805837	0.548127
425.	SESTD1	91404	No ID	SEC14 and spectrin domain containing 1	1.10416	1.06537

426.	SLITRK6	84189	221200;609681	SLIT and NTRK like family member 6	0.786628	0.553729
427.	TMTC3	160418	No ID	transmembrane and tetratricopeptide repeat containing 3	0.828496	0.643048
428.	SCIN	85477	613416	scinderin	1.3387	1.19559
429.	SEMA3C	10512	602645	semaphorin 3C	0.865222	0.66281
430.	SEMA3D	223117	609907	semaphorin 3D	1.48777	1.16477
431.	SORT1	6272	602458;613589	sortilin 1	0.749978	0.522416
432.	STON1	11037	605357	stonin 1	1.5401	1.4028
433.	TBC1D7	51256	248000;612655	TBC1 domain family member 7	0.921924	0.83745
434.	RCC2	55920	609587	regulator of chromosome condensation 2	0.401982	0.591432
435.	TDG	6996	601423	thymine DNA glycosylase	0.815785	0.81763
436.	TET3	200424	613555	tet methylcytosine dioxygenase 3	0.923868	1.01061
437.	TMEM117	84216	No ID	transmembrane protein 117	1.76039	1.52825
438.	TMEM164	84187	No ID	transmembrane protein 164	0.8879	0.803582
439.	TMEM45B	120224	No ID	transmembrane protein 45B	0.797278	0.752252
440.	TOP2B	7155	126431	topoisomerase (DNA) II beta	0.707055	0.519007
441.	NTRK2	4915	600456;613886	neurotrophic receptor tyrosine kinase 2	0.987398	1.21638
442.	UBTD2	92181	610174	ubiquitin domain containing 2	0.753085	0.627815
443.	UNC5C	8633	603610	unc-5 netrin receptor C	1.2494	1.47876
444.	VAMP7	6845	300053	vesicle associated membrane protein 7	0.950366	0.81555
445.	WNT2B	7482	601968	Wnt family member 2B	1.1319	1.0473
446.	TRAK2	66008	607334	trafficking kinesin protein 2	0.706495	0.568506
447.	KIT	3815	154800;164920;172800;273300;601626;606764	KIT proto-oncogene receptor tyrosine kinase	1.99239	1.71144

Supplementary Table 1C: miR-29a unique targets in MCF-7 cells

					MCF-7_Pre-miR-29a vs Anti-miR-29a
	**Gene Symbol**	**Entrez Gene IDs**	**OMIM IDs**	**Gene Name**	**log2(fold_change)**
448.	CDR1	1038	302650	cerebellar degeneration related protein 1	1.25927
449.	CASP14	23581	605848	caspase 14	3.26142
450.	DNMT3B	1789	242860;602900	DNA methyltransferase 3 beta	0.592581
451.	SGK196	84197	615247;615249;616094	protein-O-mannose kinase	0.792492
452.	SERPINH1	871	600943;610504;613848	serpin family H member 1	0.691306
453.	NRBP1	29959	606010	nuclear receptor binding protein 1	0.446701
454.	ZNF469	84627	229200;612078	zinc finger protein 469	0.819815

Supplementary Table 2: miR-29 targets in LCC9 cells

Supplementary Table 2A: miR-29b-1 unique targets in LCC9 cells

	Input IDs	Gene Symbol	Gene Name	LCC9_Pre-miR-29b1 vs Anti-miR-29a log2(fold_change)
1.	ENSG00000106049	HIBADH	3-hydroxyisobutyrate dehydrogenase	0.491816
2.	ENSG00000150756	FAM173B	family with sequence similarity 173 member B	0.610799
3.	ENSG00000204574	ABCF1	ATP binding cassette subfamily F member 1	0.404991
4.	ENSG00000197150	ABCB8	ATP binding cassette subfamily B member 8	0.490734
5.	ENSG00000060971	ACAA1	acetyl-CoA acyltransferase 1	0.400058
6.	ENSG00000141385	AFG3L2	AFG3 like matrix AAA peptidase subunit 2	0.728778
7.	ENSG00000188266	HYKK	hydroxylysine kinase	0.511482
8.	ENSG00000155085	AK9	adenylate kinase 9	0.652831
9.	ENSG00000187134	AKR1C1	aldo-keto reductase family 1 member C1	0.648362
10.	ENSG00000196139	AKR1C3	aldo-keto reductase family 1 member C3	0.556808
11.	ENSG00000172339	ALG14	"ALG14, UDP-N-acetylglucosaminyltransferase subunit"	0.826509
12.	ENSG00000137760	ALKBH8	"alkB homolog 8, tRNA methyltransferase"	0.63568
13.	ENSG00000088448	ANKRD10	ankyrin repeat domain 10	0.692931
14.	ENSG00000132623	ANKEF1	ankyrin repeat and EF-hand domain containing 1	0.491522
15.	ENSG00000116819	TFAP2E	transcription factor AP-2 epsilon	1.24266
16.	ENSG00000149089	APIP	APAF1 interacting protein	0.585429
17.	ENSG00000169621	APLF	aprataxin and PNKP like factor	0.666282
18.	ENSG00000113966	ARL6	ADP ribosylation factor like GTPase 6	0.590646
19.	ENSG00000143437	ARNT	aryl hydrocarbon receptor nuclear translocator	0.576123
20.	ENSG00000130707	ASS1	argininosuccinate synthase 1	0.569205
21.	ENSG00000176208	ATAD5	"ATPase family, AAA domain containing 5"	0.409172
22.	ENSG00000115966	ATF2	activating transcription factor 2	0.636007
23.	ENSG00000122507	BBS9	Bardet-Biedl syndrome 9	0.732789
24.	ENSG00000236824	BCYRN1	brain cytoplasmic RNA 1	0.733708
25.	ENSG00000136573	BLK	"BLK proto-oncogene, Src family tyrosine kinase"	1.73238
26.	ENSG00000184992	BRI3BP	BRI3 binding protein	0.388747
27.	ENSG00000074317	SNCB	synuclein beta	1.28303
28.	ENSG00000122378	FAM213A	family with sequence similarity 213 member A	0.500409
29.	ENSG00000022277	RTFDC1	replication termination factor 2 domain containing 1	0.382306
30.	ENSG00000184809	B3GALT5-AS1	B3GALT5 antisense RNA 1	1.15966
31.	ENSG00000172478	C2orf54	chromosome 2 open reading frame 54	1.28875
32.	ENSG00000196821	C6orf106	chromosome 6 open reading frame 106	0.542815
33.	ENSG00000203778	FAM229B	family with sequence similarity 229 member B	0.720804
34.	ENSG00000147894	C9orf72	chromosome 9 open reading frame 72	0.647372
35.	ENSG00000003989	SLC7A2	solute carrier family 7 member 2	0.476024
36.	ENSG00000172785	CBWD1	COBW domain containing 1	0.417456
37.	ENSG00000163001	CFAP36	cilia and flagella associated protein 36	0.533663
38.	ENSG00000109881	CCDC34	coiled-coil domain containing 34	0.651414
39.	ENSG00000141519	CCDC40	coiled-coil domain containing 40	1.16175
40.	ENSG00000173588	CEP83	centrosomal protein 83	0.710338
41.	ENSG00000081377	CDC14B	cell division cycle 14B	0.657699
42.	ENSG00000100526	CDKN3	cyclin dependent kinase inhibitor 3	0.378184
43.	ENSG00000166446	CDYL2	chromodomain Y-like 2	0.756545
44.	ENSG00000114107	CEP70	centrosomal protein 70	0.527855
45.	ENSG00000188153	COL4A5	collagen type IV alpha 5 chain	1.26463
46.	ENSG00000138663	COPS4	COP9 signalosome subunit 4	0.575052
47.	ENSG00000111652	COPS7A	COP9 signalosome subunit 7A	0.470495
48.	ENSG00000096006	CRISP3	cysteine rich secretory protein 3	0.74899
49.	ENSG00000049656	CLPTM1L	CLPTM1 like	0.516426
50.	ENSG00000145681	HAPLN1	hyaluronan and proteoglycan link protein 1	4.09352
51.	ENSG00000124207	CSE1L	chromosome segregation 1 like	0.396727
52.	ENSG00000150527	CTAGE5	"CTAGE family member 5, ER export factor"	0.479836
53.	ENSG00000137770	CTDSPL2	CTD small phosphatase like 2	0.660265
54.	ENSG00000111249	CUX2	cut like homeobox 2	0.772002

55.	ENSG00000161921	CXCL16	C-X-C motif chemokine ligand 16	0.654154
56.	ENSG00000174437	ATP2A2	ATPase sarcoplasmic/endoplasmic reticulum Ca2+ transporting 2	0.464372
57.	ENSG00000152495	CAMK4	calcium/calmodulin dependent protein kinase IV	1.09332
58.	ENSG00000132906	CASP9	caspase 9	0.586727
59.	ENSG00000150764	DIXDC1	DIX domain containing 1	0.874583
60.	ENSG00000167670	CHAF1A	chromatin assembly factor 1 subunit A	0.759801
61.	ENSG00000111907	TPD52L1	tumor protein D52-like 1	0.484994
62.	ENSG00000134107	BHLHE40	basic helix-loop-helix family member e40	0.5102
63.	ENSG00000155792	DEPTOR	DEP domain containing MTOR-interacting protein	1.01048
64.	ENSG00000136044	APPL2	"adaptor protein, phosphotyrosine interacting with PH domain and leucine zipper 2"	0.847216
65.	ENSG00000126698	DNAJC8	DnaJ heat shock protein family (Hsp40) member C8	0.444634
66.	ENSG00000189212	DPY19L2P1	DPY19L2 pseudogene 1	0.557364
67.	ENSG00000165891	E2F7	E2F transcription factor 7	0.580426
68.	ENSG00000164330	EBF1	early B-cell factor 1	2.01344
69.	ENSG00000109381	ELF2	E74 like ETS transcription factor 2	0.584111
70.	ENSG00000138185	ENTPD1	ectonucleoside triphosphate diphosphohydrolase 1	0.51802
71.	ENSG00000187672	ERC2	ELKS/RAB6-interacting/CAST family member 2	0.765149
72.	ENSG00000170515	PA2G4	proliferation-associated 2G4	0.534069
73.	ENSG00000185900	POMK	protein-O-mannose kinase	0.865861
74.	ENSG00000133216	EPHB2	EPH receptor B2	0.546443
75.	ENSG00000031003	FAM13B	family with sequence similarity 13 member B	0.523452
76.	ENSG00000154511	FAM69A	family with sequence similarity 69 member A	0.569141
77.	ENSG00000158169	FANCC	Fanconi anemia complementation group C	0.900585
78.	ENSG00000160752	FDPS	farnesyl diphosphate synthase	0.65476
79.	ENSG00000169018	FEM1B	fem-1 homolog B	0.807097
80.	ENSG00000197296	FITM2	fat storage inducing transmembrane protein 2	0.374061
81.	ENSG00000198468	FLVCR1-AS1	FLVCR1 antisense RNA 1 (head to head)	0.934593
82.	ENSG00000138759	FRAS1	Fraser extracellular matrix complex subunit 1	0.825708
83.	ENSG00000150667	FSIP1	fibrous sheath interacting protein 1	4.68256
84.	ENSG00000114450	GNB4	G protein subunit beta 4	0.651437
85.	ENSG00000242616	GNG10	G protein subunit gamma 10	0.677874
86.	ENSG00000172380	GNG12	G protein subunit gamma 12	0.792578
87.	ENSG00000164949	GEM	GTP binding protein overexpressed in skeletal muscle	0.570737
88.	ENSG00000198814	GK	glycerol kinase	1.29919
89.	ENSG00000130309	COLGALT1	collagen beta(1-O)galactosyltransferase 1	0.676398
90.	ENSG00000073605	GSDMB	gasdermin B	0.835803
91.	ENSG00000226259	GTF2H2B	general transcription factor IIH subunit 2B (pseudogene)	1.24475
92.	ENSG00000234741	GAS5	growth arrest specific 5 (non-protein coding)	0.584347
93.	ENSG00000179409	GEMIN4	gem nuclear organelle associated protein 4	0.498702
94.	ENSG00000175697	GPR156	G protein-coupled receptor 156	0.564154
95.	ENSG00000249115	HAUS5	HAUS augmin like complex subunit 5	0.562282
96.	ENSG00000048052	HDAC9	histone deacetylase 9	1.17183
97.	ENSG00000206053	HN1L	hematological and neurological expressed 1 like	0.716046
98.	ENSG00000149428	HYOU1	hypoxia up-regulated 1	0.37912
99.	ENSG00000146674	IGFBP3	insulin like growth factor binding protein 3	1.78987
100.	ENSG00000032742	IFT88	intraflagellar transport 88	0.561653
101.	ENSG00000243646	IL10RB	interleukin 10 receptor subunit beta	1.09275
102.	ENSG00000016402	IL20RA	interleukin 20 receptor subunit alpha	0.687926
103.	ENSG00000148950	IMMP1L	inner mitochondrial membrane peptidase subunit 1	1.24312
104.	ENSG00000240682	ISY1	ISY1 splicing factor homolog	0.766524
105.	ENSG00000082781	ITGB5	integrin subunit beta 5	0.537403
106.	ENSG00000140057	AK7	adenylate kinase 7	0.986999
107.	ENSG00000140854	KATNB1	katanin regulatory subunit B1	0.560459
108.	ENSG00000122778	KIAA1549	KIAA1549	0.738185
109.	ENSG00000102271	KLHL4	kelch like family member 4	0.978512
110.	ENSG00000118162	KPTN	"kaptin, actin binding protein"	0.648915
111.	ENSG00000102781	KATNAL1	katanin catalytic subunit A1 like 1	0.545075
112.	ENSG00000170523	KRT83	keratin 83	0.821228
113.	ENSG00000170745	KCNS3	potassium voltage-gated channel modifier subfamily S member 3	0.504918
114.	ENSG00000131023	LATS1	large tumor suppressor kinase 1	0.454589
115.	ENSG00000134333	LDHA	lactate dehydrogenase A	0.556593

116.	ENSG00000179241	LDLRAD3	low density lipoprotein receptor class A domain containing 3	0.659617
117.	ENSG00000117114	ADGRL2	adhesion G protein-coupled receptor L2	1.38087
118.	ENSG00000235823	OLMALINC	oligodendrocyte maturation-associated long intergenic non-coding RNA	0.489124
119.	ENSG00000145832	SLC25A48	solute carrier family 25 member 48	2.62358
120.	ENSG00000213468	FIRRE	firre intergenic repeating RNA element	0.60146
121.	ENSG00000237732	LOC440934	uncharacterized LOC440934	1.06641
122.	ENSG00000236850	BMS1P20	"BMS1, ribosome biogenesis factor pseudogene 20"	0.42462
123.	ENSG00000198121	LPAR1	lysophosphatidic acid receptor 1	1.78063
124.	ENSG00000117600	PLPPR4	phospholipid phosphatase related 4	2.03628
125.	ENSG00000168904	LRRC28	leucine rich repeat containing 28	0.44925
126.	ENSG00000066557	LRRC40	leucine rich repeat containing 40	0.368294
127.	ENSG00000106853	PTGR1	prostaglandin reductase 1	0.548995
128.	ENSG00000049323	LTBP1	latent transforming growth factor beta binding protein 1	0.591944
129.	ENSG00000178802	MPI	mannose phosphate isomerase	0.488671
130.	ENSG00000116586	LAMTOR2	"late endosomal/lysosomal adaptor, MAPK and MTOR activator 2"	0.365329
131.	ENSG00000166974	MAPRE2	microtubule associated protein RP/EB family member 2	1.40219
132.	ENSG00000076003	MCM6	minichromosome maintenance complex component 6	0.531703
133.	ENSG00000065833	ME1	malic enzyme 1	0.423041
134.	ENSG00000175581	MRPL48	mitochondrial ribosomal protein L48	0.562058
135.	ENSG00000048544	MRPS10	mitochondrial ribosomal protein S10	0.366277
136.	ENSG00000164953	TMEM67	transmembrane protein 67	0.890988
137.	ENSG00000147166	ITGB1BP2	integrin subunit beta 1 binding protein 2	1.7052
138.	ENSG00000126858	RHOT1	ras homolog family member T1	0.621903
139.	ENSG00000078177	N4BP2	NEDD4 binding protein 2	0.775605
140.	ENSG00000228224	NACAP1	nascent polypeptide associated complex alpha subunit pseudogene 1	0.852377
141.	ENSG00000125814	NAPB	NSF attachment protein beta	0.41552
142.	ENSG00000110583	NAA40	"N(alpha)-acetyltransferase 40, NatD catalytic subunit"	0.93559
143.	ENSG00000196290	NIF3L1	NGG1 interacting factor 3 like 1	0.477036
144.	ENSG00000176171	BNIP3	BCL2 interacting protein 3	0.400854
145.	ENSG00000113389	NPR3	natriuretic peptide receptor 3	0.731253
146.	ENSG00000184117	NIPSNAP1	nipsnap homolog 1 (C. elegans)	0.419627
147.	ENSG00000084628	NKAIN1	Na+/K+ transporting ATPase interacting 1	0.930873
148.	ENSG00000218336	TENM3	teneurin transmembrane protein 3	0.946142
149.	ENSG00000122884	P4HA1	prolyl 4-hydroxylase subunit alpha 1	0.670883
150.	ENSG00000180228	PRKRA	protein activator of interferon induced protein kinase EIF2AK2	0.415149
151.	ENSG00000141127	PRPSAP2	phosphoribosyl pyrophosphate synthetase associated protein 2	0.413774
152.	ENSG00000177839	PCDHB9	protocadherin beta 9	0.795703
153.	ENSG00000205268	PDE7A	phosphodiesterase 7A	0.626122
154.	ENSG00000170962	PDGFD	platelet derived growth factor D	1.08647
155.	ENSG00000100227	POLDIP3	DNA polymerase delta interacting protein 3	0.621359
156.	ENSG00000198721	ECI2	enoyl-CoA delta isomerase 2	0.724378
157.	ENSG00000173868	PHOSPHO1	phosphoethanolamine/phosphocholine phosphatase	1.0788
158.	ENSG00000169756	LIMS1	LIM zinc finger domain containing 1	0.507993
159.	ENSG00000005249	PRKAR2B	protein kinase cAMP-dependent type II regulatory subunit beta	0.96804
160.	ENSG00000169621	PROKR1	prokineticin receptor 1	0.666282
161.	ENSG00000106086	PLEKHA8	pleckstrin homology domain containing A8	0.54894
162.	ENSG00000014138	POLA2	"DNA polymerase alpha 2, accessory subunit"	0.465248
163.	ENSG00000144231	POLR2D	RNA polymerase II subunit D	0.50258
164.	ENSG00000185238	PRMT3	protein arginine methyltransferase 3	0.399367
165.	ENSG00000166033	HTRA1	HtrA serine peptidase 1	0.448859
166.	ENSG00000121766	ZCCHC17	zinc finger CCHC-type containing 17	0.745644
167.	ENSG00000138430	OLA1	Obg like ATPase 1	0.454699
168.	ENSG00000139304	PTPRQ	"protein tyrosine phosphatase, receptor type Q"	1.30696
169.	ENSG00000091127	PUS7	pseudouridylate synthase 7 (putative)	1.05676

170.	ENSG00000059573	ALDH18A1	aldehyde dehydrogenase 18 family member A1	0.531767
171.	ENSG00000004399	PLXND1	plexin D1	0.785074
172.	ENSG00000143801	PSEN2	presenilin 2	0.577816
173.	ENSG00000089050	RBBP9	"RB binding protein 9, serine hydrolase"	0.62783
174.	ENSG00000213516	RBMXL1	"RNA binding motif protein, X-linked like 1"	0.83167
175.	ENSG00000162444	RBP7	retinol binding protein 7	0.731566
176.	ENSG00000122707	RECK	reversion inducing cysteine rich protein with kazal motifs	0.634226
177.	ENSG00000203668	CHML	"CHM like, Rab escort protein 2"	0.51309
178.	ENSG00000111404	RERGL	RERG like	2.21964
179.	ENSG00000165731	RET	ret proto-oncogene	0.50531
180.	ENSG00000121481	RNF2	ring finger protein 2	0.509205
181.	ENSG00000180530	NRIP1	nuclear receptor interacting protein 1	0.37917
182.	ENSG00000110315	RNF141	ring finger protein 141	0.670646
183.	ENSG00000198242	RPL23A	ribosomal protein L23a	0.381132
184.	ENSG00000089009	RPL6	ribosomal protein L6	0.636679
185.	ENSG00000146223	RPL7L1	ribosomal protein L7 like 1	0.477004
186.	ENSG00000139998	RAB15	"RAB15, member RAS oncogene family"	1.00517
187.	ENSG00000133318	RTN3	reticulon 3	0.404803
188.	ENSG00000140943	MBTPS1	"membrane bound transcription factor peptidase, site 1"	0.791293
189.	ENSG00000092108	SCFD1	sec1 family domain containing 1	0.462588
190.	ENSG00000102098	SCML2	sex comb on midleg-like 2 (Drosophila)	0.614317
191.	ENSG00000143653	SCCPDH	saccharopine dehydrogenase (putative)	0.383584
192.	ENSG00000086475	SEPHS1	selenophosphate synthetase 1	0.581941
193.	ENSG00000196136	SERPINA3	serpin family A member 3	0.834504
194.	ENSG00000176974	SHMT1	serine hydroxymethyltransferase 1	0.464031
195.	ENSG00000146414	SHPRH	SNF2 histone linker PHD RING helicase	0.631553
196.	ENSG00000072858	SIDT1	SID1 transmembrane family member 1	0.514638
197.	ENSG00000179542	SLITRK4	SLIT and NTRK like family member 4	0.584921
198.	ENSG00000139324	TMTC3	transmembrane and tetratricopeptide repeat containing 3	0.659666
199.	ENSG00000188176	SMTNL2	smoothelin like 2	1.49332
200.	ENSG00000132639	SNAP25	synaptosome associated protein 25	2.16937
201.	ENSG00000129673	AANAT	aralkylamine N-acetyltransferase	2.09567
202.	ENSG00000112335	SNX3	sorting nexin 3	0.378761
203.	ENSG00000145375	SPATA5	spermatogenesis associated 5	0.491729
204.	ENSG00000149136	SSRP1	structure specific recognition protein 1	0.392846
205.	ENSG00000110066	KMT5B	lysine methyltransferase 5B	0.482419
206.	ENSG00000159164	SV2A	synaptic vesicle glycoprotein 2A	0.398681
207.	ENSG00000006747	SCIN	scinderin	0.478395
208.	ENSG00000150961	SEC24D	"SEC24 homolog D, COPII coat complex component"	0.852999
209.	ENSG00000167680	SEMA6B	semaphorin 6B	0.508275
210.	ENSG00000198879	SFMBT2	Scm-like with four mbt domains 2	0.390137
211.	ENSG00000187164	SHTN1	shootin 1	0.496706
212.	ENSG00000150753	CCT5	chaperonin containing TCP1 subunit 5	0.621055
213.	ENSG00000204852	TCTN1	tectonic family member 1	0.814604
214.	ENSG00000083544	TDRD3	tudor domain containing 3	0.599608
215.	ENSG00000187735	TCEA1	transcription elongation factor A1	0.363363
216.	ENSG00000118707	TGIF2	TGFB induced factor homeobox 2	0.91099
217.	ENSG00000111602	TIMELESS	timeless circadian clock	0.468897
218.	ENSG00000100234	TIMP3	TIMP metallopeptidase inhibitor 3	0.884252
219.	ENSG00000185650	ZFP36L1	ZFP36 ring finger protein like 1	0.363495
220.	ENSG00000164841	TMEM74	transmembrane protein 74	0.659316
221.	ENSG00000168234	TTC39C	tetratricopeptide repeat domain 39C	0.645904
222.	ENSG00000114999	TTL	tubulin tyrosine ligase	0.436904
223.	ENSG00000253352	TUG1	taurine up-regulated 1 (non-protein coding)	0.663468
224.	ENSG00000102241	HTATSF1	HIV-1 Tat specific factor 1	0.438483
225.	ENSG00000140534	TICRR	TOPBP1 interacting checkpoint and replication regulator	0.49207
226.	ENSG00000152086	TUBA3E	tubulin alpha 3e	2.34108
227.	ENSG00000123416	TUBA1B	tubulin alpha 1b	0.396279
228.	ENSG00000074935	TUBE1	tubulin epsilon 1	0.587981
229.	ENSG00000117143	UAP1	UDP-N-acetylglucosamine pyrophosphorylase 1	0.455078
230.	ENSG00000078967	UBE2D4	ubiquitin conjugating enzyme E2 D4 (putative)	0.778811
231.	ENSG00000165816	VWA2	von Willebrand factor A domain containing 2	0.928517

232.	ENSG00000132970	WASF3	WAS protein family member 3	0.629683
233.	ENSG00000065183	WDR3	WD repeat domain 3	0.500013
234.	ENSG00000176105	YES1	"YES proto-oncogene 1, Src family tyrosine kinase"	0.431171
235.	ENSG00000257267	ZNF271P	"zinc finger protein 271, pseudogene"	0.497983
236.	ENSG00000130844	ZNF331	zinc finger protein 331	0.516318
237.	ENSG00000160094	ZNF362	zinc finger protein 362	0.911363
238.	ENSG00000142556	ZNF614	zinc finger protein 614	0.740139
239.	ENSG00000186777	ZNF732	zinc finger protein 732	0.491996
240.	ENSG00000091428	RAPGEF4	Rap guanine nucleotide exchange factor 4	1.03583
241.	ENSG00000138660	AP1AR	adaptor related protein complex 1 associated regulatory protein	1.03233
242.	ENSG00000256806	C17orf100	chromosome 17 open reading frame 100	1.62273
243.	ENSG00000207652	MIR621	microRNA 621	1.4975

Supplementary Table 2B: Common miR-29b-1 and miR-29a targets in LCC9 cells

				LCC9_Pre-miR-29b1 vs Anti-miR-29a	LCC9_Pre-miR-29a vs Anti-miR-29a
	Input IDs	Gene Symbol	Gene Name	log2(fold_change)	log2(fold_change)
	ENSG00000108953	YWHAE	tyrosine 3-monooxygenase/tryptophan 5-monooxygenase activation protein epsilon	0.487217	0.457738
	ENSG00000223414	LINC00473	long intergenic non-protein coding RNA 473	4.3032	5.28493
	ENSG00000172243	CLEC7A	C-type lectin domain family 7 member A	2.31239	4.17419
	ENSG00000152661	GJA1	gap junction protein alpha 1	4.11427	3.86025
	ENSG00000168306	ACOX2	acyl-CoA oxidase 2	4.65734	3.66441
	ENSG00000187678	SPRY4	sprouty RTK signaling antagonist 4	2.72265	3.40137
	ENSG00000198937	CCDC167	coiled-coil domain containing 167	3.39142	3.38646
	ENSG00000113140	SPARC	secreted protein acidic and cysteine rich	2.76873	3.35492
	ENSG00000150779	TIMM8B	translocase of inner mitochondrial membrane 8 homolog B	2.80661	3.09556
	ENSG00000143546	S100A8	S100 calcium binding protein A8	1.92461	3.08523
	ENSG00000175832	ETV4	ETS variant 4	2.98963	2.98716
	ENSG00000128271	ADORA2A	adenosine A2a receptor	2.30518	2.9572
	ENSG00000145979	TBC1D7	TBC1 domain family member 7	2.64112	2.93259
	ENSG00000144891	AGTR1	angiotensin II receptor type 1	2.79834	2.8424
	ENSG00000064205	WISP2	WNT1 inducible signaling pathway protein 2	2.80975	2.83438
	ENSG00000169181	GSG1L	GSG1 like	3.08116	2.83152
	ENSG00000163485	ADORA1	adenosine A1 receptor	2.51826	2.81189
	ENSG00000160050	CCDC28B	coiled-coil domain containing 28B	2.65578	2.80182
	ENSG00000134901	KDELC1	KDEL motif containing 1	3.01117	2.79997
	ENSG00000071575	TRIB2	tribbles pseudokinase 2	2.8007	2.78841
	ENSG00000174453	VWC2L	von Willebrand factor C domain containing protein 2-like	3.02552	2.7854
	ENSG00000123560	PLP1	proteolipid protein 1	1.78971	2.78286
	ENSG00000198890	PRMT6	protein arginine methyltransferase 6	2.51833	2.72602
	ENSG00000126010	GRPR	gastrin releasing peptide receptor	2.54854	2.69536
	ENSG00000116774	OLFML3	olfactomedin like 3	2.60869	2.68797
	ENSG00000244405	ETV5	ETS variant 5	1.71214	2.63838
	ENSG00000162892	IL24	interleukin 24	2.76179	2.61736
	ENSG00000064995	TAF11	TATA-box binding protein associated factor 11	2.53393	2.61299
	ENSG00000164120	HPGD	hydroxyprostaglandin dehydrogenase 15-(NAD)	2.94784	2.59741
	ENSG00000105671	DDX49	DEAD-box helicase 49	2.34706	2.59104
	ENSG00000163220	S100A9	S100 calcium binding protein A9	1.53666	2.52338
	ENSG00000164761	TNFRSF11B	TNF receptor superfamily member 11b	2.27648	2.51838
	ENSG00000179051	RCC2	regulator of chromosome condensation 2	2.40005	2.4671
	ENSG00000132821	VSTM2L	V-set and transmembrane domain containing 2 like	2.51467	2.41676
	ENSG00000105967	TFEC	transcription factor EC	2.11704	2.40168
	ENSG00000142224	IL19	interleukin 19	3.06232	2.39884
	ENSG00000104147	OIP5	Opa interacting protein 5	2.11953	2.39693
	ENSG00000092208	GEMIN2	gem nuclear organelle associated protein 2	2.09195	2.368
	ENSG00000136783	NIPSNAP3A	nipsnap homolog 3A	1.98433	2.36503
	ENSG00000138326	RPS24	ribosomal protein S24	2.25259	2.36119
	ENSG00000198856	OSTC	oligosaccharyltransferase complex non-catalytic subunit	2.19415	2.35898
	ENSG00000139372	TDG	thymine DNA glycosylase	2.25669	2.35879
	ENSG00000163347	CLDN1	claudin 1	1.59109	2.35224
	ENSG00000166106	ADAMTS15	ADAM metallopeptidase with thrombospondin type 1 motif 15	2.63156	2.34575
	ENSG00000256870	SLC5A8	solute carrier family 5 member 8	2.78152	2.31415
	ENSG00000183036	PCP4	Purkinje cell protein 4	2.79053	2.29124
	ENSG00000260231	JHDM1D-AS1	JHDM1D antisense RNA 1 (head to head)	1.91105	2.28899
	ENSG00000173950	XXYLT1	xyloside xylosyltransferase 1	2.16137	2.27884
	ENSG00000179388	EGR3	early growth response 3	2.35585	2.26571
	ENSG00000132004	FBXW9	F-box and WD repeat domain containing 9	1.83408	2.26133
	ENSG00000123364	HOXC13	homeobox C13	2.13121	2.22351
	ENSG00000158164	TMSB15A	thymosin beta 15a	2.53916	2.21981
	ENSG00000169248	CXCL11	C-X-C motif chemokine ligand 11	2.02203	2.21208

	ENSG00000242265	PEG10	paternally expressed 10	2.10984	2.21207
	ENSG00000137441	FGFBP2	fibroblast growth factor binding protein 2	2.90166	2.19779
	ENSG00000135046	ANXA1	annexin A1	2.13207	2.17304
	ENSG00000170855	TRIAP1	TP53 regulated inhibitor of apoptosis 1	2.00172	2.1629
	ENSG00000168938	PPIC	peptidylprolyl isomerase C	2.04242	2.15937
	ENSG00000135346	CGA	"glycoprotein hormones, alpha polypeptide"	1.43795	2.15628
	ENSG00000108984	MAP2K6	mitogen-activated protein kinase kinase 6	2.61089	2.15353
	ENSG00000006625	GGCT	gamma-glutamylcyclotransferase	2.08826	2.14783
	ENSG00000146425	DYNLT1	dynein light chain Tctex-type 1	1.94914	2.14702
	ENSG00000101057	MYBL2	MYB proto-oncogene like 2	2.03609	2.14457
	ENSG00000179010	MRFAP1	Morf4 family associated protein 1	2.03319	2.14254
	ENSG00000108578	BLMH	bleomycin hydrolase	2.51864	2.13864
	ENSG00000139055	ERP27	endoplasmic reticulum protein 27	1.63098	2.08328
	ENSG00000075290	WNT8B	Wnt family member 8B	1.5311	2.08131
	ENSG00000226950	DANCR	differentiation antagonizing non-protein coding RNA	1.67735	2.07894
	ENSG00000124702	KLHDC3	kelch domain containing 3	1.94245	2.07298
	ENSG00000149257	SERPINH1	serpin family H member 1	1.97041	2.06858
	ENSG00000121005	CRISPLD1	cysteine rich secretory protein LCCL domain containing 1	2.05979	2.05316
	ENSG00000108511	HOXB6	homeobox B6	1.8322	2.04239
	ENSG00000106484	MEST	mesoderm specific transcript	2.28148	2.02826
	ENSG00000198729	PPP1R14C	protein phosphatase 1 regulatory inhibitor subunit 14C	2.03073	2.02062
	ENSG00000170899	GSTA4	glutathione S-transferase alpha 4	1.77556	2.01732
	ENSG00000153012	LGI2	leucine rich repeat LGI family member 2	2.18862	2.00029
	ENSG00000140905	GCSH	glycine cleavage system protein H	2.03522	1.98461
	ENSG00000168256	NKIRAS2	NFKB inhibitor interacting Ras like 2	1.79244	1.98048
	ENSG00000233101	HOXB-AS3	HOXB cluster antisense RNA 3	2.07946	1.9725
	ENSG00000157404	KIT	KIT proto-oncogene receptor tyrosine kinase	2.10612	1.97156
	ENSG00000144451	SPAG16	sperm associated antigen 16	2.78745	1.96532
	ENSG00000164362	TERT	telomerase reverse transcriptase	1.89687	1.94267
	ENSG00000144857	BOC	"BOC cell adhesion associated, oncogene regulated"	2.16198	1.93153
	ENSG00000120875	DUSP4	dual specificity phosphatase 4	2.05306	1.92245
	ENSG00000170465	KRT6C	keratin 6C	1.27008	1.92117
	ENSG00000119929	CUTC	cutC copper transporter	1.76035	1.9119
	ENSG00000178585	CTNNBIP1	catenin beta interacting protein 1	1.85451	1.90993
	ENSG00000181788	SIAH2	siah E3 ubiquitin protein ligase 2	1.85018	1.90909
	ENSG00000078725	BRINP1	BMP/retinoic acid inducible neural specific 1	1.72664	1.90507
	ENSG00000243317	C7orf73	chromosome 7 open reading frame 73	1.89354	1.90098
	ENSG00000146409	SLC18B1	solute carrier family 18 member B1	1.68871	1.89729
	ENSG00000035141	FAM136A	family with sequence similarity 136 member A	1.77743	1.89115
	ENSG00000145632	PLK2	polo like kinase 2	2.01513	1.87612
	ENSG00000174721	FGFBP3	fibroblast growth factor binding protein 3	1.23721	1.87457
	ENSG00000066583	ISOC1	isochorismatase domain containing 1	1.88871	1.87293
	ENSG00000165806	CASP7	caspase 7	1.81119	1.87049
	ENSG00000169282	KCNAB1	potassium voltage-gated channel subfamily A member regulatory beta subunit 1	2.691	1.84939
	ENSG00000167536	DHRS13	dehydrogenase/reductase 13	1.78083	1.83441
	ENSG00000135930	EIF4E2	eukaryotic translation initiation factor 4E family member 2	1.81313	1.83072
	ENSG00000114646	CSPG5	chondroitin sulfate proteoglycan 5	1.77322	1.8202
	ENSG00000131153	GINS2	GINS complex subunit 2	1.68133	1.7959
	ENSG00000137965	IFI44	interferon induced protein 44	1.07807	1.79376
	ENSG00000155330	C16orf87	chromosome 16 open reading frame 87	1.76765	1.78206
	ENSG00000118418	HMGN3	high mobility group nucleosomal binding domain 3	1.75435	1.77512
	ENSG00000114744	COMMD2	COMM domain containing 2	1.81109	1.76506
	ENSG00000102683	SGCG	sarcoglycan gamma	1.58645	1.76154
	ENSG00000247556	OIP5-AS1	OIP5 antisense RNA 1	1.90865	1.75325
	ENSG00000134258	VTCN1	V-set domain containing T cell activation inhibitor 1	1.77544	1.75288
	ENSG00000174827	PDZK1	PDZ domain containing 1	2.32078	1.75265
	ENSG00000103495	MAZ	MYC associated zinc finger protein	1.56603	1.75118
	ENSG00000204103	MAFB	MAF bZIP transcription factor B	1.68057	1.74892
	ENSG00000112149	CD83	CD83 molecule	1.92141	1.74713
	ENSG00000140600	SH3GL3	"SH3 domain containing GRB2 like 3, endophilin A3"	1.68462	1.73615

	ENSG00000165061	ZMAT4	zinc finger matrin-type 4	1.53596	1.73342
	ENSG00000123219	CENPK	centromere protein K	1.76657	1.73323
	ENSG00000186480	INSIG1	insulin induced gene 1	1.83312	1.73253
	ENSG00000133466	C1QTNF6	C1q and tumor necrosis factor related protein 6	1.65012	1.72005
	ENSG00000071909	MYO3B	myosin IIIB	1.78755	1.71407
	ENSG00000112394	SLC16A10	solute carrier family 16 member 10	1.92999	1.71366
	ENSG00000172331	BPGM	bisphosphoglycerate mutase	1.74992	1.71232
	ENSG00000216490	IFI30	"IFI30, lysosomal thiol reductase"	1.18563	1.69327
	ENSG00000163444	TMEM183A	transmembrane protein 183A	1.61754	1.69108
	ENSG00000143633	C1orf131	chromosome 1 open reading frame 131	1.49237	1.68842
	ENSG00000148053	NTRK2	neurotrophic receptor tyrosine kinase 2	2.31639	1.68192
	ENSG00000088305	DNMT3B	DNA methyltransferase 3 beta	1.75426	1.6786
	ENSG00000115380	EFEMP1	EGF containing fibulin like extracellular matrix protein 1	1.53166	1.67697
	ENSG00000126709	IFI6	interferon alpha inducible protein 6	0.854428	1.67039
	ENSG00000188643	S100A16	S100 calcium binding protein A16	1.68258	1.66285
	ENSG00000141627	DYM	dymeclin	1.72405	1.65591
	ENSG00000235123	DSCAM-AS1	DSCAM antisense RNA 1	1.79876	1.65099
	ENSG00000066926	FECH	ferrochelatase	1.68044	1.65016
	ENSG00000188613	NANOS1	nanos C2HC-type zinc finger 1	1.56703	1.64991
	ENSG00000164619	BMPER	BMP binding endothelial regulator	1.90746	1.64764
	ENSG00000184515	BEX5	brain expressed X-linked 5	1.2185	1.64046
	ENSG00000162704	ARPC5	actin related protein 2/3 complex subunit 5	1.505	1.64043
	ENSG00000197696	NMB	neuromedin B	1.57817	1.63764
	ENSG00000113583	C5orf15	chromosome 5 open reading frame 15	1.65814	1.63619
	ENSG00000177971	IMP3	"IMP3, U3 small nucleolar ribonucleoprotein"	1.2763	1.63331
	ENSG00000107833	NPM3	nucleophosmin/nucleoplasmin 3	1.59262	1.6237
	ENSG00000168913	ENHO	energy homeostasis associated	1.61978	1.62355
	ENSG00000108423	TUBD1	tubulin delta 1	1.66967	1.62302
	ENSG00000124333	VAMP7	vesicle associated membrane protein 7	1.74104	1.60978
	ENSG00000162599	NFIA	nuclear factor I A	1.93518	1.60973
	ENSG00000186470	BTN3A2	butyrophilin subfamily 3 member A2	1.66846	1.60858
	ENSG00000123570	RAB9B	"RAB9B, member RAS oncogene family"	1.39674	1.60651
	ENSG00000198797	BRINP2	BMP/retinoic acid inducible neural specific 2	2.03546	1.60345
	ENSG00000141696	P3H4	prolyl 3-hydroxylase family member 4 (non-enzymatic)	1.43784	1.60295
	ENSG00000117148	ACTL8	actin like 8	1.37881	1.60098
	ENSG00000149547	EI24	"EI24, autophagy associated transmembrane protein"	1.58026	1.59393
	ENSG00000149596	JPH2	junctophilin 2	1.83951	1.59197
	ENSG00000223658	C1GALT1C1L	C1GALT1-specific chaperone 1 like	1.17377	1.59184
	ENSG00000165996	HACD1	3-hydroxyacyl-CoA dehydratase 1	1.31717	1.5829
	ENSG00000152766	ANKRD22	ankyrin repeat domain 22	1.83286	1.5803
	ENSG00000180329	CCDC43	coiled-coil domain containing 43	1.47159	1.5781
	ENSG00000246223	LINC01550	long intergenic non-protein coding RNA 1550	1.32543	1.57637
	ENSG00000100362	PVALB	parvalbumin	1.21037	1.57428
	ENSG00000144401	METTL21A	methyltransferase like 21A	1.81833	1.57057
	ENSG00000157593	SLC35B2	solute carrier family 35 member B2	1.49914	1.5692
	ENSG00000113838	TBCCD1	TBCC domain containing 1	1.68387	1.5637
	ENSG00000152284	TCF7L1	transcription factor 7 like 1	1.48257	1.55383
	ENSG00000122035	RASL11A	RAS like family 11 member A	1.18544	1.55333
	ENSG00000236603	RANP1	"RAN, member RAS oncogene family pseudogene 1"	0.818454	1.5527
	ENSG00000136982	DSCC1	DNA replication and sister chromatid cohesion 1	1.65166	1.54324
	ENSG00000134748	PRPF38A	pre-mRNA processing factor 38A	1.47559	1.5399
	ENSG00000117122	MFAP2	microfibrillar associated protein 2	1.55689	1.53872
	ENSG00000162642	C1orf52	chromosome 1 open reading frame 52	1.30657	1.53613
	ENSG00000115252	PDE1A	phosphodiesterase 1A	1.68231	1.53396
	ENSG00000107562	CXCL12	C-X-C motif chemokine ligand 12	1.71022	1.52676
	ENSG00000087111	PIGS	phosphatidylinositol glycan anchor biosynthesis class S	1.71025	1.52542
	ENSG00000164932	CTHRC1	collagen triple helix repeat containing 1	1.78111	1.52537
	ENSG00000103942	HOMER2	homer scaffolding protein 2	1.61365	1.51969
	ENSG00000157502	MUM1L1	MUM1 like 1	1.46894	1.51818
	ENSG00000154274	C4orf19	chromosome 4 open reading frame 19	1.57704	1.5167
	ENSG00000171503	ETFDH	electron transfer flavoprotein dehydrogenase	1.6454	1.51574
	ENSG00000104679	R3HCC1	R3H domain and coiled-coil containing 1	1.47594	1.51118

	ENSG00000181274	FRAT2	frequently rearranged in advanced T-cell lymphomas 2	1.26584	1.50653
	ENSG00000148229	POLE3	"DNA polymerase epsilon 3, accessory subunit"	1.31072	1.50374
	ENSG00000030110	BAK1	BCL2 antagonist/killer 1	1.22959	1.49069
	ENSG00000127124	HIVEP3	human immunodeficiency virus type I enhancer binding protein 3	1.71264	1.48377
	ENSG00000141540	TTYH2	tweety family member 2	1.39606	1.47698
	ENSG00000196839	ADA	adenosine deaminase	1.37348	1.47158
	ENSG00000149600	COMMD7	COMM domain containing 7	1.39645	1.46911
	ENSG00000185745	IFIT1	interferon induced protein with tetratricopeptide repeats 1	0.683847	1.46504
	ENSG00000171119	NRTN	neurturin	1.42941	1.46177
	ENSG00000170775	GPR37	G protein-coupled receptor 37	1.6004	1.4605
	ENSG00000168874	ATOH8	atonal bHLH transcription factor 8	1.53391	1.45938
	ENSG00000180834	MAP6D1	MAP6 domain containing 1	1.64809	1.45758
	ENSG00000168350	DEGS2	"delta 4-desaturase, sphingolipid 2"	1.16509	1.45397
	ENSG00000156299	TIAM1	T-cell lymphoma invasion and metastasis 1	1.78994	1.45061
	ENSG00000157600	TMEM164	transmembrane protein 164	1.45174	1.44885
	ENSG00000135218	CD36	CD36 molecule	1.5895	1.44303
	ENSG00000204950	LRRC10B	leucine rich repeat containing 10B	1.33852	1.43715
	ENSG00000004864	SLC25A13	solute carrier family 25 member 13	1.29903	1.43565
	ENSG00000240849	TMEM189	transmembrane protein 189	1.4711	1.43429
	ENSG00000135114	OASL	2'-5'-oligoadenylate synthetase like	0.68925	1.43106
	ENSG00000186184	POLR1D	RNA polymerase I subunit D	1.21672	1.4292
	ENSG00000130024	PHF10	PHD finger protein 10	1.47714	1.42481
	ENSG00000186832	KRT16	keratin 16	1.41614	1.42172
	ENSG00000135374	ELF5	E74 like ETS transcription factor 5	1.54622	1.41993
	ENSG00000214063	TSPAN4	tetraspanin 4	1.20445	1.41946
	ENSG00000157303	SUSD3	sushi domain containing 3	1.38012	1.41753
	ENSG00000060138	YBX3	Y-box binding protein 3	1.38701	1.41547
	ENSG00000110330	BIRC2	baculoviral IAP repeat containing 2	1.50289	1.41292
	ENSG00000175928	LRRN1	leucine rich repeat neuronal 1	1.58509	1.41155
	ENSG00000076604	TRAF4	TNF receptor associated factor 4	1.40716	1.41039
	ENSG00000168569	TMEM223	transmembrane protein 223	1.13466	1.40979
	ENSG00000159763	PIP	prolactin induced protein	1.58833	1.40631
	ENSG00000166803	KIAA0101	KIAA0101	1.25341	1.39906
	ENSG00000212747	FAM127C	family with sequence similarity 127 member C	1.25769	1.39421
	ENSG00000162639	HENMT1	HEN1 methyltransferase homolog 1	1.27558	1.38603
	ENSG00000009844	VTA1	vesicle trafficking 1	1.32726	1.38368
	ENSG00000168246	UBTD2	ubiquitin domain containing 2	1.45232	1.38079
	ENSG00000139173	TMEM117	transmembrane protein 117	1.22016	1.36977
	ENSG00000149043	SYT8	synaptotagmin 8	1.65318	1.36842
	ENSG00000132383	RPA1	replication protein A1	1.3934	1.36769
	ENSG00000042753	AP2S1	adaptor related protein complex 2 sigma 1 subunit	1.22989	1.36345
	ENSG00000117411	B4GALT2	"beta-1,4-galactosyltransferase 2"	1.18107	1.36107
	ENSG00000187601	MAGEH1	MAGE family member H1	1.16952	1.36045
	ENSG00000141985	SH3GL1	"SH3 domain containing GRB2 like 1, endophilin A2"	1.20402	1.35972
	ENSG00000175730	BAK1P1	BCL2 antagonist/killer 1 pseudogene 1	1.33959	1.35957
	ENSG00000128309	MPST	mercaptopyruvate sulfurtransferase	0.954081	1.3583
	ENSG00000156113	KCNMA1	potassium calcium-activated channel subfamily M alpha 1	1.55427	1.35738
	ENSG00000170819	BFSP2	beaded filament structural protein 2	1.60905	1.3469
	ENSG00000172663	TMEM134	transmembrane protein 134	0.907853	1.34654
	ENSG00000165629	ATP5C1	"ATP synthase, H+ transporting, mitochondrial F1 complex, gamma polypeptide 1"	1.43753	1.34628
	ENSG00000145425	RPS3A	ribosomal protein S3A	1.35179	1.34338
	ENSG00000213753	CENPBD1P1	CENPB DNA-binding domains containing 1 pseudogene 1	1.15072	1.34084
	ENSG00000134986	NREP	neuronal regeneration related protein	1.5593	1.33906
	ENSG00000137959	IFI44L	interferon induced protein 44 like	0.595391	1.33428
	ENSG00000138336	TET1	tet methylcytosine dioxygenase 1	1.61365	1.33383
	ENSG00000176396	EID2	EP300 interacting inhibitor of differentiation 2	1.02888	1.32895
	ENSG00000162441	LZIC	leucine zipper and CTNNBIP1 domain containing	1.51983	1.32526
	ENSG00000123562	MORF4L2	mortality factor 4 like 2	1.31419	1.32475
	ENSG00000171612	SLC25A33	solute carrier family 25 member 33	1.28595	1.32461
	ENSG00000196620	UGT2B15	UDP glucuronosyltransferase family 2 member B15	1.18061	1.32403

	ENSG00000109321	AREG	amphiregulin	1.42032	1.32116
	ENSG00000174599	TRAM1L1	translocation associated membrane protein 1-like 1	1.03518	1.32007
	ENSG00000144677	CTDSPL	CTD small phosphatase like	1.29415	1.31864
	ENSG00000239900	ADSL	adenylosuccinate lyase	1.28924	1.31677
	ENSG00000175130	MARCKSL1	MARCKS like 1	1.18066	1.31506
	ENSG00000180992	MRPL14	mitochondrial ribosomal protein L14	1.09525	1.31287
	ENSG00000113648	H2AFY	H2A histone family member Y	1.35164	1.31266
	ENSG00000255302	EID1	EP300 interacting inhibitor of differentiation 1	1.13643	1.31191
	ENSG00000188681	TEKT4P2	tektin 4 pseudogene 2	1.25626	1.31105
	ENSG00000180573	HIST1H2AC	histone cluster 1 H2A family member c	1.2603	1.30951
	ENSG00000083807	SLC27A5	solute carrier family 27 member 5	1.0881	1.3092
	ENSG00000183688	RFLNB	refilin B	1.58933	1.30077
	ENSG00000253719	ATXN7L3B	ataxin 7 like 3B	1.23747	1.29717
	ENSG00000205643	CDPF1	cysteine rich DPF motif domain containing 1	0.989699	1.29684
	ENSG00000145808	ADAMTS19	ADAM metallopeptidase with thrombospondin type 1 motif 19	1.47001	1.29029
	ENSG00000131238	PPT1	palmitoyl-protein thioesterase 1	1.33288	1.28796
	ENSG00000037280	FLT4	fms related tyrosine kinase 4	0.967415	1.2875
	ENSG00000105549	THEG	theg spermatid protein	1.36115	1.28725
	ENSG00000184678	HIST2H2BE	histone cluster 2 H2B family member e	1.19281	1.28682
	ENSG00000163156	TNFAIP8L2-SCNM1	TNFAIP8L2-SCNM1 readthrough	1.15396	1.28294
	ENSG00000172201	ID4	"inhibitor of DNA binding 4, HLH protein"	1.1042	1.28042
	ENSG00000136261	BZW2	basic leucine zipper and W2 domains 2	1.33163	1.28027
	ENSG00000171476	HOPX	HOP homeobox	1.87771	1.27803
	ENSG00000163006	CCDC138	coiled-coil domain containing 138	1.54328	1.27542
	ENSG00000256073	URB1-AS1	URB1 antisense RNA 1 (head to head)	0.768621	1.27432
	ENSG00000185885	IFITM1	interferon induced transmembrane protein 1	0.866949	1.27411
	ENSG00000149646	CNBD2	cyclic nucleotide binding domain containing 2	0.837055	1.27329
	ENSG00000197763	TXNRD3NB	thioredoxin reductase 3 neighbor	1.05308	1.27236
	ENSG00000160183	TMPRSS3	"transmembrane protease, serine 3"	1.50993	1.27099
	ENSG00000113761	ZNF346	zinc finger protein 346	1.50192	1.26861
	ENSG00000100211	CBY1	"chibby family member 1, beta catenin antagonist"	1.33359	1.26603
	ENSG00000237984	PTENP1	phosphatase and tensin homolog pseudogene 1	1.06433	1.26423
	ENSG00000198054	DSCR8	Down syndrome critical region 8	1.86764	1.26283
	ENSG00000168303	MPLKIP	M-phase specific PLK1 interacting protein	0.939126	1.25982
	ENSG00000136295	TTYH3	tweety family member 3	1.29448	1.25873
	ENSG00000172428	COPS9	COP9 signalosome subunit 9	1.02218	1.25621
	ENSG00000137364	TPMT	thiopurine S-methyltransferase	1.40831	1.2533
	ENSG00000196739	COL27A1	collagen type XXVII alpha 1 chain	1.67382	1.25171
	ENSG00000165724	ZMYND19	zinc finger MYND-type containing 19	1.05418	1.24949
	ENSG00000167930	FAM234A	family with sequence similarity 234 member A	1.20283	1.24764
	ENSG00000111181	SLC6A12	solute carrier family 6 member 12	1.42094	1.24514
	ENSG00000176401	EID2B	EP300 interacting inhibitor of differentiation 2B	0.663137	1.24476
	ENSG00000168405	CMAHP	"cytidine monophospho-N-acetylneuraminic acid hydroxylase, pseudogene"	0.825125	1.24412
	ENSG00000139970	RTN1	reticulon 1	1.33667	1.24382
	ENSG00000186162	CIDECP	cell death-inducing DFFA-like effector c pseudogene	1.01528	1.24263
	ENSG00000176022	B3GALT6	"beta-1,3-galactosyltransferase 6"	0.891879	1.24243
	ENSG00000121022	COPS5	COP9 signalosome subunit 5	1.20623	1.24081
	ENSG00000164172	MOCS2	molybdenum cofactor synthesis 2	1.286	1.24044
	ENSG00000166450	PRTG	protogenin	1.31376	1.23996
	ENSG00000160180	TFF3	trefoil factor 3	1.06078	1.23842
	ENSG00000115657	ABCB6	ATP binding cassette subfamily B member 6 (Langereis blood group)	1.20686	1.2379
	ENSG00000134809	TIMM10	translocase of inner mitochondrial membrane 10 homolog (yeast)	1.08019	1.23627
	ENSG00000155380	SLC16A1	solute carrier family 16 member 1	1.27052	1.23591
	ENSG00000205363	C15orf59	chromosome 15 open reading frame 59	1.07989	1.23498
	ENSG00000213741	RPS29	ribosomal protein S29	1.07896	1.23262
	ENSG00000134758	RNF138	ring finger protein 138	0.993903	1.2314
	ENSG00000064835	POU1F1	POU class 1 homeobox 1	1.10366	1.22851
	ENSG00000184232	OAF	out at first homolog	0.614108	1.2268
	ENSG00000164111	ANXA5	annexin A5	1.17391	1.22543
	ENSG00000160588	MPZL3	myelin protein zero like 3	1.30983	1.22454
	ENSG00000166794	PPIB	peptidylprolyl isomerase B	1.11594	1.2242

	ENSG00000262904	TMPOP2	thymopoietin pseudogene 2	0.815511	1.2229
	ENSG00000165512	ZNF22	zinc finger protein 22	1.10956	1.22216
	ENSG00000101098	RIMS4	regulating synaptic membrane exocytosis 4	1.25577	1.2212
	ENSG00000150893	FREM2	FRAS1 related extracellular matrix protein 2	1.6484	1.22025
	ENSG00000137558	PI15	peptidase inhibitor 15	1.38949	1.21943
	ENSG00000111653	ING4	inhibitor of growth family member 4	1.19538	1.21837
	ENSG00000185787	MORF4L1	mortality factor 4 like 1	1.23656	1.21828
	ENSG00000224287	MSL3P1	male-specific lethal 3 homolog (Drosophila) pseudogene 1	1.08888	1.21766
	ENSG00000250722	SEPP1	"selenoprotein P, plasma, 1"	1.09129	1.21632
	ENSG00000148300	REXO4	"REX4 homolog, 3'-5' exonuclease"	0.991587	1.21563
	ENSG00000172867	KRT2	keratin 2	1.2364	1.21345
	ENSG00000204228	HSD17B8	hydroxysteroid 17-beta dehydrogenase 8	1.0486	1.21188
	ENSG00000100814	CCNB1IP1	cyclin B1 interacting protein 1	1.22499	1.20591
	ENSG00000171631	P2RY6	pyrimidinergic receptor P2Y6	1.07541	1.20488
	ENSG00000196636	SDHAF3	succinate dehydrogenase complex assembly factor 3	1.03412	1.20257
	ENSG00000102172	SMS	spermine synthase	1.1773	1.20089
	ENSG00000154553	PDLIM3	PDZ and LIM domain 3	1.21686	1.19913
	ENSG00000102743	SLC25A15	solute carrier family 25 member 15	1.27744	1.19857
	ENSG00000122068	FYTTD1	forty-two-three domain containing 1	1.3472	1.19733
	ENSG00000167767	KRT80	keratin 80	1.24017	1.19701
	ENSG00000091592	NLRP1	NLR family pyrin domain containing 1	1.09248	1.19686
	ENSG00000102543	CDADC1	cytidine and dCMP deaminase domain containing 1	0.972441	1.19253
	ENSG00000185475	TMEM179B	transmembrane protein 179B	1.13071	1.1919
	ENSG00000166073	GPR176	G protein-coupled receptor 176	0.911931	1.19186
	ENSG00000186603	HPDL	4-hydroxyphenylpyruvate dioxygenase like	0.801218	1.19059
	ENSG00000104980	TIMM44	translocase of inner mitochondrial membrane 44	0.990688	1.19053
	ENSG00000178896	EXOSC4	exosome component 4	0.624955	1.19043
	ENSG00000104341	LAPTM4B	lysosomal protein transmembrane 4 beta	1.26545	1.18734
	ENSG00000155329	ZCCHC10	zinc finger CCHC-type containing 10	0.96443	1.18733
	ENSG00000164291	ARSK	arylsulfatase family member K	1.09553	1.18466
	ENSG00000040275	SPDL1	spindle apparatus coiled-coil protein 1	1.36245	1.18421
	ENSG00000041357	PSMA4	proteasome subunit alpha 4	1.20228	1.18312
	ENSG00000184486	POU3F2	POU class 3 homeobox 2	1.23518	1.18252
	ENSG00000010319	SEMA3G	semaphorin 3G	1.08938	1.18135
	ENSG00000131373	HACL1	2-hydroxyacyl-CoA lyase 1	1.08995	1.17999
	ENSG00000185504	FAAP100	Fanconi anemia core complex associated protein 100	0.924334	1.1769
	ENSG00000244187	TMEM141	transmembrane protein 141	1.08181	1.17586
	ENSG00000171984	C20orf196	chromosome 20 open reading frame 196	0.846608	1.17566
	ENSG00000120697	ALG5	"ALG5, dolichyl-phosphate beta-glucosyltransferase"	0.974665	1.17464
	ENSG00000110871	COQ5	"coenzyme Q5, methyltransferase"	1.12072	1.17251
	ENSG00000180611	MB21D2	Mab-21 domain containing 2	1.01576	1.17096
	ENSG00000101255	TRIB3	tribbles pseudokinase 3	1.04783	1.16733
	ENSG00000131475	VPS25	vacuolar protein sorting 25 homolog	1.09281	1.16585
	ENSG00000169908	TM4SF1	transmembrane 4 L six family member 1	0.892293	1.16473
	ENSG00000134864	GGACT	gamma-glutamylamine cyclotransferase	0.730741	1.16279
	ENSG00000101546	RBFA	ribosome binding factor A (putative)	1.08745	1.15657
	ENSG00000128294	TPST2	tyrosylprotein sulfotransferase 2	1.27295	1.15574
	ENSG00000186767	SPIN4	spindlin family member 4	1.20897	1.15524
	ENSG00000169218	RSPO1	R-spondin 1	1.00213	1.14493
	ENSG00000160446	ZDHHC12	zinc finger DHHC-type containing 12	0.720658	1.1442
	ENSG00000188763	FZD9	frizzled class receptor 9	0.881945	1.14293
	ENSG00000066697	MSANTD3	Myb/SANT DNA binding domain containing 3	1.03394	1.14267
	ENSG00000168785	TSPAN5	tetraspanin 5	1.45388	1.14094
	ENSG00000182575	NXPH3	neurexophilin 3	1.14279	1.13923
	ENSG00000144867	SRPRB	SRP receptor beta subunit	1.135	1.13818
	ENSG00000162891	IL20	interleukin 20	1.37049	1.13747
	ENSG00000164128	NPY1R	neuropeptide Y receptor Y1	1.33529	1.13624
	ENSG00000169976	SF3B5	splicing factor 3b subunit 5	0.996388	1.13546
	ENSG00000150687	PRSS23	"protease, serine 23"	1.31424	1.13168
	ENSG00000173141	MRPL57	mitochondrial ribosomal protein L57	0.811779	1.13103
	ENSG00000110442	COMMD9	COMM domain containing 9	1.13099	1.13093
	ENSG00000101850	GPR143	G protein-coupled receptor 143	0.864325	1.13005

	ENSG00000182117	NOP10	NOP10 ribonucleoprotein	1.02759	1.12941
	ENSG00000077274	CAPN6	calpain 6	1.02668	1.12836
	ENSG00000188820	FAM26F	family with sequence similarity 26 member F	0.969095	1.12822
	ENSG00000124813	RUNX2	runt related transcription factor 2	1.40906	1.12666
	ENSG00000142619	PADI3	peptidyl arginine deiminase 3	1.29671	1.12462
	ENSG00000196741	LINC01560	long intergenic non-protein coding RNA 1560	0.9892	1.12458
	ENSG00000103257	SLC7A5	solute carrier family 7 member 5	1.31711	1.12174
	ENSG00000110435	PDHX	pyruvate dehydrogenase complex component X	1.11496	1.1193
	ENSG00000121753	ADGRB2	adhesion G protein-coupled receptor B2	0.874388	1.11731
	ENSG00000065268	WDR18	WD repeat domain 18	0.938405	1.11267
	ENSG00000168002	POLR2G	RNA polymerase II subunit G	1.0195	1.11076
	ENSG00000148824	MTG1	mitochondrial ribosome associated GTPase 1	0.930824	1.10945
	ENSG00000143013	LMO4	LIM domain only 4	1.21488	1.10918
	ENSG00000243646	IL10RB	interleukin 10 receptor subunit beta	1.09275	1.10894
	ENSG00000184588	PDE4B	phosphodiesterase 4B	1.30251	1.10839
	ENSG00000103415	HMOX2	heme oxygenase 2	0.981092	1.10676
	ENSG00000165389	SPTSSA	serine palmitoyltransferase small subunit A	1.13176	1.10577
	ENSG00000139144	PIK3C2G	phosphatidylinositol-4-phosphate 3-kinase catalytic subunit type 2 gamma	1.32998	1.10551
	ENSG00000145545	SRD5A1	steroid 5 alpha-reductase 1	0.903325	1.10497
	ENSG00000204344	STK19	serine/threonine kinase 19	1.04042	1.10347
	ENSG00000162384	C1orf123	chromosome 1 open reading frame 123	0.966238	1.10235
	ENSG00000103855	CD276	CD276 molecule	1.24127	1.1014
	ENSG00000035687	ADSS	adenylosuccinate synthase	0.989145	1.09993
	ENSG00000185090	MANEAL	mannosidase endo-alpha like	1.09	1.09727
	ENSG00000087842	PIR	pirin	1.18405	1.09699
	ENSG00000169372	CRADD	CASP2 and RIPK1 domain containing adaptor with death domain	0.94901	1.09059
	ENSG00000133627	ACTR3B	ARP3 actin related protein 3 homolog B	1.03853	1.08886
	ENSG00000137992	DBT	dihydrolipoamide branched chain transacylase E2	1.19273	1.08744
	ENSG00000109452	INPP4B	inositol polyphosphate-4-phosphatase type II B	1.41143	1.08739
	ENSG00000108010	GLRX3	glutaredoxin 3	0.917546	1.0863
	ENSG00000169884	WNT10B	Wnt family member 10B	0.553471	1.08361
	ENSG00000122873	CISD1	CDGSH iron sulfur domain 1	1.01027	1.0821
	ENSG00000230844	ZNF674-AS1	ZNF674 antisense RNA 1 (head to head)	0.734379	1.08159
	ENSG00000246705	H2AFJ	H2A histone family member J	0.688296	1.08134
	ENSG00000176108	CHMP6	charged multivesicular body protein 6	0.715407	1.0806
	ENSG00000115596	WNT6	Wnt family member 6	0.563029	1.08002
	ENSG00000204576	PRR3	proline rich 3	1.05653	1.07873
	ENSG00000184220	CMSS1	cms1 ribosomal small subunit homolog (yeast)	1.11261	1.07853
	ENSG00000040731	CDH10	cadherin 10	1.37135	1.077
	ENSG00000196776	CD47	CD47 molecule	1.08686	1.07416
	ENSG00000188042	ARL4C	ADP ribosylation factor like GTPase 4C	1.11503	1.06863
	ENSG00000166123	GPT2	glutamic--pyruvic transaminase 2	1.20088	1.06805
	ENSG00000119326	CTNNAL1	catenin alpha like 1	1.10965	1.06564
	ENSG00000138670	RASGEF1B	RasGEF domain family member 1B	0.863543	1.06333
	ENSG00000168495	POLR3D	RNA polymerase III subunit D	0.922376	1.05985
	ENSG00000175137	SH3BP5L	SH3 binding domain protein 5 like	1.04061	1.05831
	ENSG00000094804	CDC6	cell division cycle 6	0.995412	1.05737
	ENSG00000171428	NAT1	N-acetyltransferase 1	0.828996	1.0541
	ENSG00000167552	TUBA1A	tubulin alpha 1a	1.22901	1.05345
	ENSG00000143845	ETNK2	ethanolamine kinase 2	1.11182	1.05306
	ENSG00000174109	C16orf91	chromosome 16 open reading frame 91	0.529916	1.05276
	ENSG00000135828	RNASEL	ribonuclease L	1.11074	1.05212
	ENSG00000110987	BCL7A	BCL tumor suppressor 7A	0.971165	1.052
	ENSG00000185615	PDIA2	protein disulfide isomerase family A member 2	0.84232	1.0513
	ENSG00000171135	JAGN1	jagunal homolog 1	0.901859	1.05076
	ENSG00000107968	MAP3K8	mitogen-activated protein kinase kinase kinase 8	0.80049	1.05051
	ENSG00000173894	CBX2	chromobox 2	1.05339	1.05031
	ENSG00000160182	TFF1	trefoil factor 1	1.12383	1.05027
	ENSG00000158715	SLC45A3	solute carrier family 45 member 3	0.928566	1.04996
	ENSG00000143162	CREG1	cellular repressor of E1A stimulated genes 1	1.06377	1.04923
	ENSG00000117461	PIK3R3	phosphoinositide-3-kinase regulatory subunit 3	1.14694	1.04752
	ENSG00000185015	CA13	carbonic anhydrase 13	1.36253	1.04568
	ENSG00000158769	F11R	F11 receptor	1.09778	1.04492
	ENSG00000167695	FAM57A	family with sequence similarity 57 member A	1.08004	1.04424

	ENSG00000145882	PCYOX1L	prenylcysteine oxidase 1 like	1.0197	1.04292
	ENSG00000133142	TCEAL4	transcription elongation factor A like 4	0.865075	1.04156
	ENSG00000163053	SLC16A14	solute carrier family 16 member 14	0.890857	1.04069
	ENSG00000124006	OBSL1	obscurin like 1	0.990759	1.04061
	ENSG00000151917	BEND6	BEN domain containing 6	1.01679	1.03956
	ENSG00000169682	SPNS1	sphingolipid transporter 1 (putative)	0.993692	1.03893
	ENSG00000131043	AAR2	AAR2 splicing factor homolog	0.927601	1.03828
	ENSG00000177614	PGBD5	piggyBac transposable element derived 5	0.874885	1.03782
	ENSG00000165732	DDX21	DEAD-box helicase 21	1.0968	1.03752
	ENSG00000172171	TEFM	"transcription elongation factor, mitochondrial"	0.886352	1.03729
	ENSG00000117479	SLC19A2	solute carrier family 19 member 2	0.994844	1.03721
	ENSG00000270170	NCBP2-AS2	NCBP2 antisense RNA 2 (head to head)	0.585973	1.03664
	ENSG00000197894	ADH5	"alcohol dehydrogenase 5 (class III), chi polypeptide"	1.00464	1.03491
	ENSG00000187231	SESTD1	SEC14 and spectrin domain containing 1	1.24413	1.03235
	ENSG00000135148	TRAFD1	TRAF-type zinc finger domain containing 1	1.11782	1.02991
	ENSG00000153485	TMEM251	transmembrane protein 251	0.873496	1.02926
	ENSG00000113356	POLR3G	RNA polymerase III subunit G	0.810646	1.02777
	ENSG00000136527	TRA2B	transformer 2 beta homolog (Drosophila)	0.93612	1.02773
	ENSG00000177383	MAGEF1	MAGE family member F1	0.775277	1.0272
	ENSG00000158042	MRPL17	mitochondrial ribosomal protein L17	0.790089	1.02674
	ENSG00000113209	PCDHB5	protocadherin beta 5	1.21352	1.02547
	ENSG00000168101	NUDT16L1	nudix hydrolase 16 like 1	0.742033	1.02527
	ENSG00000158050	DUSP2	dual specificity phosphatase 2	0.861033	1.02365
	ENSG00000167272	POP5	"POP5 homolog, ribonuclease P/MRP subunit"	0.785138	1.02197
	ENSG00000043039	BARX2	BARX homeobox 2	1.10383	1.02157
	ENSG00000131015	ULBP2	UL16 binding protein 2	1.14969	1.02138
	ENSG00000167747	C19orf48	chromosome 19 open reading frame 48	0.550821	1.02046
	ENSG00000140280	LYSMD2	LysM domain containing 2	0.879903	1.02031
	ENSG00000143179	UCK2	uridine-cytidine kinase 2	0.9374	1.01923
	ENSG00000175768	TOMM5	translocase of outer mitochondrial membrane 5	0.924343	1.01822
	ENSG00000151611	MMAA	methylmalonic aciduria (cobalamin deficiency) cblA type	0.736563	1.01764
	ENSG00000116761	CTH	cystathionine gamma-lyase	1.37027	1.01686
	ENSG00000181634	TNFSF15	tumor necrosis factor superfamily member 15	0.836726	1.01551
	ENSG00000221926	TRIM16	tripartite motif containing 16	0.993213	1.01247
	ENSG00000154429	CCSAP	"centriole, cilia and spindle associated protein"	1.0959	1.01218
	ENSG00000163041	H3F3A	H3 histone family member 3A	0.858372	1.01209
	ENSG00000267368	UPK3BL	uroplakin 3B-like	0.806669	1.01034
	ENSG00000163931	TKT	transketolase	0.929334	1.00705
	ENSG00000132429	POPDC3	popeye domain containing 3	0.787233	1.0069
	ENSG00000178096	BOLA1	bolA family member 1	0.570531	1.00556
	ENSG00000151575	TEX9	testis expressed 9	0.870901	1.00428
	ENSG00000174013	FBXO45	F-box protein 45	1.04396	1.00334
	ENSG00000139211	AMIGO2	adhesion molecule with Ig like domain 2	1.01909	1.00302
	ENSG00000159640	ACE	angiotensin I converting enzyme	1.21031	1.00259
	ENSG00000154518	ATP5G3	"ATP synthase, H+ transporting, mitochondrial Fo complex subunit C3 (subunit 9)"	0.885476	1.00146
	ENSG00000008083	JARID2	jumonji and AT-rich interaction domain containing 2	1.09834	0.998283
	ENSG00000156374	PCGF6	polycomb group ring finger 6	0.935854	0.998118
	ENSG00000167780	SOAT2	sterol O-acyltransferase 2	0.946383	0.996519
	ENSG00000092841	MYL6	myosin light chain 6	0.876249	0.994649
	ENSG00000113924	HGD	"homogentisate 1,2-dioxygenase"	1.31709	0.994539
	ENSG00000232388	LINC00493	long intergenic non-protein coding RNA 493	0.794676	0.993629
	ENSG00000198055	GRK6	G protein-coupled receptor kinase 6	0.718213	0.990066
	ENSG00000136143	SUCLA2	succinate-CoA ligase ADP-forming beta subunit	1.17971	0.989095
	ENSG00000134240	HMGCS2	3-hydroxy-3-methylglutaryl-CoA synthase 2	1.89403	0.988791
	ENSG00000023902	PLEKHO1	pleckstrin homology domain containing O1	0.955779	0.986967
	ENSG00000143061	IGSF3	immunoglobulin superfamily member 3	1.06424	0.986889
	ENSG00000239672	NME1	NME/NM23 nucleoside diphosphate kinase 1	0.856472	0.982628
	ENSG00000130332	LSM7	"LSM7 homolog, U6 small nuclear RNA and mRNA degradation associated"	0.610844	0.982496
	ENSG00000171863	RPS7	ribosomal protein S7	0.976177	0.982406
	ENSG00000112320	SOBP	sine oculis binding protein homolog	1.12312	0.97963
	ENSG00000133874	RNF122	ring finger protein 122	1.14128	0.979431
	ENSG00000030066	NUP160	nucleoporin 160	1.02944	0.977176

	ENSG00000174749	C4orf32	chromosome 4 open reading frame 32	0.895463	0.976444
	ENSG00000163710	PCOLCE2	procollagen C-endopeptidase enhancer 2	0.623997	0.976315
	ENSG00000138166	DUSP5	dual specificity phosphatase 5	1.16304	0.974547
	ENSG00000007392	LUC7L	LUC7 like	0.729032	0.973398
	ENSG00000147533	GOLGA7	golgin A7	1.14222	0.971833
	ENSG00000108826	MRPL27	mitochondrial ribosomal protein L27	0.756929	0.97082
	ENSG00000125977	EIF2S2	eukaryotic translation initiation factor 2 subunit beta	0.929427	0.970642
	ENSG00000188636	LDOC1L	leucine zipper down-regulated in cancer 1 like	1.00147	0.970258
	ENSG00000189007	ADAT2	"adenosine deaminase, tRNA specific 2"	0.875681	0.969717
	ENSG00000143947	RPS27A	ribosomal protein S27a	0.982195	0.969381
	ENSG00000023697	DERA	deoxyribose-phosphate aldolase	0.991452	0.967232
	ENSG00000168291	PDHB	pyruvate dehydrogenase (lipoamide) beta	1.02254	0.966478
	ENSG00000267100	ILF3-AS1	ILF3 antisense RNA 1 (head to head)	0.605486	0.965656
	ENSG00000127337	YEATS4	YEATS domain containing 4	0.743376	0.965598
	ENSG00000162244	RPL29	ribosomal protein L29	0.678791	0.965449
	ENSG00000163584	RPL22L1	ribosomal protein L22 like 1	0.864808	0.965201
	ENSG00000100395	L3MBTL2	L3MBTL2 polycomb repressive complex 1 subunit	0.91279	0.965081
	ENSG00000169826	CSGALNACT2	chondroitin sulfate N-acetylgalactosaminyltransferase 2	1.09471	0.964817
	ENSG00000163933	RFT1	RFT1 homolog	1.00573	0.963011
	ENSG00000006459	KDM7A	lysine demethylase 7A	1.0876	0.962187
	ENSG00000073536	NLE1	notchless homolog 1	0.739256	0.961983
	ENSG00000145604	SKP2	"S-phase kinase-associated protein 2, E3 ubiquitin protein ligase"	0.987079	0.961049
	ENSG00000156103	MMP16	matrix metallopeptidase 16	1.33017	0.960805
	ENSG00000131849	ZNF132	zinc finger protein 132	0.974712	0.960395
	ENSG00000079257	LXN	latexin	0.957785	0.959065
	ENSG00000183648	NDUFB1	NADH:ubiquinone oxidoreductase subunit B1	0.794603	0.958529
	ENSG00000107020	PLGRKT	plasminogen receptor with a C-terminal lysine	1.03486	0.956894
	ENSG00000111361	EIF2B1	eukaryotic translation initiation factor 2B subunit alpha	0.842259	0.955928
	ENSG00000135250	SRPK2	SRSF protein kinase 2	0.962142	0.955164
	ENSG00000134419	RPS15A	ribosomal protein S15a	0.97588	0.954983
	ENSG00000205542	TMSB4X	"thymosin beta 4, X-linked"	0.828414	0.954921
	ENSG00000166557	TMED3	transmembrane p24 trafficking protein 3	0.862257	0.952594
	ENSG00000170027	YWHAG	tyrosine 3-monooxygenase/tryptophan 5-monooxygenase activation protein gamma	0.868706	0.950922
	ENSG00000156398	SFXN2	sideroflexin 2	0.977583	0.949226
	ENSG00000259803	SLC22A31	solute carrier family 22 member 31	0.856728	0.948328
	ENSG00000166226	CCT2	chaperonin containing TCP1 subunit 2	0.955846	0.94792
	ENSG00000132768	DPH2	DPH2 homolog	0.873147	0.947896
	ENSG00000107938	EDRF1	erythroid differentiation regulatory factor 1	0.87891	0.947828
	ENSG00000168672	FAM84B	family with sequence similarity 84 member B	0.978182	0.9469
	ENSG00000133111	RFXAP	regulatory factor X associated protein	1.06703	0.94652
	ENSG00000142864	SERBP1	SERPINE1 mRNA binding protein 1	0.99881	0.945595
	ENSG00000183665	TRMT12	tRNA methyltransferase 12 homolog	0.867535	0.94512
	ENSG00000142632	ARHGEF19	Rho guanine nucleotide exchange factor 19	0.840479	0.944296
	ENSG00000023734	STRAP	serine/threonine kinase receptor associated protein	0.923435	0.944209
	ENSG00000111341	MGP	matrix Gla protein	0.989572	0.94395
	ENSG00000063241	ISOC2	isochorismatase domain containing 2	0.578063	0.943153
	ENSG00000214194	LINC00998	long intergenic non-protein coding RNA 998	0.915797	0.942618
	ENSG00000154639	CXADR	coxsackie virus and adenovirus receptor	1.04079	0.942615
	ENSG00000155254	MARVELD1	MARVEL domain containing 1	0.787191	0.941691
	ENSG00000143977	SNRPG	small nuclear ribonucleoprotein polypeptide G	0.722707	0.941404
	ENSG00000093009	CDC45	cell division cycle 45	0.95113	0.941068
	ENSG00000123131	PRDX4	peroxiredoxin 4	0.874995	0.937589
	ENSG00000163479	SSR2	signal sequence receptor subunit 2	0.883125	0.937574
	ENSG00000170779	CDCA4	cell division cycle associated 4	0.679391	0.937433
	ENSG00000184675	AMER1	APC membrane recruitment protein 1	0.999996	0.936081
	ENSG00000221990	EXOC3-AS1	EXOC3 antisense RNA 1	0.65992	0.935699
	ENSG00000066468	FGFR2	fibroblast growth factor receptor 2	0.947844	0.935403
	ENSG00000131873	CHSY1	chondroitin sulfate synthase 1	1.12941	0.935212
	ENSG00000171574	ZNF584	zinc finger protein 584	0.928914	0.934971
	ENSG00000136319	TTC5	tetratricopeptide repeat domain 5	0.895979	0.934807
	ENSG00000006576	PHTF2	putative homeodomain transcription factor 2	0.995553	0.932872
	ENSG00000120341	SEC16B	"SEC16 homolog B, endoplasmic reticulum export factor"	1.14986	0.932813

	ENSG00000005022	SLC25A5	solute carrier family 25 member 5	0.874204	0.931417
	ENSG00000187605	TET3	tet methylcytosine dioxygenase 3	0.964832	0.931351
	ENSG00000140450	ARRDC4	arrestin domain containing 4	0.676729	0.931238
	ENSG00000090520	DNAJB11	DnaJ heat shock protein family (Hsp40) member B11	0.904611	0.930958
	ENSG00000162377	COA7	cytochrome c oxidase assembly factor 7 (putative)	0.72858	0.930403
	ENSG00000242372	EIF6	eukaryotic translation initiation factor 6	0.77994	0.930112
	ENSG00000188010	MORN2	MORN repeat containing 2	1.01867	0.929174
	ENSG00000107014	RLN2	relaxin 2	0.992626	0.927344
	ENSG00000124588	NQO2	NAD(P)H quinone dehydrogenase 2	0.893087	0.924738
	ENSG00000100604	CHGA	chromogranin A	0.921886	0.924259
	ENSG00000131844	MCCC2	methylcrotonoyl-CoA carboxylase 2	0.928812	0.924177
	ENSG00000196449	YRDC	yrdC N6-threonylcarbamoyltransferase domain containing	0.756552	0.92334
	ENSG00000166896	ATP23	ATP23 metallopeptidase and ATP synthase assembly factor homolog	0.957749	0.922681
	ENSG00000108448	TRIM16L	tripartite motif containing 16-like	0.910647	0.922332
	ENSG00000100028	SNRPD3	small nuclear ribonucleoprotein D3 polypeptide	0.859864	0.921748
	ENSG00000152229	PSTPIP2	proline-serine-threonine phosphatase interacting protein 2	0.853152	0.921707
	ENSG00000204315	FKBPL	FK506 binding protein like	0.676845	0.921371
	ENSG00000176055	MBLAC2	metallo-beta-lactamase domain containing 2	0.798222	0.920713
	ENSG00000116221	MRPL37	mitochondrial ribosomal protein L37	0.844301	0.91952
	ENSG00000105058	FAM32A	family with sequence similarity 32 member A	0.887943	0.919503
	ENSG00000232654	FAM136BP	"family with sequence similarity 136 member B, pseudogene"	1.19572	0.919494
	ENSG00000071054	MAP4K4	mitogen-activated protein kinase kinase kinase kinase 4	1.24666	0.919106
	ENSG00000165688	PMPCA	"peptidase, mitochondrial processing alpha subunit"	0.741118	0.917951
	ENSG00000180817	PPA1	pyrophosphatase (inorganic) 1	0.889133	0.91749
	ENSG00000225470	JPX	"JPX transcript, XIST activator (non-protein coding)"	0.797853	0.917472
	ENSG00000109775	UFSP2	UFM1 specific peptidase 2	0.918514	0.915898
	ENSG00000172551	MUCL1	mucin like 1	1.29882	0.915705
	ENSG00000167118	URM1	ubiquitin related modifier 1	0.777266	0.915202
	ENSG00000131469	RPL27	ribosomal protein L27	0.818785	0.914966
	ENSG00000124370	MCEE	methylmalonyl-CoA epimerase	0.841898	0.914268
	ENSG00000172819	RARG	retinoic acid receptor gamma	0.99176	0.914053
	ENSG00000115204	MPV17	"MPV17, mitochondrial inner membrane protein"	0.799041	0.913382
	ENSG00000227345	PARG	poly(ADP-ribose) glycohydrolase	0.88996	0.913123
	ENSG00000087301	TXNDC16	thioredoxin domain containing 16	1.2185	0.912998
	ENSG00000070785	EIF2B3	eukaryotic translation initiation factor 2B subunit gamma	0.854297	0.912875
	ENSG00000205670	SMIM11A	small integral membrane protein 11A	0.844039	0.910736
	ENSG00000163466	ARPC2	actin related protein 2/3 complex subunit 2	0.827089	0.909638
	ENSG00000198133	TMEM229B	transmembrane protein 229B	0.964364	0.908993
	ENSG00000167645	YIF1B	"Yip1 interacting factor homolog B, membrane trafficking protein"	0.721536	0.907742
	ENSG00000033867	SLC4A7	solute carrier family 4 member 7	0.972962	0.90704
	ENSG00000108352	RAPGEFL1	Rap guanine nucleotide exchange factor like 1	1.07791	0.906922
	ENSG00000004059	ARF5	ADP ribosylation factor 5	0.739554	0.905469
	ENSG00000166171	DPCD	deleted in primary ciliary dyskinesia homolog (mouse)	0.96135	0.905369
	ENSG00000178053	MLF1	myeloid leukemia factor 1	1.03219	0.903088
	ENSG00000082482	KCNK2	potassium two pore domain channel subfamily K member 2	1.34367	0.902705
	ENSG00000162636	FAM102B	family with sequence similarity 102 member B	1.13096	0.902373
	ENSG00000164627	KIF6	kinesin family member 6	0.904727	0.902278
	ENSG00000180185	FAHD1	fumarylacetoacetate hydrolase domain containing 1	0.636851	0.901924
	ENSG00000183765	CHEK2	checkpoint kinase 2	0.827479	0.90164
	ENSG00000182518	FAM104B	family with sequence similarity 104 member B	0.817937	0.900684
	ENSG00000070501	POLB	DNA polymerase beta	0.679722	0.900578
	ENSG00000178773	CPNE7	copine 7	0.884544	0.900155
	ENSG00000136997	MYC	v-myc avian myelocytomatosis viral oncogene homolog	0.888765	0.900107

	ENSG00000166402	TUB	tubby bipartite transcription factor	1.14111	0.899701
	ENSG00000113916	BCL6	B-cell CLL/lymphoma 6	1.04653	0.899591
	ENSG00000094880	CDC23	cell division cycle 23	0.89469	0.898371
	ENSG00000197261	C6orf141	chromosome 6 open reading frame 141	0.756503	0.897626
	ENSG00000009950	MLXIPL	MLX interacting protein like	0.650685	0.897178
	ENSG00000110042	DTX4	deltex E3 ubiquitin ligase 4	1.46128	0.89692
	ENSG00000112039	FANCE	Fanconi anemia complementation group E	0.788509	0.896597
	ENSG00000115486	GGCX	gamma-glutamyl carboxylase	0.84325	0.896333
	ENSG00000183741	CBX6	chromobox 6	0.744626	0.895712
	ENSG00000137692	DCUN1D5	defective in cullin neddylation 1 domain containing 5	0.895144	0.894684
	ENSG00000103550	KNOP1	lysine rich nucleolar protein 1	0.996013	0.893312
	ENSG00000100116	GCAT	glycine C-acetyltransferase	0.816279	0.888683
	ENSG00000149573	MPZL2	myelin protein zero like 2	0.733585	0.887167
	ENSG00000134202	GSTM3	glutathione S-transferase mu 3	0.95424	0.886405
	ENSG00000165795	NDRG2	NDRG family member 2	1.06254	0.885938
	ENSG00000125743	SNRPD2	small nuclear ribonucleoprotein D2 polypeptide	0.748278	0.885684
	ENSG00000187889	C1orf168	chromosome 1 open reading frame 168	1.05566	0.884423
	ENSG00000163923	RPL39L	ribosomal protein L39 like	0.71943	0.884334
	ENSG00000178449	COX14	"COX14, cytochrome c oxidase assembly factor"	0.697269	0.884169
	ENSG00000158882	TOMM40L	translocase of outer mitochondrial membrane 40 like	0.675673	0.883447
	ENSG00000168505	GBX2	gastrulation brain homeobox 2	0.911229	0.883168
	ENSG00000170191	NANP	N-acetylneuraminic acid phosphatase	0.857126	0.883009
	ENSG00000126767	ELK1	"ELK1, ETS transcription factor"	0.774281	0.883002
	ENSG00000182195	LDOC1	leucine zipper down-regulated in cancer 1	0.909283	0.882885
	ENSG00000117385	P3H1	prolyl 3-hydroxylase 1	1.17836	0.882606
	ENSG00000160221	C21orf33	chromosome 21 open reading frame 33	0.576514	0.882266
	ENSG00000103187	COTL1	coactosin like F-actin binding protein 1	0.904161	0.881233
	ENSG00000139726	DENR	density regulated re-initiation and release factor	0.927438	0.880601
	ENSG00000105227	PRX	periaxin	1.08466	0.880227
	ENSG00000075618	FSCN1	fascin actin-bundling protein 1	0.883641	0.88007
	ENSG00000171858	RPS21	ribosomal protein S21	0.612498	0.878411
	ENSG00000235655	H3F3AP4	"H3 histone, family 3A, pseudogene 4"	0.994267	0.877585
	ENSG00000157111	TMEM171	transmembrane protein 171	0.878415	0.876701
	ENSG00000128228	SDF2L1	stromal cell derived factor 2 like 1	0.554218	0.876166
	ENSG00000117407	ARTN	artemin	0.779861	0.876064
	ENSG00000128708	HAT1	histone acetyltransferase 1	0.915354	0.875117
	ENSG00000100483	VCPKMT	valosin containing protein lysine methyltransferase	0.605394	0.87448
	ENSG00000143155	TIPRL	TOR signaling pathway regulator	0.811212	0.873609
	ENSG00000243479	MNX1-AS1	MNX1 antisense RNA 1 (head to head)	0.688844	0.870034
	ENSG00000148925	BTBD10	BTB domain containing 10	0.947219	0.868464
	ENSG00000107949	BCCIP	BRCA2 and CDKN1A interacting protein	0.802714	0.867996
	ENSG00000112164	GLP1R	glucagon like peptide 1 receptor	0.964153	0.86756
	ENSG00000164039	BDH2	"3-hydroxybutyrate dehydrogenase, type 2"	0.843871	0.866672
	ENSG00000119705	SLIRP	SRA stem-loop interacting RNA binding protein	0.769817	0.866559
	ENSG00000215386	MIR99AHG	mir-99a-let-7c cluster host gene	0.706262	0.866057
	ENSG00000132780	NASP	nuclear autoantigenic sperm protein	0.889616	0.865998
	ENSG00000140395	WDR61	WD repeat domain 61	0.783432	0.865257
	ENSG00000130066	SAT1	spermidine/spermine N1-acetyltransferase 1	0.701296	0.864274
	ENSG00000104756	KCTD9	potassium channel tetramerization domain containing 9	0.999242	0.863835
	ENSG00000108219	TSPAN14	tetraspanin 14	0.869488	0.86275
	ENSG00000131876	SNRPA1	small nuclear ribonucleoprotein polypeptide A'	0.885467	0.861662
	ENSG00000141622	RNF165	ring finger protein 165	1.06013	0.861514
	ENSG00000175061	LRRC75A-AS1	LRRC75A antisense RNA 1	0.730571	0.859008
	ENSG00000109475	RPL34	ribosomal protein L34	0.791899	0.858151
	ENSG00000164983	TMEM65	transmembrane protein 65	1.01325	0.856892
	ENSG00000102996	MMP15	matrix metallopeptidase 15	0.898084	0.856742
	ENSG00000253626	EIF5AL1	eukaryotic translation initiation factor 5A-like 1	0.937528	0.856409
	ENSG00000125901	MRPS26	mitochondrial ribosomal protein S26	0.590285	0.855188
	ENSG00000171953	ATPAF2	ATP synthase mitochondrial F1 complex assembly factor 2	0.668871	0.853675
	ENSG00000108561	C1QBP	complement C1q binding protein	0.842575	0.853417
	ENSG00000092036	HAUS4	HAUS augmin like complex subunit 4	0.81204	0.852708
	ENSG00000143486	EIF2D	eukaryotic translation initiation factor 2D	0.89687	0.852116

	ENSG00000120322	PCDHB8	protocadherin beta 8	1.12031	0.851423
	ENSG00000106628	POLD2	"DNA polymerase delta 2, accessory subunit"	0.947729	0.850791
	ENSG00000100600	LGMN	legumain	0.997135	0.849279
	ENSG00000139547	RDH16	retinol dehydrogenase 16 (all-trans)	0.549674	0.848992
	ENSG00000173638	SLC19A1	solute carrier family 19 member 1	0.711999	0.848981
	ENSG00000166961	MS4A15	membrane spanning 4-domains A15	1.42859	0.848195
	ENSG00000127152	BCL11B	B-cell CLL/lymphoma 11B	0.889491	0.847944
	ENSG00000214113	LYRM4	LYR motif containing 4	0.816561	0.847357
	ENSG00000169740	ZNF32	zinc finger protein 32	0.790834	0.846533
	ENSG00000153574	RPIA	ribose 5-phosphate isomerase A	0.794538	0.845553
	ENSG00000222041	LINC00152	long intergenic non-protein coding RNA 152	0.642621	0.845507
	ENSG00000088832	FKBP1A	FK506 binding protein 1A	0.791857	0.845083
	ENSG00000075336	TIMM21	translocase of inner mitochondrial membrane 21	0.679132	0.844579
	ENSG00000174672	BRSK2	BR serine/threonine kinase 2	1.05141	0.844313
	ENSG00000111911	HINT3	histidine triad nucleotide binding protein 3	0.862346	0.844222
	ENSG00000150433	TMEM218	transmembrane protein 218	0.819994	0.843916
	ENSG00000197563	PIGN	phosphatidylinositol glycan anchor biosynthesis class N	1.27463	0.843688
	ENSG00000148803	FUOM	fucose mutarotase	0.512164	0.843375
	ENSG00000126953	TIMM8A	translocase of inner mitochondrial membrane 8 homolog A (yeast)	0.716225	0.843205
	ENSG00000088035	ALG6	"ALG6, alpha-1,3-glucosyltransferase"	0.725901	0.842662
	ENSG00000229117	RPL41	ribosomal protein L41	0.863473	0.842525
	ENSG00000145912	NHP2	NHP2 ribonucleoprotein	0.656477	0.842416
	ENSG00000087510	TFAP2C	transcription factor AP-2 gamma	0.827	0.84
	ENSG00000152939	MARVELD2	MARVEL domain containing 2	0.848956	0.83838
	ENSG00000106603	COA1	cytochrome c oxidase assembly factor 1 homolog	0.866394	0.834569
	ENSG00000122406	RPL5	ribosomal protein L5	0.876493	0.834361
	ENSG00000241127	YAE1D1	Yae1 domain containing 1	0.528961	0.833542
	ENSG00000165916	PSMC3	"proteasome 26S subunit, ATPase 3"	0.867627	0.83266
	ENSG00000131174	COX7B	cytochrome c oxidase subunit 7B	0.743747	0.832255
	ENSG00000134882	UBAC2	UBA domain containing 2	0.761043	0.832168
	ENSG00000114054	PCCB	propionyl-CoA carboxylase beta subunit	0.879864	0.830985
	ENSG00000137834	SMAD6	SMAD family member 6	0.666859	0.829978
	ENSG00000157827	FMNL2	formin like 2	0.941409	0.829786
	ENSG00000136636	KCTD3	potassium channel tetramerization domain containing 3	0.882766	0.829573
	ENSG00000204308	RNF5	ring finger protein 5	0.74448	0.82896
	ENSG00000170836	PPM1D	"protein phosphatase, Mg2+/Mn2+ dependent 1D"	0.805558	0.828481
	ENSG00000189046	ALKBH2	"alkB homolog 2, alpha-ketoglutarate dependent dioxygenase"	0.534917	0.826004
	ENSG00000116833	NR5A2	nuclear receptor subfamily 5 group A member 2	1.21764	0.825542
	ENSG00000183010	PYCR1	pyrroline-5-carboxylate reductase 1	0.667415	0.824556
	ENSG00000261437	LOC100288748	uncharacterized LOC100288748	0.514374	0.824425
	ENSG00000250588	IQCJ-SCHIP1	IQCJ-SCHIP1 readthrough	1.07634	0.824338
	ENSG00000164967	RPP25L	ribonuclease P/MRP subunit p25 like	0.589825	0.823949
	ENSG00000073464	CLCN4	chloride voltage-gated channel 4	1.05748	0.823801
	ENSG00000169902	TPST1	tyrosylprotein sulfotransferase 1	0.973886	0.823435
	ENSG00000181350	LRRC75A	leucine rich repeat containing 75A	0.828751	0.822573
	ENSG00000135549	PKIB	"protein kinase (cAMP-dependent, catalytic) inhibitor beta"	0.923158	0.820975
	ENSG00000169021	UQCRFS1	"ubiquinol-cytochrome c reductase, Rieske iron-sulfur polypeptide 1"	0.673997	0.820654
	ENSG00000138035	PNPT1	polyribonucleotide nucleotidyltransferase 1	0.771053	0.820103
	ENSG00000099284	H2AFY2	H2A histone family member Y2	0.758477	0.819214
	ENSG00000105552	BCAT2	branched chain amino acid transaminase 2	0.627225	0.819078
	ENSG00000003436	TFPI	tissue factor pathway inhibitor	0.685574	0.818259
	ENSG00000111850	SMIM8	small integral membrane protein 8	0.664991	0.817361
	ENSG00000139428	MMAB	methylmalonic aciduria (cobalamin deficiency) cblB type	0.698355	0.816792
	ENSG00000165028	NIPSNAP3B	nipsnap homolog 3B	0.977539	0.816229
	ENSG00000176124	DLEU1	deleted in lymphocytic leukemia 1	1.11632	0.816194
	ENSG00000072954	TMEM38A	transmembrane protein 38A	0.820518	0.816127
	ENSG00000203875	SNHG5	small nucleolar RNA host gene 5	0.587049	0.815636
	ENSG00000173467	AGR3	"anterior gradient 3, protein disulphide isomerase family member"	0.59845	0.815454
	ENSG00000164163	ABCE1	ATP binding cassette subfamily E member 1	0.876384	0.815314

	ENSG00000137038	TMEM261	transmembrane protein 261	0.671823	0.814308
	ENSG00000155508	CNOT8	CCR4-NOT transcription complex subunit 8	0.78093	0.812067
	ENSG00000164096	C4orf3	chromosome 4 open reading frame 3	0.665988	0.811939
	ENSG00000026950	BTN3A1	butyrophilin subfamily 3 member A1	0.701139	0.811308
	ENSG00000155876	RRAGA	Ras related GTP binding A	0.713067	0.811297
	ENSG00000171421	MRPL36	mitochondrial ribosomal protein L36	0.541391	0.810266
	ENSG00000164751	PEX2	peroxisomal biogenesis factor 2	0.769444	0.809132
	ENSG00000071462	WBSCR22	Williams-Beuren syndrome chromosome region 22	0.693725	0.809007
	ENSG00000112667	DNPH1	2'-deoxynucleoside 5'-phosphate N-hydrolase 1	0.415004	0.808615
	ENSG00000120093	HOXB3	homeobox B3	0.917468	0.808306
	ENSG00000134759	ELP2	elongator acetyltransferase complex subunit 2	0.751258	0.807974
	ENSG00000150093	ITGB1	integrin subunit beta 1	0.987881	0.80671
	ENSG00000168389	MFSD2A	major facilitator superfamily domain containing 2A	1.167	0.806571
	ENSG00000145220	LYAR	Ly1 antibody reactive	0.775255	0.806391
	ENSG00000104064	GABPB1	GA binding protein transcription factor beta subunit 1	0.78914	0.806187
	ENSG00000134375	TIMM17A	translocase of inner mitochondrial membrane 17 homolog A (yeast)	0.741463	0.806169
	ENSG00000169020	ATP5I	"ATP synthase, H+ transporting, mitochondrial Fo complex subunit E"	0.572728	0.805795
	ENSG00000128694	OSGEPL1	O-sialoglycoprotein endopeptidase like 1	0.946834	0.805438
	ENSG00000113621	TXNDC15	thioredoxin domain containing 15	0.674132	0.804971
	ENSG00000172345	STARD5	StAR related lipid transfer domain containing 5	0.757691	0.804855
	ENSG00000151729	SLC25A4	solute carrier family 25 member 4	0.688413	0.804581
	ENSG00000114391	RPL24	ribosomal protein L24	0.775667	0.80338
	ENSG00000185627	PSMD13	"proteasome 26S subunit, non-ATPase 13"	0.720864	0.803083
	ENSG00000168769	TET2	tet methylcytosine dioxygenase 2	0.974007	0.8011
	ENSG00000165264	NDUFB6	NADH:ubiquinone oxidoreductase subunit B6	0.611903	0.800763
	ENSG00000124641	MED20	mediator complex subunit 20	0.783132	0.800067
	ENSG00000167693	NXN	nucleoredoxin	0.717945	0.799884
	ENSG00000140740	UQCRC2	ubiquinol-cytochrome c reductase core protein II	0.756596	0.79963
	ENSG00000146950	SHROOM2	shroom family member 2	0.930158	0.799281
	ENSG00000170260	ZNF212	zinc finger protein 212	0.573246	0.799225
	ENSG00000184182	UBE2F	ubiquitin conjugating enzyme E2 F (putative)	0.847813	0.799021
	ENSG00000254999	BRK1	"BRICK1, SCAR/WAVE actin nucleating complex subunit"	0.750387	0.79901
	ENSG00000189120	SP6	Sp6 transcription factor	0.627875	0.798127
	ENSG00000163682	RPL9	ribosomal protein L9	0.790399	0.797841
	ENSG00000181392	SYNE4	spectrin repeat containing nuclear envelope family member 4	0.83352	0.797764
	ENSG00000237190	CDKN2AIPNL	CDKN2A interacting protein N-terminal like	0.749947	0.797643
	ENSG00000129195	FAM64A	family with sequence similarity 64 member A	0.685687	0.797623
	ENSG00000238227	C9orf69	chromosome 9 open reading frame 69	0.476335	0.79721
	ENSG00000108830	RND2	Rho family GTPase 2	0.528641	0.797044
	ENSG00000198042	MAK16	MAK16 homolog	0.777235	0.79678
	ENSG00000132661	NXT1	nuclear transport factor 2 like export factor 1	0.594323	0.796174
	ENSG00000262814	MRPL12	mitochondrial ribosomal protein L12	0.508657	0.795649
	ENSG00000088356	PDRG1	p53 and DNA damage regulated 1	0.59653	0.79471
	ENSG00000243147	MRPL33	mitochondrial ribosomal protein L33	0.747995	0.79456
	ENSG00000198918	RPL39	ribosomal protein L39	0.790025	0.793589
	ENSG00000156384	SFR1	SWI5 dependent homologous recombination repair protein 1	0.733793	0.792653
	ENSG00000184402	SS18L1	"SS18L1, nBAF chromatin remodeling complex subunit"	0.699734	0.792439
	ENSG00000112290	WASF1	WAS protein family member 1	1.0033	0.791938
	ENSG00000013561	RNF14	ring finger protein 14	0.744299	0.790873
	ENSG00000138138	ATAD1	"ATPase family, AAA domain containing 1"	0.789302	0.790079
	ENSG00000069956	MAPK6	mitogen-activated protein kinase 6	0.93147	0.789119
	ENSG00000106153	CHCHD2	coiled-coil-helix-coiled-coil-helix domain containing 2	0.673485	0.788832
	ENSG00000104131	EIF3J	eukaryotic translation initiation factor 3 subunit J	0.920679	0.788568
	ENSG00000173660	UQCRH	ubiquinol-cytochrome c reductase hinge protein	0.730635	0.787734
	ENSG00000023892	DEF6	"DEF6, guanine nucleotide exchange factor"	0.674619	0.787547
	ENSG00000136897	MRPL50	mitochondrial ribosomal protein L50	0.727963	0.787498
	ENSG00000166347	CYB5A	cytochrome b5 type A	0.776375	0.787099
	ENSG00000179431	FJX1	four jointed box 1	0.673003	0.786922
	ENSG00000173436	MINOS1	mitochondrial inner membrane organizing system 1	0.553804	0.786255

	ENSG00000135469	COQ10A	coenzyme Q10A	0.61573	0.786051
	ENSG00000223802	GDF1	growth differentiation factor 1	0.939212	0.785672
	ENSG00000113387	SUB1	"SUB1 homolog, transcriptional regulator"	0.789299	0.78565
	ENSG00000160703	NLRX1	NLR family member X1	0.861659	0.784992
	ENSG00000118690	ARMC2	armadillo repeat containing 2	0.696103	0.782873
	ENSG00000027001	MIPEP	mitochondrial intermediate peptidase	0.710182	0.782334
	ENSG00000214114	MYCBP	MYC binding protein	0.834701	0.780983
	ENSG00000175279	APITD1	"apoptosis-inducing, TAF9-like domain 1"	0.815897	0.780221
	ENSG00000180777	ANKRD30B	ankyrin repeat domain 30B	0.863248	0.779758
	ENSG00000163138	PACRGL	PARK2 coregulated like	0.71929	0.778749
	ENSG00000076248	UNG	uracil DNA glycosylase	0.816379	0.778265
	ENSG00000170846	LOC93622	Morf4 family associated protein 1 like 1 pseudogene	0.617748	0.775301
	ENSG00000267296	CEBPA-AS1	CEBPA antisense RNA 1 (head to head)	0.653322	0.775162
	ENSG00000125991	ERGIC3	ERGIC and golgi 3	0.703577	0.774335
	ENSG00000124614	RPS10	ribosomal protein S10	0.69203	0.772701
	ENSG00000185432	METTL7A	methyltransferase like 7A	0.867371	0.772139
	ENSG00000143622	RIT1	Ras like without CAAX 1	0.743862	0.771635
	ENSG00000114686	MRPL3	mitochondrial ribosomal protein L3	0.752811	0.771381
	ENSG00000137168	PPIL1	peptidylprolyl isomerase like 1	0.72316	0.771098
	ENSG00000114650	SCAP	SREBF chaperone	0.857089	0.770539
	ENSG00000120158	RCL1	RNA terminal phosphate cyclase like 1	0.58902	0.770248
	ENSG00000133818	RRAS2	related RAS viral (r-ras) oncogene homolog 2	0.839157	0.770152
	ENSG00000156253	RWDD2B	RWD domain containing 2B	0.760684	0.77008
	ENSG00000168116	KIAA1586	KIAA1586	0.802731	0.769537
	ENSG00000101003	GINS1	GINS complex subunit 1	0.825391	0.768927
	ENSG00000177508	IRX3	iroquois homeobox 3	0.704955	0.768635
	ENSG00000143621	ILF2	interleukin enhancer binding factor 2	0.778354	0.768432
	ENSG00000125843	AP5S1	adaptor related protein complex 5 sigma 1 subunit	0.456215	0.768152
	ENSG00000079739	PGM1	phosphoglucomutase 1	0.901442	0.766433
	ENSG00000106367	AP1S1	adaptor related protein complex 1 sigma 1 subunit	0.712681	0.766289
	ENSG00000142937	RPS8	ribosomal protein S8	0.798614	0.76595
	ENSG00000166902	MRPL16	mitochondrial ribosomal protein L16	0.6387	0.765245
	ENSG00000112972	HMGCS1	3-hydroxy-3-methylglutaryl-CoA synthase 1	0.995823	0.765179
	ENSG00000183527	PSMG1	proteasome assembly chaperone 1	0.681266	0.765086
	ENSG00000164687	FABP5	fatty acid binding protein 5	0.582434	0.764601
	ENSG00000002549	LAP3	leucine aminopeptidase 3	0.654389	0.76435
	ENSG00000155660	PDIA4	protein disulfide isomerase family A member 4	0.832177	0.763579
	ENSG00000114812	VIPR1	vasoactive intestinal peptide receptor 1	0.945101	0.763107
	ENSG00000132507	EIF5A	eukaryotic translation initiation factor 5A	0.682438	0.762379
	ENSG00000154930	ACSS1	acyl-CoA synthetase short-chain family member 1	0.732909	0.762267
	ENSG00000073282	TP63	tumor protein p63	1.17284	0.761436
	ENSG00000169139	UBE2V2	ubiquitin conjugating enzyme E2 V2	0.847768	0.761367
	ENSG00000189403	HMGB1	high mobility group box 1	0.862235	0.760277
	ENSG00000165506	DNAAF2	dynein axonemal assembly factor 2	0.627346	0.76001
	ENSG00000071994	PDCD2	programmed cell death 2	0.709717	0.75917
	ENSG00000143319	ISG20L2	interferon stimulated exonuclease gene 20 like 2	0.800567	0.759151
	ENSG00000138587	MNS1	meiosis specific nuclear structural 1	0.840035	0.758889
	ENSG00000175093	SPSB4	splA/ryanodine receptor domain and SOCS box containing 4	1.17641	0.758689
	ENSG00000265688	MAFG-AS1	MAFG antisense RNA 1 (head to head)	0.488091	0.758365
	ENSG00000139620	KANSL2	KAT8 regulatory NSL complex subunit 2	0.647085	0.758256
	ENSG00000137210	TMEM14B	transmembrane protein 14B	0.746991	0.758224
	ENSG00000181924	COA4	cytochrome c oxidase assembly factor 4 homolog	0.567114	0.757669
	ENSG00000146963	LUC7L2	"LUC7 like 2, pre-mRNA splicing factor"	0.803454	0.757396
	ENSG00000170425	ADORA2B	adenosine A2b receptor	0.715716	0.757347
	ENSG00000116353	MECR	mitochondrial trans-2-enoyl-CoA reductase	0.629844	0.756609
	ENSG00000159792	PSKH1	protein serine kinase H1	0.564947	0.756137
	ENSG00000090971	NAT14	N-acetyltransferase 14 (putative)	0.436621	0.755597
	ENSG00000182899	RPL35A	ribosomal protein L35a	0.687875	0.754828
	ENSG00000196782	MAML3	mastermind like transcriptional coactivator 3	1.17245	0.753546
	ENSG00000102390	PBDC1	polysaccharide biosynthesis domain containing 1	0.702885	0.752183
	ENSG00000198876	DCAF12	DDB1 and CUL4 associated factor 12	0.792873	0.751584
	ENSG00000035499	DEPDC1B	DEP domain containing 1B	0.948026	0.751444
	ENSG00000137154	RPS6	ribosomal protein S6	0.80126	0.751089
	ENSG00000109534	GAR1	GAR1 ribonucleoprotein	0.712978	0.750716

	ENSG00000105948	TTC26	tetratricopeptide repeat domain 26	0.645484	0.750698
	ENSG00000198755	RPL10A	ribosomal protein L10a	0.714578	0.750405
	ENSG00000091140	DLD	dihydrolipoamide dehydrogenase	0.797122	0.750107
	ENSG00000140416	TPM1	tropomyosin 1 (alpha)	0.801783	0.748702
	ENSG00000071553	ATP6AP1	ATPase H+ transporting accessory protein 1	0.725615	0.747385
	ENSG00000106588	PSMA2	proteasome subunit alpha 2	0.711013	0.747366
	ENSG00000104915	STX10	syntaxin 10	0.682659	0.746872
	ENSG00000105173	CCNE1	cyclin E1	0.708192	0.746412
	ENSG00000214706	IFRD2	interferon related developmental regulator 2	0.559552	0.745983
	ENSG00000164182	NDUFAF2	NADH:ubiquinone oxidoreductase complex assembly factor 2	0.783876	0.745836
	ENSG00000164305	CASP3	caspase 3	0.729553	0.745127
	ENSG00000149273	RPS3	ribosomal protein S3	0.756681	0.745014
	ENSG00000172500	FIBP	FGF1 intracellular binding protein	0.762325	0.744858
	ENSG00000144120	TMEM177	transmembrane protein 177	0.455281	0.744802
	ENSG00000161267	BDH1	"3-hydroxybutyrate dehydrogenase, type 1"	0.715878	0.744706
	ENSG00000213782	DDX47	DEAD-box helicase 47	0.765994	0.743859
	ENSG00000164978	NUDT2	nudix hydrolase 2	0.587172	0.742747
	ENSG00000215021	PHB2	prohibitin 2	0.658567	0.742473
	ENSG00000130414	NDUFA10	NADH:ubiquinone oxidoreductase subunit A10	0.587629	0.742002
	ENSG00000163738	MTHFD2L	methylenetetrahydrofolate dehydrogenase (NADP+ dependent) 2-like	0.589263	0.741144
	ENSG00000047634	SCML1	sex comb on midleg-like 1 (Drosophila)	0.763639	0.740902
	ENSG00000129071	MBD4	"methyl-CpG binding domain 4, DNA glycosylase"	0.679262	0.740153
	ENSG00000174173	TRMT10C	"tRNA methyltransferase 10C, mitochondrial RNase P subunit"	0.656297	0.739582
	ENSG00000083845	RPS5	ribosomal protein S5	0.563525	0.738254
	ENSG00000165997	ARL5B	ADP ribosylation factor like GTPase 5B	0.799245	0.737761
	ENSG00000122026	RPL21	ribosomal protein L21	0.755891	0.73765
	ENSG00000070831	CDC42	cell division cycle 42	0.724433	0.737603
	ENSG00000152795	HNRNPDL	heterogeneous nuclear ribonucleoprotein D like	0.624528	0.737366
	ENSG00000182512	GLRX5	glutaredoxin 5	0.741695	0.736694
	ENSG00000056736	IL17RB	interleukin 17 receptor B	0.884077	0.736462
	ENSG00000105607	GCDH	glutaryl-CoA dehydrogenase	0.848594	0.735966
	ENSG00000059378	PARP12	poly(ADP-ribose) polymerase family member 12	0.41123	0.735736
	ENSG00000232112	TMA7	translation machinery associated 7 homolog	0.568972	0.735707
	ENSG00000164405	UQCRQ	ubiquinol-cytochrome c reductase complex III subunit VII	0.655835	0.735397
	ENSG00000110955	ATP5B	"ATP synthase, H+ transporting, mitochondrial F1 complex, beta polypeptide"	0.738957	0.734603
	ENSG00000105677	TMEM147	transmembrane protein 147	0.659977	0.73448
	ENSG00000136930	PSMB7	proteasome subunit beta 7	0.703839	0.733512
	ENSG00000183605	SFXN4	sideroflexin 4	0.706769	0.733447
	ENSG00000135587	SMPD2	sphingomyelin phosphodiesterase 2	0.898892	0.733124
	ENSG00000133678	TMEM254	transmembrane protein 254	0.725767	0.733062
	ENSG00000123600	METTL8	methyltransferase like 8	0.803202	0.731764
	ENSG00000231500	RPS18	ribosomal protein S18	0.638633	0.730978
	ENSG00000152782	PANK1	pantothenate kinase 1	0.797274	0.730937
	ENSG00000146535	GNA12	G protein subunit alpha 12	0.878071	0.730274
	ENSG00000181817	LSM10	"LSM10, U7 small nuclear RNA associated"	0.568817	0.730227
	ENSG00000109911	ELP4	elongator acetyltransferase complex subunit 4	0.670908	0.728065
	ENSG00000169738	DCXR	dicarbonyl and L-xylulose reductase	0.639569	0.72724
	ENSG00000100865	CINP	cyclin dependent kinase 2 interacting protein	0.658014	0.726846
	ENSG00000147155	EBP	emopamil binding protein (sterol isomerase)	0.72739	0.726358
	ENSG00000087053	MTMR2	myotubularin related protein 2	0.910336	0.726056
	ENSG00000161980	POLR3K	RNA polymerase III subunit K	0.601195	0.724844
	ENSG00000206503	HLA-A	"major histocompatibility complex, class I, A"	0.642678	0.723941
	ENSG00000198142	SOWAHC	sosondowah ankyrin repeat domain family member C	0.757397	0.721909
	ENSG00000141384	TAF4B	TATA-box binding protein associated factor 4b	0.927441	0.721482
	ENSG00000196976	LAGE3	L antigen family member 3	0.51342	0.72033
	ENSG00000137563	GGH	gamma-glutamyl hydrolase	0.863208	0.719927
	ENSG00000140718	FTO	fat mass and obesity associated	0.807692	0.719746
	ENSG00000224877	NDUFAF8	NADH:ubiquinone oxidoreductase complex assembly factor 8	0.438455	0.719152
	ENSG00000132341	RAN	"RAN, member RAS oncogene family"	0.649795	0.716611
	ENSG00000070761	CFAP20	cilia and flagella associated protein 20	0.673339	0.71513

	ENSG00000185989	RASA3	RAS p21 protein activator 3	0.752342	0.715053
	ENSG00000154642	C21orf91	chromosome 21 open reading frame 91	0.691422	0.713411
	ENSG00000167283	ATP5L	"ATP synthase, H+ transporting, mitochondrial Fo complex subunit G"	0.641905	0.713396
	ENSG00000197345	MRPL21	mitochondrial ribosomal protein L21	0.620408	0.711494
	ENSG00000108651	UTP6	"UTP6, small subunit processome component"	0.626186	0.711473
	ENSG00000171954	CYP4F22	cytochrome P450 family 4 subfamily F member 22	0.607833	0.711376
	ENSG00000197728	RPS26	ribosomal protein S26	0.600671	0.710175
	ENSG00000122783	C7orf49	chromosome 7 open reading frame 49	0.651726	0.709833
	ENSG00000116285	ERRFI1	ERBB receptor feedback inhibitor 1	0.525362	0.709608
	ENSG00000075413	MARK3	microtubule affinity regulating kinase 3	0.541086	0.708198
	ENSG00000159228	CBR1	carbonyl reductase 1	0.60378	0.707936
	ENSG00000123179	EBPL	emopamil binding protein like	0.759841	0.707649
	ENSG00000136770	DNAJC1	DnaJ heat shock protein family (Hsp40) member C1	0.628416	0.707132
	ENSG00000051596	THOC3	THO complex 3	0.747607	0.706681
	ENSG00000092094	OSGEP	O-sialoglycoprotein endopeptidase	0.590015	0.706425
	ENSG00000127334	DYRK2	dual specificity tyrosine phosphorylation regulated kinase 2	0.937423	0.706077
	ENSG00000217555	CKLF	chemokine like factor	0.618983	0.705915
	ENSG00000178537	SLC25A20	solute carrier family 25 member 20	0.55318	0.705508
	ENSG00000132591	ERAL1	Era like 12S mitochondrial rRNA chaperone 1	0.638027	0.704868
	ENSG00000167862	MRPL58	mitochondrial ribosomal protein L58	0.589747	0.704769
	ENSG00000125484	GTF3C4	general transcription factor IIIC subunit 4	0.760099	0.704617
	ENSG00000116922	C1orf109	chromosome 1 open reading frame 109	0.578077	0.703725
	ENSG00000111832	RWDD1	RWD domain containing 1	0.790947	0.703144
	ENSG00000187713	TMEM203	transmembrane protein 203	0.536841	0.70267
	ENSG00000144354	CDCA7	cell division cycle associated 7	0.780818	0.700558
	ENSG00000107223	EDF1	endothelial differentiation related factor 1	0.499046	0.698612
	ENSG00000154217	PITPNC1	"phosphatidylinositol transfer protein, cytoplasmic 1"	0.752945	0.698521
	ENSG00000137267	TUBB2A	tubulin beta 2A class IIa	0.556047	0.69846
	ENSG00000175322	ZNF519	zinc finger protein 519	0.57224	0.698319
	ENSG00000047621	C12orf4	chromosome 12 open reading frame 4	0.589379	0.697994
	ENSG00000055211	GINM1	glycoprotein integral membrane 1	0.775513	0.697549
	ENSG00000066379	ZNRD1	zinc ribbon domain containing 1	0.479523	0.696981
	ENSG00000080189	SLC35C2	solute carrier family 35 member C2	0.614949	0.696564
	ENSG00000179862	CITED4	Cbp/p300 interacting transactivator with Glu/Asp rich carboxy-terminal domain 4	0.482466	0.696028
	ENSG00000076067	RBMS2	RNA binding motif single stranded interacting protein 2	0.988779	0.695869
	ENSG00000088451	TGDS	"TDP-glucose 4,6-dehydratase"	0.708024	0.695662
	ENSG00000182307	C8orf33	chromosome 8 open reading frame 33	0.660074	0.695409
	ENSG00000166441	RPL27A	ribosomal protein L27a	0.602351	0.694477
	ENSG00000186132	C2orf76	chromosome 2 open reading frame 76	0.748832	0.69391
	ENSG00000004779	NDUFAB1	NADH:ubiquinone oxidoreductase subunit AB1	0.596311	0.692596
	ENSG00000112977	DAP	death associated protein	0.676437	0.692041
	ENSG00000168028	RPSA	ribosomal protein SA	0.728888	0.69114
	ENSG00000078070	MCCC1	methylcrotonoyl-CoA carboxylase 1	0.606684	0.691066
	ENSG00000169684	CHRNA5	cholinergic receptor nicotinic alpha 5 subunit	0.704088	0.690746
	ENSG00000142541	RPL13A	ribosomal protein L13a	0.565281	0.690617
	ENSG00000117691	NENF	neudesin neurotrophic factor	0.63689	0.689993
	ENSG00000231584	FAHD2CP	"fumarylacetoacetate hydrolase domain containing 2C, pseudogene"	0.698245	0.689497
	ENSG00000109919	MTCH2	mitochondrial carrier 2	0.643666	0.688932
	ENSG00000012660	ELOVL5	ELOVL fatty acid elongase 5	0.835545	0.688405
	ENSG00000164976	KIAA1161	KIAA1161	0.655048	0.688324
	ENSG00000164442	CITED2	Cbp/p300 interacting transactivator with Glu/Asp rich carboxy-terminal domain 2	0.558277	0.687849
	ENSG00000137513	NARS2	"asparaginyl-tRNA synthetase 2, mitochondrial (putative)"	0.792649	0.687521
	ENSG00000163818	LZTFL1	leucine zipper transcription factor like 1	0.597137	0.686555
	ENSG00000187193	MT1X	metallothionein 1X	0.426134	0.685788
	ENSG00000076513	ANKRD13A	ankyrin repeat domain 13A	0.696859	0.685671
	ENSG00000176903	PNMA1	paraneoplastic Ma antigen 1	0.569994	0.68552
	ENSG00000100804	PSMB5	proteasome subunit beta 5	0.647283	0.685028

	ENSG00000132467	UTP3	"UTP3, small subunit processome component homolog (S. cerevisiae)"	0.539582	0.683986
	ENSG00000166710	B2M	beta-2-microglobulin	0.660654	0.683885
	ENSG00000172115	CYCS	"cytochrome c, somatic"	0.634708	0.683778
	ENSG00000163249	CCNYL1	cyclin Y like 1	0.891729	0.683649
	ENSG00000164109	MAD2L1	MAD2 mitotic arrest deficient-like 1 (yeast)	0.684397	0.683566
	ENSG00000074201	CLNS1A	chloride nucleotide-sensitive channel 1A	0.581412	0.683439
	ENSG00000077549	CAPZB	capping actin protein of muscle Z-line beta subunit	0.666538	0.682933
	ENSG00000163382	NAXE	NAD(P)HX epimerase	0.699496	0.682152
	ENSG00000148335	NTMT1	N-terminal Xaa-Pro-Lys N-methyltransferase 1	0.366753	0.68196
	ENSG00000136942	RPL35	ribosomal protein L35	0.587071	0.681671
	ENSG00000162961	DPY30	"dpy-30, histone methyltransferase complex regulatory subunit"	0.745031	0.681298
	ENSG00000152455	SUV39H2	suppressor of variegation 3-9 homolog 2	0.68922	0.680572
	ENSG00000116273	PHF13	PHD finger protein 13	0.548822	0.679934
	ENSG00000117697	NSL1	"NSL1, MIS12 kinetochore complex component"	0.575272	0.679574
	ENSG00000204392	LSM2	"LSM2 homolog, U6 small nuclear RNA and mRNA degradation associated"	0.576788	0.679191
	ENSG00000138764	CCNG2	cyclin G2	0.662931	0.679027
	ENSG00000186468	RPS23	ribosomal protein S23	0.644234	0.677774
	ENSG00000114850	SSR3	signal sequence receptor subunit 3	0.712814	0.67759
	ENSG00000145990	GFOD1	glucose-fructose oxidoreductase domain containing 1	0.655897	0.677425
	ENSG00000111669	TPI1	triosephosphate isomerase 1	0.662608	0.676696
	ENSG00000162894	FCMR	Fc fragment of IgM receptor	0.62598	0.67407
	ENSG00000174547	MRPL11	mitochondrial ribosomal protein L11	0.561701	0.673595
	ENSG00000177519	RPRM	"reprimo, TP53 dependent G2 arrest mediator candidate"	0.725484	0.672673
	ENSG00000145386	CCNA2	cyclin A2	0.683344	0.672626
	ENSG00000198680	TUSC1	tumor suppressor candidate 1	0.434468	0.671859
	ENSG00000139832	RAB20	"RAB20, member RAS oncogene family"	0.54106	0.671545
	ENSG00000138442	WDR12	WD repeat domain 12	0.631358	0.671058
	ENSG00000187049	TMEM216	transmembrane protein 216	0.578493	0.670693
	ENSG00000137393	RNF144B	ring finger protein 144B	0.536437	0.670161
	ENSG00000150456	EEF1AKMT1	eukaryotic translation elongation factor 1 alpha lysine methyltransferase 1	0.694379	0.670003
	ENSG00000075223	SEMA3C	semaphorin 3C	0.907207	0.669273
	ENSG00000160783	PMF1	polyamine modulated factor 1	0.49455	0.668554
	ENSG00000103121	CMC2	C-X9-C motif containing 2	0.595229	0.668355
	ENSG00000077152	UBE2T	ubiquitin conjugating enzyme E2 T	0.641735	0.668278
	ENSG00000109255	NMU	neuromedin U	1.0744	0.667768
	ENSG00000088766	CRLS1	cardiolipin synthase 1	0.531102	0.667133
	ENSG00000256269	HMBS	hydroxymethylbilane synthase	0.562652	0.666744
	ENSG00000111845	PAK1IP1	PAK1 interacting protein 1	0.562759	0.666736
	ENSG00000158427	TMSB15B	thymosin beta 15B	0.668357	0.666676
	ENSG00000100462	PRMT5	protein arginine methyltransferase 5	0.737209	0.665835
	ENSG00000062582	MRPS24	mitochondrial ribosomal protein S24	0.555455	0.665775
	ENSG00000114942	EEF1B2	eukaryotic translation elongation factor 1 beta 2	0.68232	0.665074
	ENSG00000095203	EPB41L4B	erythrocyte membrane protein band 4.1 like 4B	0.73561	0.664133
	ENSG00000171735	CAMTA1	calmodulin binding transcription activator 1	0.642045	0.662908
	ENSG00000164587	RPS14	ribosomal protein S14	0.610268	0.66288
	ENSG00000198807	PAX9	paired box 9	0.58445	0.662648
	ENSG00000173207	CKS1B	CDC28 protein kinase regulatory subunit 1B	0.59821	0.662221
	ENSG00000132275	RRP8	"ribosomal RNA processing 8, methyltransferase, homolog (yeast)"	0.501806	0.66187
	ENSG00000213339	QTRT1	queuine tRNA-ribosyltransferase catalytic subunit 1	0.471824	0.661715
	ENSG00000272047	GTF2H5	general transcription factor IIH subunit 5	0.482877	0.661587
	ENSG00000108443	RPS6KB1	ribosomal protein S6 kinase B1	0.690924	0.661297
	ENSG00000167526	RPL13	ribosomal protein L13	0.48154	0.660912
	ENSG00000116685	KIAA2013	KIAA2013	0.458529	0.66025
	ENSG00000164008	C1orf50	chromosome 1 open reading frame 50	0.506735	0.660153
	ENSG00000176422	SPRYD4	SPRY domain containing 4	0.498969	0.658557
	ENSG00000125691	RPL23	ribosomal protein L23	0.713014	0.658251
	ENSG00000178913	TAF7	TATA-box binding protein associated factor 7	0.495937	0.658128
	ENSG00000135617	PRADC1	protease associated domain containing 1	0.539953	0.658061
	ENSG00000188372	ZP3	zona pellucida glycoprotein 3	0.598498	0.657539
	ENSG00000165502	RPL36AL	ribosomal protein L36a like	0.544335	0.657496

	ENSG00000117139	KDM5B	lysine demethylase 5B	0.845657	0.65676
	ENSG00000137274	BPHL	biphenyl hydrolase like	0.722898	0.655604
	ENSG00000065328	MCM10	minichromosome maintenance 10 replication initiation factor	0.658391	0.655249
	ENSG00000182004	SNRPE	small nuclear ribonucleoprotein polypeptide E	0.579614	0.655083
	ENSG00000114491	UMPS	uridine monophosphate synthetase	0.618575	0.654156
	ENSG00000118181	RPS25	ribosomal protein S25	0.679746	0.654124
	ENSG00000100811	YY1	YY1 transcription factor	0.656623	0.652961
	ENSG00000139289	PHLDA1	pleckstrin homology like domain family A member 1	0.52809	0.652852
	ENSG00000104549	SQLE	squalene epoxidase	0.859419	0.65273
	ENSG00000114125	RNF7	ring finger protein 7	0.475151	0.652396
	ENSG00000174444	RPL4	ribosomal protein L4	0.727798	0.651767
	ENSG00000114023	FAM162A	family with sequence similarity 162 member A	0.512759	0.651472
	ENSG00000205581	HMGN1	high mobility group nucleosome binding domain 1	0.671758	0.650796
	ENSG00000134533	RERG	RAS like estrogen regulated growth inhibitor	0.739758	0.650693
	ENSG00000107882	SUFU	SUFU negative regulator of hedgehog signaling	0.632929	0.650479
	ENSG00000164258	NDUFS4	NADH:ubiquinone oxidoreductase subunit S4	0.599484	0.649652
	ENSG00000133247	KMT5C	lysine methyltransferase 5C	0.564812	0.649637
	ENSG00000170089	LOC728554	THO complex 3 pseudogene	0.691449	0.649272
	ENSG00000155438	NIFK	nucleolar protein interacting with the FHA domain of MKI67	0.614676	0.649105
	ENSG00000186998	EMID1	EMI domain containing 1	0.485272	0.647797
	ENSG00000089157	RPLP0	ribosomal protein lateral stalk subunit P0	0.690141	0.646959
	ENSG00000105193	RPS16	ribosomal protein S16	0.595941	0.646833
	ENSG00000092068	SLC7A8	solute carrier family 7 member 8	0.772529	0.646335
	ENSG00000198034	RPS4X	"ribosomal protein S4, X-linked"	0.617263	0.645454
	ENSG00000109016	DHRS7B	dehydrogenase/reductase 7B	0.519399	0.643293
	ENSG00000184076	UQCR10	"ubiquinol-cytochrome c reductase, complex III subunit X"	0.440544	0.643124
	ENSG00000169093	ASMTL	acetylserotonin O-methyltransferase-like	0.627943	0.643094
	ENSG00000162594	IL23R	interleukin 23 receptor	0.901047	0.643077
	ENSG00000214253	FIS1	"fission, mitochondrial 1"	0.477	0.64285
	ENSG00000121691	CAT	catalase	0.739667	0.642768
	ENSG00000172965	MIR4435-2HG	MIR4435-2 host gene	0.680475	0.642404
	ENSG00000152102	FAM168B	family with sequence similarity 168 member B	0.732542	0.642111
	ENSG00000171204	TMEM126B	transmembrane protein 126B	0.727283	0.641183
	ENSG00000088986	DYNLL1	dynein light chain LC8-type 1	0.629238	0.640647
	ENSG00000266967	AARSD1	alanyl-tRNA synthetase domain containing 1	0.659615	0.639813
	ENSG00000223797	ENTPD3-AS1	ENTPD3 antisense RNA 1	0.509316	0.639276
	ENSG00000147654	EBAG9	"estrogen receptor binding site associated, antigen, 9"	0.596474	0.638776
	ENSG00000109270	LAMTOR3	"late endosomal/lysosomal adaptor, MAPK and MTOR activator 3"	0.519875	0.638155
	ENSG00000143207	RFWD2	ring finger and WD repeat domain 2	0.768969	0.637484
	ENSG00000180739	S1PR5	sphingosine-1-phosphate receptor 5	0.544561	0.637201
	ENSG00000173409	ARV1	"ARV1 homolog, fatty acid homeostasis modulator"	0.443626	0.636535
	ENSG00000168393	DTYMK	deoxythymidylate kinase	0.550339	0.636515
	ENSG00000179941	BBS10	Bardet-Biedl syndrome 10	0.549438	0.635606
	ENSG00000198805	PNP	purine nucleoside phosphorylase	0.562185	0.634676
	ENSG00000186205	MARC1	mitochondrial amidoxime reducing component 1	0.739496	0.634046
	ENSG00000100316	RPL3	ribosomal protein L3	0.643843	0.633513
	ENSG00000148677	ANKRD1	ankyrin repeat domain 1	0.498224	0.633051
	ENSG00000165283	STOML2	stomatin like 2	0.620558	0.633006
	ENSG00000213281	NRAS	neuroblastoma RAS viral oncogene homolog	0.650742	0.632976
	ENSG00000117395	EBNA1BP2	EBNA1 binding protein 2	0.637167	0.63253
	ENSG00000175970	UNC119B	unc-119 lipid binding chaperone B	0.57547	0.632513
	ENSG00000146242	TPBG	trophoblast glycoprotein	0.510288	0.632264
	ENSG00000198130	HIBCH	3-hydroxyisobutyryl-CoA hydrolase	0.842222	0.631806
	ENSG00000138385	SSB	Sjogren syndrome antigen B	0.68798	0.631619
	ENSG00000175334	BANF1	barrier to autointegration factor 1	0.576179	0.631429
	ENSG00000116898	MRPS15	mitochondrial ribosomal protein S15	0.589601	0.631072
	ENSG00000183291	SEP15	15 kDa selenoprotein	0.612944	0.630963
	ENSG00000145982	FARS2	"phenylalanyl-tRNA synthetase 2, mitochondrial"	0.604479	0.630769
	ENSG00000113368	LMNB1	lamin B1	0.627999	0.630477
	ENSG00000169288	MRPL1	mitochondrial ribosomal protein L1	0.613311	0.6288
	ENSG00000198324	FAM109A	family with sequence similarity 109 member A	0.510703	0.628693
	ENSG00000128581	IFT22	intraflagellar transport 22	0.595712	0.628508

	ENSG00000111906	HDDC2	HD domain containing 2	0.704719	0.628219
	ENSG00000136830	FAM129B	family with sequence similarity 129 member B	0.567133	0.628176
	ENSG00000000003	TSPAN6	tetraspanin 6	0.6999	0.627904
	ENSG00000104408	EIF3E	eukaryotic translation initiation factor 3 subunit E	0.693977	0.627349
	ENSG00000125356	NDUFA1	NADH:ubiquinone oxidoreductase subunit A1	0.481754	0.626363
	ENSG00000241343	RPL36A	ribosomal protein L36a	0.665105	0.625905
	ENSG00000101361	NOP56	NOP56 ribonucleoprotein	0.588583	0.625073
	ENSG00000100528	CNIH1	cornichon family AMPA receptor auxiliary protein 1	0.527042	0.625035
	ENSG00000106610	STAG3L4	stromal antigen 3-like 4 (pseudogene)	0.59115	0.623864
	ENSG00000165948	IFI27L1	interferon alpha inducible protein 27 like 1	0.498304	0.623056
	ENSG00000053371	AKR7A2	aldo-keto reductase family 7 member A2	0.590043	0.622283
	ENSG00000188313	PLSCR1	phospholipid scramblase 1	0.400567	0.622252
	ENSG00000187189	TSPYL4	TSPY like 4	0.461534	0.620688
	ENSG00000120662	MTRF1	mitochondrial translational release factor 1	0.447792	0.619521
	ENSG00000119335	SET	SET nuclear proto-oncogene	0.742598	0.618882
	ENSG00000169567	HINT1	histidine triad nucleotide binding protein 1	0.531404	0.618637
	ENSG00000147684	NDUFB9	NADH:ubiquinone oxidoreductase subunit B9	0.555857	0.618531
	ENSG00000143333	RGS16	regulator of G-protein signaling 16	0.542244	0.617958
	ENSG00000143420	ENSA	endosulfine alpha	0.669224	0.617762
	ENSG00000101193	GID8	GID complex subunit 8 homolog	0.555707	0.617716
	ENSG00000172315	TP53RK	TP53 regulating kinase	0.565331	0.617696
	ENSG00000174371	EXO1	exonuclease 1	0.586883	0.616257
	ENSG00000079459	FDFT1	farnesyl-diphosphate farnesyltransferase 1	0.83121	0.616238
	ENSG00000170889	RPS9	ribosomal protein S9	0.494933	0.615055
	ENSG00000254093	PINX1	"PIN2/TERF1 interacting, telomerase inhibitor 1"	0.563699	0.614464
	ENSG00000069998	CECR5	"cat eye syndrome chromosome region, candidate 5"	0.4713	0.614461
	ENSG00000161057	PSMC2	"proteasome 26S subunit, ATPase 2"	0.556815	0.614312
	ENSG00000130713	EXOSC2	exosome component 2	0.584958	0.614102
	ENSG00000152402	GUCY1A2	guanylate cyclase 1 soluble subunit alpha 2	0.645431	0.613243
	ENSG00000109084	TMEM97	transmembrane protein 97	0.833185	0.612911
	ENSG00000155755	TMEM237	transmembrane protein 237	0.646639	0.612708
	ENSG00000126088	UROD	uroporphyrinogen decarboxylase	0.562775	0.612601
	ENSG00000166598	HSP90B1	heat shock protein 90 beta family member 1	0.780005	0.61235
	ENSG00000196683	TOMM7	translocase of outer mitochondrial membrane 7	0.557692	0.611233
	ENSG00000166415	WDR72	WD repeat domain 72	0.705664	0.610389
	ENSG00000176890	TYMS	thymidylate synthetase	0.594336	0.609152
	ENSG00000116815	CD58	CD58 molecule	0.574278	0.608606
	ENSG00000118939	UCHL3	ubiquitin C-terminal hydrolase L3	0.62191	0.608182
	ENSG00000011260	UTP18	"UTP18, small subunit processome component"	0.537868	0.60794
	ENSG00000214954	LRRC69	leucine rich repeat containing 69	0.555951	0.606239
	ENSG00000170142	UBE2E1	ubiquitin conjugating enzyme E2 E1	0.619358	0.605514
	ENSG00000116459	ATP5F1	"ATP synthase, H+ transporting, mitochondrial Fo complex subunit B1"	0.570357	0.604812
	ENSG00000142676	RPL11	ribosomal protein L11	0.642594	0.604273
	ENSG00000124172	ATP5E	"ATP synthase, H+ transporting, mitochondrial F1 complex, epsilon subunit"	0.553854	0.60387
	ENSG00000122034	GTF3A	general transcription factor IIIA	0.639956	0.603777
	ENSG00000161981	SNRNP25	small nuclear ribonucleoprotein U11/U12 subunit 25	0.656031	0.603566
	ENSG00000160049	DFFA	DNA fragmentation factor subunit alpha	0.675709	0.602846
	ENSG00000159873	CCDC117	coiled-coil domain containing 117	0.801064	0.602464
	ENSG00000143315	PIGM	phosphatidylinositol glycan anchor biosynthesis class M	0.537811	0.601687
	ENSG00000169439	SDC2	syndecan 2	0.629612	0.601104
	ENSG00000004455	AK2	adenylate kinase 2	0.58514	0.599814
	ENSG00000163002	NUP35	nucleoporin 35	0.649279	0.599748
	ENSG00000008283	CYB561	cytochrome b561	0.490292	0.599664
	ENSG00000136933	RABEPK	Rab9 effector protein with kelch motifs	0.550754	0.599569
	ENSG00000116030	SUMO1	small ubiquitin-like modifier 1	0.650273	0.599114
	ENSG00000100982	PCIF1	PDX1 C-terminal inhibiting factor 1	0.398303	0.598229
	ENSG00000230989	HSBP1	heat shock factor binding protein 1	0.545827	0.597753
	ENSG00000185842	DNAH14	dynein axonemal heavy chain 14	0.576577	0.597049
	ENSG00000115216	NRBP1	nuclear receptor binding protein 1	0.590665	0.596604
	ENSG00000139197	PEX5	peroxisomal biogenesis factor 5	0.622786	0.595623
	ENSG00000135070	ISCA1	iron-sulfur cluster assembly 1	0.603147	0.595407
	ENSG00000172380	GNG12	G protein subunit gamma 12	0.792578	0.595368

	ENSG00000114446	IFT57	intraflagellar transport 57	0.619088	0.594426
	ENSG00000203760	CENPW	centromere protein W	0.460512	0.594347
	ENSG00000178307	TMEM11	transmembrane protein 11	0.395127	0.594322
	ENSG00000104979	C19orf53	chromosome 19 open reading frame 53	0.508361	0.593913
	ENSG00000117448	AKR1A1	aldo-keto reductase family 1 member A1	0.690468	0.593904
	ENSG00000187607	ZNF286A	zinc finger protein 286A	0.765241	0.59238
	ENSG00000189043	NDUFA4	"NDUFA4, mitochondrial complex associated"	0.620783	0.591739
	ENSG00000197958	RPL12	ribosomal protein L12	0.609403	0.591557
	ENSG00000198015	MRPL42	mitochondrial ribosomal protein L42	0.558932	0.591286
	ENSG00000173085	COQ2	"coenzyme Q2, polyprenyltransferase"	0.51358	0.591104
	ENSG00000186416	NKRF	NFKB repressing factor	0.562578	0.591047
	ENSG00000111843	TMEM14C	transmembrane protein 14C	0.620905	0.590898
	ENSG00000156411	C14orf2	chromosome 14 open reading frame 2	0.523541	0.590597
	ENSG00000115875	SRSF7	serine and arginine rich splicing factor 7	0.558072	0.589317
	ENSG00000172586	CHCHD1	coiled-coil-helix-coiled-coil-helix domain containing 1	0.506416	0.588876
	ENSG00000118579	MED28	mediator complex subunit 28	0.537528	0.58869
	ENSG00000151743	AMN1	antagonist of mitotic exit network 1 homolog	0.683819	0.588496
	ENSG00000117906	RCN2	reticulocalbin 2	0.698454	0.588073
	ENSG00000132646	PCNA	proliferating cell nuclear antigen	0.420561	0.587194
	ENSG00000100632	ERH	enhancer of rudimentary homolog (Drosophila)	0.573351	0.58719
	ENSG00000175193	PARL	presenilin associated rhomboid like	0.535057	0.586764
	ENSG00000145293	ENOPH1	enolase-phosphatase 1	0.606649	0.586319
	ENSG00000156110	ADK	adenosine kinase	0.60946	0.586069
	ENSG00000197756	RPL37A	ribosomal protein L37a	0.534367	0.585879
	ENSG00000156471	PTDSS1	phosphatidylserine synthase 1	0.677304	0.585521
	ENSG00000186625	KATNA1	katanin catalytic subunit A1	0.442418	0.585361
	ENSG00000143033	MTF2	metal response element binding transcription factor 2	0.620632	0.58535
	ENSG00000163634	THOC7	THO complex 7	0.662423	0.585282
	ENSG00000113269	RNF130	ring finger protein 130	0.700167	0.585128
	ENSG00000168288	MMADHC	"methylmalonic aciduria and homocystinuria, cblD type"	0.552518	0.585056
	ENSG00000183978	COA3	cytochrome c oxidase assembly factor 3	0.454443	0.58493
	ENSG00000106355	LSM5	"LSM5 homolog, U6 small nuclear RNA and mRNA degradation associated"	0.556302	0.584585
	ENSG00000256525	POLG2	"DNA polymerase gamma 2, accessory subunit"	0.496162	0.584555
	ENSG00000050405	LIMA1	LIM domain and actin binding 1	0.590325	0.584341
	ENSG00000166562	SEC11C	"SEC11 homolog C, signal peptidase complex subunit"	0.493302	0.583424
	ENSG00000179918	SEPHS2	selenophosphate synthetase 2	0.470174	0.583339
	ENSG00000168653	NDUFS5	NADH:ubiquinone oxidoreductase subunit S5	0.447218	0.58327
	ENSG00000078668	VDAC3	voltage dependent anion channel 3	0.599311	0.583252
	ENSG00000141401	IMPA2	inositol monophosphatase 2	0.564067	0.582915
	ENSG00000156469	MTERF3	mitochondrial transcription termination factor 3	0.452698	0.582238
	ENSG00000106993	CDC37L1	cell division cycle 37 like 1	0.652822	0.581541
	ENSG00000133872	SARAF	store-operated calcium entry associated regulatory factor	0.651737	0.581324
	ENSG00000254858	MPV17L2	MPV17 mitochondrial inner membrane protein like 2	0.55069	0.580507
	ENSG00000131269	ABCB7	ATP binding cassette subfamily B member 7	0.688644	0.580453
	ENSG00000110063	DCPS	"decapping enzyme, scavenger"	0.545144	0.580398
	ENSG00000265681	RPL17	ribosomal protein L17	0.608482	0.579599
	ENSG00000065911	MTHFD2	"methylenetetrahydrofolate dehydrogenase (NADP+ dependent) 2, methenyltetrahydrofolate cyclohydrolase"	0.67394	0.578727
	ENSG00000113211	PCDHB6	protocadherin beta 6	0.547292	0.578213
	ENSG00000149923	PPP4C	protein phosphatase 4 catalytic subunit	0.389742	0.577441
	ENSG00000115350	POLE4	"DNA polymerase epsilon 4, accessory subunit"	0.373808	0.576856
	ENSG00000179958	DCTPP1	dCTP pyrophosphatase 1	0.486552	0.575999
	ENSG00000169570	DTWD2	DTW domain containing 2	0.732857	0.575587
	ENSG00000172062	SMN1	"survival of motor neuron 1, telomeric"	0.581755	0.575289
	ENSG00000239779	WBP1	WW domain binding protein 1	0.580605	0.575147
	ENSG00000006831	ADIPOR2	adiponectin receptor 2	0.690701	0.574571
	ENSG00000144713	RPL32	ribosomal protein L32	0.541315	0.574202
	ENSG00000106268	NUDT1	nudix hydrolase 1	0.488716	0.574153
	ENSG00000068383	INPP5A	inositol polyphosphate-5-phosphatase A	0.67894	0.574134

	ENSG00000178035	IMPDH2	inosine monophosphate dehydrogenase 2	0.480604	0.573689
	ENSG00000249264	EEF1A1P9	eukaryotic translation elongation factor 1 alpha 1 pseudogene 9	0.580221	0.572346
	ENSG00000176273	SLC35G1	solute carrier family 35 member G1	0.497059	0.57222
	ENSG00000067064	IDI1	isopentenyl-diphosphate delta isomerase 1	0.710248	0.57221
	ENSG00000167085	PHB	prohibitin	0.558869	0.571996
	ENSG00000198860	TSEN15	tRNA splicing endonuclease subunit 15	0.543937	0.571189
	ENSG00000148334	PTGES2	prostaglandin E synthase 2	0.415851	0.570339
	ENSG00000187626	ZKSCAN4	zinc finger with KRAB and SCAN domains 4	0.541057	0.569274
	ENSG00000101182	PSMA7	proteasome subunit alpha 7	0.611977	0.569242
	ENSG00000183283	DAZAP2	DAZ associated protein 2	0.572756	0.569066
	ENSG00000091483	FH	fumarate hydratase	0.517003	0.568854
	ENSG00000011052	NME1-NME2	NME1-NME2 readthrough	0.537902	0.568051
	ENSG00000113460	BRIX1	"BRX1, biogenesis of ribosomes"	0.518963	0.56702
	ENSG00000160298	C21orf58	chromosome 21 open reading frame 58	0.574076	0.566849
	ENSG00000112306	RPS12	ribosomal protein S12	0.585658	0.565778
	ENSG00000100764	PSMC1	"proteasome 26S subunit, ATPase 1"	0.568718	0.565683
	ENSG00000136943	CTSV	cathepsin V	0.653027	0.565642
	ENSG00000130935	NOL11	nucleolar protein 11	0.584325	0.564753
	ENSG00000132423	COQ3	"coenzyme Q3, methyltransferase"	0.454187	0.564536
	ENSG00000242616	GNG10	G protein subunit gamma 10	0.677874	0.564374
	ENSG00000101444	AHCY	adenosylhomocysteinase	0.494678	0.563421
	ENSG00000164904	ALDH7A1	aldehyde dehydrogenase 7 family member A1	0.711066	0.563229
	ENSG00000162433	AK4	adenylate kinase 4	0.59315	0.563226
	ENSG00000139343	SNRPF	small nuclear ribonucleoprotein polypeptide F	0.378183	0.562532
	ENSG00000133119	RFC3	replication factor C subunit 3	0.585816	0.562186
	ENSG00000113811	SELK	selenoprotein K	0.506295	0.561729
	ENSG00000116120	FARSB	phenylalanyl-tRNA synthetase beta subunit	0.589758	0.56169
	ENSG00000117592	PRDX6	peroxiredoxin 6	0.644178	0.561437
	ENSG00000102967	DHODH	dihydroorotate dehydrogenase (quinone)	0.584332	0.561185
	ENSG00000171960	PPIH	peptidylprolyl isomerase H	0.40786	0.56116
	ENSG00000128039	SRD5A3	steroid 5 alpha-reductase 3	0.53407	0.56115
	ENSG00000132950	ZMYM5	zinc finger MYM-type containing 5	0.458967	0.56089
	ENSG00000110700	RPS13	ribosomal protein S13	0.506	0.560536
	ENSG00000141101	NOB1	NIN1/PSMD8 binding protein 1 homolog	0.454083	0.560442
	ENSG00000161970	RPL26	ribosomal protein L26	0.635677	0.560063
	ENSG00000198830	HMGN2	high mobility group nucleosomal binding domain 2	0.431841	0.559308
	ENSG00000169714	CNBP	CCHC-type zinc finger nucleic acid binding protein	0.47528	0.558615
	ENSG00000138641	HERC3	HECT and RLD domain containing E3 ubiquitin protein ligase 3	0.458215	0.557969
	ENSG00000198176	TFDP1	transcription factor Dp-1	0.598627	0.557178
	ENSG00000188529	SRSF10	serine and arginine rich splicing factor 10	0.581423	0.557139
	ENSG00000169174	PCSK9	proprotein convertase subtilisin/kexin type 9	0.536665	0.556471
	ENSG00000158470	B4GALT5	"beta-1,4-galactosyltransferase 5"	0.648518	0.556421
	ENSG00000147400	CETN2	centrin 2	0.67484	0.556184
	ENSG00000141543	EIF4A3	eukaryotic translation initiation factor 4A3	0.444924	0.555671
	ENSG00000104884	ERCC2	"ERCC excision repair 2, TFIIH core complex helicase subunit"	0.760713	0.555621
	ENSG00000108298	RPL19	ribosomal protein L19	0.501585	0.555254
	ENSG00000071082	RPL31	ribosomal protein L31	0.500837	0.553839
	ENSG00000103035	PSMD7	"proteasome 26S subunit, non-ATPase 7"	0.485609	0.55362
	ENSG00000100216	TOMM22	translocase of outer mitochondrial membrane 22	0.494304	0.551779
	ENSG00000114520	SNX4	sorting nexin 4	0.537189	0.550717
	ENSG00000100372	SLC25A17	solute carrier family 25 member 17	0.567036	0.550626
	ENSG00000152147	GEMIN6	gem nuclear organelle associated protein 6	0.527315	0.549781
	ENSG00000004142	POLDIP2	DNA polymerase delta interacting protein 2	0.545445	0.547899
	ENSG00000143384	MCL1	BCL2 family apoptosis regulator	0.514158	0.547684
	ENSG00000100442	FKBP3	FK506 binding protein 3	0.598451	0.547113
	ENSG00000183207	RUVBL2	RuvB like AAA ATPase 2	0.370826	0.545808
	ENSG00000126602	TRAP1	TNF receptor associated protein 1	0.620316	0.54511
	ENSG00000138439	FAM117B	family with sequence similarity 117 member B	0.800434	0.544344
	ENSG00000011258	MBTD1	mbt domain containing 1	0.65506	0.544341
	ENSG00000119408	NEK6	NIMA related kinase 6	0.713992	0.544106
	ENSG00000141429	GALNT1	polypeptide N-acetylgalactosaminyltransferase 1	0.673639	0.543714
	ENSG00000174748	RPL15	ribosomal protein L15	0.477561	0.543018
	ENSG00000165672	PRDX3	peroxiredoxin 3	0.627377	0.542784

	ENSG00000149196	HIKESHI	"Hikeshi, heat shock protein nuclear import factor"	0.467457	0.541752
	ENSG00000018699	TTC27	tetratricopeptide repeat domain 27	0.506052	0.541612
	ENSG00000223768	LINC00205	long intergenic non-protein coding RNA 205	0.510607	0.541599
	ENSG00000162694	EXTL2	exostosin like glycosyltransferase 2	0.709238	0.541277
	ENSG00000072062	PRKACA	protein kinase cAMP-activated catalytic subunit alpha	0.628271	0.54078
	ENSG00000162063	CCNF	cyclin F	0.4926	0.539965
	ENSG00000135373	EHF	ETS homologous factor	0.793912	0.539603
	ENSG00000151287	TEX30	testis expressed 30	0.591534	0.539069
	ENSG00000120526	NUDCD1	NudC domain containing 1	0.605891	0.538786
	ENSG00000139684	ESD	esterase D	0.528035	0.538701
	ENSG00000151465	CDC123	cell division cycle 123	0.579892	0.537706
	ENSG00000157978	LDLRAP1	low density lipoprotein receptor adaptor protein 1	0.541318	0.537152
	ENSG00000140612	SEC11A	"SEC11 homolog A, signal peptidase complex subunit"	0.489579	0.537134
	ENSG00000108468	CBX1	chromobox 1	0.608134	0.536891
	ENSG00000164253	WDR41	WD repeat domain 41	0.607608	0.536312
	ENSG00000143314	MRPL24	mitochondrial ribosomal protein L24	0.506235	0.53577
	ENSG00000205981	DNAJC19	DnaJ heat shock protein family (Hsp40) member C19	0.487561	0.535434
	ENSG00000142657	PGD	phosphogluconate dehydrogenase	0.551699	0.534879
	ENSG00000163832	ELP6	elongator acetyltransferase complex subunit 6	0.412246	0.534597
	ENSG00000166851	PLK1	polo like kinase 1	0.502805	0.534145
	ENSG00000100294	MCAT	malonyl-CoA-acyl carrier protein transacylase	0.383907	0.533893
	ENSG00000171202	TMEM126A	transmembrane protein 126A	0.457839	0.533496
	ENSG00000008988	RPS20	ribosomal protein S20	0.495754	0.533154
	ENSG00000155858	LSM11	"LSM11, U7 small nuclear RNA associated"	0.573773	0.53311
	ENSG00000106803	SEC61B	Sec61 translocon beta subunit	0.380985	0.532805
	ENSG00000092010	PSME1	proteasome activator subunit 1	0.489988	0.532391
	ENSG00000023572	GLRX2	glutaredoxin 2	0.493896	0.530772
	ENSG00000138028	CGREF1	cell growth regulator with EF-hand domain 1	0.532261	0.530638
	ENSG00000108947	EFNB3	ephrin B3	0.713137	0.530624
	ENSG00000164919	COX6C	cytochrome c oxidase subunit 6C	0.473092	0.529371
	ENSG00000115944	COX7A2L	cytochrome c oxidase subunit 7A2 like	0.583686	0.527884
	ENSG00000100504	PYGL	"phosphorylase, glycogen, liver"	0.598803	0.527726
	ENSG00000091651	ORC6	origin recognition complex subunit 6	0.500223	0.527708
	ENSG00000140374	ETFA	electron transfer flavoprotein alpha subunit	0.591116	0.527411
	ENSG00000135624	CCT7	chaperonin containing TCP1 subunit 7	0.61167	0.525603
	ENSG00000101400	SNTA1	syntrophin alpha 1	0.470681	0.524896
	ENSG00000186660	ZFP91	ZFP91 zinc finger protein	0.636921	0.52485
	ENSG00000102178	UBL4A	ubiquitin like 4A	0.412535	0.524187
	ENSG00000164091	WDR82	WD repeat domain 82	0.498426	0.52336
	ENSG00000138175	ARL3	ADP ribosylation factor like GTPase 3	0.606915	0.522059
	ENSG00000224531	SMIM13	small integral membrane protein 13	0.483039	0.520542
	ENSG00000082515	MRPL22	mitochondrial ribosomal protein L22	0.41355	0.520083
	ENSG00000197977	ELOVL2	ELOVL fatty acid elongase 2	0.649693	0.519209
	ENSG00000168795	ZBTB5	zinc finger and BTB domain containing 5	0.434602	0.518307
	ENSG00000163528	CHCHD4	coiled-coil-helix-coiled-coil-helix domain containing 4	0.454512	0.51827
	ENSG00000139180	NDUFA9	NADH:ubiquinone oxidoreductase subunit A9	0.482408	0.51758
	ENSG00000116288	PARK7	Parkinsonism associated deglycase	0.541766	0.515431
	ENSG00000183077	AFMID	arylformamidase	0.518471	0.514579
	ENSG00000130560	UBAC1	UBA domain containing 1	0.517836	0.514537
	ENSG00000107262	BAG1	BCL2 associated athanogene 1	0.383929	0.514251
	ENSG00000156482	RPL30	ribosomal protein L30	0.483029	0.514125
	ENSG00000060762	MPC1	mitochondrial pyruvate carrier 1	0.595465	0.512531
	ENSG00000135446	CDK4	cyclin dependent kinase 4	0.466874	0.512105
	ENSG00000171314	PGAM1	phosphoglycerate mutase 1	0.452985	0.512096
	ENSG00000143742	SRP9	signal recognition particle 9	0.611286	0.511968
	ENSG00000113845	TIMMDC1	translocase of inner mitochondrial membrane domain containing 1	0.470364	0.511371
	ENSG00000168701	TMEM208	transmembrane protein 208	0.434963	0.511256
	ENSG00000141759	TXNL4A	thioredoxin like 4A	0.436729	0.51071
	ENSG00000124767	GLO1	glyoxalase I	0.596599	0.510487
	ENSG00000065978	YBX1	Y-box binding protein 1	0.608175	0.510208
	ENSG00000101190	TCFL5	transcription factor like 5	0.531116	0.509004
	ENSG00000171490	RSL1D1	ribosomal L1 domain containing 1	0.599977	0.50853

	ENSG00000176788	BASP1	brain abundant membrane attached signal protein 1	0.483084	0.508448
	ENSG00000132541	RIDA	reactive intermediate imine deaminase A homolog	0.692374	0.508209
	ENSG00000112699	GMDS	"GDP-mannose 4,6-dehydratase"	0.567183	0.508126
	ENSG00000014641	MDH1	malate dehydrogenase 1	0.483648	0.507619
	ENSG00000159377	PSMB4	proteasome subunit beta 4	0.447662	0.505157
	ENSG00000197006	METTL9	methyltransferase like 9	0.619183	0.505024
	ENSG00000127184	COX7C	cytochrome c oxidase subunit 7C	0.420544	0.504002
	ENSG00000178741	COX5A	cytochrome c oxidase subunit 5A	0.440628	0.503475
	ENSG00000115368	WDR75	WD repeat domain 75	0.497876	0.500219
	ENSG00000089220	PEBP1	phosphatidylethanolamine binding protein 1	0.5634	0.499523
	ENSG00000101935	AMMECR1	"Alport syndrome, mental retardation, midface hypoplasia and elliptocytosis chromosomal region gene 1"	0.486683	0.499441
	ENSG00000118600	TMEM5	transmembrane protein 5	0.426584	0.498614
	ENSG00000196363	WDR5	WD repeat domain 5	0.458789	0.498514
	ENSG00000171155	C1GALT1C1	C1GALT1 specific chaperone 1	0.504905	0.496875
	ENSG00000142534	RPS11	ribosomal protein S11	0.516476	0.496856
	ENSG00000188342	GTF2F2	general transcription factor IIF subunit 2	0.413409	0.496262
	ENSG00000176244	ACBD7	acyl-CoA binding domain containing 7	0.700393	0.496153
	ENSG00000103502	CDIPT	CDP-diacylglycerol--inositol 3-phosphatidyltransferase	0.50783	0.492623
	ENSG00000167977	KCTD5	potassium channel tetramerization domain containing 5	0.425124	0.492212
	ENSG00000123349	PFDN5	prefoldin subunit 5	0.546246	0.491788
	ENSG00000137106	GRHPR	glyoxylate and hydroxypyruvate reductase	0.445495	0.490305
	ENSG00000158089	GALNT14	polypeptide N-acetylgalactosaminyltransferase 14	0.608762	0.488799
	ENSG00000134291	TMEM106C	transmembrane protein 106C	0.585347	0.488068
	ENSG00000136574	GATA4	GATA binding protein 4	0.816668	0.487858
	ENSG00000145741	BTF3	basic transcription factor 3	0.506364	0.487342
	ENSG00000103429	BFAR	bifunctional apoptosis regulator	0.519609	0.486401
	ENSG00000160014	CALM3	calmodulin 3	0.570431	0.485807
	ENSG00000127955	GNAI1	G protein subunit alpha i1	0.590945	0.485692
	ENSG00000100784	RPS6KA5	ribosomal protein S6 kinase A5	0.591567	0.484197
	ENSG00000148303	RPL7A	ribosomal protein L7a	0.525127	0.483312
	ENSG00000100558	PLEK2	pleckstrin 2	0.612746	0.482887
	ENSG00000161016	RPL8	ribosomal protein L8	0.447702	0.482796
	ENSG00000115365	LANCL1	LanC like 1	0.596777	0.482519
	ENSG00000133773	CCDC59	coiled-coil domain containing 59	0.410004	0.482381
	ENSG00000125863	MKKS	McKusick-Kaufman syndrome	0.509373	0.481629
	ENSG00000164815	ORC5	origin recognition complex subunit 5	0.451018	0.481556
	ENSG00000145050	MANF	mesencephalic astrocyte derived neurotrophic factor	0.454652	0.481426
	ENSG00000136158	SPRY2	sprouty RTK signaling antagonist 2	0.550961	0.481338
	ENSG00000140391	TSPAN3	tetraspanin 3	0.452084	0.480864
	ENSG00000128050	PAICS	phosphoribosylaminoimidazole carboxylase; phosphoribosylaminoimidazolesuccinocarboxamide synthase	0.627836	0.480478
	ENSG00000170632	ARMC10	armadillo repeat containing 10	0.678404	0.480358
	ENSG00000181610	MRPS23	mitochondrial ribosomal protein S23	0.403958	0.480255
	ENSG00000178988	MRFAP1L1	Morf4 family associated protein 1 like 1	0.361048	0.479952
	ENSG00000228409	CCT6P1	chaperonin containing TCP1 subunit 6 pseudogene 1	0.440193	0.479515
	ENSG00000079785	DDX1	DEAD/H-box helicase 1	0.493408	0.479488
	ENSG00000112081	SRSF3	serine and arginine rich splicing factor 3	0.459896	0.479402
	ENSG00000065621	GSTO2	glutathione S-transferase omega 2	0.4087	0.479226
	ENSG00000136682	CBWD2	COBW domain containing 2	0.552169	0.479109
	ENSG00000026297	RNASET2	ribonuclease T2	0.439969	0.479086
	ENSG00000103876	FAH	fumarylacetoacetate hydrolase	0.553601	0.478695
	ENSG00000166295	ANAPC16	anaphase promoting complex subunit 16	0.486764	0.477979
	ENSG00000124787	RPP40	ribonuclease P/MRP subunit p40	0.442086	0.477138
	ENSG00000245694	CRNDE	colorectal neoplasia differentially expressed (non-protein coding)	0.437687	0.476426
	ENSG00000180389	ATP5EP2	"ATP synthase, H+ transporting, mitochondrial F1 complex, epsilon subunit pseudogene 2"	1.06228	0.475944
	ENSG00000129518	EAPP	E2F associated phosphoprotein	0.426617	0.475068
	ENSG00000138777	PPA2	pyrophosphatase (inorganic) 2	0.417186	0.475001

	ENSG00000055044	NOP58	NOP58 ribonucleoprotein	0.57573	0.474755
	ENSG00000147604	RPL7	ribosomal protein L7	0.540895	0.474478
	ENSG00000074800	ENO1	enolase 1	0.546022	0.474453
	ENSG00000169230	PRELID1	PRELI domain containing 1	0.526265	0.474331
	ENSG00000204628	RACK1	receptor for activated C kinase 1	0.479845	0.474264
	ENSG00000159593	NAE1	NEDD8 activating enzyme E1 subunit 1	0.493453	0.473918
	ENSG00000086061	DNAJA1	DnaJ heat shock protein family (Hsp40) member A1	0.475614	0.472945
	ENSG00000161904	LEMD2	LEM domain containing 2	0.483313	0.472819
	ENSG00000159055	MIS18A	MIS18 kinetochore protein A	0.390648	0.472706
	ENSG00000119718	EIF2B2	eukaryotic translation initiation factor 2B subunit beta	0.438912	0.472013
	ENSG00000198356	ASNA1	"arsA arsenite transporter, ATP-binding, homolog 1 (bacterial)"	0.380803	0.471845
	ENSG00000119778	ATAD2B	"ATPase family, AAA domain containing 2B"	0.623753	0.47118
	ENSG00000049541	RFC2	replication factor C subunit 2	0.554049	0.470145
	ENSG00000115484	CCT4	chaperonin containing TCP1 subunit 4	0.507679	0.468577
	ENSG00000169241	SLC50A1	solute carrier family 50 member 1	0.490646	0.467501
	ENSG00000161960	EIF4A1	eukaryotic translation initiation factor 4A1	0.506091	0.467426
	ENSG00000196154	S100A4	S100 calcium binding protein A4	0.677395	0.465981
	ENSG00000172172	MRPL13	mitochondrial ribosomal protein L13	0.515309	0.464846
	ENSG00000182054	IDH2	"isocitrate dehydrogenase (NADP(+)) 2, mitochondrial"	0.358842	0.464326
	ENSG00000163017	ACTG2	"actin, gamma 2, smooth muscle, enteric"	0.411173	0.462855
	ENSG00000147180	ZNF711	zinc finger protein 711	0.597437	0.462778
	ENSG00000124535	WRNIP1	Werner helicase interacting protein 1	0.463639	0.461986
	ENSG00000153989	NUS1	NUS1 dehydrodolichyl diphosphate synthase subunit	0.588642	0.461185
	ENSG00000189334	S100A14	S100 calcium binding protein A14	0.526757	0.460932
	ENSG00000228474	OST4	"oligosaccharyltransferase complex subunit 4, non-catalytic"	0.446121	0.460205
	ENSG00000163541	SUCLG1	succinate-CoA ligase alpha subunit	0.393309	0.459611
	ENSG00000090263	MRPS33	mitochondrial ribosomal protein S33	0.401031	0.459406
	ENSG00000139826	ABHD13	abhydrolase domain containing 13	0.488825	0.459061
	ENSG00000089053	ANAPC5	anaphase promoting complex subunit 5	0.562191	0.456977
	ENSG00000073712	FERMT2	fermitin family member 2	0.511322	0.456936
	ENSG00000156261	CCT8	chaperonin containing TCP1 subunit 8	0.516095	0.456799
	ENSG00000112062	MAPK14	mitogen-activated protein kinase 14	0.578104	0.456541
	ENSG00000124098	FAM210B	family with sequence similarity 210 member B	0.494677	0.455537
	ENSG00000165775	FUNDC2	FUN14 domain containing 2	0.465033	0.455508
	ENSG00000139800	ZIC5	Zic family member 5	0.519752	0.454947
	ENSG00000177954	RPS27	ribosomal protein S27	0.508871	0.454789
	ENSG00000008018	PSMB1	proteasome subunit beta 1	0.365698	0.454699
	ENSG00000105248	CCDC94	coiled-coil domain containing 94	0.459384	0.454246
	ENSG00000101391	CDK5RAP1	CDK5 regulatory subunit associated protein 1	0.421749	0.452614
	ENSG00000168118	RAB4A	"RAB4A, member RAS oncogene family"	0.553993	0.451898
	ENSG00000001497	LAS1L	"LAS1 like, ribosome biogenesis factor"	0.473209	0.451075
	ENSG00000178952	TUFM	"Tu translation elongation factor, mitochondrial"	0.445132	0.450125
	ENSG00000144746	ARL6IP5	ADP ribosylation factor like GTPase 6 interacting protein 5	0.510344	0.450079
	ENSG00000168710	AHCYL1	adenosylhomocysteinase like 1	0.599927	0.449753
	ENSG00000198258	UBL5	ubiquitin like 5	0.372425	0.448646
	ENSG00000112592	TBP	TATA-box binding protein	0.439215	0.44549
	ENSG00000174669	SLC29A2	solute carrier family 29 member 2	0.357566	0.444176
	ENSG00000104723	TUSC3	tumor suppressor candidate 3	0.476439	0.443338
	ENSG00000146733	PSPH	phosphoserine phosphatase	0.415752	0.442755
	ENSG00000101347	SAMHD1	SAM and HD domain containing deoxynucleoside triphosphate triphosphohydrolase 1	0.422929	0.441313
	ENSG00000182890	GLUD2	glutamate dehydrogenase 2	0.54971	0.439276
	ENSG00000044574	HSPA5	heat shock protein family A (Hsp70) member 5	0.461655	0.437272
	ENSG00000072506	HSD17B10	hydroxysteroid 17-beta dehydrogenase 10	0.404017	0.437113
	ENSG00000065154	OAT	ornithine aminotransferase	0.61619	0.435381
	ENSG00000165943	MOAP1	modulator of apoptosis 1	0.358154	0.435341
	ENSG00000111802	TDP2	tyrosyl-DNA phosphodiesterase 2	0.488266	0.43491
	ENSG00000112078	KCTD20	potassium channel tetramerization domain containing 20	0.59754	0.434534
	ENSG00000127884	ECHS1	"enoyl-CoA hydratase, short chain 1"	0.423954	0.434189

	ENSG00000154582	TCEB1	transcription elongation factor B subunit 1	0.384157	0.433829
	ENSG00000133112	TPT1	"tumor protein, translationally-controlled 1"	0.512395	0.431892
	ENSG00000196262	PPIA	peptidylprolyl isomerase A	0.404212	0.429638
	ENSG00000179399	GPC5	glypican 5	0.529826	0.4271
	ENSG00000188211	NCR3LG1	natural killer cell cytotoxicity receptor 3 ligand 1	0.504336	0.427074
	ENSG00000139990	DCAF5	DDB1 and CUL4 associated factor 5	0.49886	0.426772
	ENSG00000131778	CHD1L	chromodomain helicase DNA binding protein 1 like	0.459906	0.425305
	ENSG00000104047	DTWD1	DTW domain containing 1	0.501435	0.424841
	ENSG00000132963	POMP	proteasome maturation protein	0.398388	0.424103
	ENSG00000129562	DAD1	defender against cell death 1	0.483024	0.423811
	ENSG00000092199	HNRNPC	heterogeneous nuclear ribonucleoprotein C (C1/C2)	0.47602	0.42311
	ENSG00000117118	SDHB	succinate dehydrogenase complex iron sulfur subunit B	0.436855	0.422219
	ENSG00000143870	PDIA6	protein disulfide isomerase family A member 6	0.540674	0.422012
	ENSG00000146433	TMEM181	transmembrane protein 181	0.528742	0.421469
	ENSG00000197771	MCMBP	minichromosome maintenance complex binding protein	0.539588	0.420955
	ENSG00000108384	RAD51C	RAD51 paralog C	0.445142	0.420747
	ENSG00000120696	KBTBD7	kelch repeat and BTB domain containing 7	0.400185	0.420395
	ENSG00000148834	GSTO1	glutathione S-transferase omega 1	0.382099	0.419248
	ENSG00000173145	NOC3L	NOC3 like DNA replication regulator	0.463655	0.418221
	ENSG00000154978	VOPP1	"vesicular, overexpressed in cancer, prosurvival protein 1"	0.46844	0.41793
	ENSG00000175691	ZNF77	zinc finger protein 77	0.396748	0.416536
	ENSG00000128463	EMC4	ER membrane protein complex subunit 4	0.519872	0.416206
	ENSG00000164930	FZD6	frizzled class receptor 6	0.513988	0.416162
	ENSG00000129103	SUMF2	sulfatase modifying factor 2	0.425012	0.415504
	ENSG00000058804	NDC1	NDC1 transmembrane nucleoporin	0.581386	0.415238
	ENSG00000152234	ATP5A1	"ATP synthase, H+ transporting, mitochondrial F1 complex, alpha subunit 1, cardiac muscle"	0.424851	0.412725
	ENSG00000182768	NGRN	"neugrin, neurite outgrowth associated"	0.449335	0.411608
	ENSG00000115234	SNX17	sorting nexin 17	0.352968	0.410153
	ENSG00000074219	TEAD2	TEA domain transcription factor 2	0.477682	0.408097
	ENSG00000148019	CEP78	centrosomal protein 78	0.579872	0.407027
	ENSG00000124570	SERPINB6	serpin family B member 6	0.400069	0.406089
	ENSG00000107874	CUEDC2	CUE domain containing 2	0.421961	0.40473
	ENSG00000156709	AIFM1	"apoptosis inducing factor, mitochondria associated 1"	0.517603	0.400808
	ENSG00000173281	PPP1R3B	protein phosphatase 1 regulatory subunit 3B	0.433307	0.398739
	ENSG00000122042	UBL3	ubiquitin like 3	0.578444	0.397981
	ENSG00000135845	PIGC	phosphatidylinositol glycan anchor biosynthesis class C	0.41005	0.397585
	ENSG00000131370	SH3BP5	SH3 domain binding protein 5	0.463129	0.394409
	ENSG00000181751	C5orf30	chromosome 5 open reading frame 30	0.465594	0.3944
	ENSG00000254772	EEF1G	eukaryotic translation elongation factor 1 gamma	0.50351	0.394335
	ENSG00000141698	NT5C3B	"5'-nucleotidase, cytosolic IIIB"	0.390653	0.394116
	ENSG00000140259	MFAP1	microfibrillar associated protein 1	0.40971	0.392112
	ENSG00000187514	PTMA	"prothymosin, alpha"	0.472511	0.39151
	ENSG00000163902	RPN1	ribophorin I	0.436504	0.390911
	ENSG00000164332	UBLCP1	ubiquitin like domain containing CTD phosphatase 1	0.457536	0.390483
	ENSG00000115241	PPM1G	"protein phosphatase, Mg2+/Mn2+ dependent 1G"	0.421925	0.389986
	ENSG00000170185	USP38	ubiquitin specific peptidase 38	0.48234	0.389678
	ENSG00000100271	TTLL1	tubulin tyrosine ligase like 1	0.686081	0.387382
	ENSG00000134716	CYP2J2	cytochrome P450 family 2 subfamily J member 2	0.405508	0.386161
	ENSG00000136156	ITM2B	integral membrane protein 2B	0.455403	0.385767
	ENSG00000164626	KCNK5	potassium two pore domain channel subfamily K member 5	0.424791	0.385622
	ENSG00000116679	IVNS1ABP	influenza virus NS1A binding protein	0.500592	0.379788
	ENSG00000175785	PRIMA1	proline rich membrane anchor 1	0.382483	0.378951
	ENSG00000101544	ADNP2	ADNP homeobox 2	0.389951	0.375037
	ENSG00000160124	CCDC58	coiled-coil domain containing 58	0.382227	0.374341
	ENSG00000196584	XRCC2	X-ray repair cross complementing 2	0.497408	0.37365
	ENSG00000196659	TTC30B	tetratricopeptide repeat domain 30B	0.431373	0.372918
	ENSG00000081870	HSPB11	heat shock protein family B (small) member 11	0.468481	0.371903
	ENSG00000084623	EIF3I	eukaryotic translation initiation factor 3 subunit I	0.502812	0.370749

	ENSG00000188021	UBQLN2	ubiquilin 2	0.454439	0.36814
	ENSG00000125821	DTD1	D-tyrosyl-tRNA deacylase 1	0.439774	0.362775
	ENSG00000016391	CHDH	choline dehydrogenase	0.460734	0.362754
	ENSG00000137054	POLR1E	RNA polymerase I subunit E	0.416051	0.360962
	ENSG00000147669	POLR2K	RNA polymerase II subunit K	0.36188	0.359019
	ENSG00000163428	LRRC58	leucine rich repeat containing 58	0.529406	0.357706
	ENSG00000095002	MSH2	mutS homolog 2	0.453754	0.355227
	ENSG00000087302	C14orf166	chromosome 14 open reading frame 166	0.363788	0.346326
	ENSG00000115128	SF3B6	splicing factor 3b subunit 6	0.369752	0.345683

Supplementary Table 2C: miR-29a unique targets in LCC9 cells

				LCC9_Pre-miR-29a vs Anti-miR-29a
	**Input IDs**	**Gene Symbol**	**Gene Name**	**log2(fold_change)**
1.	ENSG00000089127	OAS1	2'-5'-oligoadenylate synthetase 1	1.00467
2.	ENSG00000174137	FAM53A	family with sequence similarity 53 member A	0.857463
3.	ENSG00000183513	COA5	cytochrome c oxidase assembly factor 5	0.542847
4.	ENSG00000119688	ABCD4	ATP binding cassette subfamily D member 4	0.487976
5.	ENSG00000118777	ABCG2	ATP binding cassette subfamily G member 2 (Junior blood group)	1.55682
6.	ENSG00000162482	AKR7A3	aldo-keto reductase family 7 member A3	0.843423
7.	ENSG00000137124	ALDH1B1	aldehyde dehydrogenase 1 family member B1	0.441378
8.	ENSG00000119523	ALG2	"ALG2, alpha-1,3/1,6-mannosyltransferase"	0.343699
9.	ENSG00000176248	ANAPC2	anaphase promoting complex subunit 2	0.69434
10.	ENSG00000213337	ANKRD39	ankyrin repeat domain 39	0.790548
11.	ENSG00000186106	ANKRD46	ankyrin repeat domain 46	0.550979
12.	ENSG00000214293	APTR	Alu-mediated CDKN1A/p21 transcriptional regulator (non-protein coding)	0.407018
13.	ENSG00000102030	NAA10	"N(alpha)-acetyltransferase 10, NatA catalytic subunit"	0.547257
14.	ENSG00000182196	ARL6IP4	ADP ribosylation factor like GTPase 6 interacting protein 4	0.631648
15.	ENSG00000136950	ARPC5L	actin related protein 2/3 complex subunit 5 like	0.352874
16.	ENSG00000126756	UXT	ubiquitously expressed prefoldin like chaperone	0.423882
17.	ENSG00000130768	SMPDL3B	sphingomyelin phosphodiesterase acid like 3B	0.508441
18.	ENSG00000128272	ATF4	activating transcription factor 4	0.456274
19.	ENSG00000152234	ATP5A1	"ATP synthase, H+ transporting, mitochondrial F1 complex, alpha subunit 1, cardiac muscle"	0.412725
20.	ENSG00000110955	ATP5B	"ATP synthase, H+ transporting, mitochondrial F1 complex, beta polypeptide"	0.734603
21.	ENSG00000099624	ATP5D	"ATP synthase, H+ transporting, mitochondrial F1 complex, delta subunit"	0.575794
22.	ENSG00000124172	ATP5E	"ATP synthase, H+ transporting, mitochondrial F1 complex, epsilon subunit"	0.60387
23.	ENSG00000165629	ATP5C1	"ATP synthase, H+ transporting, mitochondrial F1 complex, gamma polypeptide 1"	1.34628
24.	ENSG00000128524	ATP6V1F	ATPase H+ transporting V1 subunit F	0.541429
25.	ENSG00000130770	ATPIF1	ATPase inhibitory factor 1	0.792053
26.	ENSG00000148090	AUH	AU RNA binding methylglutaconyl-CoA hydratase	0.583462
27.	ENSG00000198563	DDX39B	DEAD-box helicase 39B	0.506986
28.	ENSG00000054116	TRAPPC3	trafficking protein particle complex 3	0.455165
29.	ENSG00000163170	BOLA3	bolA family member 3	0.453897
30.	ENSG00000130303	BST2	bone marrow stromal cell antigen 2	0.503736
31.	ENSG00000078401	EDN1	endothelin 1	0.605404

32.	ENSG00000100290	BIK	BCL2 interacting killer	0.434716
33.	ENSG00000165233	CARD19	caspase recruitment domain family member 19	0.680634
34.	ENSG00000112578	BYSL	bystin like	0.470095
35.	ENSG00000111678	C12orf57	chromosome 12 open reading frame 57	0.421145
36.	ENSG00000171224	C10orf35	chromosome 10 open reading frame 35	0.354218
37.	ENSG00000171067	C11orf24	chromosome 11 open reading frame 24	0.643369
38.	ENSG00000139438	FAM222A	family with sequence similarity 222 member A	0.560663
39.	ENSG00000151131	C12orf45	chromosome 12 open reading frame 45	0.721034
40.	ENSG00000129480	DTD2	D-tyrosyl-tRNA deacylase 2 (putative)	0.712165
41.	ENSG00000134153	EMC7	ER membrane protein complex subunit 7	0.399016
42.	ENSG00000130731	METTL26	methyltransferase like 26	0.411608
43.	ENSG00000124074	ENKD1	enkurin domain containing 1	1.20299
44.	ENSG00000182831	C16orf72	chromosome 16 open reading frame 72	0.518243
45.	ENSG00000175643	RMI2	RecQ mediated genome instability 2	0.359454
46.	ENSG00000074842	MYDGF	myeloid derived growth factor	0.462685
47.	ENSG00000197223	C1D	C1D nuclear receptor corepressor	0.440034
48.	ENSG00000057757	PITHD1	PITH domain containing 1	0.387639
49.	ENSG00000198912	C1orf174	chromosome 1 open reading frame 174	0.358429
50.	ENSG00000163866	SMIM12	small integral membrane protein 12	0.503397
51.	ENSG00000168275	COA6	cytochrome c oxidase assembly factor 6	0.436718
52.	ENSG00000203724	C1orf53	chromosome 1 open reading frame 53	0.567423
53.	ENSG00000162757	C1orf74	chromosome 1 open reading frame 74	0.715414
54.	ENSG00000116205	TCEANC2	transcription elongation factor A N-terminal and central domain containing 2	0.580425
55.	ENSG00000101220	C20orf27	chromosome 20 open reading frame 27	0.377463
56.	ENSG00000228314	CYP4F29P	"cytochrome P450 family 4 subfamily F member 29, pseudogene"	0.676683
57.	ENSG00000159079	C21orf59	chromosome 21 open reading frame 59	0.777816
58.	ENSG00000160256	FAM207A	family with sequence similarity 207 member A	0.405904
59.	ENSG00000250486	FAM218A	family with sequence similarity 218 member A	0.541897
60.	ENSG00000243449	C4orf48	chromosome 4 open reading frame 48	0.518415
61.	ENSG00000134830	C5AR2	complement component 5a receptor 2	0.41513
62.	ENSG00000151881	TMEM267	transmembrane protein 267	0.504625
63.	ENSG00000124541	RRP36	ribosomal RNA processing 36	0.454369
64.	ENSG00000204387	C6orf48	chromosome 6 open reading frame 48	0.389673
65.	ENSG00000232956	SNHG15	small nucleolar RNA host gene 15	0.773507
66.	ENSG00000146540	C7orf50	chromosome 7 open reading frame 50	0.642142
67.	ENSG00000188186	LAMTOR4	"late endosomal/lysosomal adaptor, MAPK and MTOR activator 4"	0.414919

68.	ENSG00000130193	THEM6	thioesterase superfamily member 6	0.420549
69.	ENSG00000148362	C9orf142	chromosome 9 open reading frame 142	0.387802
70.	ENSG00000163050	COQ8A	coenzyme Q8A	0.690812
71.	ENSG00000007080	CCDC124	coiled-coil domain containing 124	0.361384
72.	ENSG00000221978	CCNL2	cyclin L2	0.472603
73.	ENSG00000156345	CDK20	cyclin dependent kinase 20	0.448019
74.	ENSG00000184990	SIVA1	SIVA1 apoptosis inducing factor	0.601179
75.	ENSG00000123374	CDK2	cyclin dependent kinase 2	0.562828
76.	ENSG00000125817	CENPB	centromere protein B	0.337413
77.	ENSG00000100162	CENPM	centromere protein M	0.370338
78.	ENSG00000179604	CDC42EP4	CDC42 effector protein 4	0.416077
79.	ENSG00000166595	FAM96B	family with sequence similarity 96 member B	0.373062
80.	ENSG00000159685	CHCHD6	coiled-coil-helix-coiled-coil-helix domain containing 6	0.469047
81.	ENSG00000179271	GADD45GIP1	GADD45G interacting protein 1	0.427637
82.	ENSG00000122705	CLTA	clathrin light chain A	0.385781
83.	ENSG00000143771	CNIH4	cornichon family AMPA receptor auxiliary protein 4	0.421596
84.	ENSG00000188986	NELFB	negative elongation factor complex member B	0.363338
85.	ENSG00000135775	COG2	component of oligomeric golgi complex 2	0.403504
86.	ENSG00000170619	COMMD5	COMM domain containing 5	0.655131
87.	ENSG00000168090	COPS6	COP9 signalosome subunit 6	0.443229
88.	ENSG00000088682	COQ9	coenzyme Q9	0.821226
89.	ENSG00000131143	COX4I1	cytochrome c oxidase subunit 4I1	0.448878
90.	ENSG00000176340	COX8A	cytochrome c oxidase subunit 8A	0.517129
91.	ENSG00000112695	COX7A2	cytochrome c oxidase subunit 7A2	0.459814
92.	ENSG00000111775	COX6A1	cytochrome c oxidase subunit 6A1	0.508716
93.	ENSG00000135940	COX5B	cytochrome c oxidase subunit 5B	0.46617
94.	ENSG00000184164	CRELD2	cysteine rich with EGF like domains 2	0.52218
95.	ENSG00000233382	NKAPP1	NFKB activating protein pseudogene 1	1.42496
96.	ENSG00000179091	CYC1	cytochrome c1	0.397388
97.	ENSG00000140465	CYP1A1	cytochrome P450 family 1 subfamily A member 1	0.685945
98.	ENSG00000058404	CAMK2B	calcium/calmodulin dependent protein kinase II beta	0.714147
99.	ENSG00000004948	CALCR	calcitonin receptor	0.620437
100.	ENSG00000186222	BLOC1S4	biogenesis of lysosomal organelles complex 1 subunit 4	0.568415
101.	ENSG00000144021	CIAO1	cytosolic iron-sulfur assembly component 1	0.377027
102.	ENSG00000165215	CLDN3	claudin 3	0.476786
103.	ENSG00000181885	CLDN7	claudin 7	0.720218
104.	ENSG00000125656	CLPP	caseinolytic mitochondrial matrix peptidase proteolytic subunit	0.398841
105.	ENSG00000178531	CTXN1	cortexin 1	1.41001
106.	ENSG00000157456	CCNB2	cyclin B2	0.37596
107.	ENSG00000134480	CCNH	cyclin H	0.775269
108.	ENSG00000110104	CCDC86	coiled-coil domain containing 86	0.387673
109.	ENSG00000084072	PPIE	peptidylprolyl isomerase E	0.422785
110.	ENSG00000142871	CYR61	cysteine rich angiogenic inducer 61	0.4951
111.	ENSG00000184752	NDUFA12	NADH:ubiquinone oxidoreductase subunit A12	0.464626
112.	ENSG00000167969	ECI1	enoyl-CoA delta isomerase 1	0.525164

113.	ENSG00000137100	DCTN3	dynactin subunit 3	0.470114
114.	ENSG00000099977	DDT	D-dopachrome tautomerase	0.569309
115.	ENSG00000123136	DDX39A	DEAD-box helicase 39A	0.373273
116.	ENSG00000136271	DDX56	DEAD-box helicase 56	0.619725
117.	ENSG00000143753	DEGS1	"delta 4-desaturase, sphingolipid 1"	0.50347
118.	ENSG00000128185	DGCR6L	DiGeorge syndrome critical region gene 6-like	0.559326
119.	ENSG00000114956	DGUOK	deoxyguanosine kinase	0.398229
120.	ENSG00000104808	DHDH	dihydrodiol dehydrogenase	1.69723
121.	ENSG00000100867	DHRS2	dehydrogenase/reductase 2	0.555358
122.	ENSG00000175602	CCDC85B	coiled-coil domain containing 85B	0.992711
123.	ENSG00000203950	FAM127B	family with sequence similarity 127 member B	0.447223
124.	ENSG00000167130	DOLPP1	dolichyldiphosphatase 1	0.896244
125.	ENSG00000179085	DPM3	dolichyl-phosphate mannosyltransferase subunit 3	0.986599
126.	ENSG00000081721	DUSP12	dual specificity phosphatase 12	0.492875
127.	ENSG00000158716	DUSP23	dual specificity phosphatase 23	0.378719
128.	ENSG00000196205	EEF1A1P5	eukaryotic translation elongation factor 1 alpha 1 pseudogene 5	0.461171
129.	ENSG00000120533	ENY2	"ENY2, transcription and export complex 2 subunit"	0.63482
130.	ENSG00000224032	EPB41L4A-AS1	EPB41L4A antisense RNA 1	0.901618
131.	ENSG00000112029	FBXO5	F-box protein 5	0.374508
132.	ENSG00000144199	FAHD2B	fumarylacetoacetate hydrolase domain containing 2B	0.681793
133.	ENSG00000158234	FAIM	Fas apoptotic inhibitory molecule	0.410446
134.	ENSG00000225663	MCRIP1	MAPK regulated corepressor interacting protein 1	0.491688
135.	ENSG00000164451	FAM26D	family with sequence similarity 26 member D	1.88422
136.	ENSG00000154864	PIEZO2	piezo type mechanosensitive ion channel component 2	3.29995
137.	ENSG00000251669	FAM86EP	"family with sequence similarity 86 member E, pseudogene"	0.737164
138.	ENSG00000183161	FANCF	Fanconi anemia complementation group F	0.471178
139.	ENSG00000104870	FCGRT	Fc fragment of IgG receptor and transporter	0.776339
140.	ENSG00000156427	FGF18	fibroblast growth factor 18	0.719496
141.	ENSG00000115641	FHL2	four and a half LIM domains 2	0.445715
142.	ENSG00000160688	FLAD1	flavin adenine dinucleotide synthetase 1	0.556943
143.	ENSG00000155393	HEATR3	HEAT repeat containing 3	0.716759
144.	ENSG00000165792	METTL17	methyltransferase like 17	0.682262
145.	ENSG00000106125	FAM188B	family with sequence similarity 188 member B	0.602637
146.	ENSG00000223764	LOC100130417	uncharacterized LOC100130417	0.430213
147.	ENSG00000126391	FRMD8	FERM domain containing 8	0.585091
148.	ENSG00000186628	FSD2	fibronectin type III and SPRY domain containing 2	0.926203
149.	ENSG00000136371	MTHFS	"5,10-methenyltetrahydrofolate synthetase (5-formyltetrahydrofolate cyclo-ligase)"	0.618357
150.	ENSG00000068438	FTSJ1	FtsJ RNA methyltransferase homolog 1 (E. coli)	0.55001
151.	ENSG00000137726	FXYD6	FXYD domain containing ion transport regulator 6	0.580413
152.	ENSG00000180340	FZD2	frizzled class receptor 2	0.413671
153.	ENSG00000165060	FXN	frataxin	0.529412
154.	ENSG00000149100	EIF3M	eukaryotic translation initiation factor 3 subunit M	0.43418

155.	ENSG00000110328	GALNT18	polypeptide N-acetylgalactosaminyltransferase 18	0.394038
156.	ENSG00000174473	GALNTL6	polypeptide N-acetylgalactosaminyltransferase-like 6	0.763889
157.	ENSG00000197308	GATA3-AS1	GATA3 antisense RNA 1	0.671649
158.	ENSG00000034713	GABARAPL2	GABA type A receptor associated protein like 2	0.564498
159.	ENSG00000117228	GBP1	guanylate binding protein 1	0.784455
160.	ENSG00000120820	GLT8D2	glycosyltransferase 8 domain containing 2	1.69809
161.	ENSG00000197858	GPAA1	glycosylphosphatidylinositol anchor attachment 1	0.486139
162.	ENSG00000167468	GPX4	glutathione peroxidase 4	0.410368
163.	ENSG00000172432	GTPBP2	GTP binding protein 2	0.490921
164.	ENSG00000178605	GTPBP6	GTP binding protein 6 (putative)	0.489066
165.	ENSG00000069482	GAL	galanin and GMAP prepropeptide	0.695747
166.	ENSG00000112312	GMNN	"geminin, DNA replication inhibitor"	0.406285
167.	ENSG00000184897	H1FX	H1 histone family member X	0.388129
168.	ENSG00000213397	HAUS7	HAUS augmin like complex subunit 7	0.566935
169.	ENSG00000113070	HBEGF	heparin binding EGF like growth factor	0.895028
170.	ENSG00000164683	HEY1	hes related family bHLH transcription factor with YRPW motif 1	1.22995
171.	ENSG00000135547	HEY2	hes related family bHLH transcription factor with YRPW motif 2	0.424438
172.	ENSG00000168269	FOXI1	forkhead box I1	3.43907
173.	ENSG00000196787	HIST1H2AG	histone cluster 1 H2A family member g	0.390069
174.	ENSG00000196747	HIST1H2AI	histone cluster 1 H2A family member i	0.842689
175.	ENSG00000184260	HIST2H2AC	histone cluster 2 H2A family member c	0.939071
176.	ENSG00000181218	HIST3H2A	histone cluster 3 H2A	1.69035
177.	ENSG00000214821	HMGB1P4	high mobility group box 1 pseudogene 4	3.41823
178.	ENSG00000173917	HOXB2	homeobox B2	2.31017
179.	ENSG00000060688	SNRNP40	small nuclear ribonucleoprotein U5 subunit 40	0.487184
180.	ENSG00000133265	HSPBP1	HSPA (Hsp70) binding protein 1	0.56981
181.	ENSG00000048162	NOP16	NOP16 nucleolar protein	0.959119
182.	ENSG00000150316	CWC15	CWC15 spliceosome associated protein homolog	0.391368
183.	ENSG00000189060	H1F0	H1 histone family member 0	0.371105
184.	ENSG00000187837	HIST1H1C	histone cluster 1 H1 family member c	0.353865
185.	ENSG00000188486	H2AFX	H2A histone family member X	0.744619
186.	ENSG00000115457	IGFBP2	insulin like growth factor binding protein 2	0.597234
187.	ENSG00000125968	ID1	"inhibitor of DNA binding 1, HLH protein"	0.589337
188.	ENSG00000117318	ID3	"inhibitor of DNA binding 3, HLH protein"	0.718576
189.	ENSG00000165949	IFI27	interferon alpha inducible protein 27	1.18678
190.	ENSG00000185201	IFITM2	interferon induced transmembrane protein 2	0.714871
191.	ENSG00000142089	IFITM3	interferon induced transmembrane protein 3	0.597062
192.	ENSG00000171855	IFNB1	interferon beta 1	0.816242
193.	ENSG00000109083	IFT20	intraflagellar transport 20	1.00115

194.	ENSG00000189108	IL1RAPL2	interleukin 1 receptor accessory protein like 2	2.30333
195.	ENSG00000182393	IFNL1	interferon lambda 1	1.56572
196.	ENSG00000153391	INO80C	INO80 complex subunit C	0.530949
197.	ENSG00000169245	CXCL10	C-X-C motif chemokine ligand 10	2.51181
198.	ENSG00000160051	IQCC	IQ motif containing C	0.499756
199.	ENSG00000185507	IRF7	interferon regulatory factor 7	0.713054
200.	ENSG00000176842	IRX5	iroquois homeobox 5	0.563473
201.	ENSG00000187608	ISG15	ISG15 ubiquitin-like modifier	0.945481
202.	ENSG00000083457	ITGAE	integrin subunit alpha E	0.607598
203.	ENSG00000124249	KCNK15	potassium two pore domain channel subfamily K member 15	1.01492
204.	ENSG00000185760	KCNQ5	potassium voltage-gated channel subfamily Q member 5	1.45194
205.	ENSG00000080608	PUM3	pumilio RNA binding family member 3	0.629458
206.	ENSG00000167566	NCKAP5L	NCK associated protein 5 like	0.887664
207.	ENSG00000183655	KLHL25	kelch like family member 25	0.558841
208.	ENSG00000124743	KLHL31	kelch like family member 31	1.17539
209.	ENSG00000139187	KLRG1	killer cell lectin like receptor G1	2.02421
210.	ENSG00000157992	KRTCAP3	keratinocyte associated protein 3	0.574384
211.	ENSG00000167768	KRT1	keratin 1	1.27951
212.	ENSG00000186395	KRT10	keratin 10	0.51963
213.	ENSG00000132434	LANCL2	LanC like 2	0.746629
214.	ENSG00000106852	LHX6	LIM homeobox 6	0.774015
215.	ENSG00000224843	LINC00240	long intergenic non-protein coding RNA 240	1.60272
216.	ENSG00000203930	LINC00632	long intergenic non-protein coding RNA 632	1.50538
217.	ENSG00000175701	LINC00116	long intergenic non-protein coding RNA 116	0.452601
218.	ENSG00000221821	C6orf226	chromosome 6 open reading frame 226	0.630026
219.	ENSG00000262587	LOC554206	leucine carboxyl methyltransferase 1 pseudogene	1.35132
220.	ENSG00000223756	TSSC2	tumor suppressing subtransferable candidate 2 pseudogene	0.767636
221.	ENSG00000128011	LRFN1	leucine rich repeat and fibronectin type III domain containing 1	0.611146
222.	ENSG00000116212	LRRC42	leucine rich repeat containing 42	0.358644
223.	ENSG00000257103	LSM14A	"LSM14A, mRNA processing body assembly factor"	0.645035
224.	ENSG00000164167	LSM6	"LSM6 homolog, U6 small nuclear RNA and mRNA degradation associated"	0.417445
225.	ENSG00000073803	MAP3K13	mitogen-activated protein kinase kinase kinase 13	0.874193
226.	ENSG00000178982	EIF3K	eukaryotic translation initiation factor 3 subunit K	0.384723
227.	ENSG00000247626	MARS2	"methionyl-tRNA synthetase 2, mitochondrial"	0.42871
228.	ENSG00000168906	MAT2A	methionine adenosyltransferase 2A	0.483105
229.	ENSG00000071655	MBD3	methyl-CpG binding domain protein 3	0.865492
230.	ENSG00000106305	AIMP2	aminoacyl tRNA synthetase complex interacting multifunctional protein 2	1.51209
231.	ENSG00000076706	MCAM	melanoma cell adhesion molecule	1.18409
232.	ENSG00000037897	METTL1	methyltransferase like 1	0.906915
233.	ENSG00000087995	METTL2A	methyltransferase like 2A	0.395521

234.	ENSG00000167700	MFSD3	major facilitator superfamily domain containing 3	0.585684
235.	ENSG00000133808	MICALCL	MICAL C-terminal like	1.32669
236.	ENSG00000240972	MIF	macrophage migration inhibitory factor (glycosylation-inhibiting factor)	0.833516
237.	ENSG00000230937	MIR205HG	MIR205 host gene	1.11661
238.	ENSG00000227195	MIR663AHG	MIR663A host gene	1.20766
239.	ENSG00000108961	RANGRF	RAN guanine nucleotide release factor	0.5459
240.	ENSG00000137547	MRPL15	mitochondrial ribosomal protein L15	0.474103
241.	ENSG00000242485	MRPL20	mitochondrial ribosomal protein L20	0.531287
242.	ENSG00000106591	MRPL32	mitochondrial ribosomal protein L32	0.858848
243.	ENSG00000130312	MRPL34	mitochondrial ribosomal protein L34	0.870551
244.	ENSG00000105364	MRPL4	mitochondrial ribosomal protein L4	0.409564
245.	ENSG00000055950	MRPL43	mitochondrial ribosomal protein L43	0.599647
246.	ENSG00000259494	MRPL46	mitochondrial ribosomal protein L46	0.762839
247.	ENSG00000183617	MRPL54	mitochondrial ribosomal protein L54	0.556519
248.	ENSG00000128626	MRPS12	mitochondrial ribosomal protein S12	0.583302
249.	ENSG00000239789	MRPS17	mitochondrial ribosomal protein S17	0.736926
250.	ENSG00000096080	MRPS18A	mitochondrial ribosomal protein S18A	0.393735
251.	ENSG00000074071	MRPS34	mitochondrial ribosomal protein S34	0.627567
252.	ENSG00000243927	MRPS6	mitochondrial ribosomal protein S6	0.739482
253.	ENSG00000183155	RABIF	RAB interacting factor	0.357549
254.	ENSG00000182551	ADI1	acireductone dioxygenase 1	0.481005
255.	ENSG00000242114	MTFP1	mitochondrial fission process 1	1.47424
256.	ENSG00000162385	MAGOH	"mago homolog, exon junction complex core component"	0.457134
257.	ENSG00000173171	MTX1	metaxin 1	0.663453
258.	ENSG00000182208	MOB2	MOB kinase activator 2	0.524078
259.	ENSG00000176101	SSNA1	SS nuclear autoantigen 1	0.430655
260.	ENSG00000166681	BEX3	brain expressed X-linked 3	0.43456
261.	ENSG00000147813	NAPRT	nicotinate phosphoribosyltransferase	0.653233
262.	ENSG00000174886	NDUFA11	NADH:ubiquinone oxidoreductase subunit A11	0.599199
263.	ENSG00000131495	NDUFA2	NADH:ubiquinone oxidoreductase subunit A2	0.823501
264.	ENSG00000128609	NDUFA5	NADH:ubiquinone oxidoreductase subunit A5	0.41167
265.	ENSG00000119421	NDUFA8	NADH:ubiquinone oxidoreductase subunit A8	0.45136
266.	ENSG00000147123	NDUFB11	NADH:ubiquinone oxidoreductase subunit B11	0.603894
267.	ENSG00000090266	NDUFB2	NADH:ubiquinone oxidoreductase subunit B2	0.578461
268.	ENSG00000099795	NDUFB7	NADH:ubiquinone oxidoreductase subunit B7	0.592951
269.	ENSG00000151366	NDUFC2	NADH:ubiquinone oxidoreductase subunit C2	0.539958
270.	ENSG00000145494	NDUFS6	NADH:ubiquinone oxidoreductase subunit S6	0.406572
271.	ENSG00000167792	NDUFV1	NADH:ubiquinone oxidoreductase core subunit V1	0.384126

272.	ENSG00000160949	TONSL	"tonsoku-like, DNA repair protein"	0.477561
273.	ENSG00000100138	SNU13	"SNU13 homolog, small nuclear ribonucleoprotein (U4/U6.U5)"	0.587735
274.	ENSG00000123609	NMI	N-myc and STAT interactor	0.517159
275.	ENSG00000110107	PRPF19	pre-mRNA processing factor 19	0.526223
276.	ENSG00000107672	NSMCE4A	"NSE4 homolog A, SMC5-SMC6 complex component"	0.423395
277.	ENSG00000205309	NT5M	"5',3'-nucleotidase, mitochondrial"	0.501565
278.	ENSG00000151413	NUBPL	nucleotide binding protein like	0.558354
279.	ENSG00000083635	NUFIP1	"NUFIP1, FMR1 interacting protein 1"	0.513718
280.	ENSG00000185818	NAT8L	N-acetyltransferase 8 like	0.361264
281.	ENSG00000163938	GNL3	G protein nucleolar 3	0.741709
282.	ENSG00000154814	OXNAD1	oxidoreductase NAD binding domain containing 1	0.488446
283.	ENSG00000092010	PSME1	proteasome activator subunit 1	0.532391
284.	ENSG00000100911	PSME2	proteasome activator subunit 2	0.608229
285.	ENSG00000107951	MTPAP	mitochondrial poly(A) polymerase	0.434545
286.	ENSG00000100300	TSPO	translocator protein	0.522258
287.	ENSG00000125450	NUP85	nucleoporin 85	0.687256
288.	ENSG00000197461	PDGFA	platelet derived growth factor subunit A	0.413027
289.	ENSG00000255185	PDXDC2P	"pyridoxal dependent decarboxylase domain containing 2, pseudogene"	1.52514
290.	ENSG00000143256	PFDN2	prefoldin subunit 2	0.726832
291.	ENSG00000184207	PGP	phosphoglycolate phosphatase	0.457967
292.	ENSG00000173457	PPP1R14B	protein phosphatase 1 regulatory inhibitor subunit 14B	0.378728
293.	ENSG00000054148	PHPT1	phosphohistidine phosphatase 1	0.418984
294.	ENSG00000105559	PLEKHA4	pleckstrin homology domain containing A4	0.787814
295.	ENSG00000102934	PLLP	plasmolipin	0.437388
296.	ENSG00000114631	PODXL2	podocalyxin like 2	0.404692
297.	ENSG00000062822	POLD1	"DNA polymerase delta 1, catalytic subunit"	0.75975
298.	ENSG00000100479	POLE2	"DNA polymerase epsilon 2, accessory subunit"	0.603001
299.	ENSG00000205808	PLPP6	phospholipid phosphatase 6	0.490681
300.	ENSG00000219797	PPIAP9	peptidylprolyl isomerase A pseudogene 9	0.404168
301.	ENSG00000049769	PPP1R3F	protein phosphatase 1 regulatory subunit 3F	0.839678
302.	ENSG00000122490	PQLC1	PQ loop repeat containing 1	0.720016
303.	ENSG00000204673	AKT1S1	AKT1 substrate 1	0.445086
304.	ENSG00000123143	PKN1	protein kinase N1	0.51293
305.	ENSG00000126457	PRMT1	protein arginine methyltransferase 1	0.414225
306.	ENSG00000224940	PRRT4	proline rich transmembrane protein 4	0.60223
307.	ENSG00000205220	PSMB10	proteasome subunit beta 10	0.904058
308.	ENSG00000142507	PSMB6	proteasome subunit beta 6	0.354566
309.	ENSG00000163636	PSMD6	"proteasome 26S subunit, non-ATPase 6"	0.389134
310.	ENSG00000110801	PSMD9	"proteasome 26S subunit, non-ATPase 9"	0.380317
311.	ENSG00000157778	PSMG3	proteasome assembly chaperone 3	0.788305
312.	ENSG00000176894	PXMP2	peroxisomal membrane protein 2	1.22584
313.	ENSG00000101417	PXMP4	peroxisomal membrane protein 4	0.512692
314.	ENSG00000173599	PC	pyruvate carboxylase	1.34458

315.	ENSG00000104524	PYCRL	pyrroline-5-carboxylate reductase-like	0.68122
316.	ENSG00000099864	PALM	paralemmin	0.534304
317.	ENSG00000127445	PIN1	"peptidylprolyl cis/trans isomerase, NIMA-interacting 1"	0.517536
318.	ENSG00000102312	PORCN	porcupine homolog (Drosophila)	0.7285
319.	ENSG00000102109	PCSK1N	proprotein convertase subtilisin/kexin type 1 inhibitor	0.468133
320.	ENSG00000167964	RAB26	"RAB26, member RAS oncogene family"	0.801729
321.	ENSG00000141542	RAB40B	"RAB40B, member RAS oncogene family"	0.766176
322.	ENSG00000128045	RASL11B	RAS like family 11 member B	0.63957
323.	ENSG00000102317	RBM3	"RNA binding motif (RNP1, RRM) protein 3"	0.410045
324.	ENSG00000204977	TRIM13	tripartite motif containing 13	0.462184
325.	ENSG00000116741	RGS2	regulator of G-protein signaling 2	0.603821
326.	ENSG00000107201	DDX58	DEXD/H-box helicase 58	0.530166
327.	ENSG00000183421	RIPK4	receptor interacting serine/threonine kinase 4	0.396886
328.	ENSG00000269900	RMRP	RNA component of mitochondrial RNA processing endoribonuclease	1.02715
329.	ENSG00000202198	RN7SK	"RNA, 7SK small nuclear"	0.491338
330.	ENSG00000158717	RNF166	ring finger protein 166	0.601905
331.	ENSG00000117748	RPA2	replication protein A2	0.446348
332.	ENSG00000100142	POLR2F	RNA polymerase II subunit F	0.468156
333.	ENSG00000063177	RPL18	ribosomal protein L18	0.44233
334.	ENSG00000105640	RPL18A	ribosomal protein L18a	0.691689
335.	ENSG00000130255	RPL36	ribosomal protein L36	0.724443
336.	ENSG00000213553	RPLP0P6	ribosomal protein lateral stalk subunit P0 pseudogene 6	0.619279
337.	ENSG00000137818	RPLP1	ribosomal protein lateral stalk subunit P1	0.500618
338.	ENSG00000177600	RPLP2	ribosomal protein lateral stalk subunit P2	0.440122
339.	ENSG00000172336	POP7	"POP7 homolog, ribonuclease P/MRP subunit"	0.719657
340.	ENSG00000115268	RPS15	ribosomal protein S15	0.440372
341.	ENSG00000105372	RPS19	ribosomal protein S19	0.404088
342.	ENSG00000140988	RPS2	ribosomal protein S2	0.443262
343.	ENSG00000179041	RRS1	ribosome biogenesis regulator homolog	0.481912
344.	ENSG00000136444	RSAD1	radical S-adenosyl methionine domain containing 1	0.475825
345.	ENSG00000222014	RAB6C	"RAB6C, member RAS oncogene family"	0.768564
346.	ENSG00000123595	RAB9A	"RAB9A, member RAS oncogene family"	0.435617
347.	ENSG00000169750	RAC3	"ras-related C3 botulinum toxin substrate 3 (rho family, small GTP binding protein Rac3)"	0.460522
348.	ENSG00000099901	RANBP1	RAN binding protein 1	0.631193
349.	ENSG00000142552	RCN3	reticulocalbin 3	1.34694
350.	ENSG00000173156	RHOD	ras homolog family member D	0.615585
351.	ENSG00000166788	SAAL1	serum amyloid A like 1	0.448924
352.	ENSG00000168061	SAC3D1	SAC3 domain containing 1	0.824352
353.	ENSG00000205413	SAMD9	sterile alpha motif domain containing 9	0.587933
354.	ENSG00000150459	SAP18	Sin3A associated protein 18	0.407532
355.	ENSG00000105472	CLEC11A	C-type lectin domain family 11 member A	1.68108
356.	ENSG00000133028	SCO1	"SCO1, cytochrome c oxidase assembly protein"	0.387032
357.	ENSG00000121064	SCPEP1	serine carboxypeptidase 1	0.44248

358.	ENSG00000205138	SDHAF1	succinate dehydrogenase complex assembly factor 1	0.578079
359.	ENSG00000171222	SCAND1	SCAN domain containing 1	0.981319
360.	ENSG00000085415	SEH1L	SEH1 like nucleoporin	0.48006
361.	ENSG00000141295	SCRN2	secernin 2	0.474364
362.	ENSG00000161547	SRSF2	serine and arginine rich splicing factor 2	0.540135
363.	ENSG00000129946	SHC2	SHC adaptor protein 2	0.701544
364.	ENSG00000108528	SLC25A11	solute carrier family 25 member 11	0.529239
365.	ENSG00000125454	SLC25A19	solute carrier family 25 member 19	0.903744
366.	ENSG00000177542	SLC25A22	solute carrier family 25 member 22	0.81186
367.	ENSG00000155287	SLC25A28	solute carrier family 25 member 28	0.469913
368.	ENSG00000075415	SLC25A3	solute carrier family 25 member 3	0.430978
369.	ENSG00000122692	SMU1	DNA replication regulator and spliceosomal factor	0.391938
370.	ENSG00000188338	SLC38A3	solute carrier family 38 member 3	1.45116
371.	ENSG00000174365	SNHG11	small nucleolar RNA host gene 11	0.508052
372.	ENSG00000269893	SNHG8	small nucleolar RNA host gene 8	0.748491
373.	ENSG00000255198	SNHG9	small nucleolar RNA host gene 9	0.799658
374.	ENSG00000263934	SNORD3A	"small nucleolar RNA, C/D box 3A"	1.36868
375.	ENSG00000125835	SNRPB	small nuclear ribonucleoprotein polypeptides B and B1	0.614339
376.	ENSG00000028528	SNX1	sorting nexin 1	0.726085
377.	ENSG00000158792	SPATA2L	spermatogenesis associated 2 like	1.18964
378.	ENSG00000124193	SRSF6	serine and arginine rich splicing factor 6	0.382337
379.	ENSG00000106028	SSBP1	single stranded DNA binding protein 1	0.443889
380.	ENSG00000180879	SSR4	signal sequence receptor subunit 4	0.559991
381.	ENSG00000180953	ST20	suppressor of tumorigenicity 20	0.847186
382.	ENSG00000169689	STRA13	stimulated by retinoic acid 13	0.585916
383.	ENSG00000148290	SURF1	"SURF1, cytochrome c oxidase assembly factor"	0.530676
384.	ENSG00000148296	SURF6	surfeit 6	0.394365
385.	ENSG00000157703	SVOPL	SVOP like	1.37631
386.	ENSG00000100321	SYNGR1	synaptogyrin 1	0.498366
387.	ENSG00000180104	EXOC3	exocyst complex component 3	0.550317
388.	ENSG00000073169	SELO	selenoprotein O	0.435145
389.	ENSG00000170892	TSEN34	tRNA splicing endonuclease subunit 34	0.365
390.	ENSG00000162878	PKDCC	"protein kinase domain containing, cytoplasmic"	0.511279
391.	ENSG00000144040	SFXN5	sideroflexin 5	1.0396
392.	ENSG00000089163	SIRT4	sirtuin 4	1.43437
393.	ENSG00000184047	DIABLO	diablo IAP-binding mitochondrial protein	0.674556
394.	ENSG00000197457	STMN3	stathmin 3	0.666294
395.	ENSG00000135111	TBX3	T-box 3	0.958524
396.	ENSG00000261787	TCF24	transcription factor 24	0.967519
397.	ENSG00000148308	GTF3C5	general transcription factor IIIC subunit 5	0.479835
398.	ENSG00000168286	THAP11	THAP domain containing 11	0.720457
399.	ENSG00000184436	THAP7	THAP domain containing 7	0.749494
400.	ENSG00000161277	THAP8	THAP domain containing 8	0.669541

401.	ENSG00000133321	RARRES3	retinoic acid receptor responder 3	0.850546
402.	ENSG00000180346	TIGD2	tigger transposable element derived 2	0.38924
403.	ENSG00000105197	TIMM50	translocase of inner mitochondrial membrane 50	0.566558
404.	ENSG00000075131	TIPIN	TIMELESS interacting protein	0.433057
405.	ENSG00000134851	TMEM165	transmembrane protein 165	0.539908
406.	ENSG00000226287	TMEM191A	transmembrane protein 191A (pseudogene)	1.01268
407.	ENSG00000188807	TMEM201	transmembrane protein 201	0.624589
408.	ENSG00000180730	SHISA2	shisa family member 2	0.457364
409.	ENSG00000178821	TMEM52	transmembrane protein 52	0.772302
410.	ENSG00000135211	TMEM60	transmembrane protein 60	0.510866
411.	ENSG00000175606	TMEM70	transmembrane protein 70	0.484836
412.	ENSG00000127774	EMC6	ER membrane protein complex subunit 6	0.852474
413.	ENSG00000187653	TMSB4XP8	"thymosin beta 4, X-linked pseudogene 8"	1.1459
414.	ENSG00000164758	MED30	mediator complex subunit 30	0.408796
415.	ENSG00000181029	TRAPPC5	trafficking protein particle complex 5	0.784778
416.	ENSG00000123297	TSFM	"Ts translation elongation factor, mitochondrial"	0.512266
417.	ENSG00000180543	TSPYL5	TSPY like 5	0.435672
418.	ENSG00000128311	TST	thiosulfate sulfurtransferase	0.563092
419.	ENSG00000104522	TSTA3	tissue specific transplantation antigen P35B	0.469903
420.	ENSG00000011295	TTC19	tetratricopeptide repeat domain 19	0.883377
421.	ENSG00000183891	TTC32	tetratricopeptide repeat domain 32	0.483994
422.	ENSG00000129235	TXNDC17	thioredoxin domain containing 17	0.462714
423.	ENSG00000156521	TYSND1	trypsin domain containing 1	0.487563
424.	ENSG00000177370	TIMM22	translocase of inner mitochondrial membrane 22 homolog	0.343774
425.	ENSG00000170315	UBB	ubiquitin B	0.431675
426.	ENSG00000160087	UBE2J2	ubiquitin conjugating enzyme E2 J2	0.371906
427.	ENSG00000156587	UBE2L6	ubiquitin conjugating enzyme E2 L6	0.405366
428.	ENSG00000115446	UNC50	unc-50 inner nuclear membrane RNA binding protein	0.381783
429.	ENSG00000127540	UQCR11	"ubiquinol-cytochrome c reductase, complex III subunit XI"	0.70226
430.	ENSG00000184979	USP18	ubiquitin specific peptidase 18	0.673708
431.	ENSG00000176125	UFSP1	UFM1 specific peptidase 1 (inactive)	0.82111
432.	ENSG00000136146	MED4	mediator complex subunit 4	0.486201
433.	ENSG00000167397	VKORC1	vitamin K epoxide reductase complex subunit 1	0.60749
434.	ENSG00000100749	VRK1	vaccinia related kinase 1	0.561483
435.	ENSG00000085449	WDFY1	WD repeat and FYVE domain containing 1	0.477846
436.	ENSG00000119333	WDR34	WD repeat domain 34	0.967734
437.	ENSG00000133316	WDR74	WD repeat domain 74	0.430277
438.	ENSG00000152763	WDR78	WD repeat domain 78	1.73907
439.	ENSG00000136936	XPA	"XPA, DNA damage recognition and repair factor"	0.635993
440.	ENSG00000103489	XYLT1	xylosyltransferase 1	0.468212
441.	ENSG00000247315	ZCCHC3	zinc finger CCHC-type containing 3	0.530817
442.	ENSG00000112473	SLC39A7	solute carrier family 39 member 7	0.427647
443.	ENSG00000197961	ZNF121	zinc finger protein 121	0.478525

444.	ENSG00000249471	ZNF324B	zinc finger protein 324B	0.824604
445.	ENSG00000131116	ZNF428	zinc finger protein 428	0.664193
446.	ENSG00000198298	ZNF485	zinc finger protein 485	0.48972
447.	ENSG00000204946	ZNF783	zinc finger family member 783	0.774064
448.	ENSG00000152475	ZNF837	zinc finger protein 837	0.399333
449.	ENSG00000166707	ZCCHC18	zinc finger CCHC-type containing 18	1.40314
450.	ENSG00000115194	SLC30A3	solute carrier family 30 member 3	1.0649
451.	ENSG00000139405	RITA1	RBPJ interacting and tubulin associated 1	0.39254

Supplementary Table 3: miR-29 targets common in MCF-7 and LCC9s

Supplementary Table 3A: Common miR-29b-1 and miR-29a targets unique MCF-7 cells

			MCF-7_Pre-miR-29b1 vs Anti-miR-29a	MCF-7_Pre-miR-29a vs Anti-miR-29a
	Gene Symbol	Gene Name	log2(fold_change)	log2(fold_change)
1.	ACO1	aconitase 1	0.950629	0.626089
2.	AIM1	absent in melanoma 1	0.778095	0.658569
3.	AKR1C3	aldo-keto reductase family 1 member C3	0.885048	0.645128
4.	ANKRD50	ankyrin repeat domain 50	0.73071	0.554446
5.	ATP1A1	ATPase Na+/K+ transporting subunit alpha 1	0.64489	0.431066
6.	ATP7A	ATPase copper transporting alpha	0.798753	0.616152
7.	ATRN	attractin	0.717643	0.492748
8.	BMP5	bone morphogenetic protein 5	0.836287	0.658327
9.	CAV1	caveolin 1	1.16046	1.01584
10.	CMBL	carboxymethylenebutenolidase homolog	0.765169	0.549379
11.	CRISP3	cysteine rich secretory protein 3	1.1613	0.739149
12.	CSGALNACT1	chondroitin sulfate N-acetylgalactosaminyltransferase 1	1.41892	1.27735
13.	CYP19A1	cytochrome P450 family 19 subfamily A member 1	2.32583	2.35814
14.	DNAJC28	DnaJ heat shock protein family (Hsp40) member C28	1.06775	0.92687
15.	DSC2	desmocollin 2	1.29529	1.10679
16.	DSG2	desmoglein 2	0.743464	0.481257
17.	EPHA6	EPH receptor A6	1.03363	0.877418
18.	EPHA7	EPH receptor A7	0.879505	0.701687
19.	ERMP1	endoplasmic reticulum metallopeptidase 1	0.939574	0.679547
20.	FBP1	fructose-bisphosphatase 1	0.45042	0.490642
21.	FILIP1L	filamin A interacting protein 1 like	0.667145	0.583131
22.	HEPHL1	hephaestin like 1	1.38329	1.14249
23.	HMGCLL1	3-hydroxymethyl-3-methylglutaryl-CoA lyase like 1	1.15732	1.03274
24.	IGFBP5	insulin like growth factor binding protein 5	0.52921	0.390971
25.	KCNJ8	potassium voltage-gated channel subfamily J member 8	0.940332	0.848572
26.	KIAA1549	KIAA1549	0.770061	0.663556
27.	KLHL4	kelch like family member 4	1.07248	0.81465
28.	KLHL5	kelch like family member 5	0.756199	0.602252
29.	KRT23	keratin 23	0.859914	0.780114
30.	LTBP1	latent transforming growth factor beta binding protein 1	1.32021	0.921458
31.	MALL	"mal, T-cell differentiation protein like"	1.60014	1.58334
32.	MIB1	mindbomb E3 ubiquitin protein ligase 1	0.73351	0.521844
33.	MIR17HG	miR-17-92a-1 cluster host gene	2.37174	2.2729
34.	NAALADL2	N-acetylated alpha-linked acidic dipeptidase like 2	0.69365	0.67568
35.	NPNT	nephronectin	0.774074	0.579707
36.	PANX1	pannexin 1	0.666987	0.487432
37.	PDK3	pyruvate dehydrogenase kinase 3	0.771457	0.564068

38.	PERP	"PERP, TP53 apoptosis effector"	0.587256	0.421452
39.	PGM5	phosphoglucomutase 5	2.04583	1.71094
40.	PKP1	plakophilin 1	1.25349	1.28295
41.	PSG9	pregnancy specific beta-1-glycoprotein 9	0.84128	0.757355
42.	RTN3	reticulon 3	0.612048	0.503042
43.	S100A10	S100 calcium binding protein A10	0.574568	0.414773
44.	SCIN	scinderin	1.3387	1.19559
45.	SCUBE2	"signal peptide, CUB domain and EGF like domain containing 2"	0.935904	0.815793
46.	SEMA3D	semaphorin 3D	1.48777	1.16477
47.	SERINC5	serine incorporator 5	0.805837	0.548127
48.	SLC16A2	solute carrier family 16 member 2	1.25596	0.854277
49.	SLC24A3	solute carrier family 24 member 3	0.589257	0.4987
50.	SLITRK6	SLIT and NTRK like family member 6	0.786628	0.553729
51.	SORT1	sortilin 1	0.749978	0.522416
52.	STK38	serine/threonine kinase 38	0.455075	0.426797
53.	STON1	stonin 1	1.5401	1.4028
54.	TMEM45B	transmembrane protein 45B	0.797278	0.752252
55.	TMTC3	transmembrane and tetratricopeptide repeat containing 3	0.828496	0.643048
56.	TOP2B	topoisomerase (DNA) II beta	0.707055	0.519007
57.	TRAK2	trafficking kinesin protein 2	0.706495	0.568506
58.	UNC5C	unc-5 netrin receptor C	1.2494	1.47876
59.	VAT1L	vesicle amine transport 1 like	1.16268	1.0319
60.	WNT2B	Wnt family member 2B	1.1319	1.0473

Supplementary Table 3B: Common miR-29b-1 and miR-29a targets common to both MCF-7 and LCC9 cells

			MCF-7_Pre-miR-29b1 vs Anti-miR-29a	MCF-7_Pre-miR-29a vs Anti-miR-29a	LCC9_Pre-miR-29b1 vs Anti-miR-29a	LCC9_Pre-miR-29a vs Anti-miR-29a
	Gene Symbol	Gene Name	log2(fold_change)	log2(fold_change)	log2(fold_change)	log2(fold_change)
	ANXA1	annexin A1	0.86142	0.786981	2.13207	2.17304
	ANXA5	annexin A5	0.553169	0.52333	1.17391	1.22543
	ARL4C	ADP ribosylation factor like GTPase 4C	0.554121	0.695993	1.11503	1.06863
	ARPC5	actin related protein 2/3 complex subunit 5	0.586134	0.607786	1.505	1.64043
	ATP5C1	"ATP synthase, H+ transporting, mitochondrial F1 complex, gamma polypeptide 1"	0.6197	0.540511	1.43753	1.34628
	BAK1P1	BCL2 antagonist/killer 1 pseudogene 1	0.72886	0.853615	1.33959	1.35957
	BPGM	bisphosphoglycerate mutase	0.744332	0.734537	1.74992	1.71232
	C4orf32	chromosome 4 open reading frame 32	0.716821	0.62176	0.895463	0.976444
	C7orf73	chromosome 7 open reading frame 73	0.803039	0.703753	1.89354	1.90098
	CCDC167	coiled-coil domain containing 167	0.573969	0.620072	3.39142	3.38646
	CD276	CD276 molecule	0.6514	0.687137	1.24127	1.1014
	CD36	CD36 molecule	0.991681	1.19904	1.5895	1.44303
	CLEC7A	C-type lectin domain family 7 member A	1.28516	1.75287	2.31239	4.17419
	DYNLT1	dynein light chain Tctex-type 1	0.498623	0.60201	1.94914	2.14702
	EFEMP1	EGF containing fibulin like extracellular matrix protein 1	0.943595	0.981479	1.53166	1.67697
	ELF5	E74 like ETS transcription factor 5	1.20403	1.00089	1.54622	1.41993
	ENO1	enolase 1	0.499112	0.49841	0.546022	0.474453
	EPB41L4B	erythrocyte membrane protein band 4.1 like 4B	0.59199	0.530691	0.73561	0.664133
	F11R	F11 receptor	0.829893	0.805344	1.09778	1.04492
	FAM102B	family with sequence similarity 102 member B	0.61018	0.463769	1.13096	0.902373
	FAM127C	family with sequence similarity 127 member C	0.511481	0.559729	1.25769	1.39421
	FECH	ferrochelatase	1.03486	0.802485	1.68044	1.65016
	FKBP1A	FK506 binding protein 1A	0.72236	0.541505	0.791857	0.845083
	FREM2	FRAS1 related extracellular matrix protein 2	1.2842	1.13966	1.6484	1.22025
	GALNT1	polypeptide N-acetylgalactosaminyltransferase 1	0.95207	0.767107	0.673639	0.543714
	GCSH	glycine cleavage system protein H	0.812153	0.678064	2.03522	1.98461
	GINM1	glycoprotein integral membrane 1	0.688129	0.599535	0.775513	0.697549
	GSTM3	glutathione S-transferase mu 3	0.603347	0.474469	0.95424	0.886405
	HOXC13	homeobox C13	0.546096	0.67975	2.13121	2.22351
	IGSF3	immunoglobulin superfamily member 3	0.98228	1.12764	1.06424	0.986889
	ISOC1	isochorismatase domain containing 1	0.720805	0.671419	1.88871	1.87293
	ITGB1	integrin subunit beta 1	0.816957	0.659117	0.987881	0.80671
	JARID2	jumonji and AT-rich interaction domain containing 2	0.474666	0.444702	1.09834	0.998283
	KDELC1	KDEL motif containing 1	1.0655	1.27176	3.01117	2.79997
	KDM5B	lysine demethylase 5B	0.98413	0.779044	0.845657	0.65676
	KIAA1161	KIAA1161	0.708471	0.59354	0.655048	0.688324

	KIT	KIT proto-oncogene receptor tyrosine kinase	1.99239	1.71144	2.10612	1.97156
	KLHDC3	kelch domain containing 3	0.612973	0.698181	1.94245	2.07298
	KRT80	keratin 80	0.387774	0.516173	1.24017	1.19701
	MAP2K6	mitogen-activated protein kinase kinase 6	1.35071	1.27931	2.61089	2.15353
	MAP4K4	mitogen-activated protein kinase kinase kinase kinase 4	0.6531	0.563603	1.24666	0.919106
	MBTD1	mbt domain containing 1	0.616126	0.501719	0.65506	0.544341
	METTL7A	methyltransferase like 7A	0.81592	0.96939	0.867371	0.772139
	MORF4L1	mortality factor 4 like 1	0.614757	0.563526	1.23656	1.21828
	MPZL3	myelin protein zero like 3	0.796882	0.842667	1.30983	1.22454
	MRFAP1	Morf4 family associated protein 1	0.465383	0.536648	2.03319	2.14254
	NANOS1	nanos C2HC-type zinc finger 1	0.858277	0.9082	1.56703	1.64991
	NRAS	neuroblastoma RAS viral oncogene homolog	0.552152	0.43652	0.650742	0.632976
	NTRK2	neurotrophic receptor tyrosine kinase 2	0.987398	1.21638	2.31639	1.68192
	PCP4	Purkinje cell protein 4	0.864802	0.712848	2.79053	2.29124
	PIK3R3	phosphoinositide-3-kinase regulatory subunit 3	0.801967	0.829185	1.14694	1.04752
	PPIC	peptidylprolyl isomerase C	1.21937	1.17803	2.04242	2.15937
	PPT1	palmitoyl-protein thioesterase 1	0.742549	0.688119	1.33288	1.28796
	PRMT6	protein arginine methyltransferase 6	0.576114	0.729643	2.51833	2.72602
	RCC2	regulator of chromosome condensation 2	0.401982	0.591432	2.40005	2.4671
	RCN2	reticulocalbin 2	0.531026	0.452163	0.698454	0.588073
	RPS24	ribosomal protein S24	0.555705	0.488549	2.25259	2.36119
	RPS3A	ribosomal protein S3A	0.687786	0.491714	1.35179	1.34338
	S100A16	S100 calcium binding protein A16	0.669323	0.682906	1.68258	1.66285
	SEMA3C	semaphorin 3C	0.865222	0.66281	0.907207	0.669273
	SESTD1	SEC14 and spectrin domain containing 1	1.10416	1.06537	1.24413	1.03235
	TBC1D7	TBC1 domain family member 7	0.921924	0.83745	2.64112	2.93259
	TDG	thymine DNA glycosylase	0.815785	0.81763	2.25669	2.35879
	TET1	tet methylcytosine dioxygenase 1	1.14532	1.06875	1.61365	1.33383
	TET3	tet methylcytosine dioxygenase 3	0.923868	1.01061	0.964832	0.931351
	TMEM117	transmembrane protein 117	1.76039	1.52825	1.22016	1.36977
	TMEM164	transmembrane protein 164	0.8879	0.803582	1.45174	1.44885
	TMSB4X	"thymosin beta 4, X-linked"	0.559619	0.483911	0.828414	0.954921
	UBTD2	ubiquitin domain containing 2	0.753085	0.627815	1.45232	1.38079
	VAMP7	vesicle associated membrane protein 7	0.950366	0.81555	1.74104	1.60978
	VOPP1	"vesicular, overexpressed in cancer, prosurvival protein 1"	0.476688	0.451507	0.46844	0.41793
	XXYLT1	xyloside xylosyltransferase 1	0.716947	0.799999	2.16137	2.27884

Supplementary Table 3C: **Common miR-29b-1 and miR-29a targets unique LCC9 cells**

			**LCC9_Pre-miR-29b1 vs Anti-miR-29a**	**LCC9_Pre-miR-29a vs Anti-miR-29a**
	**Gene Symbol**	**Gene Name**	**log2(fold_change)**	**log2(fold_change)**
	AAR2	AAR2 splicing factor homolog	0.927601	1.03828
	AARSD1	alanyl-tRNA synthetase domain containing 1	0.659615	0.639813
	ABCB6	ATP binding cassette subfamily B member 6 (Langereis blood group)	1.20686	1.2379
	ABCB7	ATP binding cassette subfamily B member 7	0.688644	0.580453
	ABCE1	ATP binding cassette subfamily E member 1	0.876384	0.815314
	ABHD13	abhydrolase domain containing 13	0.488825	0.459061
	ACBD7	acyl-CoA binding domain containing 7	0.700393	0.496153
	ACE	angiotensin I converting enzyme	1.21031	1.00259
	ACOX2	acyl-CoA oxidase 2	4.65734	3.66441
	ACSS1	acyl-CoA synthetase short-chain family member 1	0.732909	0.762267
	ACTG2	"actin, gamma 2, smooth muscle, enteric"	0.411173	0.462855
	ACTL8	actin like 8	1.37881	1.60098
	ACTR3B	ARP3 actin related protein 3 homolog B	1.03853	1.08886
	ADA	adenosine deaminase	1.37348	1.47158
	ADAMTS15	ADAM metallopeptidase with thrombospondin type 1 motif 15	2.63156	2.34575
	ADAMTS19	ADAM metallopeptidase with thrombospondin type 1 motif 19	1.47001	1.29029
	ADAT2	"adenosine deaminase, tRNA specific 2"	0.875681	0.969717
	ADGRB2	adhesion G protein-coupled receptor B2	0.874388	1.11731
	ADH5	"alcohol dehydrogenase 5 (class III), chi polypeptide"	1.00464	1.03491
	ADIPOR2	adiponectin receptor 2	0.690701	0.574571
	ADK	adenosine kinase	0.60946	0.586069
	ADNP2	ADNP homeobox 2	0.389951	0.375037
	ADORA1	adenosine A1 receptor	2.51826	2.81189
	ADORA2A	adenosine A2a receptor	2.30518	2.9572
	ADORA2B	adenosine A2b receptor	0.715716	0.757347
	ADSL	adenylosuccinate lyase	1.28924	1.31677
	ADSS	adenylosuccinate synthase	0.989145	1.09993
	AFMID	arylformamidase	0.518471	0.514579
	AGR3	"anterior gradient 3, protein disulphide isomerase family member"	0.59845	0.815454
	AGTR1	angiotensin II receptor type 1	2.79834	2.8424
	AHCY	adenosylhomocysteinase	0.494678	0.563421
	AHCYL1	adenosylhomocysteinase like 1	0.599927	0.449753
	AIFM1	"apoptosis inducing factor, mitochondria associated 1"	0.517603	0.400808
	AK2	adenylate kinase 2	0.58514	0.599814
	AK4	adenylate kinase 4	0.59315	0.563226
	AKR1A1	aldo-keto reductase family 1 member A1	0.690468	0.593904
	AKR7A2	aldo-keto reductase family 7 member A2	0.590043	0.622283
	ALDH7A1	aldehyde dehydrogenase 7 family member A1	0.711066	0.563229
	ALG5	"ALG5, dolichyl-phosphate beta-glucosyltransferase"	0.974665	1.17464
	ALG6	"ALG6, alpha-1,3-glucosyltransferase"	0.725901	0.842662
	ALKBH2	"alkB homolog 2, alpha-ketoglutarate dependent dioxygenase"	0.534917	0.826004
	AMER1	APC membrane recruitment protein 1	0.999996	0.936081
	AMIGO2	adhesion molecule with Ig like domain 2	1.01909	1.00302
	AMMECR1	"Alport syndrome, mental retardation, midface hypoplasia and elliptocytosis chromosomal region gene 1"	0.486683	0.499441
	AMN1	antagonist of mitotic exit network 1 homolog	0.683819	0.588496
	ANAPC16	anaphase promoting complex subunit 16	0.486764	0.477979
	ANAPC5	anaphase promoting complex subunit 5	0.562191	0.456977
	ANKRD1	ankyrin repeat domain 1	0.498224	0.633051
	ANKRD13A	ankyrin repeat domain 13A	0.696859	0.685671

	ANKRD22	ankyrin repeat domain 22	1.83286	1.5803
	ANKRD30B	ankyrin repeat domain 30B	0.863248	0.779758
	AP1S1	adaptor related protein complex 1 sigma 1 subunit	0.712681	0.766289
	AP2S1	adaptor related protein complex 2 sigma 1 subunit	1.22989	1.36345
	AP5S1	adaptor related protein complex 5 sigma 1 subunit	0.456215	0.768152
	APITD1	"apoptosis-inducing, TAF9-like domain 1"	0.815897	0.780221
	AREG	amphiregulin	1.42032	1.32116
	ARF5	ADP ribosylation factor 5	0.739554	0.905469
	ARHGEF19	Rho guanine nucleotide exchange factor 19	0.840479	0.944296
	ARL3	ADP ribosylation factor like GTPase 3	0.606915	0.522059
	ARL5B	ADP ribosylation factor like GTPase 5B	0.799245	0.737761
	ARL6IP5	ADP ribosylation factor like GTPase 6 interacting protein 5	0.510344	0.450079
	ARMC10	armadillo repeat containing 10	0.678404	0.480358
	ARMC2	armadillo repeat containing 2	0.696103	0.782873
	ARPC2	actin related protein 2/3 complex subunit 2	0.827089	0.909638
	ARRDC4	arrestin domain containing 4	0.676729	0.931238
	ARSK	arylsulfatase family member K	1.09553	1.18466
	ARTN	artemin	0.779861	0.876064
	ARV1	"ARV1 homolog, fatty acid homeostasis modulator"	0.443626	0.636535
	ASMTL	acetylserotonin O-methyltransferase-like	0.627943	0.643094
	ASNA1	"arsA arsenite transporter, ATP-binding, homolog 1 (bacterial)"	0.380803	0.471845
	ATAD1	"ATPase family, AAA domain containing 1"	0.789302	0.790079
	ATAD2B	"ATPase family, AAA domain containing 2B"	0.623753	0.47118
	ATOH8	atonal bHLH transcription factor 8	1.53391	1.45938
	ATP23	ATP23 metallopeptidase and ATP synthase assembly factor homolog	0.957749	0.922681
	ATP5A1	"ATP synthase, H+ transporting, mitochondrial F1 complex, alpha subunit 1, cardiac muscle"	0.424851	0.412725
	ATP5B	"ATP synthase, H+ transporting, mitochondrial F1 complex, beta polypeptide"	0.738957	0.734603
	ATP5E	"ATP synthase, H+ transporting, mitochondrial F1 complex, epsilon subunit"	0.553854	0.60387
	ATP5EP2	"ATP synthase, H+ transporting, mitochondrial F1 complex, epsilon subunit pseudogene 2"	1.06228	0.475944
	ATP5F1	"ATP synthase, H+ transporting, mitochondrial Fo complex subunit B1"	0.570357	0.604812
	ATP5G3	"ATP synthase, H+ transporting, mitochondrial Fo complex subunit C3 (subunit 9)"	0.885476	1.00146
	ATP5I	"ATP synthase, H+ transporting, mitochondrial Fo complex subunit E"	0.572728	0.805795
	ATP5L	"ATP synthase, H+ transporting, mitochondrial Fo complex subunit G"	0.641905	0.713396
	ATP6AP1	ATPase H+ transporting accessory protein 1	0.725615	0.747385
	ATPAF2	ATP synthase mitochondrial F1 complex assembly factor 2	0.668871	0.853675
	ATXN7L3B	ataxin 7 like 3B	1.23747	1.29717
	B2M	beta-2-microglobulin	0.660654	0.683885
	B3GALT6	"beta-1,3-galactosyltransferase 6"	0.891879	1.24243
	B4GALT2	"beta-1,4-galactosyltransferase 2"	1.18107	1.36107
	B4GALT5	"beta-1,4-galactosyltransferase 5"	0.648518	0.556421
	BAG1	BCL2 associated athanogene 1	0.383929	0.514251
	BAK1	BCL2 antagonist/killer 1	1.22959	1.49069
	BANF1	barrier to autointegration factor 1	0.576179	0.631429
	BARX2	BARX homeobox 2	1.10383	1.02157
	BASP1	brain abundant membrane attached signal protein 1	0.483084	0.508448
	BBS10	Bardet-Biedl syndrome 10	0.549438	0.635606
	BCAT2	branched chain amino acid transaminase 2	0.627225	0.819078
	BCCIP	BRCA2 and CDKN1A interacting protein	0.802714	0.867996
	BCL11B	B-cell CLL/lymphoma 11B	0.889491	0.847944
	BCL6	B-cell CLL/lymphoma 6	1.04653	0.899591
	BCL7A	BCL tumor suppressor 7A	0.971165	1.052
	BDH1	"3-hydroxybutyrate dehydrogenase, type 1"	0.715878	0.744706
	BDH2	"3-hydroxybutyrate dehydrogenase, type 2"	0.843871	0.866672
	BEND6	BEN domain containing 6	1.01679	1.03956

	BEX5	brain expressed X-linked 5	1.2185	1.64046
	BFAR	bifunctional apoptosis regulator	0.519609	0.486401
	BFSP2	beaded filament structural protein 2	1.60905	1.3469
	BIRC2	baculoviral IAP repeat containing 2	1.50289	1.41292
	BLMH	bleomycin hydrolase	2.51864	2.13864
	BMPER	BMP binding endothelial regulator	1.90746	1.64764
	BOC	"BOC cell adhesion associated, oncogene regulated"	2.16198	1.93153
	BOLA1	bolA family member 1	0.570531	1.00556
	BPHL	biphenyl hydrolase like	0.722898	0.655604
	BRINP1	BMP/retinoic acid inducible neural specific 1	1.72664	1.90507
	BRINP2	BMP/retinoic acid inducible neural specific 2	2.03546	1.60345
	BRIX1	"BRX1, biogenesis of ribosomes"	0.518963	0.56702
	BRK1	"BRICK1, SCAR/WAVE actin nucleating complex subunit"	0.750387	0.79901
	BRSK2	BR serine/threonine kinase 2	1.05141	0.844313
	BTBD10	BTB domain containing 10	0.947219	0.868464
	BTF3	basic transcription factor 3	0.506364	0.487342
	BTN3A1	butyrophilin subfamily 3 member A1	0.701139	0.811308
	BTN3A2	butyrophilin subfamily 3 member A2	1.66846	1.60858
	BZW2	basic leucine zipper and W2 domains 2	1.33163	1.28027
	C12orf4	chromosome 12 open reading frame 4	0.589379	0.697994
	C14orf166	chromosome 14 open reading frame 166	0.363788	0.346326
	C14orf2	chromosome 14 open reading frame 2	0.523541	0.590597
	C15orf59	chromosome 15 open reading frame 59	1.07989	1.23498
	C16orf87	chromosome 16 open reading frame 87	1.76765	1.78206
	C16orf91	chromosome 16 open reading frame 91	0.529916	1.05276
	C19orf48	chromosome 19 open reading frame 48	0.550821	1.02046
	C19orf53	chromosome 19 open reading frame 53	0.508361	0.593913
	C1GALT1C1	C1GALT1 specific chaperone 1	0.504905	0.496875
	C1GALT1C1L	C1GALT1-specific chaperone 1 like	1.17377	1.59184
	C1orf109	chromosome 1 open reading frame 109	0.578077	0.703725
	C1orf123	chromosome 1 open reading frame 123	0.966238	1.10235
	C1orf131	chromosome 1 open reading frame 131	1.49237	1.68842
	C1orf168	chromosome 1 open reading frame 168	1.05566	0.884423
	C1orf50	chromosome 1 open reading frame 50	0.506735	0.660153
	C1orf52	chromosome 1 open reading frame 52	1.30657	1.53613
	C1QBP	complement C1q binding protein	0.842575	0.853417
	C1QTNF6	C1q and tumor necrosis factor related protein 6	1.65012	1.72005
	C20orf196	chromosome 20 open reading frame 196	0.846608	1.17566
	C21orf33	chromosome 21 open reading frame 33	0.576514	0.882266
	C21orf58	chromosome 21 open reading frame 58	0.574076	0.566849
	C21orf91	chromosome 21 open reading frame 91	0.691422	0.713411
	C2orf76	chromosome 2 open reading frame 76	0.748832	0.69391
	C4orf19	chromosome 4 open reading frame 19	1.57704	1.5167
	C4orf3	chromosome 4 open reading frame 3	0.665988	0.811939
	C5orf15	chromosome 5 open reading frame 15	1.65814	1.63619
	C5orf30	chromosome 5 open reading frame 30	0.465594	0.3944
	C6orf141	chromosome 6 open reading frame 141	0.756503	0.897626
	C7orf49	chromosome 7 open reading frame 49	0.651726	0.709833
	C8orf33	chromosome 8 open reading frame 33	0.660074	0.695409
	C9orf69	chromosome 9 open reading frame 69	0.476335	0.79721
	CA13	carbonic anhydrase 13	1.36253	1.04568
	CALM3	calmodulin 3	0.570431	0.485807
	CAMTA1	calmodulin binding transcription activator 1	0.642045	0.662908
	CAPN6	calpain 6	1.02668	1.12836
	CAPZB	capping actin protein of muscle Z-line beta subunit	0.666538	0.682933
	CASP3	caspase 3	0.729553	0.745127
	CASP7	caspase 7	1.81119	1.87049
	CAT	catalase	0.739667	0.642768
	CBR1	carbonyl reductase 1	0.60378	0.707936
	CBWD2	COBW domain containing 2	0.552169	0.479109
	CBX1	chromobox 1	0.608134	0.536891
	CBX2	chromobox 2	1.05339	1.05031
	CBX6	chromobox 6	0.744626	0.895712
	CBY1	"chibby family member 1, beta catenin antagonist"	1.33359	1.26603

	CCDC117	coiled-coil domain containing 117	0.801064	0.602464
	CCDC138	coiled-coil domain containing 138	1.54328	1.27542
	CCDC28B	coiled-coil domain containing 28B	2.65578	2.80182
	CCDC43	coiled-coil domain containing 43	1.47159	1.5781
	CCDC58	coiled-coil domain containing 58	0.382227	0.374341
	CCDC59	coiled-coil domain containing 59	0.410004	0.482381
	CCDC94	coiled-coil domain containing 94	0.459384	0.454246
	CCNA2	cyclin A2	0.683344	0.672626
	CCNB1IP1	cyclin B1 interacting protein 1	1.22499	1.20591
	CCNE1	cyclin E1	0.708192	0.746412
	CCNF	cyclin F	0.4926	0.539965
	CCNG2	cyclin G2	0.662931	0.679027
	CCNYL1	cyclin Y like 1	0.891729	0.683649
	CCSAP	"centriole, cilia and spindle associated protein"	1.0959	1.01218
	CCT2	chaperonin containing TCP1 subunit 2	0.955846	0.94792
	CCT4	chaperonin containing TCP1 subunit 4	0.507679	0.468577
	CCT6P1	chaperonin containing TCP1 subunit 6 pseudogene 1	0.440193	0.479515
	CCT7	chaperonin containing TCP1 subunit 7	0.61167	0.525603
	CCT8	chaperonin containing TCP1 subunit 8	0.516095	0.456799
	CD47	CD47 molecule	1.08686	1.07416
	CD58	CD58 molecule	0.574278	0.608606
	CD83	CD83 molecule	1.92141	1.74713
	CDADC1	cytidine and dCMP deaminase domain containing 1	0.972441	1.19253
	CDC123	cell division cycle 123	0.579892	0.537706
	CDC23	cell division cycle 23	0.89469	0.898371
	CDC37L1	cell division cycle 37 like 1	0.652822	0.581541
	CDC42	cell division cycle 42	0.724433	0.737603
	CDC45	cell division cycle 45	0.95113	0.941068
	CDC6	cell division cycle 6	0.995412	1.05737
	CDCA4	cell division cycle associated 4	0.679391	0.937433
	CDCA7	cell division cycle associated 7	0.780818	0.700558
	CDH10	cadherin 10	1.37135	1.077
	CDIPT	CDP-diacylglycerol--inositol 3-phosphatidyltransferase	0.50783	0.492623
	CDK4	cyclin dependent kinase 4	0.466874	0.512105
	CDK5RAP1	CDK5 regulatory subunit associated protein 1	0.421749	0.452614
	CDKN2AIPNL	CDKN2A interacting protein N-terminal like	0.749947	0.797643
	CDPF1	cysteine rich DPF motif domain containing 1	0.989699	1.29684
	CEBPA-AS1	CEBPA antisense RNA 1 (head to head)	0.653322	0.775162
	CECR5	"cat eye syndrome chromosome region, candidate 5"	0.4713	0.614461
	CENPBD1P1	CENPB DNA-binding domains containing 1 pseudogene 1	1.15072	1.34084
	CENPK	centromere protein K	1.76657	1.73323
	CENPW	centromere protein W	0.460512	0.594347
	CEP78	centrosomal protein 78	0.579872	0.407027
	CETN2	centrin 2	0.67484	0.556184
	CFAP20	cilia and flagella associated protein 20	0.673339	0.71513
	CGA	"glycoprotein hormones, alpha polypeptide"	1.43795	2.15628
	CGREF1	cell growth regulator with EF-hand domain 1	0.532261	0.530638
	CHCHD1	coiled-coil-helix-coiled-coil-helix domain containing 1	0.506416	0.588876
	CHCHD2	coiled-coil-helix-coiled-coil-helix domain containing 2	0.673485	0.788832
	CHCHD4	coiled-coil-helix-coiled-coil-helix domain containing 4	0.454512	0.51827
	CHD1L	chromodomain helicase DNA binding protein 1 like	0.459906	0.425305
	CHDH	choline dehydrogenase	0.460734	0.362754
	CHEK2	checkpoint kinase 2	0.827479	0.90164
	CHGA	chromogranin A	0.921886	0.924259
	CHMP6	charged multivesicular body protein 6	0.715407	1.0806
	CHRNA5	cholinergic receptor nicotinic alpha 5 subunit	0.704088	0.690746
	CHSY1	chondroitin sulfate synthase 1	1.12941	0.935212
	CIDECP	cell death-inducing DFFA-like effector c pseudogene	1.01528	1.24263
	CINP	cyclin dependent kinase 2 interacting protein	0.658014	0.726846

	CISD1	CDGSH iron sulfur domain 1	1.01027	1.0821
	CITED2	Cbp/p300 interacting transactivator with Glu/Asp rich carboxy-terminal domain 2	0.558277	0.687849
	CITED4	Cbp/p300 interacting transactivator with Glu/Asp rich carboxy-terminal domain 4	0.482466	0.696028
	CKLF	chemokine like factor	0.618983	0.705915
	CKS1B	CDC28 protein kinase regulatory subunit 1B	0.59821	0.662221
	CLCN4	chloride voltage-gated channel 4	1.05748	0.823801
	CLDN1	claudin 1	1.59109	2.35224
	CLNS1A	chloride nucleotide-sensitive channel 1A	0.581412	0.683439
	CMAHP	"cytidine monophospho-N-acetylneuraminic acid hydroxylase, pseudogene"	0.825125	1.24412
	CMC2	C-X9-C motif containing 2	0.595229	0.668355
	CMSS1	cms1 ribosomal small subunit homolog (yeast)	1.11261	1.07853
	CNBD2	cyclic nucleotide binding domain containing 2	0.837055	1.27329
	CNBP	CCHC-type zinc finger nucleic acid binding protein	0.47528	0.558615
	CNIH1	cornichon family AMPA receptor auxiliary protein 1	0.527042	0.625035
	CNOT8	CCR4-NOT transcription complex subunit 8	0.78093	0.812067
	COA1	cytochrome c oxidase assembly factor 1 homolog	0.866394	0.834569
	COA3	cytochrome c oxidase assembly factor 3	0.454443	0.58493
	COA4	cytochrome c oxidase assembly factor 4 homolog	0.567114	0.757669
	COA7	cytochrome c oxidase assembly factor 7 (putative)	0.72858	0.930403
	COL27A1	collagen type XXVII alpha 1 chain	1.67382	1.25171
	COMMD2	COMM domain containing 2	1.81109	1.76506
	COMMD7	COMM domain containing 7	1.39645	1.46911
	COMMD9	COMM domain containing 9	1.13099	1.13093
	COPS5	COP9 signalosome subunit 5	1.20623	1.24081
	COPS9	COP9 signalosome subunit 9	1.02218	1.25621
	COQ10A	coenzyme Q10A	0.61573	0.786051
	COQ2	"coenzyme Q2, polyprenyltransferase"	0.51358	0.591104
	COQ3	"coenzyme Q3, methyltransferase"	0.454187	0.564536
	COQ5	"coenzyme Q5, methyltransferase"	1.12072	1.17251
	COTL1	coactosin like F-actin binding protein 1	0.904161	0.881233
	COX14	"COX14, cytochrome c oxidase assembly factor"	0.697269	0.884169
	COX5A	cytochrome c oxidase subunit 5A	0.440628	0.503475
	COX6C	cytochrome c oxidase subunit 6C	0.473092	0.529371
	COX7A2L	cytochrome c oxidase subunit 7A2 like	0.583686	0.527884
	COX7B	cytochrome c oxidase subunit 7B	0.743747	0.832255
	COX7C	cytochrome c oxidase subunit 7C	0.420544	0.504002
	CPNE7	copine 7	0.884544	0.900155
	CRADD	CASP2 and RIPK1 domain containing adaptor with death domain	0.94901	1.09059
	CREG1	cellular repressor of E1A stimulated genes 1	1.06377	1.04923
	CRISPLD1	cysteine rich secretory protein LCCL domain containing 1	2.05979	2.05316
	CRLS1	cardiolipin synthase 1	0.531102	0.667133
	CRNDE	colorectal neoplasia differentially expressed (non-protein coding)	0.437687	0.476426
	CSGALNACT2	chondroitin sulfate N-acetylgalactosaminyltransferase 2	1.09471	0.964817
	CSPG5	chondroitin sulfate proteoglycan 5	1.77322	1.8202
	CTDSPL	CTD small phosphatase like	1.29415	1.31864
	CTH	cystathionine gamma-lyase	1.37027	1.01686
	CTHRC1	collagen triple helix repeat containing 1	1.78111	1.52537
	CTNNAL1	catenin alpha like 1	1.10965	1.06564
	CTNNBIP1	catenin beta interacting protein 1	1.85451	1.90993
	CTSV	cathepsin V	0.653027	0.565642
	CUEDC2	CUE domain containing 2	0.421961	0.40473
	CUTC	cutC copper transporter	1.76035	1.9119
	CXADR	coxsackie virus and adenovirus receptor	1.04079	0.942615
	CXCL11	C-X-C motif chemokine ligand 11	2.02203	2.21208
	CXCL12	C-X-C motif chemokine ligand 12	1.71022	1.52676
	CYB561	cytochrome b561	0.490292	0.599664
	CYB5A	cytochrome b5 type A	0.776375	0.787099
	CYCS	"cytochrome c, somatic"	0.634708	0.683778
	CYP2J2	cytochrome P450 family 2 subfamily J member 2	0.405508	0.386161
	CYP4F22	cytochrome P450 family 4 subfamily F member 22	0.607833	0.711376

	DAD1	defender against cell death 1	0.483024	0.423811
	DANCR	differentiation antagonizing non-protein coding RNA	1.67735	2.07894
	DAP	death associated protein	0.676437	0.692041
	DAZAP2	DAZ associated protein 2	0.572756	0.569066
	DBT	dihydrolipoamide branched chain transacylase E2	1.19273	1.08744
	DCAF12	DDB1 and CUL4 associated factor 12	0.792873	0.751584
	DCAF5	DDB1 and CUL4 associated factor 5	0.49886	0.426772
	DCPS	"decapping enzyme, scavenger"	0.545144	0.580398
	DCTPP1	dCTP pyrophosphatase 1	0.486552	0.575999
	DCUN1D5	defective in cullin neddylation 1 domain containing 5	0.895144	0.894684
	DCXR	dicarbonyl and L-xylulose reductase	0.639569	0.72724
	DDX1	DEAD/H-box helicase 1	0.493408	0.479488
	DDX21	DEAD-box helicase 21	1.0968	1.03752
	DDX47	DEAD-box helicase 47	0.765994	0.743859
	DDX49	DEAD-box helicase 49	2.34706	2.59104
	DEF6	"DEF6, guanine nucleotide exchange factor"	0.674619	0.787547
	DEGS2	"delta 4-desaturase, sphingolipid 2"	1.16509	1.45397
	DENR	density regulated re-initiation and release factor	0.927438	0.880601
	DEPDC1B	DEP domain containing 1B	0.948026	0.751444
	DERA	deoxyribose-phosphate aldolase	0.991452	0.967232
	DFFA	DNA fragmentation factor subunit alpha	0.675709	0.602846
	DHODH	dihydroorotate dehydrogenase (quinone)	0.584332	0.561185
	DHRS13	dehydrogenase/reductase 13	1.78083	1.83441
	DHRS7B	dehydrogenase/reductase 7B	0.519399	0.643293
	DLD	dihydrolipoamide dehydrogenase	0.797122	0.750107
	DLEU1	deleted in lymphocytic leukemia 1	1.11632	0.816194
	DNAAF2	dynein axonemal assembly factor 2	0.627346	0.76001
	DNAH14	dynein axonemal heavy chain 14	0.576577	0.597049
	DNAJA1	DnaJ heat shock protein family (Hsp40) member A1	0.475614	0.472945
	DNAJB11	DnaJ heat shock protein family (Hsp40) member B11	0.904611	0.930958
	DNAJC1	DnaJ heat shock protein family (Hsp40) member C1	0.628416	0.707132
	DNAJC19	DnaJ heat shock protein family (Hsp40) member C19	0.487561	0.535434
	DNMT3B	DNA methyltransferase 3 beta	1.75426	1.6786
	DNPH1	2'-deoxynucleoside 5'-phosphate N-hydrolase 1	0.415004	0.808615
	DPCD	deleted in primary ciliary dyskinesia homolog (mouse)	0.96135	0.905369
	DPH2	DPH2 homolog	0.873147	0.947896
	DPY30	"dpy-30, histone methyltransferase complex regulatory subunit"	0.745031	0.681298
	DSCAM-AS1	DSCAM antisense RNA 1	1.79876	1.65099
	DSCC1	DNA replication and sister chromatid cohesion 1	1.65166	1.54324
	DSCR8	Down syndrome critical region 8	1.86764	1.26283
	DTD1	D-tyrosyl-tRNA deacylase 1	0.439774	0.362775
	DTWD1	DTW domain containing 1	0.501435	0.424841
	DTWD2	DTW domain containing 2	0.732857	0.575587
	DTX4	deltex E3 ubiquitin ligase 4	1.46128	0.89692
	DTYMK	deoxythymidylate kinase	0.550339	0.636515
	DUSP2	dual specificity phosphatase 2	0.861033	1.02365
	DUSP4	dual specificity phosphatase 4	2.05306	1.92245
	DUSP5	dual specificity phosphatase 5	1.16304	0.974547
	DYM	dymeclin	1.72405	1.65591
	DYNLL1	dynein light chain LC8-type 1	0.629238	0.640647
	DYRK2	dual specificity tyrosine phosphorylation regulated kinase 2	0.937423	0.706077
	EAPP	E2F associated phosphoprotein	0.426617	0.475068
	EBAG9	"estrogen receptor binding site associated, antigen, 9"	0.596474	0.638776
	EBNA1BP2	EBNA1 binding protein 2	0.637167	0.63253
	EBP	emopamil binding protein (sterol isomerase)	0.72739	0.726358
	EBPL	emopamil binding protein like	0.759841	0.707649
	ECHS1	"enoyl-CoA hydratase, short chain 1"	0.423954	0.434189

	EDF1	endothelial differentiation related factor 1	0.499046	0.698612
	EDRF1	erythroid differentiation regulatory factor 1	0.87891	0.947828
	EEF1A1P9	eukaryotic translation elongation factor 1 alpha 1 pseudogene 9	0.580221	0.572346
	EEF1AKMT1	eukaryotic translation elongation factor 1 alpha lysine methyltransferase 1	0.694379	0.670003
	EEF1B2	eukaryotic translation elongation factor 1 beta 2	0.68232	0.665074
	EEF1G	eukaryotic translation elongation factor 1 gamma	0.50351	0.394335
	EFNB3	ephrin B3	0.713137	0.530624
	EGR3	early growth response 3	2.35585	2.26571
	EHF	ETS homologous factor	0.793912	0.539603
	EI24	"EI24, autophagy associated transmembrane protein"	1.58026	1.59393
	EID1	EP300 interacting inhibitor of differentiation 1	1.13643	1.31191
	EID2	EP300 interacting inhibitor of differentiation 2	1.02888	1.32895
	EID2B	EP300 interacting inhibitor of differentiation 2B	0.663137	1.24476
	EIF2B1	eukaryotic translation initiation factor 2B subunit alpha	0.842259	0.955928
	EIF2B2	eukaryotic translation initiation factor 2B subunit beta	0.438912	0.472013
	EIF2B3	eukaryotic translation initiation factor 2B subunit gamma	0.854297	0.912875
	EIF2D	eukaryotic translation initiation factor 2D	0.89687	0.852116
	EIF2S2	eukaryotic translation initiation factor 2 subunit beta	0.929427	0.970642
	EIF3E	eukaryotic translation initiation factor 3 subunit E	0.693977	0.627349
	EIF3I	eukaryotic translation initiation factor 3 subunit I	0.502812	0.370749
	EIF3J	eukaryotic translation initiation factor 3 subunit J	0.920679	0.788568
	EIF4A1	eukaryotic translation initiation factor 4A1	0.506091	0.467426
	EIF4A3	eukaryotic translation initiation factor 4A3	0.444924	0.555671
	EIF4E2	eukaryotic translation initiation factor 4E family member 2	1.81313	1.83072
	EIF5A	eukaryotic translation initiation factor 5A	0.682438	0.762379
	EIF5AL1	eukaryotic translation initiation factor 5A-like 1	0.937528	0.856409
	EIF6	eukaryotic translation initiation factor 6	0.77994	0.930112
	ELK1	"ELK1, ETS transcription factor"	0.774281	0.883002
	ELOVL2	ELOVL fatty acid elongase 2	0.649693	0.519209
	ELOVL5	ELOVL fatty acid elongase 5	0.835545	0.688405
	ELP2	elongator acetyltransferase complex subunit 2	0.751258	0.807974
	ELP4	elongator acetyltransferase complex subunit 4	0.670908	0.728065
	ELP6	elongator acetyltransferase complex subunit 6	0.412246	0.534597
	EMC4	ER membrane protein complex subunit 4	0.519872	0.416206
	EMID1	EMI domain containing 1	0.485272	0.647797
	ENHO	energy homeostasis associated	1.61978	1.62355
	ENOPH1	enolase-phosphatase 1	0.606649	0.586319
	ENSA	endosulfine alpha	0.669224	0.617762
	ENTPD3-AS1	ENTPD3 antisense RNA 1	0.509316	0.639276
	ERAL1	Era like 12S mitochondrial rRNA chaperone 1	0.638027	0.704868
	ERCC2	"ERCC excision repair 2, TFIIH core complex helicase subunit"	0.760713	0.555621
	ERGIC3	ERGIC and golgi 3	0.703577	0.774335
	ERH	enhancer of rudimentary homolog (Drosophila)	0.573351	0.58719
	ERP27	endoplasmic reticulum protein 27	1.63098	2.08328
	ERRFI1	ERBB receptor feedback inhibitor 1	0.525362	0.709608
	ESD	esterase D	0.528035	0.538701
	ETFA	electron transfer flavoprotein alpha subunit	0.591116	0.527411
	ETFDH	electron transfer flavoprotein dehydrogenase	1.6454	1.51574
	ETNK2	ethanolamine kinase 2	1.11182	1.05306
	ETV4	ETS variant 4	2.98963	2.98716
	ETV5	ETS variant 5	1.71214	2.63838
	EXO1	exonuclease 1	0.586883	0.616257
	EXOC3-AS1	EXOC3 antisense RNA 1	0.65992	0.935699
	EXOSC2	exosome component 2	0.584958	0.614102
	EXOSC4	exosome component 4	0.624955	1.19043
	EXTL2	exostosin like glycosyltransferase 2	0.709238	0.541277
	FAAP100	Fanconi anemia core complex associated protein 100	0.924334	1.1769
	FABP5	fatty acid binding protein 5	0.582434	0.764601

	FAH	fumarylacetoacetate hydrolase	0.553601	0.478695
	FAHD1	fumarylacetoacetate hydrolase domain containing 1	0.636851	0.901924
	FAHD2CP	"fumarylacetoacetate hydrolase domain containing 2C, pseudogene"	0.698245	0.689497
	FAM104B	family with sequence similarity 104 member B	0.817937	0.900684
	FAM109A	family with sequence similarity 109 member A	0.510703	0.628693
	FAM117B	family with sequence similarity 117 member B	0.800434	0.544344
	FAM129B	family with sequence similarity 129 member B	0.567133	0.628176
	FAM136A	family with sequence similarity 136 member A	1.77743	1.89115
	FAM136BP	"family with sequence similarity 136 member B, pseudogene"	1.19572	0.919494
	FAM162A	family with sequence similarity 162 member A	0.512759	0.651472
	FAM168B	family with sequence similarity 168 member B	0.732542	0.642111
	FAM210B	family with sequence similarity 210 member B	0.494677	0.455537
	FAM234A	family with sequence similarity 234 member A	1.20283	1.24764
	FAM26F	family with sequence similarity 26 member F	0.969095	1.12822
	FAM32A	family with sequence similarity 32 member A	0.887943	0.919503
	FAM57A	family with sequence similarity 57 member A	1.08004	1.04424
	FAM64A	family with sequence similarity 64 member A	0.685687	0.797623
	FAM84B	family with sequence similarity 84 member B	0.978182	0.9469
	FANCE	Fanconi anemia complementation group E	0.788509	0.896597
	FARS2	"phenylalanyl-tRNA synthetase 2, mitochondrial"	0.604479	0.630769
	FARSB	phenylalanyl-tRNA synthetase beta subunit	0.589758	0.56169
	FBXO45	F-box protein 45	1.04396	1.00334
	FBXW9	F-box and WD repeat domain containing 9	1.83408	2.26133
	FCMR	Fc fragment of IgM receptor	0.62598	0.67407
	FDFT1	farnesyl-diphosphate farnesyltransferase 1	0.83121	0.616238
	FERMT2	fermitin family member 2	0.511322	0.456936
	FGFBP2	fibroblast growth factor binding protein 2	2.90166	2.19779
	FGFBP3	fibroblast growth factor binding protein 3	1.23721	1.87457
	FGFR2	fibroblast growth factor receptor 2	0.947844	0.935403
	FH	fumarate hydratase	0.517003	0.568854
	FIBP	FGF1 intracellular binding protein	0.762325	0.744858
	FIS1	"fission, mitochondrial 1"	0.477	0.64285
	FJX1	four jointed box 1	0.673003	0.786922
	FKBP3	FK506 binding protein 3	0.598451	0.547113
	FKBPL	FK506 binding protein like	0.676845	0.921371
	FLT4	fms related tyrosine kinase 4	0.967415	1.2875
	FMNL2	formin like 2	0.941409	0.829786
	FRAT2	frequently rearranged in advanced T-cell lymphomas 2	1.26584	1.50653
	FSCN1	fascin actin-bundling protein 1	0.883641	0.88007
	FTO	fat mass and obesity associated	0.807692	0.719746
	FUNDC2	FUN14 domain containing 2	0.465033	0.455508
	FUOM	fucose mutarotase	0.512164	0.843375
	FYTTD1	forty-two-three domain containing 1	1.3472	1.19733
	FZD6	frizzled class receptor 6	0.513988	0.416162
	FZD9	frizzled class receptor 9	0.881945	1.14293
	GABPB1	GA binding protein transcription factor beta subunit 1	0.78914	0.806187
	GALNT14	polypeptide N-acetylgalactosaminyltransferase 14	0.608762	0.488799
	GAR1	GAR1 ribonucleoprotein	0.712978	0.750716
	GATA4	GATA binding protein 4	0.816668	0.487858
	GBX2	gastrulation brain homeobox 2	0.911229	0.883168
	GCAT	glycine C-acetyltransferase	0.816279	0.888683
	GCDH	glutaryl-CoA dehydrogenase	0.848594	0.735966
	GDF1	growth differentiation factor 1	0.939212	0.785672
	GEMIN2	gem nuclear organelle associated protein 2	2.09195	2.368
	GEMIN6	gem nuclear organelle associated protein 6	0.527315	0.549781
	GFOD1	glucose-fructose oxidoreductase domain containing 1	0.655897	0.677425
	GGACT	gamma-glutamylamine cyclotransferase	0.730741	1.16279
	GGCT	gamma-glutamylcyclotransferase	2.08826	2.14783
	GGCX	gamma-glutamyl carboxylase	0.84325	0.896333
	GGH	gamma-glutamyl hydrolase	0.863208	0.719927
	GID8	GID complex subunit 8 homolog	0.555707	0.617716

	GINS1	GINS complex subunit 1	0.825391	0.768927
	GINS2	GINS complex subunit 2	1.68133	1.7959
	GJA1	gap junction protein alpha 1	4.11427	3.86025
	GLO1	glyoxalase I	0.596599	0.510487
	GLP1R	glucagon like peptide 1 receptor	0.964153	0.86756
	GLRX2	glutaredoxin 2	0.493896	0.530772
	GLRX3	glutaredoxin 3	0.917546	1.0863
	GLRX5	glutaredoxin 5	0.741695	0.736694
	GLUD2	glutamate dehydrogenase 2	0.54971	0.439276
	GMDS	"GDP-mannose 4,6-dehydratase"	0.567183	0.508126
	GNA12	G protein subunit alpha 12	0.878071	0.730274
	GNAI1	G protein subunit alpha i1	0.590945	0.485692
	GNG10	G protein subunit gamma 10	0.677874	0.564374
	GNG12	G protein subunit gamma 12	0.792578	0.595368
	GOLGA7	golgin A7	1.14222	0.971833
	GPC5	glypican 5	0.529826	0.4271
	GPR143	G protein-coupled receptor 143	0.864325	1.13005
	GPR176	G protein-coupled receptor 176	0.911931	1.19186
	GPR37	G protein-coupled receptor 37	1.6004	1.4605
	GPT2	glutamic--pyruvic transaminase 2	1.20088	1.06805
	GRHPR	glyoxylate and hydroxypyruvate reductase	0.445495	0.490305
	GRK6	G protein-coupled receptor kinase 6	0.718213	0.990066
	GRPR	gastrin releasing peptide receptor	2.54854	2.69536
	GSG1L	GSG1 like	3.08116	2.83152
	GSTA4	glutathione S-transferase alpha 4	1.77556	2.01732
	GSTO1	glutathione S-transferase omega 1	0.382099	0.419248
	GSTO2	glutathione S-transferase omega 2	0.4087	0.479226
	GTF2F2	general transcription factor IIF subunit 2	0.413409	0.496262
	GTF2H5	general transcription factor IIH subunit 5	0.482877	0.661587
	GTF3A	general transcription factor IIIA	0.639956	0.603777
	GTF3C4	general transcription factor IIIC subunit 4	0.760099	0.704617
	GUCY1A2	guanylate cyclase 1 soluble subunit alpha 2	0.645431	0.613243
	H2AFJ	H2A histone family member J	0.688296	1.08134
	H2AFY	H2A histone family member Y	1.35164	1.31266
	H2AFY2	H2A histone family member Y2	0.758477	0.819214
	H3F3A	H3 histone family member 3A	0.858372	1.01209
	H3F3AP4	"H3 histone, family 3A, pseudogene 4"	0.994267	0.877585
	HACD1	3-hydroxyacyl-CoA dehydratase 1	1.31717	1.5829
	HACL1	2-hydroxyacyl-CoA lyase 1	1.08995	1.17999
	HAT1	histone acetyltransferase 1	0.915354	0.875117
	HAUS4	HAUS augmin like complex subunit 4	0.81204	0.852708
	HDDC2	HD domain containing 2	0.704719	0.628219
	HENMT1	HEN1 methyltransferase homolog 1	1.27558	1.38603
	HERC3	HECT and RLD domain containing E3 ubiquitin protein ligase 3	0.458215	0.557969
	HGD	"homogentisate 1,2-dioxygenase"	1.31709	0.994539
	HIBCH	3-hydroxyisobutyryl-CoA hydrolase	0.842222	0.631806
	HIKESHI	"Hikeshi, heat shock protein nuclear import factor"	0.467457	0.541752
	HINT1	histidine triad nucleotide binding protein 1	0.531404	0.618637
	HINT3	histidine triad nucleotide binding protein 3	0.862346	0.844222
	HIST1H2AC	histone cluster 1 H2A family member c	1.2603	1.30951
	HIST2H2BE	histone cluster 2 H2B family member e	1.19281	1.28682
	HIVEP3	human immunodeficiency virus type I enhancer binding protein 3	1.71264	1.48377
	HLA-A	"major histocompatibility complex, class I, A"	0.642678	0.723941
	HMBS	hydroxymethylbilane synthase	0.562652	0.666744
	HMGB1	high mobility group box 1	0.862235	0.760277
	HMGCS1	3-hydroxy-3-methylglutaryl-CoA synthase 1	0.995823	0.765179
	HMGCS2	3-hydroxy-3-methylglutaryl-CoA synthase 2	1.89403	0.988791
	HMGN1	high mobility group nucleosome binding domain 1	0.671758	0.650796
	HMGN2	high mobility group nucleosomal binding domain 2	0.431841	0.559308
	HMGN3	high mobility group nucleosomal binding domain 3	1.75435	1.77512
	HMOX2	heme oxygenase 2	0.981092	1.10676
	HNRNPC	heterogeneous nuclear ribonucleoprotein C (C1/C2)	0.47602	0.42311
	HNRNPDL	heterogeneous nuclear ribonucleoprotein D like	0.624528	0.737366

	HOMER2	homer scaffolding protein 2	1.61365	1.51969
	HOPX	HOP homeobox	1.87771	1.27803
	HOXB3	homeobox B3	0.917468	0.808306
	HOXB6	homeobox B6	1.8322	2.04239
	HOXB-AS3	HOXB cluster antisense RNA 3	2.07946	1.9725
	HPDL	4-hydroxyphenylpyruvate dioxygenase like	0.801218	1.19059
	HPGD	hydroxyprostaglandin dehydrogenase 15-(NAD)	2.94784	2.59741
	HSBP1	heat shock factor binding protein 1	0.545827	0.597753
	HSD17B10	hydroxysteroid 17-beta dehydrogenase 10	0.404017	0.437113
	HSD17B8	hydroxysteroid 17-beta dehydrogenase 8	1.0486	1.21188
	HSP90B1	heat shock protein 90 beta family member 1	0.780005	0.61235
	HSPA5	heat shock protein family A (Hsp70) member 5	0.461655	0.437272
	HSPB11	heat shock protein family B (small) member 11	0.468481	0.371903
	ID4	"inhibitor of DNA binding 4, HLH protein"	1.1042	1.28042
	IDH2	"isocitrate dehydrogenase (NADP(+)) 2, mitochondrial"	0.358842	0.464326
	IDI1	isopentenyl-diphosphate delta isomerase 1	0.710248	0.57221
	IFI27L1	interferon alpha inducible protein 27 like 1	0.498304	0.623056
	IFI30	"IFI30, lysosomal thiol reductase"	1.18563	1.69327
	IFI44	interferon induced protein 44	1.07807	1.79376
	IFI44L	interferon induced protein 44 like	0.595391	1.33428
	IFI6	interferon alpha inducible protein 6	0.854428	1.67039
	IFIT1	interferon induced protein with tetratricopeptide repeats 1	0.683847	1.46504
	IFITM1	interferon induced transmembrane protein 1	0.866949	1.27411
	IFRD2	interferon related developmental regulator 2	0.559552	0.745983
	IFT22	intraflagellar transport 22	0.595712	0.628508
	IFT57	intraflagellar transport 57	0.619088	0.594426
	IL10RB	interleukin 10 receptor subunit beta	1.09275	1.10894
	IL17RB	interleukin 17 receptor B	0.884077	0.736462
	IL19	interleukin 19	3.06232	2.39884
	IL20	interleukin 20	1.37049	1.13747
	IL23R	interleukin 23 receptor	0.901047	0.643077
	IL24	interleukin 24	2.76179	2.61736
	ILF2	interleukin enhancer binding factor 2	0.778354	0.768432
	ILF3-AS1	ILF3 antisense RNA 1 (head to head)	0.605486	0.965656
	IMP3	"IMP3, U3 small nucleolar ribonucleoprotein"	1.2763	1.63331
	IMPA2	inositol monophosphatase 2	0.564067	0.582915
	IMPDH2	inosine monophosphate dehydrogenase 2	0.480604	0.573689
	ING4	inhibitor of growth family member 4	1.19538	1.21837
	INPP4B	inositol polyphosphate-4-phosphatase type II B	1.41143	1.08739
	INPP5A	inositol polyphosphate-5-phosphatase A	0.67894	0.574134
	INSIG1	insulin induced gene 1	1.83312	1.73253
	IQCJ-SCHIP1	IQCJ-SCHIP1 readthrough	1.07634	0.824338
	IRX3	iroquois homeobox 3	0.704955	0.768635
	ISCA1	iron-sulfur cluster assembly 1	0.603147	0.595407
	ISG20L2	interferon stimulated exonuclease gene 20 like 2	0.800567	0.759151
	ISOC2	isochorismatase domain containing 2	0.578063	0.943153
	ITM2B	integral membrane protein 2B	0.455403	0.385767
	IVNS1ABP	influenza virus NS1A binding protein	0.500592	0.379788
	JAGN1	jagunal homolog 1	0.901859	1.05076
	JHDM1D-AS1	JHDM1D antisense RNA 1 (head to head)	1.91105	2.28899
	JPH2	junctophilin 2	1.83951	1.59197
	JPX	"JPX transcript, XIST activator (non-protein coding)"	0.797853	0.917472
	KANSL2	KAT8 regulatory NSL complex subunit 2	0.647085	0.758256
	KATNA1	katanin catalytic subunit A1	0.442418	0.585361
	KBTBD7	kelch repeat and BTB domain containing 7	0.400185	0.420395
	KCNAB1	potassium voltage-gated channel subfamily A member regulatory beta subunit 1	2.691	1.84939
	KCNK2	potassium two pore domain channel subfamily K member 2	1.34367	0.902705
	KCNK5	potassium two pore domain channel subfamily K member 5	0.424791	0.385622
	KCNMA1	potassium calcium-activated channel subfamily M alpha 1	1.55427	1.35738

	KCTD20	potassium channel tetramerization domain containing 20	0.59754	0.434534
	KCTD3	potassium channel tetramerization domain containing 3	0.882766	0.829573
	KCTD5	potassium channel tetramerization domain containing 5	0.425124	0.492212
	KCTD9	potassium channel tetramerization domain containing 9	0.999242	0.863835
	KDM7A	lysine demethylase 7A	1.0876	0.962187
	KIAA0101	KIAA0101	1.25341	1.39906
	KIAA1586	KIAA1586	0.802731	0.769537
	KIAA2013	KIAA2013	0.458529	0.66025
	KIF6	kinesin family member 6	0.904727	0.902278
	KMT5C	lysine methyltransferase 5C	0.564812	0.649637
	KNOP1	lysine rich nucleolar protein 1	0.996013	0.893312
	KRT16	keratin 16	1.41614	1.42172
	KRT2	keratin 2	1.2364	1.21345
	KRT6C	keratin 6C	1.27008	1.92117
	L3MBTL2	L3MBTL2 polycomb repressive complex 1 subunit	0.91279	0.965081
	LAGE3	L antigen family member 3	0.51342	0.72033
	LAMTOR3	"late endosomal/lysosomal adaptor, MAPK and MTOR activator 3"	0.519875	0.638155
	LANCL1	LanC like 1	0.596777	0.482519
	LAP3	leucine aminopeptidase 3	0.654389	0.76435
	LAPTM4B	lysosomal protein transmembrane 4 beta	1.26545	1.18734
	LAS1L	"LAS1 like, ribosome biogenesis factor"	0.473209	0.451075
	LDLRAP1	low density lipoprotein receptor adaptor protein 1	0.541318	0.537152
	LDOC1	leucine zipper down-regulated in cancer 1	0.909283	0.882885
	LDOC1L	leucine zipper down-regulated in cancer 1 like	1.00147	0.970258
	LEMD2	LEM domain containing 2	0.483313	0.472819
	LGI2	leucine rich repeat LGI family member 2	2.18862	2.00029
	LGMN	legumain	0.997135	0.849279
	LIMA1	LIM domain and actin binding 1	0.590325	0.584341
	LINC00152	long intergenic non-protein coding RNA 152	0.642621	0.845507
	LINC00205	long intergenic non-protein coding RNA 205	0.510607	0.541599
	LINC00473	long intergenic non-protein coding RNA 473	4.3032	5.28493
	LINC00493	long intergenic non-protein coding RNA 493	0.794676	0.993629
	LINC00998	long intergenic non-protein coding RNA 998	0.915797	0.942618
	LINC01550	long intergenic non-protein coding RNA 1550	1.32543	1.57637
	LINC01560	long intergenic non-protein coding RNA 1560	0.9892	1.12458
	LMNB1	lamin B1	0.627999	0.630477
	LMO4	LIM domain only 4	1.21488	1.10918
	LOC100288748	uncharacterized LOC100288748	0.514374	0.824425
	LOC728554	THO complex 3 pseudogene	0.691449	0.649272
	LOC93622	Morf4 family associated protein 1 like 1 pseudogene	0.617748	0.775301
	LRRC10B	leucine rich repeat containing 10B	1.33852	1.43715
	LRRC58	leucine rich repeat containing 58	0.529406	0.357706
	LRRC69	leucine rich repeat containing 69	0.555951	0.606239
	LRRC75A	leucine rich repeat containing 75A	0.828751	0.822573
	LRRC75A-AS1	LRRC75A antisense RNA 1	0.730571	0.859008
	LRRN1	leucine rich repeat neuronal 1	1.58509	1.41155
	LSM10	"LSM10, U7 small nuclear RNA associated"	0.568817	0.730227
	LSM11	"LSM11, U7 small nuclear RNA associated"	0.573773	0.53311
	LSM2	"LSM2 homolog, U6 small nuclear RNA and mRNA degradation associated"	0.576788	0.679191
	LSM5	"LSM5 homolog, U6 small nuclear RNA and mRNA degradation associated"	0.556302	0.584585
	LSM7	"LSM7 homolog, U6 small nuclear RNA and mRNA degradation associated"	0.610844	0.982496
	LUC7L	LUC7 like	0.729032	0.973398
	LUC7L2	"LUC7 like 2, pre-mRNA splicing factor"	0.803454	0.757396
	LXN	latexin	0.957785	0.959065
	LYAR	Ly1 antibody reactive	0.775255	0.806391
	LYRM4	LYR motif containing 4	0.816561	0.847357
	LYSMD2	LysM domain containing 2	0.879903	1.02031
	LZIC	leucine zipper and CTNNBIP1 domain containing	1.51983	1.32526

	LZTFL1	leucine zipper transcription factor like 1	0.597137	0.686555
	MAD2L1	MAD2 mitotic arrest deficient-like 1 (yeast)	0.684397	0.683566
	MAFB	MAF bZIP transcription factor B	1.68057	1.74892
	MAFG-AS1	MAFG antisense RNA 1 (head to head)	0.488091	0.758365
	MAGEF1	MAGE family member F1	0.775277	1.0272
	MAGEH1	MAGE family member H1	1.16952	1.36045
	MAK16	MAK16 homolog	0.777235	0.79678
	MAML3	mastermind like transcriptional coactivator 3	1.17245	0.753546
	MANEAL	mannosidase endo-alpha like	1.09	1.09727
	MANF	mesencephalic astrocyte derived neurotrophic factor	0.454652	0.481426
	MAP3K8	mitogen-activated protein kinase kinase kinase 8	0.80049	1.05051
	MAP6D1	MAP6 domain containing 1	1.64809	1.45758
	MAPK14	mitogen-activated protein kinase 14	0.578104	0.456541
	MAPK6	mitogen-activated protein kinase 6	0.93147	0.789119
	MARC1	mitochondrial amidoxime reducing component 1	0.739496	0.634046
	MARCKSL1	MARCKS like 1	1.18066	1.31506
	MARK3	microtubule affinity regulating kinase 3	0.541086	0.708198
	MARVELD1	MARVEL domain containing 1	0.787191	0.941691
	MARVELD2	MARVEL domain containing 2	0.848956	0.83838
	MAZ	MYC associated zinc finger protein	1.56603	1.75118
	MB21D2	Mab-21 domain containing 2	1.01576	1.17096
	MBD4	"methyl-CpG binding domain 4, DNA glycosylase"	0.679262	0.740153
	MBLAC2	metallo-beta-lactamase domain containing 2	0.798222	0.920713
	MCAT	malonyl-CoA-acyl carrier protein transacylase	0.383907	0.533893
	MCCC1	methylcrotonoyl-CoA carboxylase 1	0.606684	0.691066
	MCCC2	methylcrotonoyl-CoA carboxylase 2	0.928812	0.924177
	MCEE	methylmalonyl-CoA epimerase	0.841898	0.914268
	MCL1	BCL2 family apoptosis regulator	0.514158	0.547684
	MCM10	minichromosome maintenance 10 replication initiation factor	0.658391	0.655249
	MCMBP	minichromosome maintenance complex binding protein	0.539588	0.420955
	MDH1	malate dehydrogenase 1	0.483648	0.507619
	MECR	mitochondrial trans-2-enoyl-CoA reductase	0.629844	0.756609
	MED20	mediator complex subunit 20	0.783132	0.800067
	MED28	mediator complex subunit 28	0.537528	0.58869
	MEST	mesoderm specific transcript	2.28148	2.02826
	METTL21A	methyltransferase like 21A	1.81833	1.57057
	METTL8	methyltransferase like 8	0.803202	0.731764
	METTL9	methyltransferase like 9	0.619183	0.505024
	MFAP1	microfibrillar associated protein 1	0.40971	0.392112
	MFAP2	microfibrillar associated protein 2	1.55689	1.53872
	MFSD2A	major facilitator superfamily domain containing 2A	1.167	0.806571
	MGP	matrix Gla protein	0.989572	0.94395
	MINOS1	mitochondrial inner membrane organizing system 1	0.553804	0.786255
	MIPEP	mitochondrial intermediate peptidase	0.710182	0.782334
	MIR4435-2HG	MIR4435-2 host gene	0.680475	0.642404
	MIR99AHG	mir-99a-let-7c cluster host gene	0.706262	0.866057
	MIS18A	MIS18 kinetochore protein A	0.390648	0.472706
	MKKS	McKusick-Kaufman syndrome	0.509373	0.481629
	MLF1	myeloid leukemia factor 1	1.03219	0.903088
	MLXIPL	MLX interacting protein like	0.650685	0.897178
	MMAA	methylmalonic aciduria (cobalamin deficiency) cblA type	0.736563	1.01764
	MMAB	methylmalonic aciduria (cobalamin deficiency) cblB type	0.698355	0.816792
	MMADHC	"methylmalonic aciduria and homocystinuria, cblD type"	0.552518	0.585056
	MMP15	matrix metallopeptidase 15	0.898084	0.856742
	MMP16	matrix metallopeptidase 16	1.33017	0.960805
	MNS1	meiosis specific nuclear structural 1	0.840035	0.758889
	MNX1-AS1	MNX1 antisense RNA 1 (head to head)	0.688844	0.870034
	MOAP1	modulator of apoptosis 1	0.358154	0.435341
	MOCS2	molybdenum cofactor synthesis 2	1.286	1.24044
	MORF4L2	mortality factor 4 like 2	1.31419	1.32475
	MORN2	MORN repeat containing 2	1.01867	0.929174

	MPC1	mitochondrial pyruvate carrier 1	0.595465	0.512531
	MPLKIP	M-phase specific PLK1 interacting protein	0.939126	1.25982
	MPST	mercaptopyruvate sulfurtransferase	0.954081	1.3583
	MPV17	"MPV17, mitochondrial inner membrane protein"	0.799041	0.913382
	MPV17L2	MPV17 mitochondrial inner membrane protein like 2	0.55069	0.580507
	MPZL2	myelin protein zero like 2	0.733585	0.887167
	MRFAP1L1	Morf4 family associated protein 1 like 1	0.361048	0.479952
	MRPL1	mitochondrial ribosomal protein L1	0.613311	0.6288
	MRPL11	mitochondrial ribosomal protein L11	0.561701	0.673595
	MRPL12	mitochondrial ribosomal protein L12	0.508657	0.795649
	MRPL13	mitochondrial ribosomal protein L13	0.515309	0.464846
	MRPL14	mitochondrial ribosomal protein L14	1.09525	1.31287
	MRPL16	mitochondrial ribosomal protein L16	0.6387	0.765245
	MRPL17	mitochondrial ribosomal protein L17	0.790089	1.02674
	MRPL21	mitochondrial ribosomal protein L21	0.620408	0.711494
	MRPL22	mitochondrial ribosomal protein L22	0.41355	0.520083
	MRPL24	mitochondrial ribosomal protein L24	0.506235	0.53577
	MRPL27	mitochondrial ribosomal protein L27	0.756929	0.97082
	MRPL3	mitochondrial ribosomal protein L3	0.752811	0.771381
	MRPL33	mitochondrial ribosomal protein L33	0.747995	0.79456
	MRPL36	mitochondrial ribosomal protein L36	0.541391	0.810266
	MRPL37	mitochondrial ribosomal protein L37	0.844301	0.91952
	MRPL42	mitochondrial ribosomal protein L42	0.558932	0.591286
	MRPL50	mitochondrial ribosomal protein L50	0.727963	0.787498
	MRPL57	mitochondrial ribosomal protein L57	0.811779	1.13103
	MRPL58	mitochondrial ribosomal protein L58	0.589747	0.704769
	MRPS15	mitochondrial ribosomal protein S15	0.589601	0.631072
	MRPS23	mitochondrial ribosomal protein S23	0.403958	0.480255
	MRPS24	mitochondrial ribosomal protein S24	0.555455	0.665775
	MRPS26	mitochondrial ribosomal protein S26	0.590285	0.855188
	MRPS33	mitochondrial ribosomal protein S33	0.401031	0.459406
	MS4A15	membrane spanning 4-domains A15	1.42859	0.848195
	MSANTD3	Myb/SANT DNA binding domain containing 3	1.03394	1.14267
	MSH2	mutS homolog 2	0.453754	0.355227
	MSL3P1	male-specific lethal 3 homolog (Drosophila) pseudogene 1	1.08888	1.21766
	MT1X	metallothionein 1X	0.426134	0.685788
	MTCH2	mitochondrial carrier 2	0.643666	0.688932
	MTERF3	mitochondrial transcription termination factor 3	0.452698	0.582238
	MTF2	metal response element binding transcription factor 2	0.620632	0.58535
	MTG1	mitochondrial ribosome associated GTPase 1	0.930824	1.10945
	MTHFD2	"methylenetetrahydrofolate dehydrogenase (NADP+ dependent) 2, methenyltetrahydrofolate cyclohydrolase"	0.67394	0.578727
	MTHFD2L	methylenetetrahydrofolate dehydrogenase (NADP+ dependent) 2-like	0.589263	0.741144
	MTMR2	myotubularin related protein 2	0.910336	0.726056
	MTRF1	mitochondrial translational release factor 1	0.447792	0.619521
	MUCL1	mucin like 1	1.29882	0.915705
	MUM1L1	MUM1 like 1	1.46894	1.51818
	MYBL2	MYB proto-oncogene like 2	2.03609	2.14457
	MYC	v-myc avian myelocytomatosis viral oncogene homolog	0.888765	0.900107
	MYCBP	MYC binding protein	0.834701	0.780983
	MYL6	myosin light chain 6	0.876249	0.994649
	MYO3B	myosin IIIB	1.78755	1.71407
	NAE1	NEDD8 activating enzyme E1 subunit 1	0.493453	0.473918
	NANP	N-acetylneuraminic acid phosphatase	0.857126	0.883009
	NARS2	"asparaginyl-tRNA synthetase 2, mitochondrial (putative)"	0.792649	0.687521
	NASP	nuclear autoantigenic sperm protein	0.889616	0.865998
	NAT1	N-acetyltransferase 1	0.828996	1.0541
	NAT14	N-acetyltransferase 14 (putative)	0.436621	0.755597
	NAXE	NAD(P)HX epimerase	0.699496	0.682152
	NCBP2-AS2	NCBP2 antisense RNA 2 (head to head)	0.585973	1.03664

	NCR3LG1	natural killer cell cytotoxicity receptor 3 ligand 1	0.504336	0.427074
	NDC1	NDC1 transmembrane nucleoporin	0.581386	0.415238
	NDRG2	NDRG family member 2	1.06254	0.885938
	NDUFA1	NADH:ubiquinone oxidoreductase subunit A1	0.481754	0.626363
	NDUFA10	NADH:ubiquinone oxidoreductase subunit A10	0.587629	0.742002
	NDUFA4	"NDUFA4, mitochondrial complex associated"	0.620783	0.591739
	NDUFA9	NADH:ubiquinone oxidoreductase subunit A9	0.482408	0.51758
	NDUFAB1	NADH:ubiquinone oxidoreductase subunit AB1	0.596311	0.692596
	NDUFAF2	NADH:ubiquinone oxidoreductase complex assembly factor 2	0.783876	0.745836
	NDUFAF8	NADH:ubiquinone oxidoreductase complex assembly factor 8	0.438455	0.719152
	NDUFB1	NADH:ubiquinone oxidoreductase subunit B1	0.794603	0.958529
	NDUFB6	NADH:ubiquinone oxidoreductase subunit B6	0.611903	0.800763
	NDUFB9	NADH:ubiquinone oxidoreductase subunit B9	0.555857	0.618531
	NDUFS4	NADH:ubiquinone oxidoreductase subunit S4	0.599484	0.649652
	NDUFS5	NADH:ubiquinone oxidoreductase subunit S5	0.447218	0.58327
	NEK6	NIMA related kinase 6	0.713992	0.544106
	NENF	neudesin neurotrophic factor	0.63689	0.689993
	NFIA	nuclear factor I A	1.93518	1.60973
	NGRN	"neugrin, neurite outgrowth associated"	0.449335	0.411608
	NHP2	NHP2 ribonucleoprotein	0.656477	0.842416
	NIFK	nucleolar protein interacting with the FHA domain of MKI67	0.614676	0.649105
	NIPSNAP3A	nipsnap homolog 3A	1.98433	2.36503
	NIPSNAP3B	nipsnap homolog 3B	0.977539	0.816229
	NKIRAS2	NFKB inhibitor interacting Ras like 2	1.79244	1.98048
	NKRF	NFKB repressing factor	0.562578	0.591047
	NLE1	notchless homolog 1	0.739256	0.961983
	NLRP1	NLR family pyrin domain containing 1	1.09248	1.19686
	NLRX1	NLR family member X1	0.861659	0.784992
	NMB	neuromedin B	1.57817	1.63764
	NME1	NME/NM23 nucleoside diphosphate kinase 1	0.856472	0.982628
	NME1-NME2	NME1-NME2 readthrough	0.537902	0.568051
	NMU	neuromedin U	1.0744	0.667768
	NOB1	NIN1/PSMD8 binding protein 1 homolog	0.454083	0.560442
	NOC3L	NOC3 like DNA replication regulator	0.463655	0.418221
	NOL11	nucleolar protein 11	0.584325	0.564753
	NOP10	NOP10 ribonucleoprotein	1.02759	1.12941
	NOP56	NOP56 ribonucleoprotein	0.588583	0.625073
	NOP58	NOP58 ribonucleoprotein	0.57573	0.474755
	NPM3	nucleophosmin/nucleoplasmin 3	1.59262	1.6237
	NPY1R	neuropeptide Y receptor Y1	1.33529	1.13624
	NQO2	NAD(P)H quinone dehydrogenase 2	0.893087	0.924738
	NR5A2	nuclear receptor subfamily 5 group A member 2	1.21764	0.825542
	NRBP1	nuclear receptor binding protein 1	0.590665	0.596604
	NREP	neuronal regeneration related protein	1.5593	1.33906
	NRTN	neurturin	1.42941	1.46177
	NSL1	"NSL1, MIS12 kinetochore complex component"	0.575272	0.679574
	NT5C3B	"5'-nucleotidase, cytosolic IIIB"	0.390653	0.394116
	NTMT1	N-terminal Xaa-Pro-Lys N-methyltransferase 1	0.366753	0.68196
	NUDCD1	NudC domain containing 1	0.605891	0.538786
	NUDT1	nudix hydrolase 1	0.488716	0.574153
	NUDT16L1	nudix hydrolase 16 like 1	0.742033	1.02527
	NUDT2	nudix hydrolase 2	0.587172	0.742747
	NUP160	nucleoporin 160	1.02944	0.977176
	NUP35	nucleoporin 35	0.649279	0.599748
	NUS1	NUS1 dehydrodolichyl diphosphate synthase subunit	0.588642	0.461185
	NXN	nucleoredoxin	0.717945	0.799884
	NXPH3	neurexophilin 3	1.14279	1.13923
	NXT1	nuclear transport factor 2 like export factor 1	0.594323	0.796174
	OAF	out at first homolog	0.614108	1.2268
	OASL	2'-5'-oligoadenylate synthetase like	0.68925	1.43106
	OAT	ornithine aminotransferase	0.61619	0.435381
	OBSL1	obscurin like 1	0.990759	1.04061

	OIP5	Opa interacting protein 5	2.11953	2.39693
	OIP5-AS1	OIP5 antisense RNA 1	1.90865	1.75325
	OLFML3	olfactomedin like 3	2.60869	2.68797
	ORC5	origin recognition complex subunit 5	0.451018	0.481556
	ORC6	origin recognition complex subunit 6	0.500223	0.527708
	OSGEP	O-sialoglycoprotein endopeptidase	0.590015	0.706425
	OSGEPL1	O-sialoglycoprotein endopeptidase like 1	0.946834	0.805438
	OST4	"oligosaccharyltransferase complex subunit 4, non-catalytic"	0.446121	0.460205
	OSTC	oligosaccharyltransferase complex non-catalytic subunit	2.19415	2.35898
	P2RY6	pyrimidinergic receptor P2Y6	1.07541	1.20488
	P3H1	prolyl 3-hydroxylase 1	1.17836	0.882606
	P3H4	prolyl 3-hydroxylase family member 4 (non-enzymatic)	1.43784	1.60295
	PACRGL	PARK2 coregulated like	0.71929	0.778749
	PADI3	peptidyl arginine deiminase 3	1.29671	1.12462
	PAICS	phosphoribosylaminoimidazole carboxylase; phosphoribosylaminoimidazolesuccinocarboxamide synthase	0.627836	0.480478
	PAK1IP1	PAK1 interacting protein 1	0.562759	0.666736
	PANK1	pantothenate kinase 1	0.797274	0.730937
	PARG	poly(ADP-ribose) glycohydrolase	0.88996	0.913123
	PARK7	Parkinsonism associated deglycase	0.541766	0.515431
	PARL	presenilin associated rhomboid like	0.535057	0.586764
	PARP12	poly(ADP-ribose) polymerase family member 12	0.41123	0.735736
	PAX9	paired box 9	0.58445	0.662648
	PBDC1	polysaccharide biosynthesis domain containing 1	0.702885	0.752183
	PCCB	propionyl-CoA carboxylase beta subunit	0.879864	0.830985
	PCDHB5	protocadherin beta 5	1.21352	1.02547
	PCDHB6	protocadherin beta 6	0.547292	0.578213
	PCDHB8	protocadherin beta 8	1.12031	0.851423
	PCGF6	polycomb group ring finger 6	0.935854	0.998118
	PCIF1	PDX1 C-terminal inhibiting factor 1	0.398303	0.598229
	PCNA	proliferating cell nuclear antigen	0.420561	0.587194
	PCOLCE2	procollagen C-endopeptidase enhancer 2	0.623997	0.976315
	PCSK9	proprotein convertase subtilisin/kexin type 9	0.536665	0.556471
	PCYOX1L	prenylcysteine oxidase 1 like	1.0197	1.04292
	PDCD2	programmed cell death 2	0.709717	0.75917
	PDE1A	phosphodiesterase 1A	1.68231	1.53396
	PDE4B	phosphodiesterase 4B	1.30251	1.10839
	PDHB	pyruvate dehydrogenase (lipoamide) beta	1.02254	0.966478
	PDHX	pyruvate dehydrogenase complex component X	1.11496	1.1193
	PDIA2	protein disulfide isomerase family A member 2	0.84232	1.0513
	PDIA4	protein disulfide isomerase family A member 4	0.832177	0.763579
	PDIA6	protein disulfide isomerase family A member 6	0.540674	0.422012
	PDLIM3	PDZ and LIM domain 3	1.21686	1.19913
	PDRG1	p53 and DNA damage regulated 1	0.59653	0.79471
	PDZK1	PDZ domain containing 1	2.32078	1.75265
	PEBP1	phosphatidylethanolamine binding protein 1	0.5634	0.499523
	PEG10	paternally expressed 10	2.10984	2.21207
	PEX2	peroxisomal biogenesis factor 2	0.769444	0.809132
	PEX5	peroxisomal biogenesis factor 5	0.622786	0.595623
	PFDN5	prefoldin subunit 5	0.546246	0.491788
	PGAM1	phosphoglycerate mutase 1	0.452985	0.512096
	PGBD5	piggyBac transposable element derived 5	0.874885	1.03782
	PGD	phosphogluconate dehydrogenase	0.551699	0.534879
	PGM1	phosphoglucomutase 1	0.901442	0.766433
	PHB	prohibitin	0.558869	0.571996
	PHB2	prohibitin 2	0.658567	0.742473
	PHF10	PHD finger protein 10	1.47714	1.42481
	PHF13	PHD finger protein 13	0.548822	0.679934
	PHLDA1	pleckstrin homology like domain family A member 1	0.52809	0.652852
	PHTF2	putative homeodomain transcription factor 2	0.995553	0.932872
	PI15	peptidase inhibitor 15	1.38949	1.21943
	PIGC	phosphatidylinositol glycan anchor biosynthesis class C	0.41005	0.397585

	PIGM	phosphatidylinositol glycan anchor biosynthesis class M	0.537811	0.601687
	PIGN	phosphatidylinositol glycan anchor biosynthesis class N	1.27463	0.843688
	PIGS	phosphatidylinositol glycan anchor biosynthesis class S	1.71025	1.52542
	PIK3C2G	phosphatidylinositol-4-phosphate 3-kinase catalytic subunit type 2 gamma	1.32998	1.10551
	PINX1	"PIN2/TERF1 interacting, telomerase inhibitor 1"	0.563699	0.614464
	PIP	prolactin induced protein	1.58833	1.40631
	PIR	pirin	1.18405	1.09699
	PITPNC1	"phosphatidylinositol transfer protein, cytoplasmic 1"	0.752945	0.698521
	PKIB	"protein kinase (cAMP-dependent, catalytic) inhibitor beta"	0.923158	0.820975
	PLEK2	pleckstrin 2	0.612746	0.482887
	PLEKHO1	pleckstrin homology domain containing O1	0.955779	0.986967
	PLGRKT	plasminogen receptor with a C-terminal lysine	1.03486	0.956894
	PLK1	polo like kinase 1	0.502805	0.534145
	PLK2	polo like kinase 2	2.01513	1.87612
	PLP1	proteolipid protein 1	1.78971	2.78286
	PLSCR1	phospholipid scramblase 1	0.400567	0.622252
	PMF1	polyamine modulated factor 1	0.49455	0.668554
	PMPCA	"peptidase, mitochondrial processing alpha subunit"	0.741118	0.917951
	PNMA1	paraneoplastic Ma antigen 1	0.569994	0.68552
	PNP	purine nucleoside phosphorylase	0.562185	0.634676
	PNPT1	polyribonucleotide nucleotidyltransferase 1	0.771053	0.820103
	POLB	DNA polymerase beta	0.679722	0.900578
	POLD2	"DNA polymerase delta 2, accessory subunit"	0.947729	0.850791
	POLDIP2	DNA polymerase delta interacting protein 2	0.545445	0.547899
	POLE3	"DNA polymerase epsilon 3, accessory subunit"	1.31072	1.50374
	POLE4	"DNA polymerase epsilon 4, accessory subunit"	0.373808	0.576856
	POLG2	"DNA polymerase gamma 2, accessory subunit"	0.496162	0.584555
	POLR1D	RNA polymerase I subunit D	1.21672	1.4292
	POLR1E	RNA polymerase I subunit E	0.416051	0.360962
	POLR2G	RNA polymerase II subunit G	1.0195	1.11076
	POLR2K	RNA polymerase II subunit K	0.36188	0.359019
	POLR3D	RNA polymerase III subunit D	0.922376	1.05985
	POLR3G	RNA polymerase III subunit G	0.810646	1.02777
	POLR3K	RNA polymerase III subunit K	0.601195	0.724844
	POMP	proteasome maturation protein	0.398388	0.424103
	POP5	"POP5 homolog, ribonuclease P/MRP subunit"	0.785138	1.02197
	POPDC3	popeye domain containing 3	0.787233	1.0069
	POU1F1	POU class 1 homeobox 1	1.10366	1.22851
	POU3F2	POU class 3 homeobox 2	1.23518	1.18252
	PPA1	pyrophosphatase (inorganic) 1	0.889133	0.91749
	PPA2	pyrophosphatase (inorganic) 2	0.417186	0.475001
	PPIA	peptidylprolyl isomerase A	0.404212	0.429638
	PPIB	peptidylprolyl isomerase B	1.11594	1.2242
	PPIH	peptidylprolyl isomerase H	0.40786	0.56116
	PPIL1	peptidylprolyl isomerase like 1	0.72316	0.771098
	PPM1D	"protein phosphatase, Mg2+/Mn2+ dependent 1D"	0.805558	0.828481
	PPM1G	"protein phosphatase, Mg2+/Mn2+ dependent 1G"	0.421925	0.389986
	PPP1R14C	protein phosphatase 1 regulatory inhibitor subunit 14C	2.03073	2.02062
	PPP1R3B	protein phosphatase 1 regulatory subunit 3B	0.433307	0.398739
	PPP4C	protein phosphatase 4 catalytic subunit	0.389742	0.577441
	PRADC1	protease associated domain containing 1	0.539953	0.658061
	PRDX3	peroxiredoxin 3	0.627377	0.542784
	PRDX4	peroxiredoxin 4	0.874995	0.937589
	PRDX6	peroxiredoxin 6	0.644178	0.561437
	PRELID1	PRELI domain containing 1	0.526265	0.474331
	PRIMA1	proline rich membrane anchor 1	0.382483	0.378951
	PRKACA	protein kinase cAMP-activated catalytic subunit alpha	0.628271	0.54078
	PRMT5	protein arginine methyltransferase 5	0.737209	0.665835

	PRPF38A	pre-mRNA processing factor 38A	1.47559	1.5399
	PRR3	proline rich 3	1.05653	1.07873
	PRSS23	"protease, serine 23"	1.31424	1.13168
	PRTG	protogenin	1.31376	1.23996
	PRX	periaxin	1.08466	0.880227
	PSKH1	protein serine kinase H1	0.564947	0.756137
	PSMA2	proteasome subunit alpha 2	0.711013	0.747366
	PSMA4	proteasome subunit alpha 4	1.20228	1.18312
	PSMA7	proteasome subunit alpha 7	0.611977	0.569242
	PSMB1	proteasome subunit beta 1	0.365698	0.454699
	PSMB4	proteasome subunit beta 4	0.447662	0.505157
	PSMB5	proteasome subunit beta 5	0.647283	0.685028
	PSMB7	proteasome subunit beta 7	0.703839	0.733512
	PSMC1	"proteasome 26S subunit, ATPase 1"	0.568718	0.565683
	PSMC2	"proteasome 26S subunit, ATPase 2"	0.556815	0.614312
	PSMC3	"proteasome 26S subunit, ATPase 3"	0.867627	0.83266
	PSMD13	"proteasome 26S subunit, non-ATPase 13"	0.720864	0.803083
	PSMD7	"proteasome 26S subunit, non-ATPase 7"	0.485609	0.55362
	PSME1	proteasome activator subunit 1	0.489988	0.532391
	PSMG1	proteasome assembly chaperone 1	0.681266	0.765086
	PSPH	phosphoserine phosphatase	0.415752	0.442755
	PSTPIP2	proline-serine-threonine phosphatase interacting protein 2	0.853152	0.921707
	PTDSS1	phosphatidylserine synthase 1	0.677304	0.585521
	PTENP1	phosphatase and tensin homolog pseudogene 1	1.06433	1.26423
	PTGES2	prostaglandin E synthase 2	0.415851	0.570339
	PTMA	"prothymosin, alpha"	0.472511	0.39151
	PVALB	parvalbumin	1.21037	1.57428
	PYCR1	pyrroline-5-carboxylate reductase 1	0.667415	0.824556
	PYGL	"phosphorylase, glycogen, liver"	0.598803	0.527726
	QTRT1	queuine tRNA-ribosyltransferase catalytic subunit 1	0.471824	0.661715
	R3HCC1	R3H domain and coiled-coil containing 1	1.47594	1.51118
	RAB20	"RAB20, member RAS oncogene family"	0.54106	0.671545
	RAB4A	"RAB4A, member RAS oncogene family"	0.553993	0.451898
	RAB9B	"RAB9B, member RAS oncogene family"	1.39674	1.60651
	RABEPK	Rab9 effector protein with kelch motifs	0.550754	0.599569
	RACK1	receptor for activated C kinase 1	0.479845	0.474264
	RAD51C	RAD51 paralog C	0.445142	0.420747
	RAN	"RAN, member RAS oncogene family"	0.649795	0.716611
	RANP1	"RAN, member RAS oncogene family pseudogene 1"	0.818454	1.5527
	RAPGEFL1	Rap guanine nucleotide exchange factor like 1	1.07791	0.906922
	RARG	retinoic acid receptor gamma	0.99176	0.914053
	RASA3	RAS p21 protein activator 3	0.752342	0.715053
	RASGEF1B	RasGEF domain family member 1B	0.863543	1.06333
	RASL11A	RAS like family 11 member A	1.18544	1.55333
	RBFA	ribosome binding factor A (putative)	1.08745	1.15657
	RBMS2	RNA binding motif single stranded interacting protein 2	0.988779	0.695869
	RCL1	RNA terminal phosphate cyclase like 1	0.58902	0.770248
	RDH16	retinol dehydrogenase 16 (all-trans)	0.549674	0.848992
	RERG	RAS like estrogen regulated growth inhibitor	0.739758	0.650693
	REXO4	"REX4 homolog, 3'-5' exonuclease"	0.991587	1.21563
	RFC2	replication factor C subunit 2	0.554049	0.470145
	RFC3	replication factor C subunit 3	0.585816	0.562186
	RFLNB	refilin B	1.58933	1.30077
	RFT1	RFT1 homolog	1.00573	0.963011
	RFWD2	ring finger and WD repeat domain 2	0.768969	0.637484
	RFXAP	regulatory factor X associated protein	1.06703	0.94652
	RGS16	regulator of G-protein signaling 16	0.542244	0.617958
	RIDA	reactive intermediate imine deaminase A homolog	0.692374	0.508209
	RIMS4	regulating synaptic membrane exocytosis 4	1.25577	1.2212
	RIT1	Ras like without CAAX 1	0.743862	0.771635
	RLN2	relaxin 2	0.992626	0.927344
	RNASEL	ribonuclease L	1.11074	1.05212
	RNASET2	ribonuclease T2	0.439969	0.479086

	RND2	Rho family GTPase 2	0.528641	0.797044
	RNF122	ring finger protein 122	1.14128	0.979431
	RNF130	ring finger protein 130	0.700167	0.585128
	RNF138	ring finger protein 138	0.993903	1.2314
	RNF14	ring finger protein 14	0.744299	0.790873
	RNF144B	ring finger protein 144B	0.536437	0.670161
	RNF165	ring finger protein 165	1.06013	0.861514
	RNF5	ring finger protein 5	0.74448	0.82896
	RNF7	ring finger protein 7	0.475151	0.652396
	RPA1	replication protein A1	1.3934	1.36769
	RPIA	ribose 5-phosphate isomerase A	0.794538	0.845553
	RPL10A	ribosomal protein L10a	0.714578	0.750405
	RPL11	ribosomal protein L11	0.642594	0.604273
	RPL12	ribosomal protein L12	0.609403	0.591557
	RPL13	ribosomal protein L13	0.48154	0.660912
	RPL13A	ribosomal protein L13a	0.565281	0.690617
	RPL15	ribosomal protein L15	0.477561	0.543018
	RPL17	ribosomal protein L17	0.608482	0.579599
	RPL19	ribosomal protein L19	0.501585	0.555254
	RPL21	ribosomal protein L21	0.755891	0.73765
	RPL22L1	ribosomal protein L22 like 1	0.864808	0.965201
	RPL23	ribosomal protein L23	0.713014	0.658251
	RPL24	ribosomal protein L24	0.775667	0.80338
	RPL26	ribosomal protein L26	0.635677	0.560063
	RPL27	ribosomal protein L27	0.818785	0.914966
	RPL27A	ribosomal protein L27a	0.602351	0.694477
	RPL29	ribosomal protein L29	0.678791	0.965449
	RPL3	ribosomal protein L3	0.643843	0.633513
	RPL30	ribosomal protein L30	0.483029	0.514125
	RPL31	ribosomal protein L31	0.500837	0.553839
	RPL32	ribosomal protein L32	0.541315	0.574202
	RPL34	ribosomal protein L34	0.791899	0.858151
	RPL35	ribosomal protein L35	0.587071	0.681671
	RPL35A	ribosomal protein L35a	0.687875	0.754828
	RPL36A	ribosomal protein L36a	0.665105	0.625905
	RPL36AL	ribosomal protein L36a like	0.544335	0.657496
	RPL37A	ribosomal protein L37a	0.534367	0.585879
	RPL39	ribosomal protein L39	0.790025	0.793589
	RPL39L	ribosomal protein L39 like	0.71943	0.884334
	RPL4	ribosomal protein L4	0.727798	0.651767
	RPL41	ribosomal protein L41	0.863473	0.842525
	RPL5	ribosomal protein L5	0.876493	0.834361
	RPL7	ribosomal protein L7	0.540895	0.474478
	RPL7A	ribosomal protein L7a	0.525127	0.483312
	RPL8	ribosomal protein L8	0.447702	0.482796
	RPL9	ribosomal protein L9	0.790399	0.797841
	RPLP0	ribosomal protein lateral stalk subunit P0	0.690141	0.646959
	RPN1	ribophorin I	0.436504	0.390911
	RPP25L	ribonuclease P/MRP subunit p25 like	0.589825	0.823949
	RPP40	ribonuclease P/MRP subunit p40	0.442086	0.477138
	RPRM	"reprimo, TP53 dependent G2 arrest mediator candidate"	0.725484	0.672673
	RPS10	ribosomal protein S10	0.69203	0.772701
	RPS11	ribosomal protein S11	0.516476	0.496856
	RPS12	ribosomal protein S12	0.585658	0.565778
	RPS13	ribosomal protein S13	0.506	0.560536
	RPS14	ribosomal protein S14	0.610268	0.66288
	RPS15A	ribosomal protein S15a	0.97588	0.954983
	RPS16	ribosomal protein S16	0.595941	0.646833
	RPS18	ribosomal protein S18	0.638633	0.730978
	RPS20	ribosomal protein S20	0.495754	0.533154
	RPS21	ribosomal protein S21	0.612498	0.878411
	RPS23	ribosomal protein S23	0.644234	0.677774
	RPS25	ribosomal protein S25	0.679746	0.654124
	RPS26	ribosomal protein S26	0.600671	0.710175
	RPS27	ribosomal protein S27	0.508871	0.454789

	RPS27A	ribosomal protein S27a	0.982195	0.969381
	RPS29	ribosomal protein S29	1.07896	1.23262
	RPS3	ribosomal protein S3	0.756681	0.745014
	RPS4X	"ribosomal protein S4, X-linked"	0.617263	0.645454
	RPS5	ribosomal protein S5	0.563525	0.738254
	RPS6	ribosomal protein S6	0.80126	0.751089
	RPS6KA5	ribosomal protein S6 kinase A5	0.591567	0.484197
	RPS6KB1	ribosomal protein S6 kinase B1	0.690924	0.661297
	RPS7	ribosomal protein S7	0.976177	0.982406
	RPS8	ribosomal protein S8	0.798614	0.76595
	RPS9	ribosomal protein S9	0.494933	0.615055
	RPSA	ribosomal protein SA	0.728888	0.69114
	RRAGA	Ras related GTP binding A	0.713067	0.811297
	RRAS2	related RAS viral (r-ras) oncogene homolog 2	0.839157	0.770152
	RRP8	"ribosomal RNA processing 8, methyltransferase, homolog (yeast)"	0.501806	0.66187
	RSL1D1	ribosomal L1 domain containing 1	0.599977	0.50853
	RSPO1	R-spondin 1	1.00213	1.14493
	RTN1	reticulon 1	1.33667	1.24382
	RUNX2	runt related transcription factor 2	1.40906	1.12666
	RUVBL2	RuvB like AAA ATPase 2	0.370826	0.545808
	RWDD1	RWD domain containing 1	0.790947	0.703144
	RWDD2B	RWD domain containing 2B	0.760684	0.77008
	S100A14	S100 calcium binding protein A14	0.526757	0.460932
	S100A4	S100 calcium binding protein A4	0.677395	0.465981
	S100A8	S100 calcium binding protein A8	1.92461	3.08523
	S100A9	S100 calcium binding protein A9	1.53666	2.52338
	S1PR5	sphingosine-1-phosphate receptor 5	0.544561	0.637201
	SAMHD1	SAM and HD domain containing deoxynucleoside triphosphate triphosphohydrolase 1	0.422929	0.441313
	SARAF	store-operated calcium entry associated regulatory factor	0.651737	0.581324
	SAT1	spermidine/spermine N1-acetyltransferase 1	0.701296	0.864274
	SCAP	SREBF chaperone	0.857089	0.770539
	SCML1	sex comb on midleg-like 1 (Drosophila)	0.763639	0.740902
	SDC2	syndecan 2	0.629612	0.601104
	SDF2L1	stromal cell derived factor 2 like 1	0.554218	0.876166
	SDHAF3	succinate dehydrogenase complex assembly factor 3	1.03412	1.20257
	SDHB	succinate dehydrogenase complex iron sulfur subunit B	0.436855	0.422219
	SEC11A	"SEC11 homolog A, signal peptidase complex subunit"	0.489579	0.537134
	SEC11C	"SEC11 homolog C, signal peptidase complex subunit"	0.493302	0.583424
	SEC16B	"SEC16 homolog B, endoplasmic reticulum export factor"	1.14986	0.932813
	SEC61B	Sec61 translocon beta subunit	0.380985	0.532805
	SELK	selenoprotein K	0.506295	0.561729
	SEMA3G	semaphorin 3G	1.08938	1.18135
	SEP15	15 kDa selenoprotein	0.612944	0.630963
	SEPHS2	selenophosphate synthetase 2	0.470174	0.583339
	SEPP1	"selenoprotein P, plasma, 1"	1.09129	1.21632
	SERBP1	SERPINE1 mRNA binding protein 1	0.99881	0.945595
	SERPINB6	serpin family B member 6	0.400069	0.406089
	SERPINH1	serpin family H member 1	1.97041	2.06858
	SET	SET nuclear proto-oncogene	0.742598	0.618882
	SF3B5	splicing factor 3b subunit 5	0.996388	1.13546
	SF3B6	splicing factor 3b subunit 6	0.369752	0.345683
	SFR1	SWI5 dependent homologous recombination repair protein 1	0.733793	0.792653
	SFXN2	sideroflexin 2	0.977583	0.949226
	SFXN4	sideroflexin 4	0.706769	0.733447
	SGCG	sarcoglycan gamma	1.58645	1.76154
	SH3BP5	SH3 domain binding protein 5	0.463129	0.394409
	SH3BP5L	SH3 binding domain protein 5 like	1.04061	1.05831

	SH3GL1	"SH3 domain containing GRB2 like 1, endophilin A2"	1.20402	1.35972
	SH3GL3	"SH3 domain containing GRB2 like 3, endophilin A3"	1.68462	1.73615
	SHROOM2	shroom family member 2	0.930158	0.799281
	SIAH2	siah E3 ubiquitin protein ligase 2	1.85018	1.90909
	SKP2	"S-phase kinase-associated protein 2, E3 ubiquitin protein ligase"	0.987079	0.961049
	SLC16A1	solute carrier family 16 member 1	1.27052	1.23591
	SLC16A10	solute carrier family 16 member 10	1.92999	1.71366
	SLC16A14	solute carrier family 16 member 14	0.890857	1.04069
	SLC18B1	solute carrier family 18 member B1	1.68871	1.89729
	SLC19A1	solute carrier family 19 member 1	0.711999	0.848981
	SLC19A2	solute carrier family 19 member 2	0.994844	1.03721
	SLC22A31	solute carrier family 22 member 31	0.856728	0.948328
	SLC25A13	solute carrier family 25 member 13	1.29903	1.43565
	SLC25A15	solute carrier family 25 member 15	1.27744	1.19857
	SLC25A17	solute carrier family 25 member 17	0.567036	0.550626
	SLC25A20	solute carrier family 25 member 20	0.55318	0.705508
	SLC25A33	solute carrier family 25 member 33	1.28595	1.32461
	SLC25A4	solute carrier family 25 member 4	0.688413	0.804581
	SLC25A5	solute carrier family 25 member 5	0.874204	0.931417
	SLC27A5	solute carrier family 27 member 5	1.0881	1.3092
	SLC29A2	solute carrier family 29 member 2	0.357566	0.444176
	SLC35B2	solute carrier family 35 member B2	1.49914	1.5692
	SLC35C2	solute carrier family 35 member C2	0.614949	0.696564
	SLC35G1	solute carrier family 35 member G1	0.497059	0.57222
	SLC45A3	solute carrier family 45 member 3	0.928566	1.04996
	SLC4A7	solute carrier family 4 member 7	0.972962	0.90704
	SLC50A1	solute carrier family 50 member 1	0.490646	0.467501
	SLC5A8	solute carrier family 5 member 8	2.78152	2.31415
	SLC6A12	solute carrier family 6 member 12	1.42094	1.24514
	SLC7A5	solute carrier family 7 member 5	1.31711	1.12174
	SLC7A8	solute carrier family 7 member 8	0.772529	0.646335
	SLIRP	SRA stem-loop interacting RNA binding protein	0.769817	0.866559
	SMAD6	SMAD family member 6	0.666859	0.829978
	SMIM11A	small integral membrane protein 11A	0.844039	0.910736
	SMIM13	small integral membrane protein 13	0.483039	0.520542
	SMIM8	small integral membrane protein 8	0.664991	0.817361
	SMN1	"survival of motor neuron 1, telomeric"	0.581755	0.575289
	SMPD2	sphingomyelin phosphodiesterase 2	0.898892	0.733124
	SMS	spermine synthase	1.1773	1.20089
	SNHG5	small nucleolar RNA host gene 5	0.587049	0.815636
	SNRNP25	small nuclear ribonucleoprotein U11/U12 subunit 25	0.656031	0.603566
	SNRPA1	small nuclear ribonucleoprotein polypeptide A'	0.885467	0.861662
	SNRPD2	small nuclear ribonucleoprotein D2 polypeptide	0.748278	0.885684
	SNRPD3	small nuclear ribonucleoprotein D3 polypeptide	0.859864	0.921748
	SNRPE	small nuclear ribonucleoprotein polypeptide E	0.579614	0.655083
	SNRPF	small nuclear ribonucleoprotein polypeptide F	0.378183	0.562532
	SNRPG	small nuclear ribonucleoprotein polypeptide G	0.722707	0.941404
	SNTA1	syntrophin alpha 1	0.470681	0.524896
	SNX17	sorting nexin 17	0.352968	0.410153
	SNX4	sorting nexin 4	0.537189	0.550717
	SOAT2	sterol O-acyltransferase 2	0.946383	0.996519
	SOBP	sine oculis binding protein homolog	1.12312	0.97963
	SOWAHC	sosondowah ankyrin repeat domain family member C	0.757397	0.721909
	SP6	Sp6 transcription factor	0.627875	0.798127
	SPAG16	sperm associated antigen 16	2.78745	1.96532
	SPARC	secreted protein acidic and cysteine rich	2.76873	3.35492
	SPDL1	spindle apparatus coiled-coil protein 1	1.36245	1.18421
	SPIN4	spindlin family member 4	1.20897	1.15524
	SPNS1	sphingolipid transporter 1 (putative)	0.993692	1.03893
	SPRY2	sprouty RTK signaling antagonist 2	0.550961	0.481338
	SPRY4	sprouty RTK signaling antagonist 4	2.72265	3.40137
	SPRYD4	SPRY domain containing 4	0.498969	0.658557

	SPSB4	splA/ryanodine receptor domain and SOCS box containing 4	1.17641	0.758689
	SPTSSA	serine palmitoyltransferase small subunit A	1.13176	1.10577
	SQLE	squalene epoxidase	0.859419	0.65273
	SRD5A1	steroid 5 alpha-reductase 1	0.903325	1.10497
	SRD5A3	steroid 5 alpha-reductase 3	0.53407	0.56115
	SRP9	signal recognition particle 9	0.611286	0.511968
	SRPK2	SRSF protein kinase 2	0.962142	0.955164
	SRPRB	SRP receptor beta subunit	1.135	1.13818
	SRSF10	serine and arginine rich splicing factor 10	0.581423	0.557139
	SRSF3	serine and arginine rich splicing factor 3	0.459896	0.479402
	SRSF7	serine and arginine rich splicing factor 7	0.558072	0.589317
	SS18L1	"SS18L1, nBAF chromatin remodeling complex subunit"	0.699734	0.792439
	SSB	Sjogren syndrome antigen B	0.68798	0.631619
	SSR2	signal sequence receptor subunit 2	0.883125	0.937574
	SSR3	signal sequence receptor subunit 3	0.712814	0.67759
	STAG3L4	stromal antigen 3-like 4 (pseudogene)	0.59115	0.623864
	STARD5	StAR related lipid transfer domain containing 5	0.757691	0.804855
	STK19	serine/threonine kinase 19	1.04042	1.10347
	STOML2	stomatin like 2	0.620558	0.633006
	STRAP	serine/threonine kinase receptor associated protein	0.923435	0.944209
	STX10	syntaxin 10	0.682659	0.746872
	SUB1	"SUB1 homolog, transcriptional regulator"	0.789299	0.78565
	SUCLA2	succinate-CoA ligase ADP-forming beta subunit	1.17971	0.989095
	SUCLG1	succinate-CoA ligase alpha subunit	0.393309	0.459611
	SUFU	SUFU negative regulator of hedgehog signaling	0.632929	0.650479
	SUMF2	sulfatase modifying factor 2	0.425012	0.415504
	SUMO1	small ubiquitin-like modifier 1	0.650273	0.599114
	SUSD3	sushi domain containing 3	1.38012	1.41753
	SUV39H2	suppressor of variegation 3-9 homolog 2	0.68922	0.680572
	SYNE4	spectrin repeat containing nuclear envelope family member 4	0.83352	0.797764
	SYT8	synaptotagmin 8	1.65318	1.36842
	TAF11	TATA-box binding protein associated factor 11	2.53393	2.61299
	TAF4B	TATA-box binding protein associated factor 4b	0.927441	0.721482
	TAF7	TATA-box binding protein associated factor 7	0.495937	0.658128
	TBCCD1	TBCC domain containing 1	1.68387	1.5637
	TBP	TATA-box binding protein	0.439215	0.44549
	TCEAL4	transcription elongation factor A like 4	0.865075	1.04156
	TCEB1	transcription elongation factor B subunit 1	0.384157	0.433829
	TCF7L1	transcription factor 7 like 1	1.48257	1.55383
	TCFL5	transcription factor like 5	0.531116	0.509004
	TDP2	tyrosyl-DNA phosphodiesterase 2	0.488266	0.43491
	TEAD2	TEA domain transcription factor 2	0.477682	0.408097
	TEFM	"transcription elongation factor, mitochondrial"	0.886352	1.03729
	TEKT4P2	tektin 4 pseudogene 2	1.25626	1.31105
	TERT	telomerase reverse transcriptase	1.89687	1.94267
	TET2	tet methylcytosine dioxygenase 2	0.974007	0.8011
	TEX30	testis expressed 30	0.591534	0.539069
	TEX9	testis expressed 9	0.870901	1.00428
	TFAP2C	transcription factor AP-2 gamma	0.827	0.84
	TFDP1	transcription factor Dp-1	0.598627	0.557178
	TFEC	transcription factor EC	2.11704	2.40168
	TFF1	trefoil factor 1	1.12383	1.05027
	TFF3	trefoil factor 3	1.06078	1.23842
	TFPI	tissue factor pathway inhibitor	0.685574	0.818259
	TGDS	"TDP-glucose 4,6-dehydratase"	0.708024	0.695662
	THEG	theg spermatid protein	1.36115	1.28725
	THOC3	THO complex 3	0.747607	0.706681
	THOC7	THO complex 7	0.662423	0.585282
	TIAM1	T-cell lymphoma invasion and metastasis 1	1.78994	1.45061
	TIMM10	translocase of inner mitochondrial membrane 10 homolog (yeast)	1.08019	1.23627
	TIMM17A	translocase of inner mitochondrial membrane 17 homolog A (yeast)	0.741463	0.806169
	TIMM21	translocase of inner mitochondrial membrane 21	0.679132	0.844579

	TIMM44	translocase of inner mitochondrial membrane 44	0.990688	1.19053
	TIMM8A	translocase of inner mitochondrial membrane 8 homolog A (yeast)	0.716225	0.843205
	TIMM8B	translocase of inner mitochondrial membrane 8 homolog B	2.80661	3.09556
	TIMMDC1	translocase of inner mitochondrial membrane domain containing 1	0.470364	0.511371
	TIPRL	TOR signaling pathway regulator	0.811212	0.873609
	TKT	transketolase	0.929334	1.00705
	TM4SF1	transmembrane 4 L six family member 1	0.892293	1.16473
	TMA7	translation machinery associated 7 homolog	0.568972	0.735707
	TMED3	transmembrane p24 trafficking protein 3	0.862257	0.952594
	TMEM106C	transmembrane protein 106C	0.585347	0.488068
	TMEM11	transmembrane protein 11	0.395127	0.594322
	TMEM126A	transmembrane protein 126A	0.457839	0.533496
	TMEM126B	transmembrane protein 126B	0.727283	0.641183
	TMEM134	transmembrane protein 134	0.907853	1.34654
	TMEM141	transmembrane protein 141	1.08181	1.17586
	TMEM147	transmembrane protein 147	0.659977	0.73448
	TMEM14B	transmembrane protein 14B	0.746991	0.758224
	TMEM14C	transmembrane protein 14C	0.620905	0.590898
	TMEM171	transmembrane protein 171	0.878415	0.876701
	TMEM177	transmembrane protein 177	0.455281	0.744802
	TMEM179B	transmembrane protein 179B	1.13071	1.1919
	TMEM181	transmembrane protein 181	0.528742	0.421469
	TMEM183A	transmembrane protein 183A	1.61754	1.69108
	TMEM189	transmembrane protein 189	1.4711	1.43429
	TMEM203	transmembrane protein 203	0.536841	0.70267
	TMEM208	transmembrane protein 208	0.434963	0.511256
	TMEM216	transmembrane protein 216	0.578493	0.670693
	TMEM218	transmembrane protein 218	0.819994	0.843916
	TMEM223	transmembrane protein 223	1.13466	1.40979
	TMEM229B	transmembrane protein 229B	0.964364	0.908993
	TMEM237	transmembrane protein 237	0.646639	0.612708
	TMEM251	transmembrane protein 251	0.873496	1.02926
	TMEM254	transmembrane protein 254	0.725767	0.733062
	TMEM261	transmembrane protein 261	0.671823	0.814308
	TMEM38A	transmembrane protein 38A	0.820518	0.816127
	TMEM5	transmembrane protein 5	0.426584	0.498614
	TMEM65	transmembrane protein 65	1.01325	0.856892
	TMEM97	transmembrane protein 97	0.833185	0.612911
	TMPOP2	thymopoietin pseudogene 2	0.815511	1.2229
	TMPRSS3	"transmembrane protease, serine 3"	1.50993	1.27099
	TMSB15A	thymosin beta 15a	2.53916	2.21981
	TMSB15B	thymosin beta 15B	0.668357	0.666676
	TNFAIP8L2-SCNM1	TNFAIP8L2-SCNM1 readthrough	1.15396	1.28294
	TNFRSF11B	TNF receptor superfamily member 11b	2.27648	2.51838
	TNFSF15	tumor necrosis factor superfamily member 15	0.836726	1.01551
	TOMM22	translocase of outer mitochondrial membrane 22	0.494304	0.551779
	TOMM40L	translocase of outer mitochondrial membrane 40 like	0.675673	0.883447
	TOMM5	translocase of outer mitochondrial membrane 5	0.924343	1.01822
	TOMM7	translocase of outer mitochondrial membrane 7	0.557692	0.611233
	TP53RK	TP53 regulating kinase	0.565331	0.617696
	TP63	tumor protein p63	1.17284	0.761436
	TPBG	trophoblast glycoprotein	0.510288	0.632264
	TPI1	triosephosphate isomerase 1	0.662608	0.676696
	TPM1	tropomyosin 1 (alpha)	0.801783	0.748702
	TPMT	thiopurine S-methyltransferase	1.40831	1.2533
	TPST1	tyrosylprotein sulfotransferase 1	0.973886	0.823435
	TPST2	tyrosylprotein sulfotransferase 2	1.27295	1.15574
	TPT1	"tumor protein, translationally-controlled 1"	0.512395	0.431892
	TRA2B	transformer 2 beta homolog (Drosophila)	0.93612	1.02773
	TRAF4	TNF receptor associated factor 4	1.40716	1.41039
	TRAFD1	TRAF-type zinc finger domain containing 1	1.11782	1.02991
	TRAM1L1	translocation associated membrane protein 1-like 1	1.03518	1.32007

	TRAP1	TNF receptor associated protein 1	0.620316	0.54511
	TRIAP1	TP53 regulated inhibitor of apoptosis 1	2.00172	2.1629
	TRIB2	tribbles pseudokinase 2	2.8007	2.78841
	TRIB3	tribbles pseudokinase 3	1.04783	1.16733
	TRIM16	tripartite motif containing 16	0.993213	1.01247
	TRIM16L	tripartite motif containing 16-like	0.910647	0.922332
	TRMT10C	"tRNA methyltransferase 10C, mitochondrial RNase P subunit"	0.656297	0.739582
	TRMT12	tRNA methyltransferase 12 homolog	0.867535	0.94512
	TSEN15	tRNA splicing endonuclease subunit 15	0.543937	0.571189
	TSPAN14	tetraspanin 14	0.869488	0.86275
	TSPAN3	tetraspanin 3	0.452084	0.480864
	TSPAN4	tetraspanin 4	1.20445	1.41946
	TSPAN5	tetraspanin 5	1.45388	1.14094
	TSPAN6	tetraspanin 6	0.6999	0.627904
	TSPYL4	TSPY like 4	0.461534	0.620688
	TTC26	tetratricopeptide repeat domain 26	0.645484	0.750698
	TTC27	tetratricopeptide repeat domain 27	0.506052	0.541612
	TTC30B	tetratricopeptide repeat domain 30B	0.431373	0.372918
	TTC5	tetratricopeptide repeat domain 5	0.895979	0.934807
	TTLL1	tubulin tyrosine ligase like 1	0.686081	0.387382
	TTYH2	tweety family member 2	1.39606	1.47698
	TTYH3	tweety family member 3	1.29448	1.25873
	TUB	tubby bipartite transcription factor	1.14111	0.899701
	TUBA1A	tubulin alpha 1a	1.22901	1.05345
	TUBB2A	tubulin beta 2A class IIa	0.556047	0.69846
	TUBD1	tubulin delta 1	1.66967	1.62302
	TUFM	"Tu translation elongation factor, mitochondrial"	0.445132	0.450125
	TUSC1	tumor suppressor candidate 1	0.434468	0.671859
	TUSC3	tumor suppressor candidate 3	0.476439	0.443338
	TXNDC15	thioredoxin domain containing 15	0.674132	0.804971
	TXNDC16	thioredoxin domain containing 16	1.2185	0.912998
	TXNL4A	thioredoxin like 4A	0.436729	0.51071
	TXNRD3NB	thioredoxin reductase 3 neighbor	1.05308	1.27236
	TYMS	thymidylate synthetase	0.594336	0.609152
	UBAC1	UBA domain containing 1	0.517836	0.514537
	UBAC2	UBA domain containing 2	0.761043	0.832168
	UBE2E1	ubiquitin conjugating enzyme E2 E1	0.619358	0.605514
	UBE2F	ubiquitin conjugating enzyme E2 F (putative)	0.847813	0.799021
	UBE2T	ubiquitin conjugating enzyme E2 T	0.641735	0.668278
	UBE2V2	ubiquitin conjugating enzyme E2 V2	0.847768	0.761367
	UBL3	ubiquitin like 3	0.578444	0.397981
	UBL4A	ubiquitin like 4A	0.412535	0.524187
	UBL5	ubiquitin like 5	0.372425	0.448646
	UBLCP1	ubiquitin like domain containing CTD phosphatase 1	0.457536	0.390483
	UBQLN2	ubiquilin 2	0.454439	0.36814
	UCHL3	ubiquitin C-terminal hydrolase L3	0.62191	0.608182
	UCK2	uridine-cytidine kinase 2	0.9374	1.01923
	UFSP2	UFM1 specific peptidase 2	0.918514	0.915898
	UGT2B15	UDP glucuronosyltransferase family 2 member B15	1.18061	1.32403
	ULBP2	UL16 binding protein 2	1.14969	1.02138
	UMPS	uridine monophosphate synthetase	0.618575	0.654156
	UNC119B	unc-119 lipid binding chaperone B	0.57547	0.632513
	UNG	uracil DNA glycosylase	0.816379	0.778265
	UPK3BL	uroplakin 3B-like	0.806669	1.01034
	UQCR10	"ubiquinol-cytochrome c reductase, complex III subunit X"	0.440544	0.643124
	UQCRC2	ubiquinol-cytochrome c reductase core protein II	0.756596	0.79963
	UQCRFS1	"ubiquinol-cytochrome c reductase, Rieske iron-sulfur polypeptide 1"	0.673997	0.820654
	UQCRH	ubiquinol-cytochrome c reductase hinge protein	0.730635	0.787734
	UQCRQ	ubiquinol-cytochrome c reductase complex III subunit VII	0.655835	0.735397
	URB1-AS1	URB1 antisense RNA 1 (head to head)	0.768621	1.27432
	URM1	ubiquitin related modifier 1	0.777266	0.915202
	UROD	uroporphyrinogen decarboxylase	0.562775	0.612601

	USP38	ubiquitin specific peptidase 38	0.48234	0.389678
	UTP18	"UTP18, small subunit processome component"	0.537868	0.60794
	UTP3	"UTP3, small subunit processome component homolog (S. cerevisiae)"	0.539582	0.683986
	UTP6	"UTP6, small subunit processome component"	0.626186	0.711473
	VCPKMT	valosin containing protein lysine methyltransferase	0.605394	0.87448
	VDAC3	voltage dependent anion channel 3	0.599311	0.583252
	VIPR1	vasoactive intestinal peptide receptor 1	0.945101	0.763107
	VPS25	vacuolar protein sorting 25 homolog	1.09281	1.16585
	VSTM2L	V-set and transmembrane domain containing 2 like	2.51467	2.41676
	VTA1	vesicle trafficking 1	1.32726	1.38368
	VTCN1	V-set domain containing T cell activation inhibitor 1	1.77544	1.75288
	VWC2L	von Willebrand factor C domain containing protein 2-like	3.02552	2.7854
	WASF1	WAS protein family member 1	1.0033	0.791938
	WBP1	WW domain binding protein 1	0.580605	0.575147
	WBSCR22	Williams-Beuren syndrome chromosome region 22	0.693725	0.809007
	WDR12	WD repeat domain 12	0.631358	0.671058
	WDR18	WD repeat domain 18	0.938405	1.11267
	WDR41	WD repeat domain 41	0.607608	0.536312
	WDR5	WD repeat domain 5	0.458789	0.498514
	WDR61	WD repeat domain 61	0.783432	0.865257
	WDR72	WD repeat domain 72	0.705664	0.610389
	WDR75	WD repeat domain 75	0.497876	0.500219
	WDR82	WD repeat domain 82	0.498426	0.52336
	WISP2	WNT1 inducible signaling pathway protein 2	2.80975	2.83438
	WNT10B	Wnt family member 10B	0.553471	1.08361
	WNT6	Wnt family member 6	0.563029	1.08002
	WNT8B	Wnt family member 8B	1.5311	2.08131
	WRNIP1	Werner helicase interacting protein 1	0.463639	0.461986
	XRCC2	X-ray repair cross complementing 2	0.497408	0.37365
	YAE1D1	Yae1 domain containing 1	0.528961	0.833542
	YBX1	Y-box binding protein 1	0.608175	0.510208
	YBX3	Y-box binding protein 3	1.38701	1.41547
	YEATS4	YEATS domain containing 4	0.743376	0.965598
	YIF1B	"Yip1 interacting factor homolog B, membrane trafficking protein"	0.721536	0.907742
	YRDC	yrdC N6-threonylcarbamoyltransferase domain containing	0.756552	0.92334
	YWHAE	tyrosine 3-monooxygenase/tryptophan 5-monooxygenase activation protein epsilon	0.487217	0.457738
	YWHAG	tyrosine 3-monooxygenase/tryptophan 5-monooxygenase activation protein gamma	0.868706	0.950922
	YY1	YY1 transcription factor	0.656623	0.652961
	ZBTB5	zinc finger and BTB domain containing 5	0.434602	0.518307
	ZCCHC10	zinc finger CCHC-type containing 10	0.96443	1.18733
	ZDHHC12	zinc finger DHHC-type containing 12	0.720658	1.1442
	ZFP91	ZFP91 zinc finger protein	0.636921	0.52485
	ZIC5	Zic family member 5	0.519752	0.454947
	ZKSCAN4	zinc finger with KRAB and SCAN domains 4	0.541057	0.569274
	ZMAT4	zinc finger matrin-type 4	1.53596	1.73342
	ZMYM5	zinc finger MYM-type containing 5	0.458967	0.56089
	ZMYND19	zinc finger MYND-type containing 19	1.05418	1.24949
	ZNF132	zinc finger protein 132	0.974712	0.960395
	ZNF212	zinc finger protein 212	0.573246	0.799225
	ZNF22	zinc finger protein 22	1.10956	1.22216
	ZNF286A	zinc finger protein 286A	0.765241	0.59238
	ZNF32	zinc finger protein 32	0.790834	0.846533
	ZNF346	zinc finger protein 346	1.50192	1.26861
	ZNF519	zinc finger protein 519	0.57224	0.698319
	ZNF584	zinc finger protein 584	0.928914	0.934971
	ZNF674-AS1	ZNF674 antisense RNA 1 (head to head)	0.734379	1.08159
	ZNF711	zinc finger protein 711	0.597437	0.462778
	ZNF77	zinc finger protein 77	0.396748	0.416536
	ZNRD1	zinc ribbon domain containing 1	0.479523	0.696981
	ZP3	zona pellucida glycoprotein 3	0.598498	0.657539

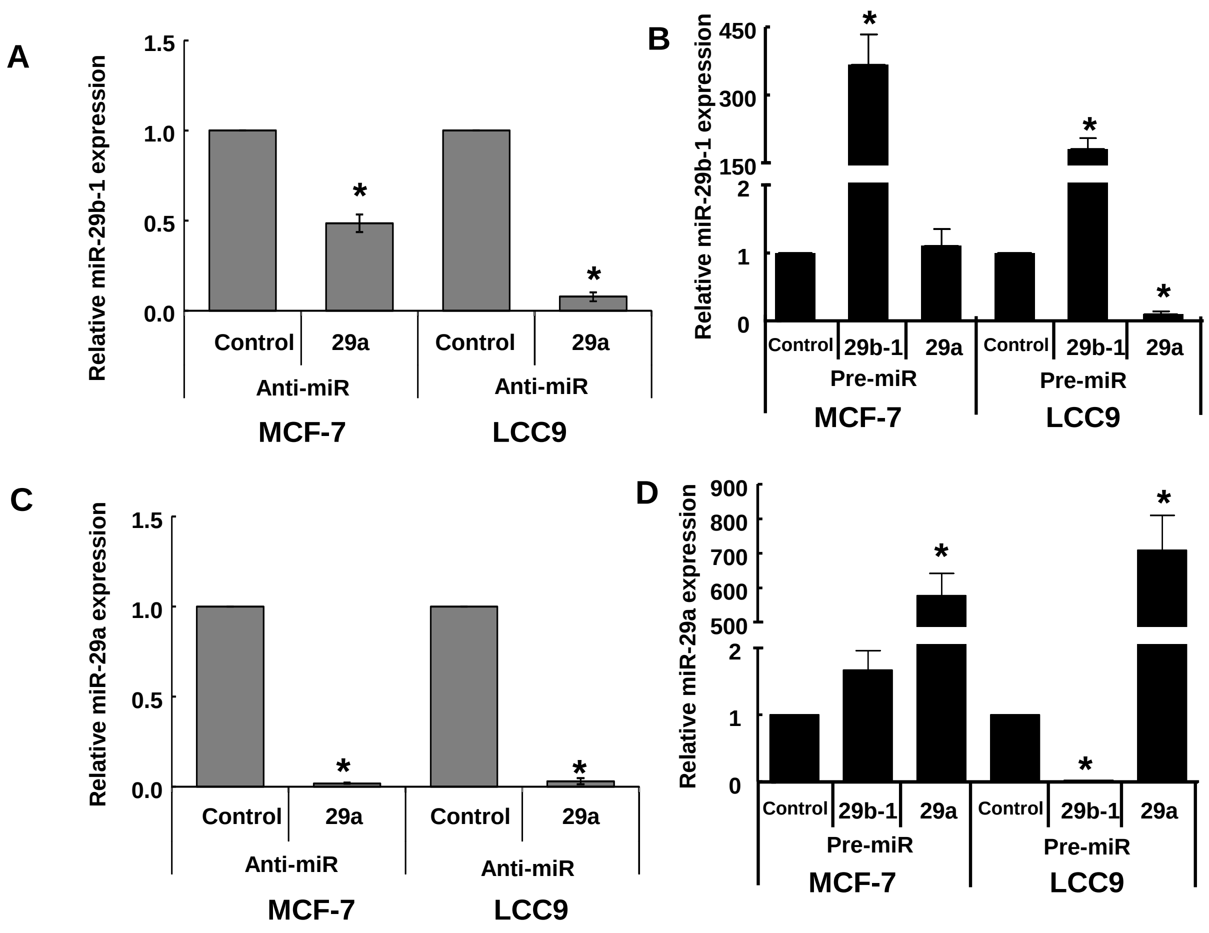

Supplementary Figure 1. Successful knockdown and upregulation of miR-29b-1 /a in MCF-7 and LCC9 cells.
Cells were grown in hormone-depleted media and transfected with anti-miR-control, anti-miR-29a (3p), pre-miR control or pre-miR-29b-1/a (3p) as indicated. RNA was extracted 48 h post transfection and qPCR performed. Values were normalized to RNU6B. Values are the mean ± SEM of 3 independent experiments. Within each experiment, each sample was run in triplicate. *p<0.05 *versus* control transfected cells. Statistical evaluation was performed using one-way ANOVA followed by Newman-Keuls Multiple Comparison Test.

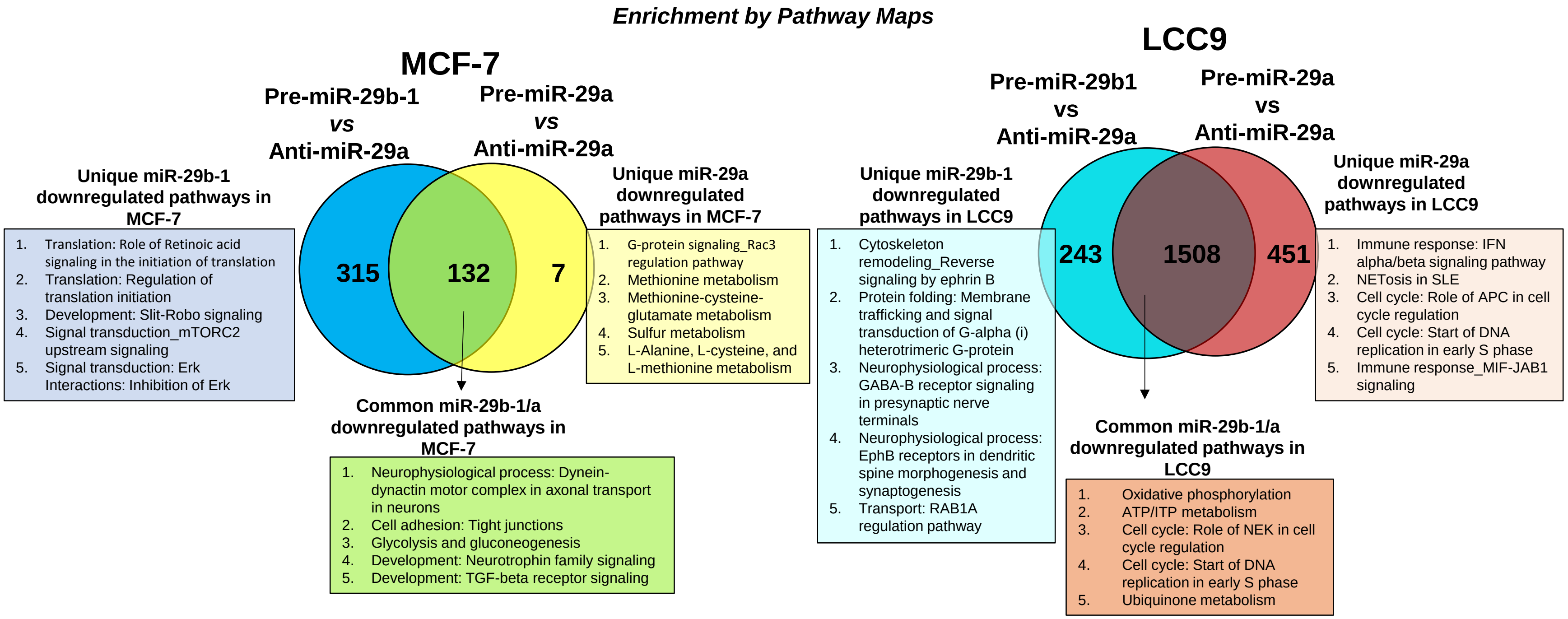

Supplementary Figure 2. Enrichment analysis of RNA-seq data in MCF-7 and LCC9 cells. Data are the same as Fig. 1A and B with an inclusion of pathways identified. The Venn diagrams show the number of differentially expressed genes identified in pairwise comparisons: Pre-miR-29b-1 *vs.* Anti-miR-29a and Anti-miR-29a *vs.* Pre-miR-29a using tophat and cufflink-cuff diff2. GeneGo Pathways Software (MetaCore^{TM}) was then used to obtain the number of common and uniquely expressed genes significantly downregulated by miR-29b-1 and miR-29a in A) MCF-7 and B) LCC9 cells. The pathways identified for each comparison are listed in the order provided by MetaCore^{TM} analysis.

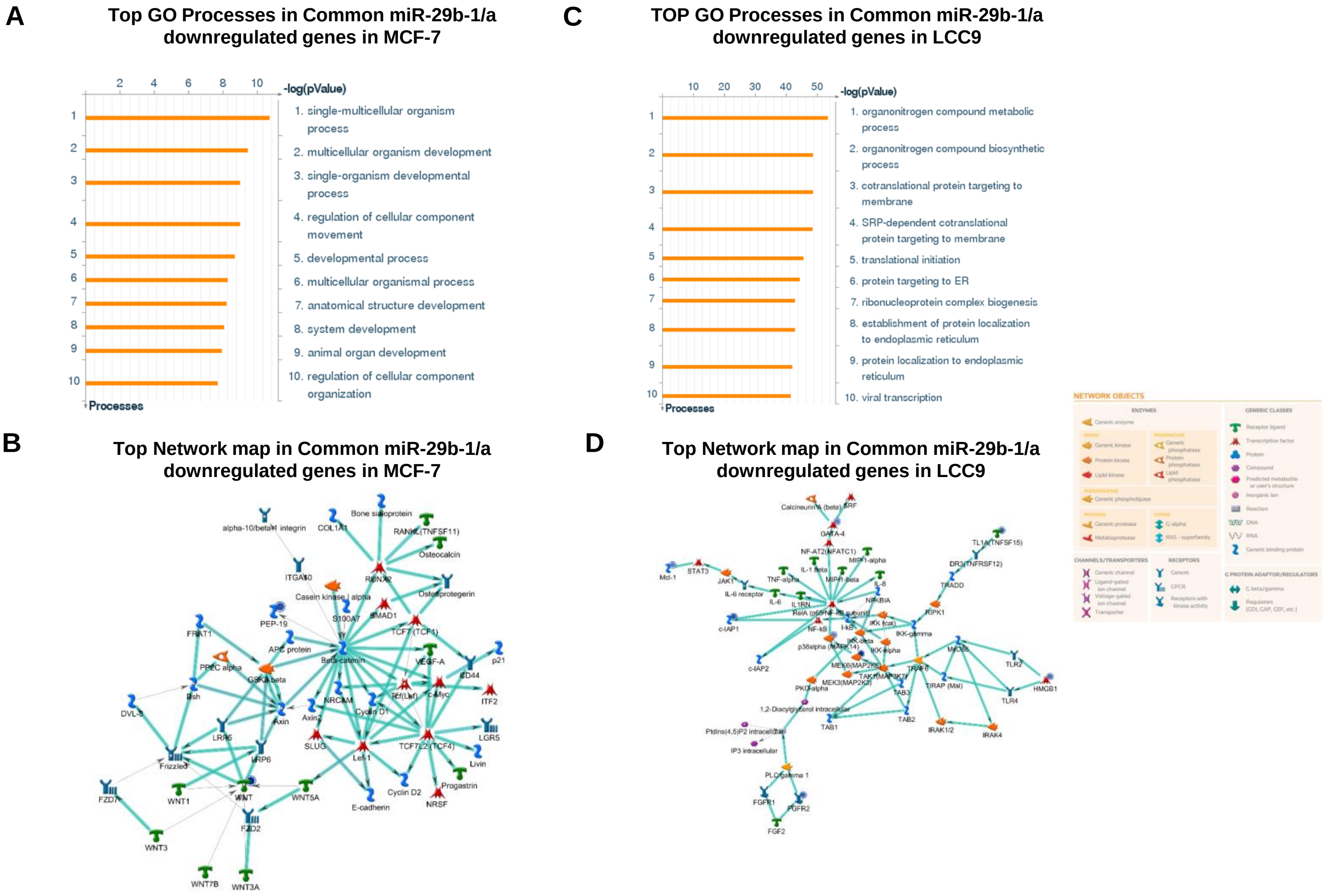

Supplementary Figure 3. Enrichment onthologies of genes regulated by miR-29b-1/a in MCF-7 and LCC9 cells. A, C) Gene Ontology (GO) cellular processes. B, D) The top scored GO network processes determined using Analyze Networks algorithm with default settings in MetaCore™. Thick cyan lines indicate canonical pathways. Items with blue circles indicate downregulated genes.

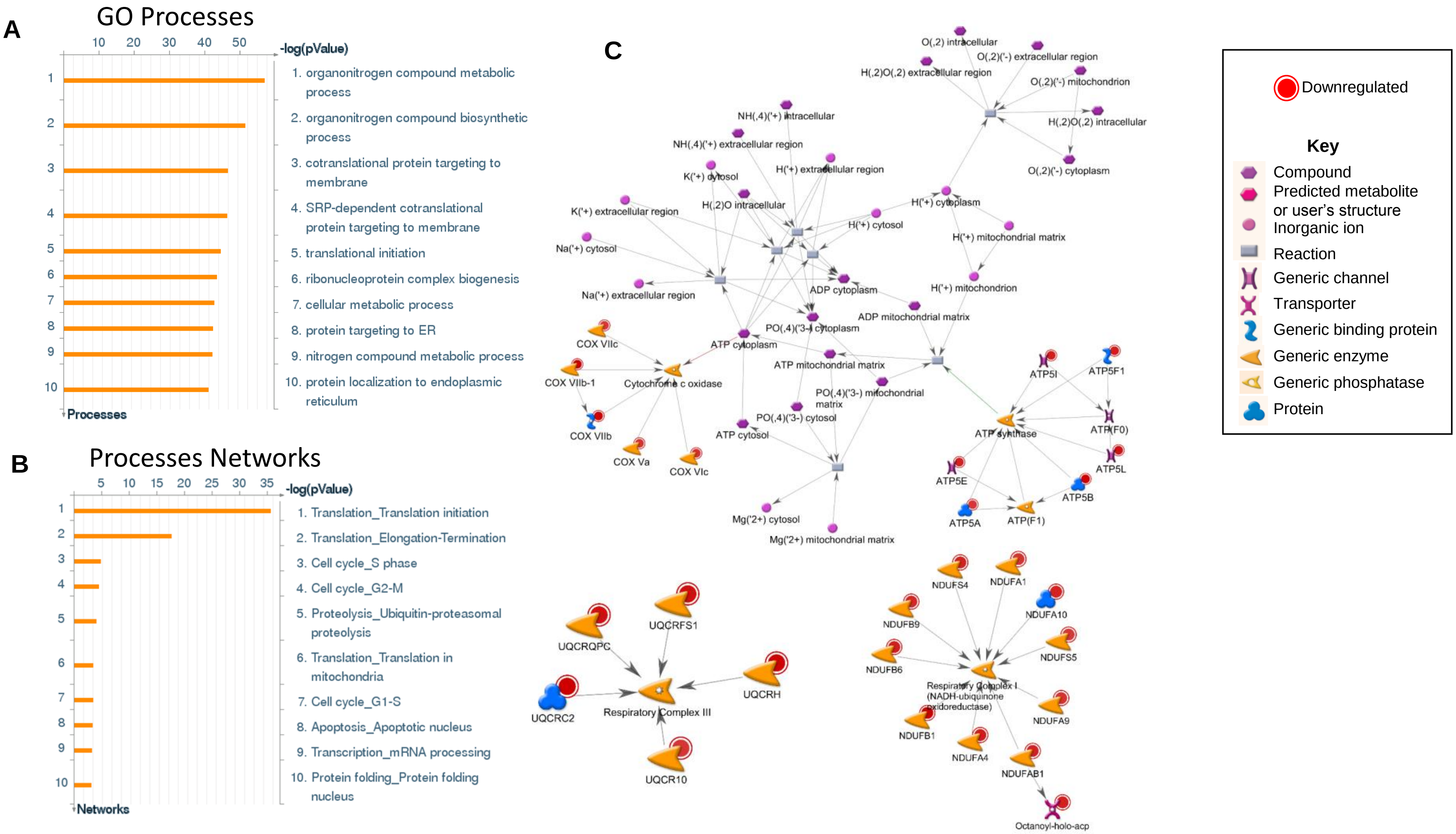

Supplementary Figure 4. Enrichment onthologies of genes uniquely downregulated in LCC9 cells. Differentially expressed genes unique to LCC9 cells identified in pairwise comparisons using cufflink-cuff diff2. MetaCore™ was then used to determine A) top Gene Ontology (GO) cellular processes and B) top Process Networks. C) Network analysis of genes mediating mitochondrial ATP synthesis coupled proton transport. miR-29 downregulated target genes are indicated by the red circle

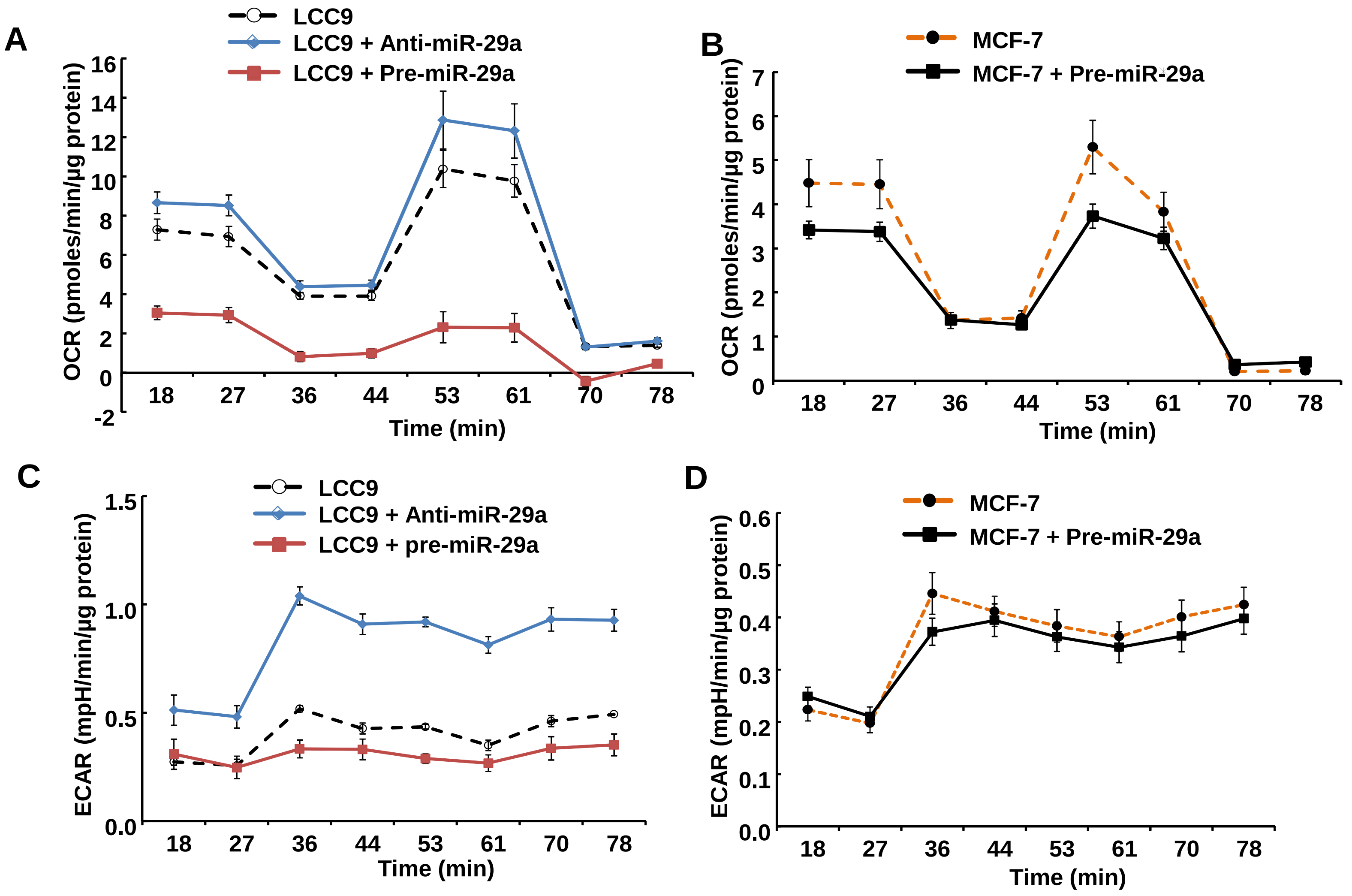

Supplementary Figure 5. miR-29a regulates mitochondrial function of MCF-7 and LCC9 BC cells. MCF-7 and LCC9 cells were plated in XF-24 plates, 'hormone-deprived' and transfected with anti-miR-29a or pre-miR-29a, versus control, as indicated, for 28 h prior to running extracellular flux assay to determine mitochondrial activity. Each point is the avg of 4 separate wells ± SEM.

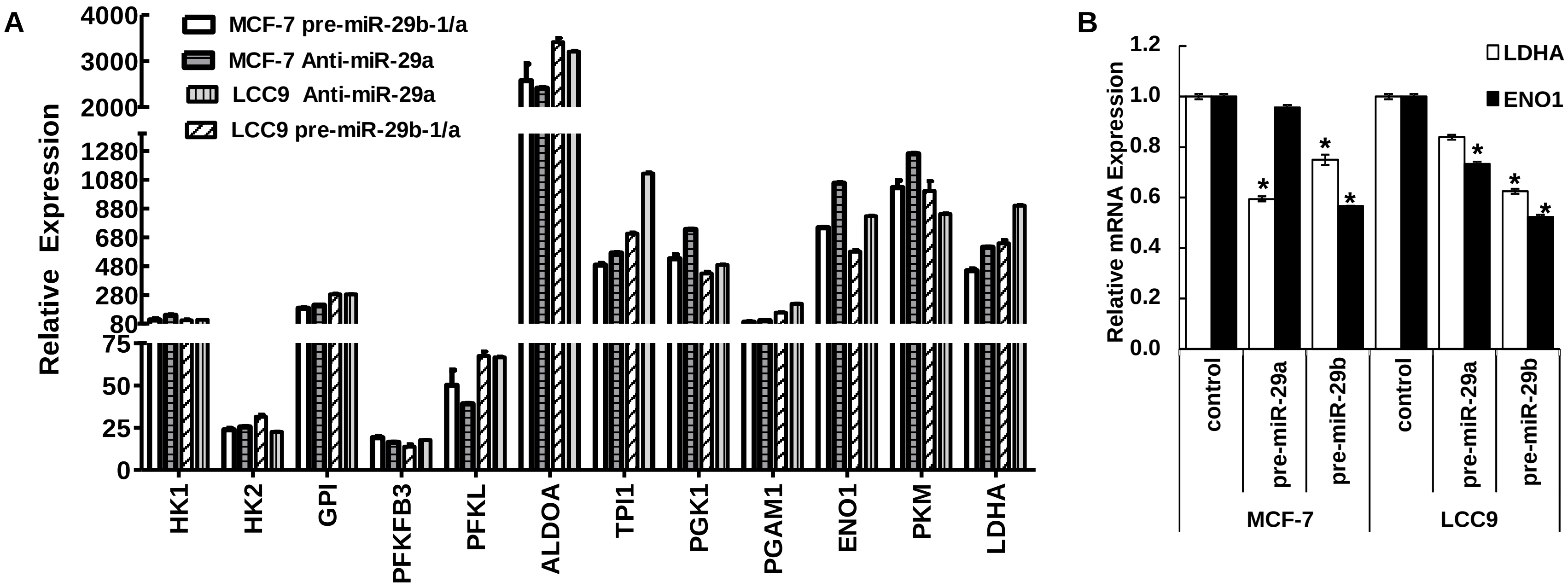

Supplementary Figure 6. Glycolytic gene expression in MCF-7 and LCC9 BC cells. MCF-7 and LCC9 cells were 'hormone-deprived' and transfected with pre-miR-29a, pre-miR-29b-1, or anti-miR-29a. A) Values are the FPKM (Fragments Per Kilobase of transcript per Million mapped reads) from GSE81620 RNAseq dataset. Bars for pre-miR-29b-1/a are the average of 6 separate experiments and bars with Anti-miR-29a are the average of 3 experiments. B) qPCR of LDHA and ENO1 mRNA transcript expression in MCF-7 and LCC9 cells transfected as indicated. Values are the mean ± SEM of 3 samples in one experiment. *p<0.05 *versus* control transfected cells. Statistical evaluation was performed using Student's t test.

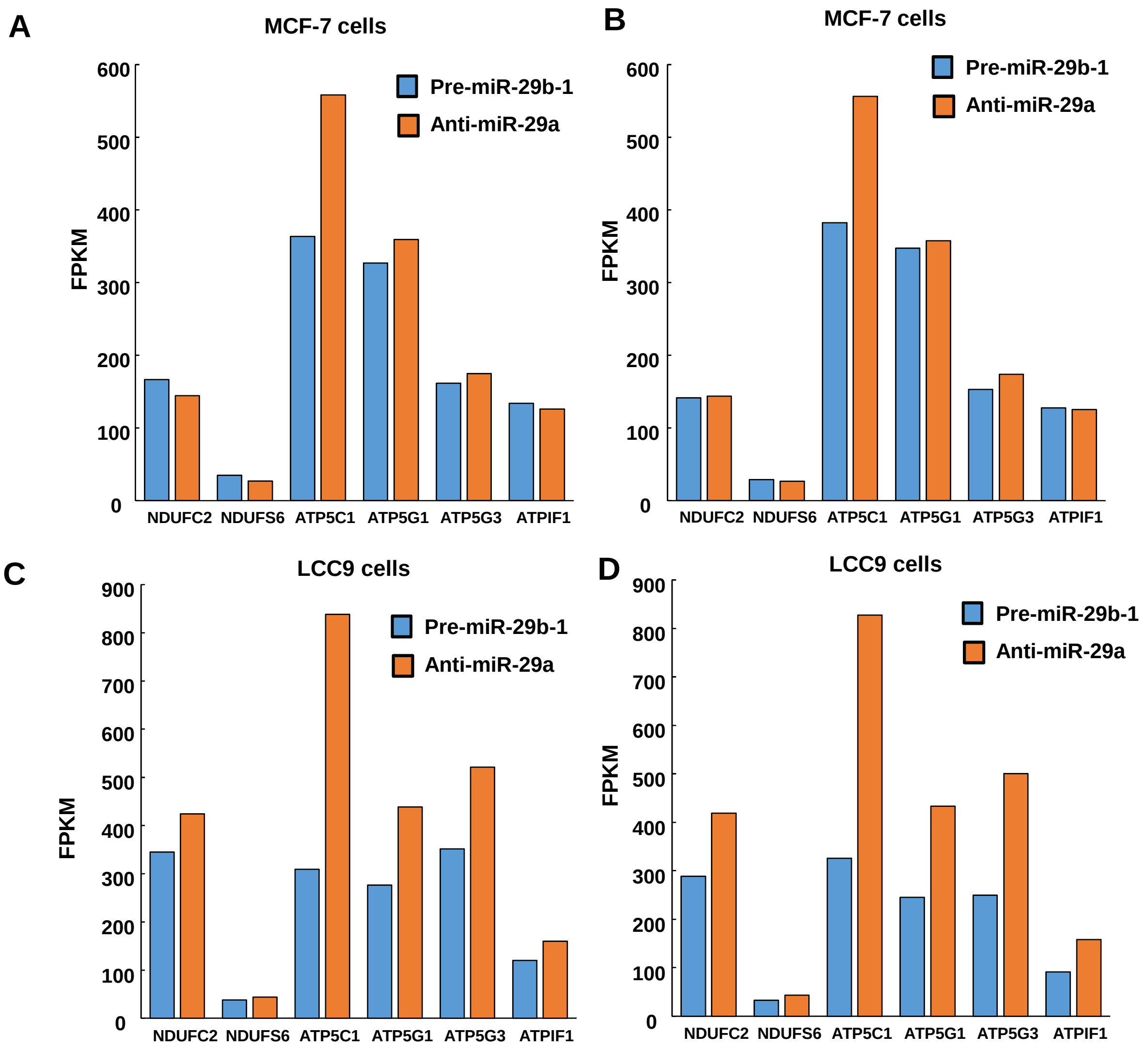

Supplementary Figure 7. RNA-seq expression profiles of *ATP5G1*, *ATP5C1*, *ATPIF1*, *ATP5G3*, *NDUFS6* and *NDUFC2* in MCF-7 and LCC9 breast cancer cells. Values are FPKM (Fragments Per Kilobase of transcript per Million mapped reads) from GSE81620 RNAseq dataset. Each bar is the average of 3 separate experiments.

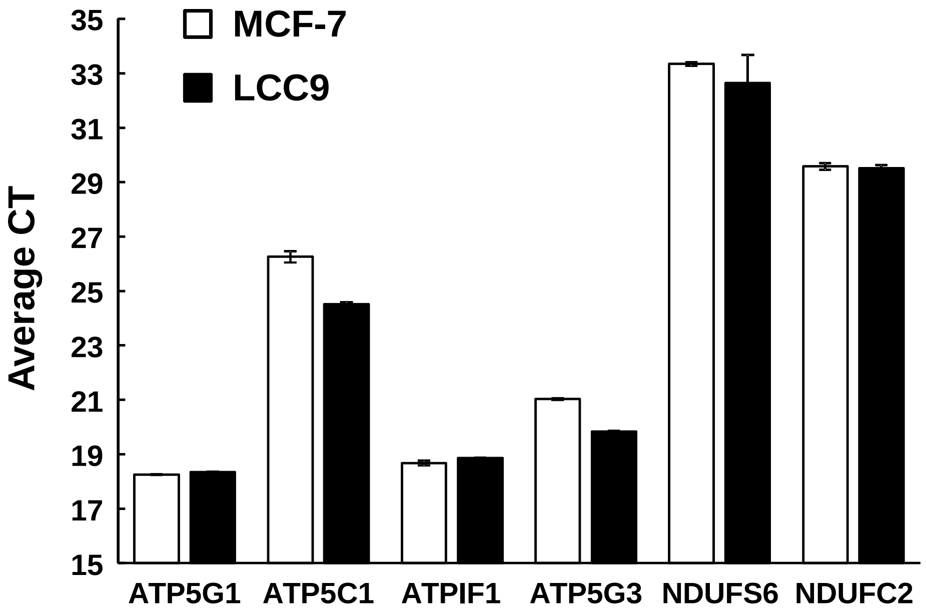

Supplementary Figure 8: Basal expression levels of putative miR-29b-1/a targets in breast cancer cells. MCF7 and LCC9 cells were grown in 'hormone-depleted' media for 48 h. RNA was isolated and qPCR performed to determine expression levels. Each bar is the avg. ± SEM of triplicate samples in one experiment.

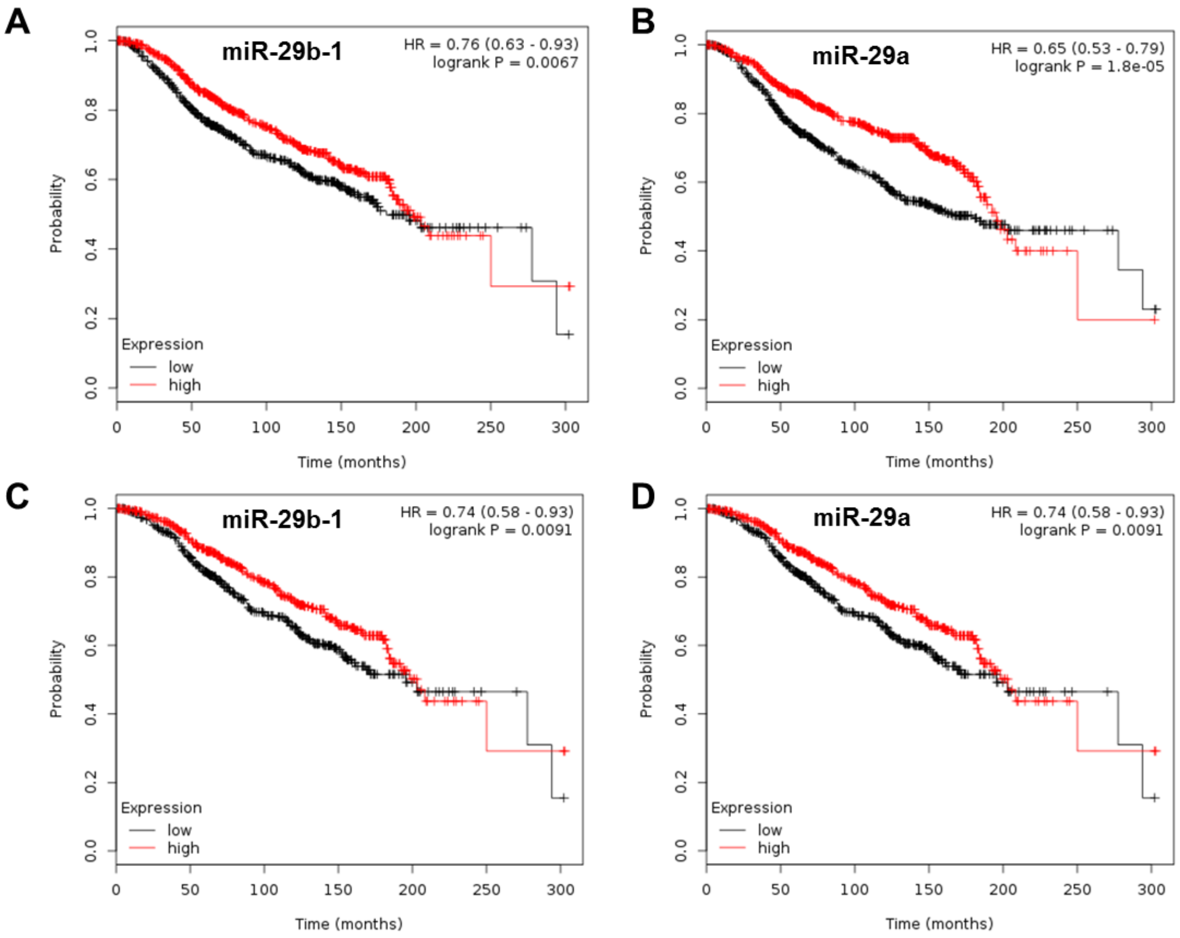

Supplementary Figure 9: Lower expression of miR-29b-1 and miR-29a is statistically associated with decreased relapse-free survival (RFS) in all breast cancer and in patients whose primary tumors are ERα+. Kaplan-Meiera nalysis was performed in miRPower for breast cancer (http://kmplot.com/analysis/index.php?p=service&cancer=breast_mirna). A) miR-29b-1 and B) miR-29a expression in all breast tumors (n = 1262). C) miR-29b-1 and D) miR-29a in ERα+ primary breast tumors (n = 996). All four plots show that lower miR-29b-1 and miR-29a expression is associated with lower RFS.

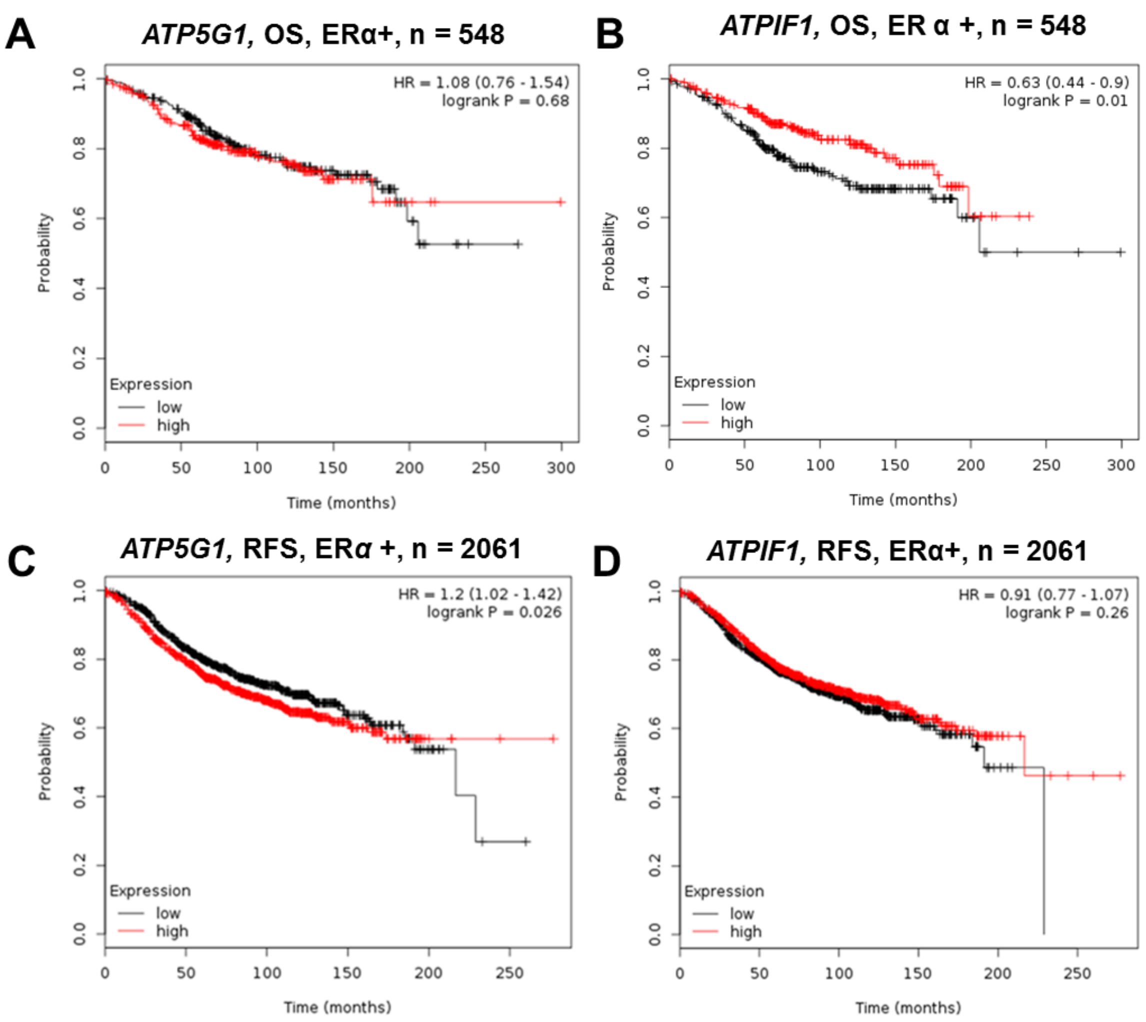

Supplementary Figure 10: Association of ATP5G1 and ATPIF1 transcript levels with Overall Survival (OS) and Relapse Free Survival (RFS) from ERα+ breast tumor data. Analysis was performed in Kaplan Meier Plotter for breast cancer (http://kmplot.com/analysis/index.php?p=service&cancer=breast). A and B are OS for ATP5G1 and ATPIF1, respectively. C and B are RFS for ATP5G1 and ATPIF1, respectively. P values are indicated. B and D are statistically significant ($P < 0.05$). n = number of breast tumors.

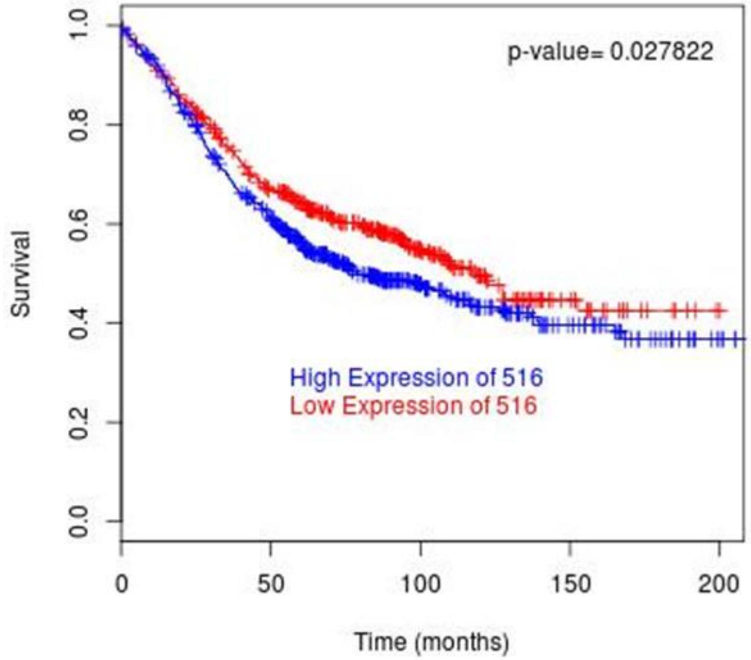

Supplementary Figure 11: Association of ATP5G1 expression and Disease Free Survival (DFS) in luminal B breast tumors. Analysis was performed for ATP5G1 transcript expression in http://glados.ucd.ie/BreastMark/mRNA_analysis.html. N = 1013.
Hazard ratio = = 1.228 (1.022 - 1.474)
Score (logrank) test = 4.84 on 1 df, p=0.02778. Higher ATP5G1 transcript levels are associated with reduced DFS in patients with primary luminal B breast tumors.

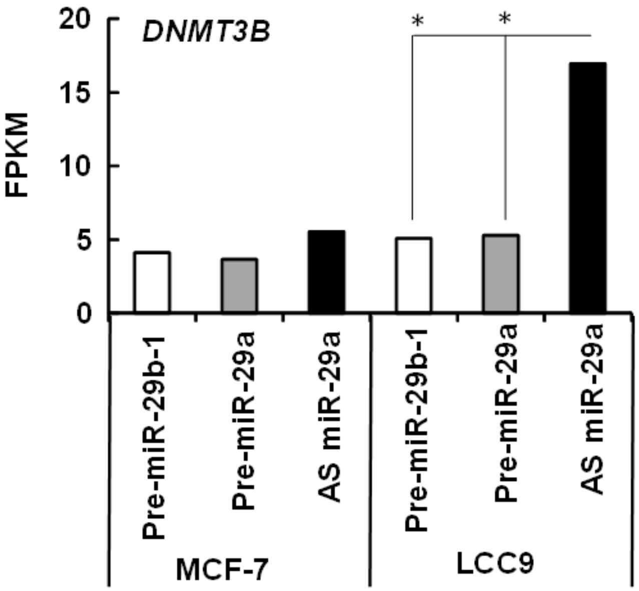

Supplementary Figure 12. ***DNMT3B* expression in MCF-7 and LCC9 cells.** Data are FPKM and are the avg. of three separate experiments. * $p > 0.00005$.

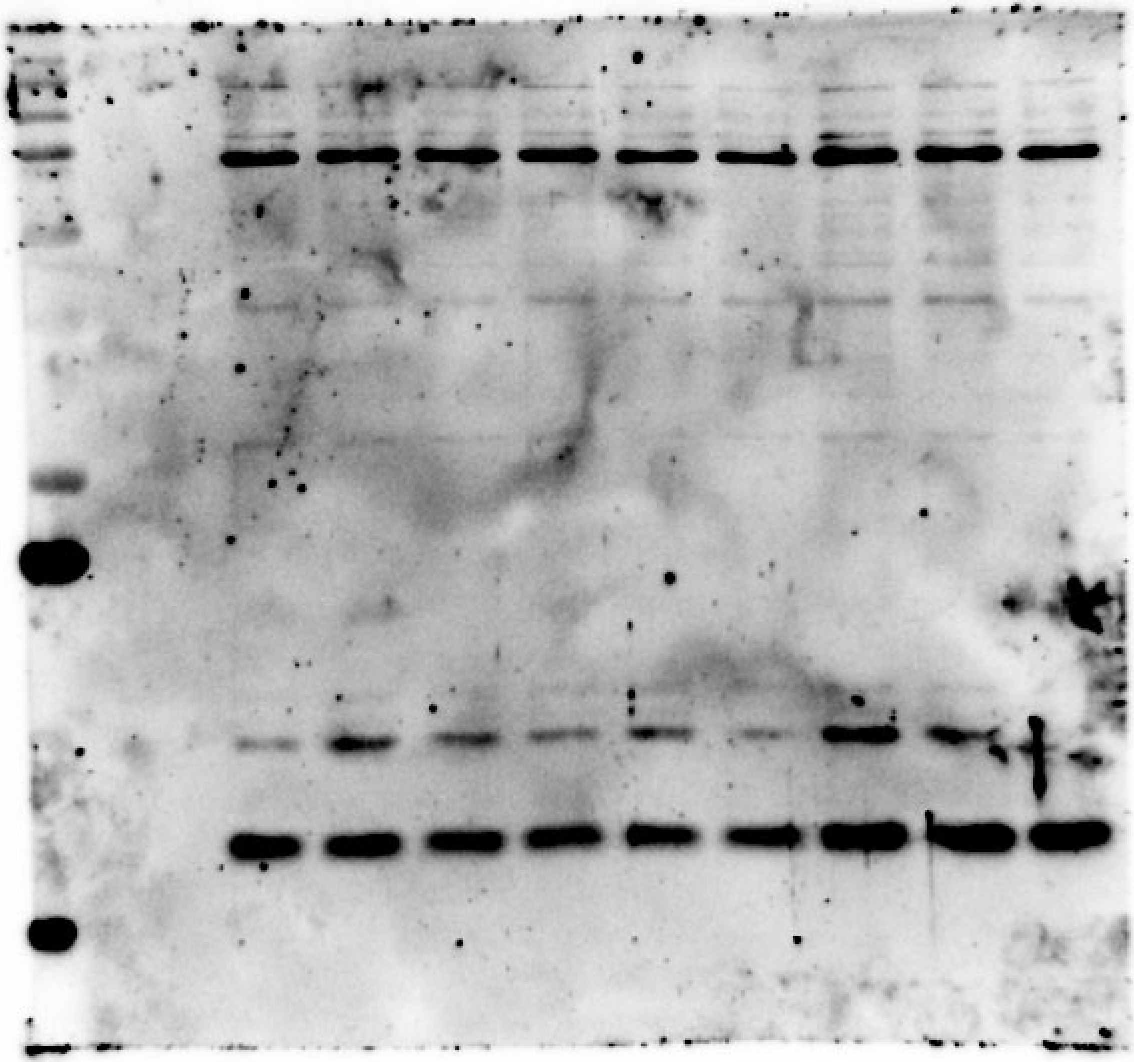

Supplementary Figure 13

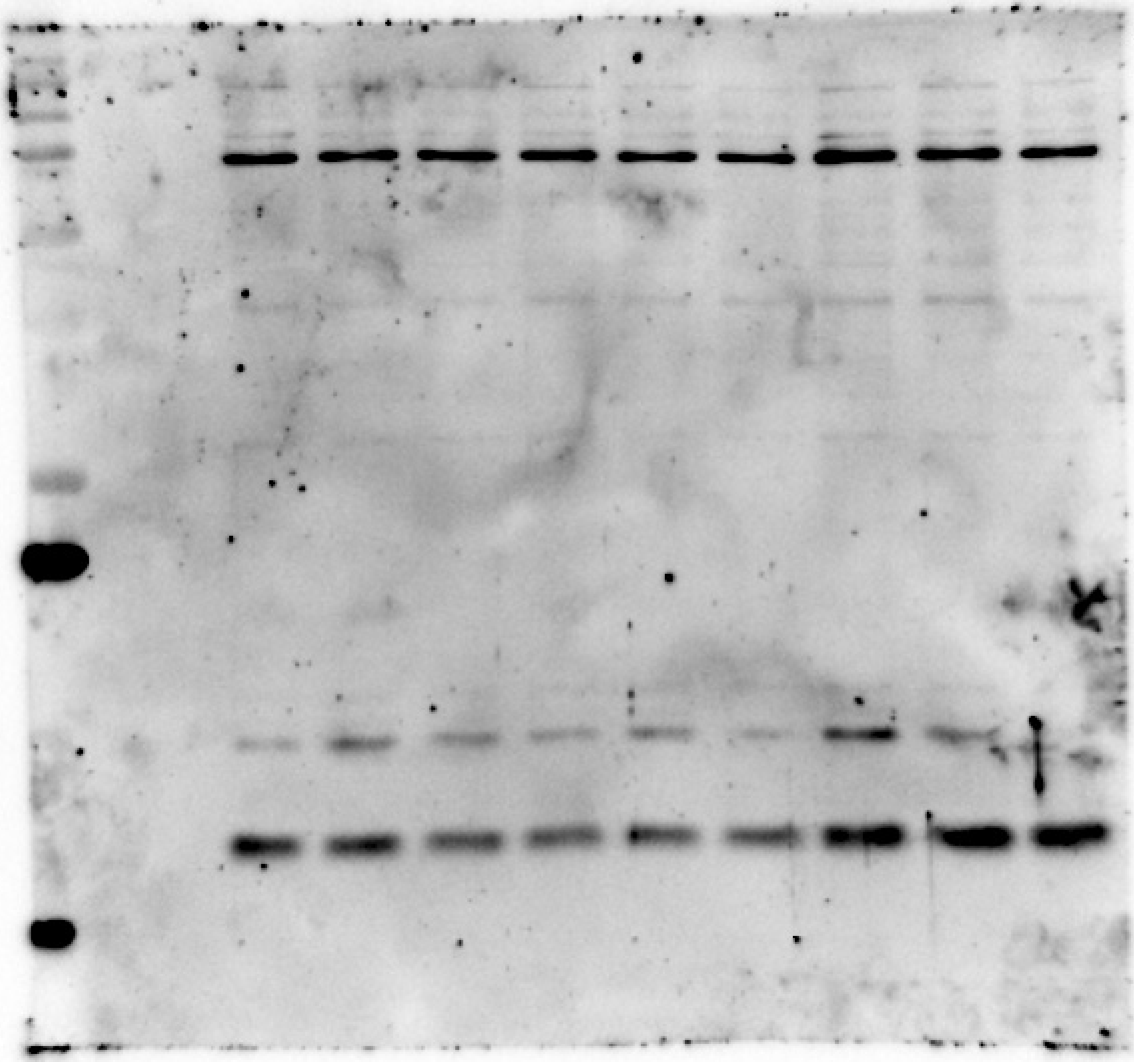

Supplementary Figure 14

Supplementary Figure 15

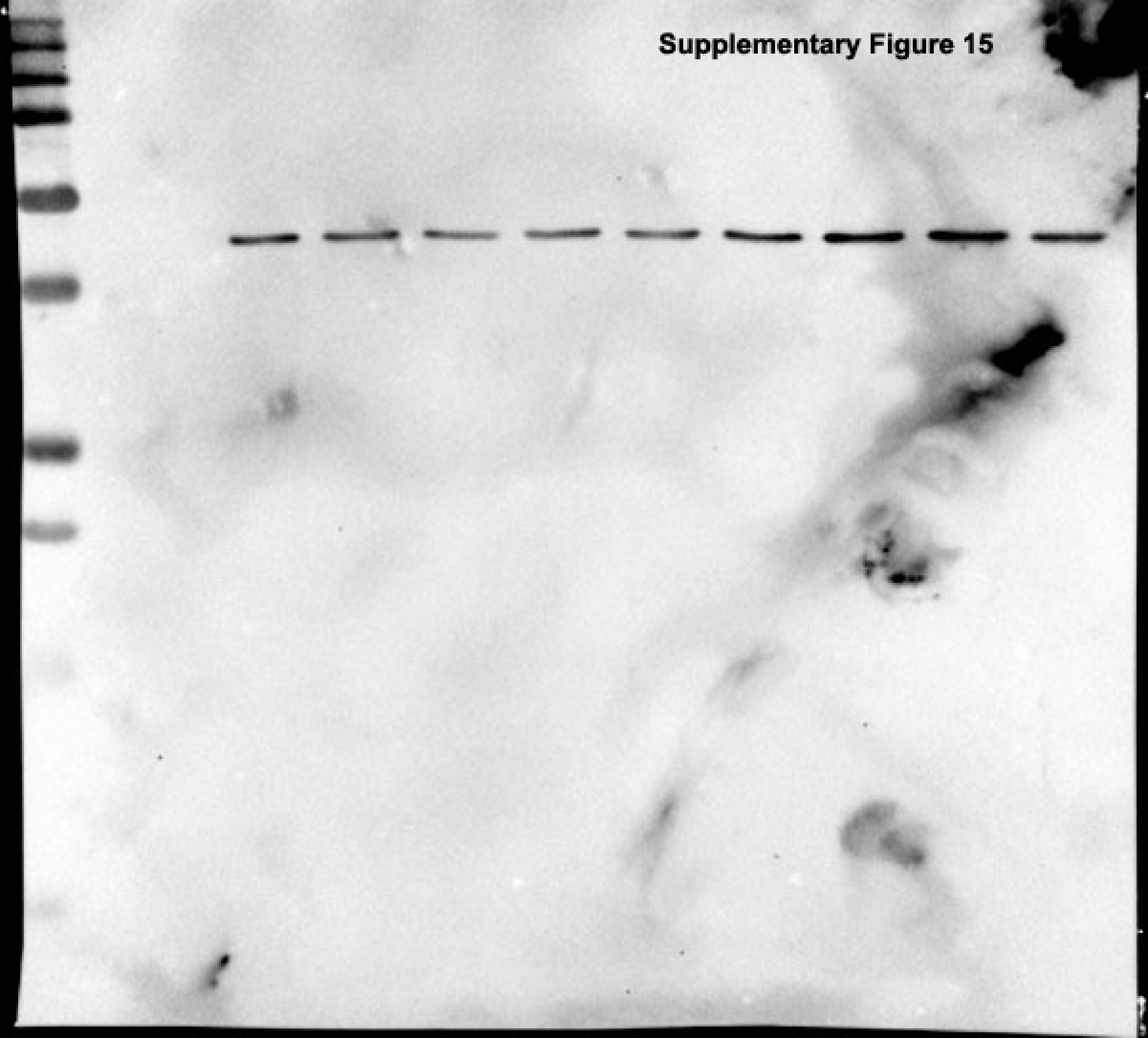

Supplement: Supplementary file 1 — Supplementary Tables and Figures [file 41598_2017_5727_MOESM1_ESM.pdf]
